# Synonymous codon bias and functional constraint on GC3-related DNA backbone dynamics in the prokaryotic nucleoid

**DOI:** 10.1093/nar/gku811

**Published:** 2014-09-08

**Authors:** Gregory A. Babbitt, Mohammed A. Alawad, Katharina V. Schulze, André O. Hudson

**Affiliations:** 1 Thomas H. Gosnell School of Life Sciences, Rochester Institute of Technology, Rochester NY, USA 14623; 2 B. Thomas Golisano College of Computing and Information Sciences, Rochester Institute of Technology, Rochester NY, USA 14623; 3 Department of Molecular and Human Genetics, Baylor College of Medicine, Houston TX, USA 77030

## Abstract

While mRNA stability has been demonstrated to control rates of translation, generating both global and local synonymous codon biases in many unicellular organisms, this explanation cannot adequately explain why codon bias strongly tracks neighboring intergene GC content; suggesting that structural dynamics of DNA might also influence codon choice. Because minor groove width is highly governed by 3-base periodicity in GC, the existence of triplet-based codons might imply a functional role for the optimization of local DNA molecular dynamics via GC content at synonymous sites (≈GC3). We confirm a strong association between GC3-related intrinsic DNA flexibility and codon bias across 24 different prokaryotic multiple whole-genome alignments. We develop a novel test of natural selection targeting synonymous sites and demonstrate that GC3-related DNA backbone dynamics have been subject to moderate selective pressure, perhaps contributing to our observation that many genes possess extreme DNA backbone dynamics for their given protein space. This dual function of codons may impose universal functional constraints affecting the evolution of synonymous and non-synonymous sites. We propose that synonymous sites may have evolved as an ‘accessory’ during an early expansion of a primordial genetic code, allowing for multiplexed protein coding and structural dynamic information within the same molecular context.

## INTRODUCTION

The nature of codon usage bias and its relation to natural selection acting at synonymous sites is a long-standing problem in molecular evolutionary theory (1,2). Since the inception of the field of molecular evolution, its founders have postulated the existence of purely neutrally evolving or ‘isosemantic’ substitutions within genomes (3–5). However, early observations of universal evolutionary changes to GC content (6) and a strong association between preferred synonymous codons and their relative representation in the t-RNA pool (7,8) has also long suggested that completely neutrally evolving base substitutions may exist only in theory, rather than fact. Zuckerkandl and Pauling (3), citing Itano (9), were also careful to remark that substitutions failing to affect amino acids might still lead to altered rates of protein synthesis, and noted that amino acids occurring with high frequency also tend to be represented by more codons, thus facilitating degeneracy in the code that might additionally facilitate functional variation in unknown ways.

While it is now clear that natural selection acts on synonymous codons (10,11), the exact molecular traits upon which this selection acts is still the topic of a large amount of speculation (reviewed in (2,12)). Recently, Drummond and Wilke (13) have invoked selection against protein misfolding due to translational error as a universal pressure underlying codon bias. While there is now good evidence in many microbes for selection acting on the efficiency/accuracy of translation, there is still no explanation as to why codon bias so strongly tracks the local intergenic GC content in the genomes of most other organisms (14,15); especially as it is hard to imagine how molecular evolution in the intergenic regions can affect the molecular events on the ribosome. Biased gene conversion, a process limited to sexually recombining genomes, has been previously invoked as an explanation (16), however it is not a universal explanation given the taxonomic limitations of this phenomena. Equally puzzling is the recent discovery of many tandem substitution events localized to adjacent synonymous and non-synonymous sites (17) suggesting that evolutionary forces acting on these neighboring sites are not as independent as has often been historically assumed. Intriguingly, a recent experiment in *Methylobacterium extorquens* demonstrated that synonymous mutations toward globally favored frequent codons in the genome, which would normally be thought to enhance translational efficiency, actually dramatically decrease Darwinian fitness in this bacterium (18), again suggesting that codon bias may not always be governed only by selection to globally optimize translation rates.

**Figure 1.**
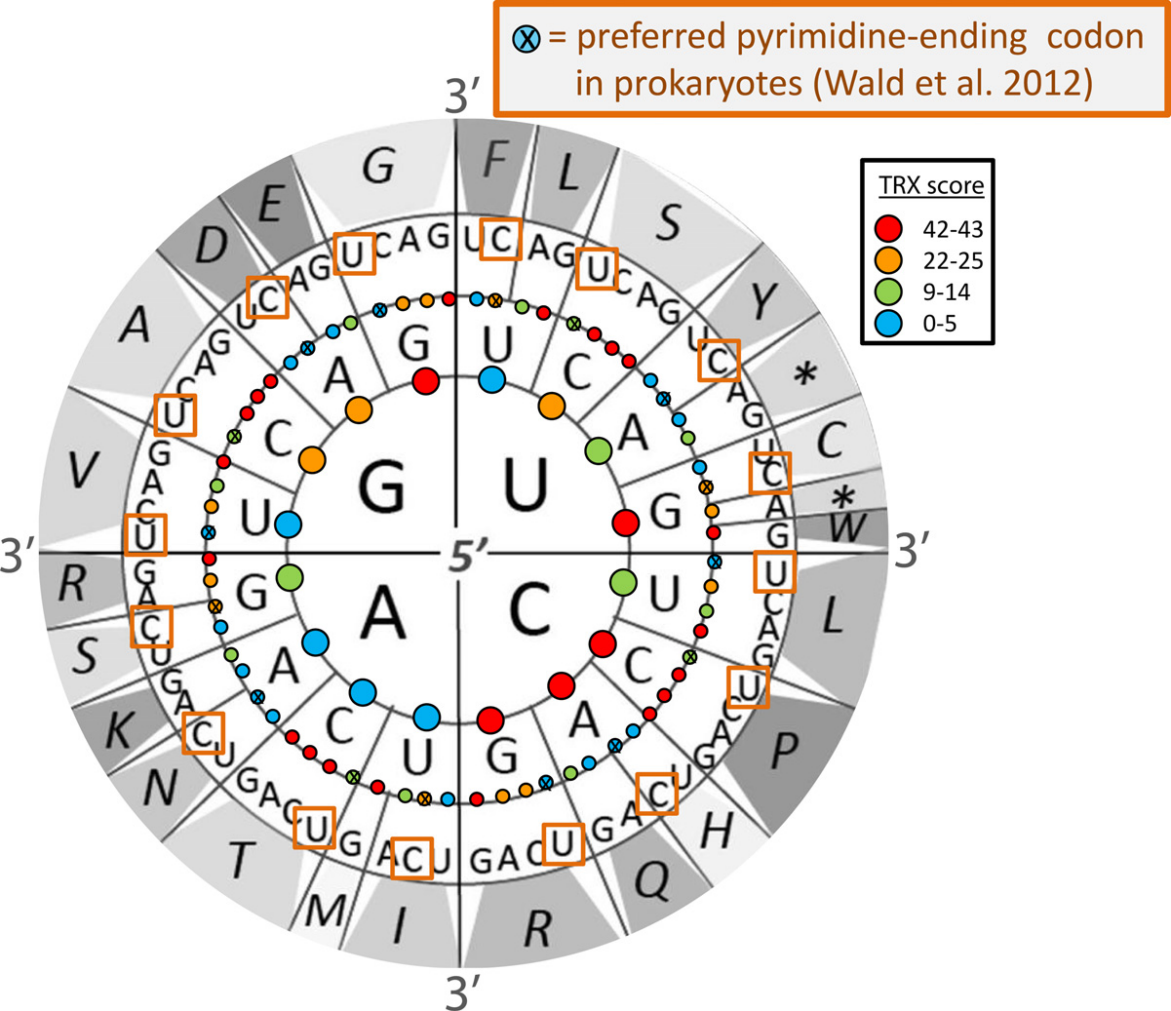
Internal phosphate linkages of codons assigned by the standard genetic code are color-coded according to their fixed levels of intrinsic DNA flexibility. The intrinsic flexibility of external phosphate linkages, located at the center and outer edge of the white circle, are variable across genes and genomes and are determined through adjacent codon usage patterns. Adapted from (28) and (29).

The highly non-random organization of codon assignment with respect to mutational impact on protein hydrophobicity is now a widely accepted evidence for an ancient history of purifying selection acting on various genetic coding schemes (19,20) and thus supporting a view that the code is a fixed or ‘frozen adaptation’ rather than a ‘frozen accident’ (21). The organization of the degeneracy in the code is also notably non-random in several ways. First, it is obviously nearly completely relegated to third-base positions, a feature historically attributed to wobble (22). While undoubtedly wobble base pairs are a basic feature of the third position mRNA–tRNA interactions on the ribosome, it is really unknown whether they are ultimately a cause or an effect of the evolution of degeneracy in the code. Second, the code is highly accommodating to variation in GC content at these third positions (GC3), especially whereas all possible 2-fold degenerate codons facilitate the synonyms A/G or T/C but never any other combination, thereby effectively allowing 2-fold degenerate codons the same degree of variation in GC3 as 4-fold degenerate codons. This peculiar organization perhaps might suggest some unknown function for regulating local GC content at synonymous sites independent of the amino acid assignments at the protein level. Previously suggested biological functions for manipulating GC include selection on mRNA stability (23), (24) and/or thermostability and structural B–Z transitions of the DNA itself (25). More recently, standard codon assignment and usage has been linked to patterns of mutational impacts on GC-related intrinsic DNA polymer flexibility in yeast genomes (26) also implicating the potential importance of DNA biophysics in driving some of the universal patterns regarding codons that have long been observed in comparative genomics. A comprehensive study of 1300+ prokaryote genomes recently discovered that, independent of the t-RNA pool, 4-fold degenerate codons are universally biased toward U at the third position while 2- and 3-fold degenerate codons are alternatively biased toward C (27). While these authors invoke a complicated combination of selective forces to possibly explain this trend, we have further noticed that with the exception of only two synonymous codon sets, this apparent trend could be explained parsimoniously by a general preference for a more stable phosphate linkage between the second- and third-base position in the codon (Figure 1)(27,28,29), again suggesting a possible selective constraint, acting outside of protein and perhaps acting on DNA flexibility via synonymous sites.

GC content is very intimately related to intrinsic DNA polymer flexibility (30), minor groove width and helical shape (31,32). Structurally, the width of the minor groove is defined at every third nucleobase (33), primarily by its propensity to be occupied by G or C at those sites. High GC content in a given sequence results in a generally wider minor groove (31,33), resulting in more separation between negatively charged phosphate groups, thereby increasing the intrinsic flexibility of the DNA polymer. Most G or C containing dinucleotides have sugar-phosphate linkages that are more bistable, spending more time switching between BI and BII conformational states (30). Conversely, A- or T-rich sequences are less bistable at sugar-phosphate linkages (i.e. more stable and less flexible). Additionally, the methylation of cytosine markedly reduces this bistability of the sugar-phosphate linkage (34) affecting evolution of GC3 (35) and perhaps creating an even greater level of sequence-independent conformational control of whole-genome folding in organisms capable of epigenetically modifying their DNA. Variation in GC content is very useful in identifying many of the functional regions of genomes due to its role in defining DNA's shape and subsequent molecular dynamics. GC content discriminates regions of coding versus non-coding sequence (36), regions of co-expressed genes defined by homogeneous base compositional domains (37), regions occupied by nucleosome (38) and even functional binding elements as defined by their structural contexts (39,40). It is clear that GC-related DNA polymer physics may play a critical role in the accessibility and regulation of genes within the dynamic chromatin landscape of eukaryotes, especially as many transcription factors and chromatin architectural proteins interact with DNA by charge neutralization at narrowed minor grooves (32). But even in the absence of complex chromosomal architecture in prokaryotes, the propensity of DNA to self-interact through supercoiling is also regulated by GC content. Recently, it has been demonstrated that GC-AT boundaries act to define supercoiling domains that precisely control the initiation and speed of transcription, providing a simple physical explanation as to why co-expressed genes tend to cluster in prokaryote genomes (41–44).

Within their genomic context, codons obviously do not exist in isolation but are connected via dynamic covalent linkages between sugars and phosphates: therefore synonymous codon biases may have significant effects on the molecular dynamics of nucleic acid polymers. It is currently unknown to what degree, codon usage in various genes and genomes is actually influenced by its biophysical context within DNA. Given the potential functional importance of three-base periodicity and GC3 in defining the molecular dynamics of DNA, we wondered if synonymous sites, which largely occur at these positions, might be under some selection to maintain functional levels flexibility in the DNA polymer. Here, we have examined whether intrinsic DNA polymer flexibility is fundamentally related to synonymous codon bias across 35 000+ prokaryotic genes representing 24 major phylogenetic clades, including Carsonella, an insect intracellular bacterial endosymbiont that demonstrates the most extreme GC content and codon bias yet discovered (45). Using a novel neutral simulation, whereby natural selection can be statistically defined as unusual mutational impacts on DNA flexibility outside of the distributional range expected by genetic drift, we explored whether changes in DNA flexibility we observe at synonymous sites are significantly targeted by selection at the gene level. We also compared the extent of evolution acting on DNA flexibility to the level of functional conservation at the protein level (i.e. dN/dS) and explored the question as to whether constraints generated at non-synonymous sites might impose limitations on the ability of organisms to mitigate mutational impacts on DNA flexibility; a prediction that would come about naturally if multiplexed information regarding protein synthesis and backbone dynamics were competing for dual transmission within the same molecular context of DNA. As proof of concept, we also investigated the DNA flexibility allowable via synonymous codon bias in a single-gene context (L,L-diaminopimelate aminotransferase or dapL) across a vastly divergent taxa. dapL is a key enzyme involved in the synthesis of intermediates that are necessary for peptidoglycan and lysine synthesis (46). This gene is well represented in the three domains of life, yet is not ubiquitous, as there are several known alternative pathways leading toward peptidoglycan and lysine. Thus, dapL orthologs have undergone substantial sequence evolution, making them ideal for investigation of deep divergences in synonymous codon bias that may relate directly to cellular phenotypes subject to natural selection.

## METHODS AND MATERIALS

### Sequences, whole-genome alignments, phylogenetic and ancestral sequence reconstructions and subsequent analysis

The prokaryotic genome alignments of orthologous genes were obtained from the Alignable Tight Genomic Clusters (ATCG) Database (47), a database of closely related microbial genomes optimized for microevolutionary research. Prokaryotic multiple alignments for only groups with four or more orthologs on the ATGC database were chosen for our study. Phylogenetic reconstructions were conducted using maximum likelihood trees on the MEGA 5.1 computational core using default parameter settings (48). Ancestral sequence reconstructions were conducted using the baseml subroutine in the PAML software package (36). Substitutions were called by comparing each extant sequence in the alignment to the closest reconstructed ancestral node on the likelihood tree. Both programs were handled as part of a larger Java scripted pipeline that processed each ATGC taxonomic grouping. All subsequent statistical analyses, computer simulations and output graphics were produced using Perl-R scripting. An efficient script for randomly repositioning mutations used subsequently in the analyses below is available in Supplementary File A.

### Selection of L,L-diaminopimelate aminotransferase (dapL) sequences and alignments for this study

dapL sequences in Table 1 were identified by performing a blastN analysis against coding sequence using dapL genes that have been experimentally confirmed as the query (46,49). The enzyme L,L-diaminopimelate aminotransferase (DapL) defines a novel variant of the lysine anabolic pathways. In certain organisms from bacteria, archea and eukaryotic organisms (photosynthetic cohorts), the enzyme catalyzes the conversion of tetrahydrodipicolinate to L,L-diaminopimelate in one step circumventing the three enzymes in the more popular acyl pathways that utilizes acetylated or succinylated intermediates facilitated by the enzymes tetrahydrodipicolinateacylase (DapD), *N*-acyl-2-amino-6-ketopimelate aminotransferase (DapC) and *N*-acyl-L,L-2,6-diaminopimelatedeacylase (DapE) (46). dapL sequences are suitable for this study because the gene is integral due to its involvement in the production of lysine, a ubiquitous primary metabolite involved in protein synthesis. In addition, in bacteria, the enzyme is also involved in the synthesis of peptidoglycan (PG) since the penultimate compound; *meso*-diaminopimelate is involved in PG cross-linking in most Gram-negative bacteria and lysine serves the same purpose in the PG of Gram-positive bacteria (50,51,52).

**Table 1.** dapL orthologs from the three domains of life along with the locus tags for each ortholog used in the study.

Organism	Locus tag
Archaea	
*Archaeoglobus fulgidus* DSM 4304	AF_0409
*Candidatus methanoregulaboonei* 6A8	Mboo_2096
*Geoarchaeota archaeon* OSPB-1	Cren1_8best_scaffolds_00013940
*Methanobacterium formicicum* DSM 3637	A994_03433
*Methanocaldococcus villosus* KIN24-T80	MetviDRAFT_0533
*Methanocella paludicola* SANAE	MCPlv_0872
*Methanomethylovorans hollandica* DSM 15978	Metho_1006
*Methanospirillum hungatei* JF-1	Mhun_2943
*Methanobacterium thermoautotrophicum* str. Delta H*	Mth_52
*Ferroglobus placidus* DSM 10642	Ferp_0809
Bacteria	
*Bacteroides fragilis*	BF2643
*Chlamydia trachomatis* D(s)2923	CtraD_010100002050
*Clostridium thermocellum* DSM 2360	ClothDRAFT_2722
*Ethanoligenens harbinense* YUAN-3	Ethha_0920
*Faecalibacterium prausnitzii* M21	FAEPRAM212_00561
*Geopsychrobacter electrodiphilus* DSM 16401	D888DRAFT_2709
*Prevotella histicola* F0411	HMPREF9138_01762
*Ruminococcus* sp	RSAG_01339
*Veillonella* sp	HMPREF0874_01484
*Verrucomicrobium spinosum* DSM 4136*	VspiD_010100012510
Eukaryota	
Arabidopsis thaliana*	At4g33680
*Chlamydomonas reinhardtii*	CHLREDRAFT_129557
*Ostreococcus lucimarinus*CCE9901	OSTLU_30412
*Ostreococcus tauri*OTH95	estExt_fgenesh1_pm.C_Chr_03.00010014.1
*Perkinsus marinus*ATCC 50983	Pmar_PMAR008102
*Phaeodactylum tricornutum*CCAP 1055/1	PHATRDRAFT_22909
*Physcomitrella patens* subsp. patens	PHYPADRAFT_121808
*Selaginella moellendorfii*	SELMODRAFT_444047
*Thalassiosira pseudonana* CCMP1335	THAPSDRAFT_31394
*Aureococcus anophagefferens*CCMP1984	-

The sequences were retrieved from the IMG (Integrated Microbial Genomes) (http://img.jgi.doe.gov/) database queried on August 27, 2013 using a characterized dapL as query to each domain (annotated by an asterisks). Annotation information pertaining to a locus tag or accession number was not available for the bacterium *Aureococcus anophagefferens* due to the fact the genome was recently sequenced and in the early stages of annotation. The dapL ortholog represented by the bacterium. The bold sequences represent orthologs that have been experimentally confirmed to be authentic (46,49,51,52).

### Measuring intrinsic DNA flexibility and codon bias

The intrinsic flexibility of DNA sequences was calculated using the TRX scoring method of Heddi *et al*. (30). Every possible combination of dinucleotides connected by a single sugar-phosphate linkage is represented by a TRX (twist, roll and X displacement) score that reflects the percentage of time that that particular linkage type resides in the less stable BII conformation in solution (measured in a large NMR meta-analysis). Higher TRX score indicates more BI ↔ BII conformational changes for a given dinucleotide and hence a higher degree of intrinsic flexibility. TRX scores for entire sequences are simply sums of all dinucleotide scores divided by the total number of linkages (i.e. length of the sequence - 1). thus

(1) \begin{equation*} TRX_{seq} = {{{\sum\nolimits_{i = 1}^L {MN_{seq} } }} \!{\left/ \right.}\!{{L - 1}}} \end{equation*}

where L = length of a given sequence and MN are the respective dinucleotides along this length.

We used an information entropy-based approach to calculate codon bias proposed by Suzuki *et al*. (53). This method gives a weighted sum of relative entropy regarding codon usage in a given sequence (Ew) scaled from 0 to 1. We considered the bias to equal (1 - Ew), thus high entropy in codon usage equated to low bias and *vice versa*. This method is nearly identical to the CodonO metric (synonymous codon usage order) proposed by Wan *et al*. (54) and differ only in their methods of computing relative entropy according to Roth *et al*. (1). The codon bias calculation of Suzuki *et al*. (53) was implemented in a Perl subroutine.

### Detecting natural selection acting on intrinsic DNA flexibility

We used a simple extension of our previous methods for computer simulation of neutral evolution (55,56) to determine if intrinsic flexibility of DNA at synonymous sites was under significant selection. Synonymous single-base substitutions were called by pairwise observation of extant sequences with the closest ancestral sequence reconstruction at the nearest node on the maximum likelihood tree. Non-synonymous substitutions were removed from the alignments by converting sites of non-synonymous substitution in the ancestral sequence with the same nucleobase observed in the extant sequence. The observed mutational impact on intrinsic flexibility of synonymous sites was obtained by comparing the TRX scores of extant and modified ancestral sequences.

(2) \begin{equation*} dTRX_{syn}^{obs} = \left| {TRX_{syn}^{extant} - TRX_{syn}^{ancestral} } \right| \end{equation*}

To obtain the expectation of the mutational impact on flexibility (TRX) for the same set of mutations set randomly with respect to the codon organization, the synonymous substitution events were randomly repositioned to sites occupied by identical nucleobases, thus base composition was held constant while dinucleotide composition was allowed to change. Note: the TRX scores are determined by sums of dinucleotide-based values for each P linkage as in Equation (1).

(3) \begin{equation*} dTRX_{syn}^{neutral} = \left| {TRX_{syn}^{extant} - TRX_{syn}^{random} } \right| \end{equation*}

Mutations were randomly repositioned within local 200 bp sliding windows moved along extant species sequences. We provide a Perl script for the purpose of generating randomly repositioned substitutions written by K.V. Schulze (Supplementary File A). The local mutational impacts under neutral recalculated 1000 times to generate a frequency distribution of neutral expectations under a model of genetic drift that maintains the same local evolutionary distances observed in the original phylogeny. If the observed mutational impacts on local DNA flexibility $(dTRX_{syn}^{obs} )$ were significantly smaller or greater than the simulated neutral impact (i.e. in the bottom or top 2.5% of distribution tails of $dTRX_{syn}^{neutral}$), then significant purifying or positive selection could be inferred. The strength of selection was defined as the ratio of the observed mutational to the mean neutral mutational impact on DNA flexibility.

(4) \begin{equation*} \omega _{GC3} = \frac{{dTRX_{syn}^{obs} }}{{\sum\nolimits_{i = 1}^{1000} {dTRX_{syn}^{neutral} /1000} }} \end{equation*}

Overall genomic frequencies of each type of selection were tabulated over all sites to generate a single genomic value. These values were collected in order to investigate comparative trends of natural selection on intrinsic DNA flexibility in prokaryotic DNA. If genomic differences in base composition have affected the evolution of intrinsic DNA flexibility, the genome-wide average frequencies of regions under both purifying and positive selection across were correlated to genome-wide average TRX score across the 24 prokaryotic genomes analyzed. Regions of selection under the repositioned mutation test were called using a *P* value of 0.025 on each tail simulated distribution of local neutral mutational impacts (i.e. overall alpha level = 0.05).

### Codon bias-driven variation in DNA flexibility for a given protein space

To test whether the synonymous codon bias of a given gene contributes to an unexpected degree of intrinsic DNA polymer flexibility/inflexibility within that gene's protein space, we calculated the local minimum and maximum TRX score allowable by synonymous codon choices throughout the coding sequence. The local TRX scores for each codon were calculated over the entire sequence. The sum TRX score of a given codon (TRX_cdn_) was calculated using both internal P linkages and both external P linkages (see Figure 1 in Babbitt and Schulze (26)). To generate the sequences with the minimum TRX score for a given protein space, synonymous codons with the lowest TRX_cdn_ score were chosen and the local TRX_cdn_ scores. The average synonymous TRX_cdn_ scores and subsequent 95% CIs were calculated using 500 synonymous randomizations of the coding sequence. All values of local TRX_cdn_ shown in figures were smoothed over a 73 bp interval. The local deviation of a given TRX_cdn_ from its synonymous average was normalized to its range within local sequence space, thus calculated as

(5) \begin{equation*} {\rm devTRX}_{{\rm cdn}} = \frac{{\left| {{\rm TRX}_{{\rm cdn}}^{{\rm real}} - {\rm TRX}_{{\rm cdn}}^{{\rm syn}} } \right|}}{{\left| {{\rm TRX}_{{\rm cdn}}^{\min } - {\rm TRX}_{{\rm cdn}}^{\max } } \right|}} \end{equation*}

where $TRX_{cdn} = \sum\nolimits_{i = 1}^4 {TRX_{mn} }$ for the 4 P linkages defined by each dinucleotide (MN).

To investigate gene-level trends, these local deviations of TRX within protein space were averaged over coding regions to obtain a mean deviation for a given gene. Across all genes in each genome, the average deviations (devTRX_cdn_) were correlated to gene-level codon biases and gene-level deviations from 50% GC content. To further investigate the functionality of different levels of intrinsic DNA flexibility, the average deviations were also correlated with codon bias across the 27 dapL genes. Species and accession numbers are listed in Table 1. Data and graphics files for all dapL genes are presented in Supplementary File B. Data and graphics files for all Carsonella genes (45) are presented in Supplementary File C.

### Genome-wide relation of prokaryotic protein conservation with the mutational impact on intrinsic DNA flexibility

Base substitution events were inferred from the comparison of extant sequences to their closest ancestral sequence on the reconstructed phylogeny. Comparisons with greater than 20% base mismatch were discarded from analysis. The base substitutions were generally classified according to their effect on the purine-pyrimidine ground-state of the sequence (i.e. transition (Ts) or transversion (Tv)) and their effect on amino acid composition (i.e. non-synonymous (non-syn) or synonymous (syn)). The average mutational impacts of each class of mutation on a given coding sequence were calculated as follows

(6) \begin{eqnarray*} &&\Delta TRX = dTRX \nonumber \\ &&= {{{\sum\nolimits_{i = 1}^n {\left( {TRX_{cdn}^{ancestral} - TRX_{cdn}^{extant} } \right)} }}/\,\!{n}} \end{eqnarray*}

where *n* = number of codons in a given coding sequence.

The respective dTRX values for each of the four functional combinations of base substitutions (i.e. synTs, synTv, non-synTs and non-synTv) were then calculated for all sequences in all genomes. The rates of substitution on synonymous sites (dS) and non-synonymous sites (dN) were used to calculate omega ratio (dN/dS), a proxy for the level of selection occurring on the protein level for a given gene. The association of dTRX and dN/dS were then examined for evidence of increased dTRX with reduced dN/dS, indicating a potential trade-off between selection at the protein level and mutational events impacting intrinsic flexibility of DNA. Data and graphics files for all genomes are presented in Supplementary File D and summarized in Table 2.

**Table 2.** F tests comparing the variance in mutational impact on intrinsic DNA flexibility (dTRX) for functionally conserved versus adaptively evolving genes (observed in Figure 7).

Species/group	*F* value syn	*P* value syn	*F* value non-syn	*P* value non-syn	*n* conserved	*n* adaptively evolving
*Methanococcus maripaludis*	5.13585	4.44 × 10^−15^	0.3902547	5.63 × 10^−11^	1169	77
Bacillus sp	6.863267	0	0.2741067	5.81 × 10^−26^	1565	94
Brucella-Ochrobactrum	8.410006	0	0.4192114	1.25 × 10^−09^	1534	77
Campylobacter sp	6.3016	5.30 × 10^−08^	0.3611305	2.31 × 10^−06^	869	31
Listeria sp	13.05	1.62 × 10^−14^	0.1765246	1.80 × 10^−22^	884	37
Mycobacterium sp	13.05	1.62 × 10^−14^	0.1765246	1.80 × 10^−22^	884	37
Nitrobacter sp	6.813388	1.38 × 10^−09^	0.5375341	0.003787364	709	36
*Prochlorococcus marinus*	3.568853	0	0.5972523	9.04 × 10^−06^	1045	147
*Pseudomonas aeruginosa*	3.263118	1.27 × 10^−07^	0.3782765	4.61 × 10^−10^	3142	59
Pseudomonas sp	8.341558	0	0.2492518	9.13 × 10^−23^	2495	63
*Pseudomonas syringae*	6.507828	0.000163719	0.3905284	0.002296555	409	15
*Rhodobacter sphaeroides*	6.981686	5.92 × 10^−05^	0.3803578	0.001223606	408	16
Rickettsia sp	14.15104	1.98 × 10^−06^	2.42576	0.05746677	607	14
Shewanella sp	4.076466	1.45 × 10^−13^	0.2152939	1.09 × 10^−36^	1770	87
Vibrio sp	5.911175	2.21 × 10^−06^	0.3008131	2.23 × 10^−07^	764	25
Yersinia sp	22.60118	0	0.1968196	7.17 × 10^−23^	1426	43

Tests were conducted independently for effect of synonymous and non-synonymous sites on dTRX across all aligned gene sets.

### Overall trends regarding average genome-wide codon bias, GC content and intrinsic DNA flexibility

For each of the 24 prokaryotic genomes analyzed above, single genome-wide averages for entropy-based codon bias, GC content and TRX score were analyzed for associative trends across species. A linear regression of genome-wide GC contents against the genome-wide TRX averages was produced. Regressions were also conducted for genome-wide codon biases against the genome-wide TRX averages. Because entropy-based codon biases do not discern the direction of bias with respect to GC3 and because codon bias tends to increase with both high and low GC content, two separate linear regressions were produced for species falling above or below the weighted average of the 10 dinucleotides comprising the TRX scale (i.e. TRX = 20.625). Thus, the trending of codon bias in high GC and low GC genomes was analyzed separately.

## RESULTS

### Overall trends regarding average genome-wide codon bias, GC content and intrinsic DNA flexibility

**Figure 2.**
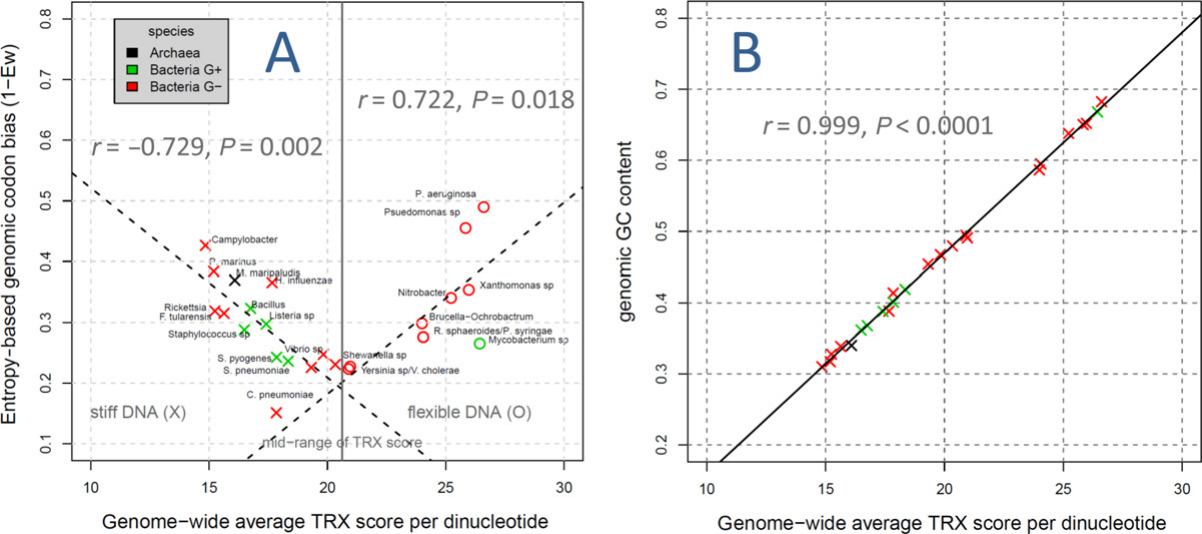
A fundamental relationship between intrinsic DNA flexibility (TRX score), genomic GC content and entropy-based codon bias. (**A**) Prokaryotic genomes with uncharacteristically stiff or flexible genome architecture, and thus deviating from the middle of the TRX scale, demonstrate increased codon bias. (**B**) The relationship between GC content and intrinsic DNA flexibility at the genomic level is particularly pronounced, reflecting the trends easily observed in the TRX scale, where flexibility increases with GC containing dinucleotides.

Across all prokaryotic species clades in our analysis, we report strong and significant overall linear trends associating the levels of codon bias and the intrinsic DNA flexibility averaged over whole genomes. These trends are inflected around the midpoint of the flexibility scale (i.e. TRX scale on the X axis) (Figure 2A). Codon bias increased with both a decline or gain in intrinsic DNA flexibility from its midpoint value (*r* = −0.729, *P* = 0.002; *r* = 0.722, *P* = 0.018, respectively), demonstrating a multi-genomic trend indicating that genome-wide codon biases are generally associated with unusual shifts away from the value of intrinsic DNA flexibility expected *in vitro* with equal base composition. The genome-wide intrinsic DNA flexibility was confirmed to be highly dependent on genome-wide GC content (*r* = 0.999, *P* < 0.001; Figure 2B), a further indication of the very strong biophysical effects of GC on backbone dynamics of DNA.

### Comparative levels of natural selection on intrinsic DNA flexibility in prokaryotic and eukaryotic DNA

**Figure 3.**
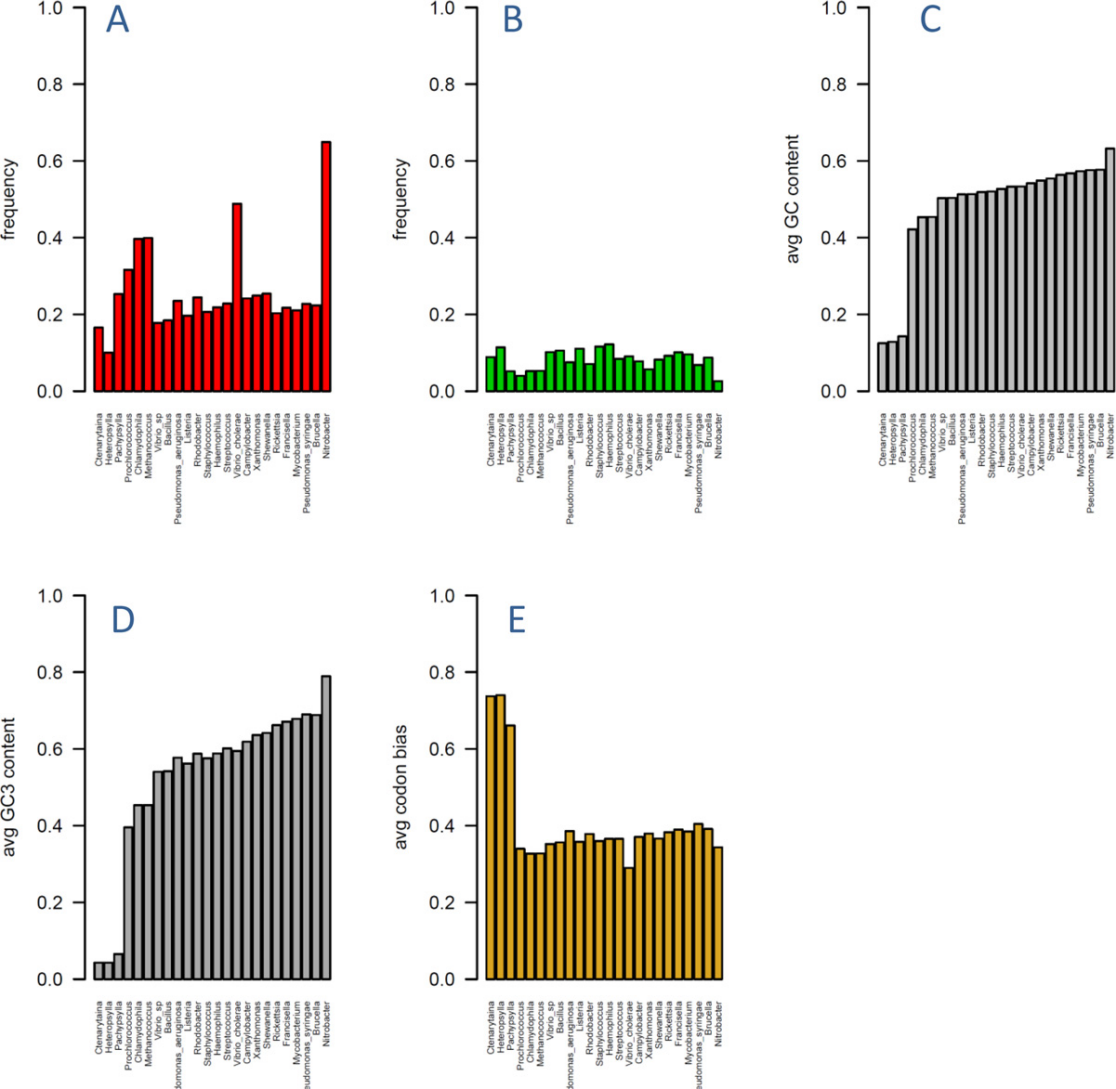
Respective frequencies of regions of the genomes under natural selection acting on intrinsic DNA flexibility (i.e. TRX score) at synonymous sites. (**A**) purifying selection or functional conservation of intrinsic DNA flexibility at synonymous sites and (**B**) positive selection or adaptive alteration of intrinsic DNA flexibility at synonymous sites. Average genomic GC, GC3 and entropy-based codon bias are also shown (C, D and E, respectively). Genes under natural selection were identified using neutral simulations of mutational impacts on TRX score. Species in all plots are ordered according to increasing genomic GC content (i.e. plot C).

The overall frequencies of natural selection acting on intrinsic DNA flexibility at synonymous sites were estimated by globally averaging the local comparisons of true ancestral → extant dTRX to simulated distributions of local neutral impacts of randomly repositioned silent (i.e. synonymous) mutations on dTRX (Repositioned Mutation or RM test; (55,56). This RM test indicated a broad range of purifying selection/functional conservation of DNA flexibility with roughly 20–40% of all genes of a given genome (Figure 3A) showing statistical significance according to this test. As expected, genes identified as subject to adaptive evolution affecting DNA flexibility were less frequently occurring than conserved genes, averaging less than 5% of all genes for most genomes (Figure 3B). Genome-wide GC, GC3 and codon bias are shown in Figure 3C–E respectively. GC content in bacterial endosymbiont genomes (Carsonella inhabiting Ctenarytaina, Heteropsylla and Pachypsylla) is thought to decay largely due to the effects of drift (i.e. Muller's rachet), so it was surprising that the overall percentage of genes where flexibility was significantly conserved was only moderately reduced (roughly 30%) compared to the other genomes (Figure 3A) possibly indicating even a weak or moderate disruption in mutation-selection balance acting on synonymous codons can result in extreme GC content when given enough time. The percentage of Carsonella genes showing significant signatures of adaptive evolution on flexibility at synonymous sites matched those of the other prokaryotes in our study (Figure 3B) thus suggesting that adaptive events may also play an unknown role in the evolution of extremely reduced GC. Boxplots showing the genome-wide distributions of the strength of selection according to our test indicate that 12 of 18 prokaryote genomes are under moderate selection to conserve intrinsic DNA flexibility at synonymous sites (Supplementary Figure S1–ATGC database and Supplementary Figure S2–Carsonella). One genome (Brucella) shows a genome-wide signature of adaptive evolution, the remaining 5 exhibit very weak selection or drift. Shewanella has a separate cluster of genes with very strongly conserved flexibility.

### Codon bias-driven variation in DNA flexibility for a given protein space

**Figure 4.**
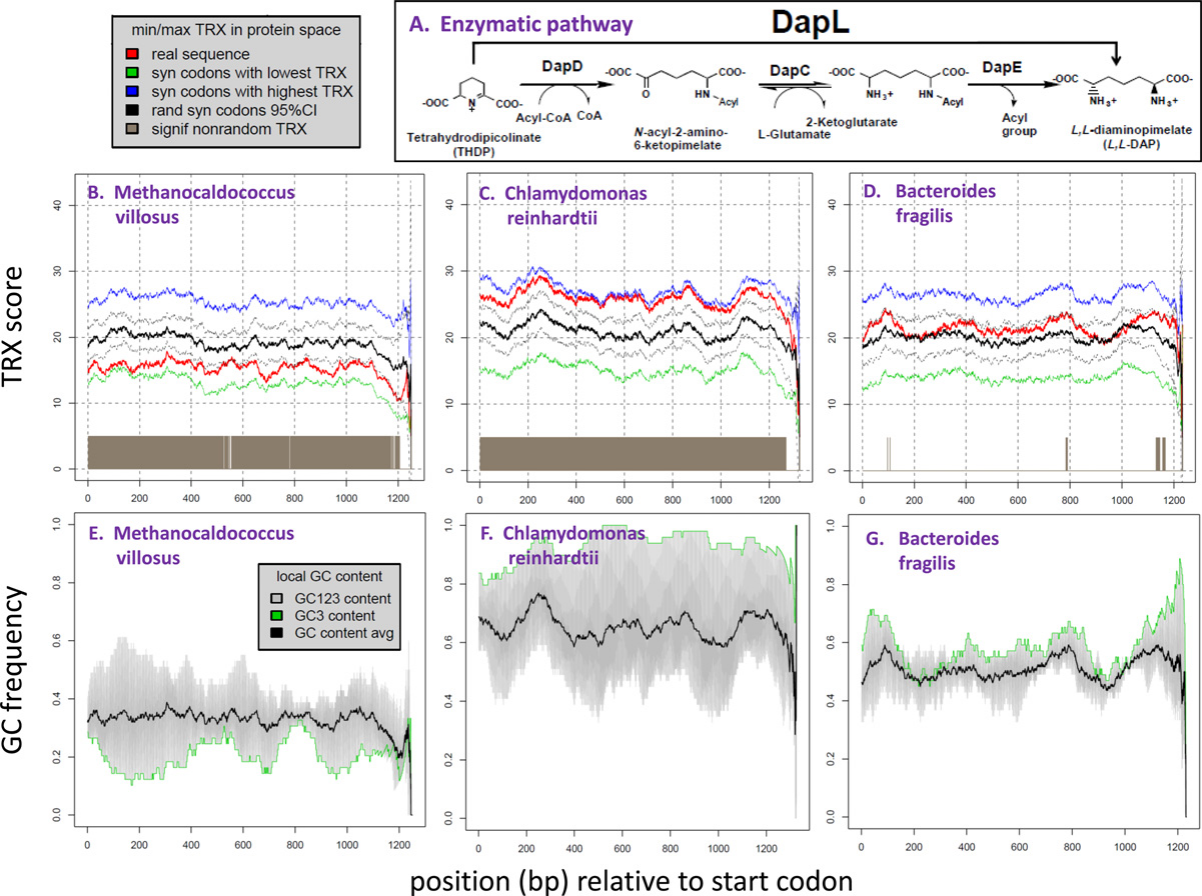
The intrinsic DNA flexibility and GC3 of the diaminopimelate aminotransferase (DapL) gene from three representative species. (**A**) Reaction catalyzed by DapL showing the ability of the enzyme to circumvent the DapD, DapC and DapE steps in the *Escherichia coli* acyl pathways. (DapD), *N*-acyl-2-amino-6-ketopimelate aminotransferase (DapC), *N*-acyl-L,L-2,6-ketopimelate deacylase (DapE), L,L-diaminopimelate aminotransferase (DapL) (46). Example plots of smoothed flexibility (TRX) scores (B–D) are shown for (B) an Archean, *Methanonocaldococcus villosus*,(C) an algae, *Chlamydomonas reinhardtii* and (D) a bacteria, *Bacteroides fragilis*. Plots B and C show DapL gene TRX scores (red) that are extreme examples of inflexible and flexible genes (respectively) for their given protein space (bounded in blue and green). The black lines indicate the average synonymous TRX score and 95% CI when synonymous codons are chosen randomly (local gene regions where observed TRX is outside this CI are flagged in brown on the X axis). A dapL gene's deviation from average synonymous flexibility is achieved largely by codon usage bias. Figures E–G show respective smoothed trends in overall GC content (black), GC content in each reading frame (gray) and GC3 (green).

**Figure 5.**
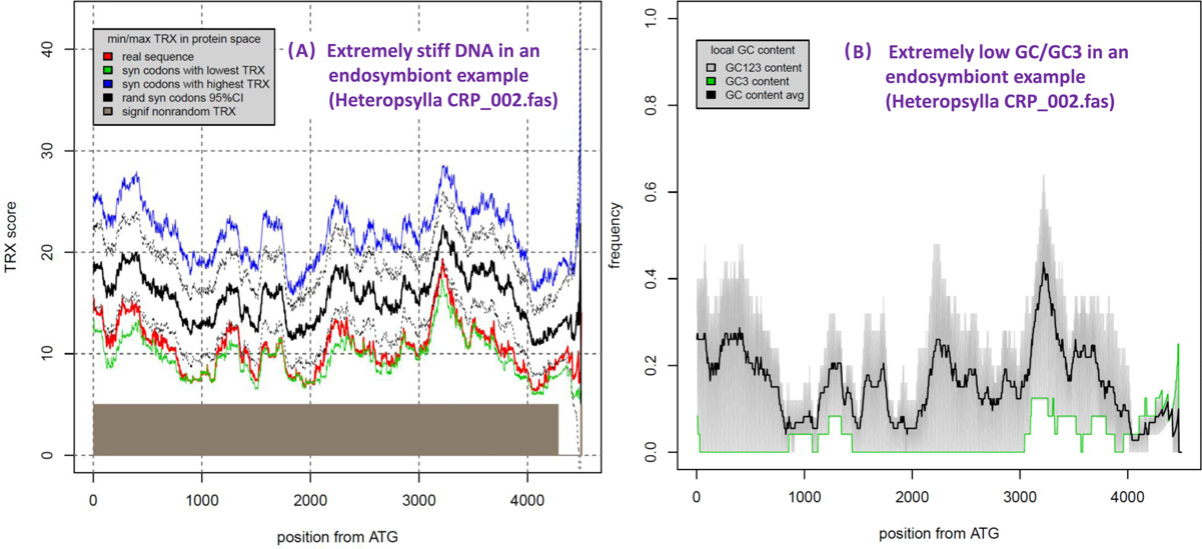
The intrinsic DNA flexibility and GC3 for a typical gene from the extreme psyllid endosymbiont, Carsonella sp in Heteropsylla. Example plots of (**A**) observed (red), minimum (green), maximum (blue) and average synonymous (black) intrinsic flexibility TRX scores and (**B**) respective trends in overall GC content (black), GC content in each reading frame (gray) and GC3 (green). Supplementary File D has plots for all known Carsonella genes.

**Figure 6.**
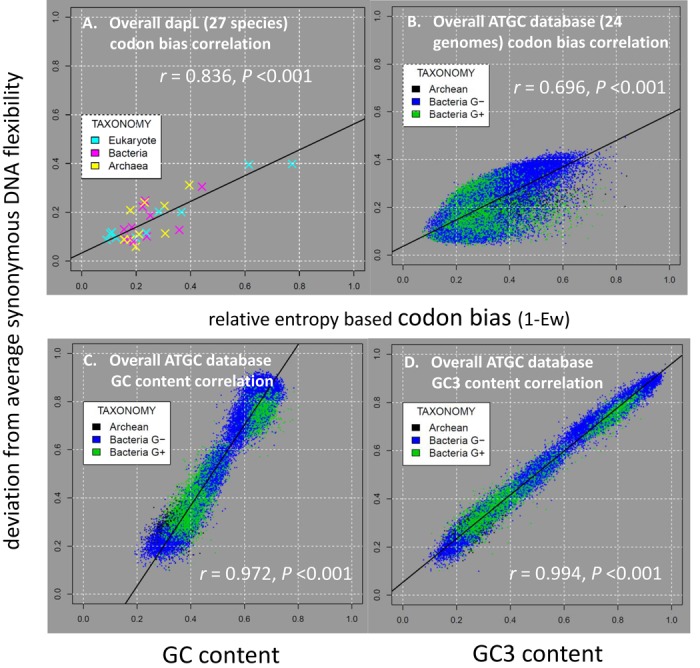
The positive association (r) of entropy-based codon bias (1-Ew) and gene-level deviations from synonymous flexibility for the given protein space for (**A**) the 27 dapL genes and (**B**) all 35 234 prokaryotic genes in the ATGC database (24 genomes). Examples of the deviation from average synonymous flexibility given its protein space for single genes are shown in Figure 5B–D (i.e. the mean devTRX_cdn_ = abs[mean red line-mean black line]/[mean blue line - mean green line]). Also shown are the positive association of (C)overall gene GC content and (D) overall gene third position GC content (GC3) with gene-level deviations from synonymous flexibility for the given protein space.

Within a single highly functional gene involved in lysine and cell wall synthesis (L,L-diaminopimelate aminotransferase or dapL, Table 1; Figure 4A), we found a surprising amount of variation in intrinsic DNA flexibility driven locally by GC3 maintained by local codon bias. The hyperthermophilic bacterium, *Methanocaldococcus*, showed an uncharacteristically low level of DNA flexibility and GC3 in dapL given its corresponding synonymous protein space (Figure 4B, E). Alternatively, the algal dapL gene (*Chlamydomonas reinhardtii*) demonstrated an even more uncharacteristically high level of DNA flexibility and GC3 across its entire coding region (Figure 4C, F). Other organisms (e.g. *Bacteroides fragilis* and many others in Supplementary File B) exhibit less extreme overall values of GC3 and flexibility values (e.g. Figure 4D, G), however GC3-driven local shifts in TRX score are clearly apparent throughout. The trends observed in dapL are even more markedly extreme in the Carsonella dataset (Figure 5; Supplementary File C), showing that the extremely low average overall GC content (14%) is accompanied by an even lower GC3 (<5%) with all genes showing long regions of total preference for A or T ending codons. Across all 27 examples of the dapL genes, representing all major domains of the tree of life, the role of synonymous codon bias in altering the flexibility of the coding region was strong and highly significant (*r* = 0.836, *P* < 0.001; Figure 6A). This trend is mirrored at the level of whole genomes. Across all 35 000+ prokaryotic genes we analyzed, we found similar significant correlations between gene average codon bias, GC/GC3 content and the gene average deviation of flexibility within synonymous protein space (codon bias: *r* = 0.696, *P* < 0.001; Figure 6B) (GC content: *r* = 0.972, *P* < 0.001; Figure 6C) (GC3 content: *r* = 0.994, *P* < 0.001; Figure 6D), indicating that GC3-related synonymous codon bias accounted for nearly 50% of the variation in intrinsic DNA flexibility across all genomes. The shifts in DNA flexibility are even more isometrically correlated with GC3 than with overall GC content (Figure 6C and D) further indicating the general importance of synonymous sites in governing the flexibility of coding regions. GC3-related synonymous codon bias and control over intrinsic DNA flexibility appear very likely functionally associated across a vast biological scale, affecting both local regions on single genes as well as broadly observed taxonomic relationships based on whole genome averages.

### Genome-wide patterns of prokaryotic protein conservation and mutational impact on intrinsic DNA flexibility

**Figure 7.**
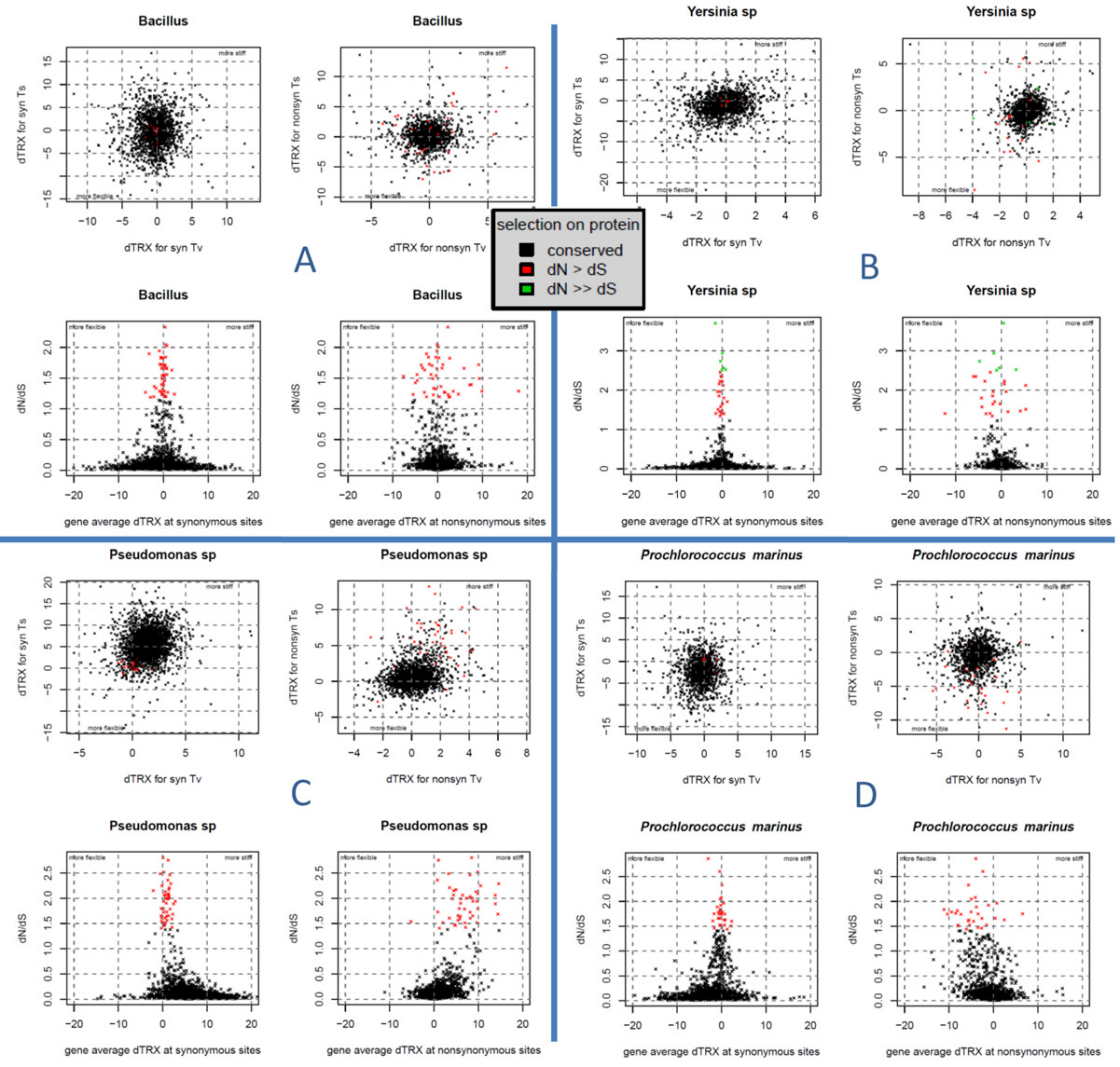
Fundamental constraints between protein evolution (dN/dS) and mutational impacts on intrinsic DNA flexibility (dTRX) and hence, genome architectural dynamics. Example plot sets are shown for two genomes with low codon bias (A) *Bacillus* and (B) *Yersinia*, and two genomes with extreme codon bias; (C) *Pseudomonas* = high GC and (D) *Prochlorococcus* = low GC. Within each plot set, genes functionally conserved at the protein level (i.e. low dN/dS) are shown in black, while genes adaptively altered at protein level are shown colored. Mutational impacts on flexibility (dTRX) are shown separately for synonymous sites (left side of plot set) and non-synonymous sites (right side of plot set). dTRX for transitions and transversions are separated in the upper plots of each set.

To investigate the potential trade-offs of evolutionary pressures acting at the level of nucleic acid polymer and protein, we plotted each average gene-level mutational impact on intrinsic DNA flexibility (i.e. dTRX) against the gene's corresponding value of dN/dS (Figure 7). While we observed no clear patterns for mutations at non-synonymous sites, we did observe that mutations at synonymous sites demonstrated a clear pattern of tremendously increased variance in dTRX when presumed functional conservation at the protein level was high (i.e. low dN/dS). This pattern was universal in all 24 prokaryotic genomes we examined. The value of dTRX at synonymous sites converged very close to zero when dN/dS> 1 indicating that these genes were likely either adaptively evolving or otherwise released from the functional organization of coding regions (e.g. pseudo-gene). This trend of convergence appears species-specific (e.g. lower in *Bacillus*, Figure 7A, than *Yersinia* Figure 7B) and often skewed according to the prevailing GC content of a given genome, indicating the presence of a unique biological signal as opposed to purely statistical sampling artifact (Figure 7C and D showing high GC *Pseudomonas* and low GC *Prochlorococcus* genomes, respectively). All other graphs for all species analyzed are given in Supplementary File D. For each genome, individual F tests comparing the variance in dTRX for functionally conserved versus adaptively evolving or drifting genes over both synonymous and non-synonymous sites are summarized in Table 2. For those species with sufficient sample sizes for genes where dN/dS > 1, F statistics for synonymous sites show that variance in dTRX is consistently many times greater in functionally conserved genes (dN/dS< 1) than in adaptively evolving or drifting genes (dN/dS> = 1), indicating a functional constraint between protein structure/function and intrinsic flexibility of DNA. This trend is not apparent at non-synonymous sites, where, in fact, a much weaker and opposite trend is observed. These sites have less effect in determining minor groove dimensions and intrinsic DNA flexibility and therefore might be expected to exhibit little or no tradeoffs with protein level constraints involving flexibility.

## DISCUSSION

### The multiplexing of information in DNA

The coding regions of genomes may present a significant informational challenge to the cell because of their need to multiplex the different types of information regarding protein synthesis, genome folding/supercoiling and transcriptional regulation into a singular molecular context of DNA. It has been recently reported that footprints of exonic transcription factors affect codon bias in roughly 15% of the codons in the human genome, supporting that the genetic code itself may have a dual coding feature (57) We believe our results may further illuminate the biophysical mechanism by which these multiplexed dual signals regarding protein structure and the genome's dynamic architecture are maintained by GC-related dynamics of the DNA polymer. This multiplexing problem of the coding region is analogous to that of telecommunication networks which have been engineered in many different ways for the overlap of analog message signals or digital data streams onto a single physical transmission medium. Methods devised for this task include the division of the transmission medium according to space, time, frequency and even the segmentation of the transmission codes themselves on these divisions. Similarly, the degeneracy of the various messages contained in DNA also appears to allow for the segmentation and simultaneous parallel messaging in one medium. Our results may suggest that messaging in DNA includes both analog components (e.g. indirect readout of DNA flexibility/dynamics, local electrostatic charge and charge regulation) in addition to its better known digital components (i.e. genetic code and direct readout of transcription factor binding motifs on the major groove). All of these molecular properties are strongly correlated to both base composition and sequence, making investigation of any single property problematic at best. However, where multiple informational signals are multiplexed into DNA, it should also be more difficult for natural selection to act on independently on any one type of information, thus inducing some fundamental evolutionary tradeoffs. We believe this is evident by the universal tradeoff between the evolution of amino acid sequence (dN/dS) and synonymous site backbone dynamics (dTRX) that we have observed in all genomes examined here. This might also imply that general differences observed in mutation rate between synonymous and non-synonymous sites are driven by the relative strengths of selection pressures acting upon these various message components, and not the existence of pure isosemantism as originally suggested by (3).

Through our investigations of the evolution of intrinsic DNA flexibility at the level of single genes and whole genomes, we have found evidence for a simple, and yet primary, functional basis for the broad taxonomic variation long observed in codon bias. We have demonstrated a broad general association of DNA polymer biophysics, codon bias and protein evolution in the coding sequences of unicellular genomes. We find an apparently universal constraint connecting the evolution of protein structure and the evolution of DNA polymer biophysics as it may affect genome folding or coiling dynamics through altered local charge and flexibility. We also find a universal relationship across taxa indicating that GC3-related codon bias acts to maintain both local gene-specific, as well as species-specific levels, of DNA flexibility. We utilized a novel method of neutral simulation to identify genes where DNA flexibility governed by synonymous sites is under significant natural selection. We further demonstrate that DNA flexibility is subject to significant and often rather taxon-specific levels of purifying selection, implying that DNA structural dynamics are quite often moderately conserved. However, our observation that bacterial endosymbionts with very extremely low levels in genomic GC and GC3 (45) have rather similar, yet moderately relaxed purifying selection on DNA flexibility compared to other prokaryotes, clearly suggests that mutational biases involved in genomic decay can drive changes in GC3 far beyond the limit of selective constraints on DNA flexibility. Taken together, the observations of this study support a conjecture that synonymous codon bias in most genomes represents a mutation-selection balance and may also represent trade-offs between selection acting on the protein level and the selection acting on functional genome architecture and gene regulation in the cell. This along with other proposed functional aspects of synonymous sites (2,58–61) may also invalidate the common practice of using global trends in GC3 for generally inferring that mutational bias is present (6,62–64).

### Hypothesis: the accessorizing of a putative primordial genetic code

While it is now relatively well-established that codon assignments in the standard genetic code are highly optimized against translational error (20), providing one of several alternative theories for the evolutionary origin of the genetic code (65,66), it has recently also been suggested that this current level of error minimization in the triplet-based genetic code represents a significant step down from all possible putative primordial doublet-based genetic codes (67). This might indicate that the expansion to a triplet code has resulted in some significant reduction in its adaptive optimization regarding molecular events surrounding the translation of RNA to protein. Together with the role of GC3 content in determining intrinsic DNA flexibility (30) and the role of 3-base periodicity in defining the minor groove width and DNA helical shape (33), our results might also suggest that the triplet codon may have been a much later adaptation for the functional multiplexing of information (66) pertaining to genomic gene regulation over a simpler and more ancient system for encoding protein information. Thus, the degeneracy of the genetic code may represent the accessorizing of a primordial doublet code to carry additional regulatory information sometime after DNA was adopted as the primary informational storage molecule in the cell. Interestingly, the observation by Novozhilov and Koonin (67) that arginine appears to bring the most non-optimality to the code, is highly congruent with the observations of Rohs *et al*. (32) for arginine's critical role in genomic architecture and gene regulation via charge neutralization of DNA through interactions on the minor groove. Thus, electrostatic charge regulation of the genome during ‘indirect readout’ by arginine-rich transcription factors may also represent a much later evolutionary modification as well. We further hypothesize that if the expansion of the code occurred for the purpose of multiplexing genomic architectural dynamics over a primordial doublet code, then its conspicuous third position degeneracy, as well as its apparent reduction in error minimization when compared to simpler codes (67), can be explained as a suboptimal yet adaptive solution for the accommodation of two types of information into one transmission media (i.e. DNA's dynamical structure). All of this conjecture is congruent with our previous findings that triplet codon assignments have helped maintain DNA flexibility over evolutionary timescales and that codon bias drives correlations between mutational impacts on DNA flexibility and Ts:Tv ratio, dN/dS and dN (26). This, in addition to our findings presented here, suggests that adaptive molecular events related to DNA self-interaction and DNA–protein interaction, and not just events surrounding RNA-driven polypeptide assembly during translation may have been instrumental in the later stages of the evolution of the genetic code.

What might be the advantage of this more highly-refined genomic architecture enabled via local changes to the structural dynamics of DNA polymer? The packaging of genes into higher order structures obviously has two ultimate effects. First, it allows more genes to be incorporated into a more compact space in the cell, and second, it tends to repress individual gene expression. Both of these genomic characteristics can then allow organisms to better program responses in gene expression to local environments. Botzman and Margalit (68) have recently demonstrated that variation in prokaryotic global codon bias and GC3 is highly associated with species tolerances to environmental variability. Based on our findings, we might surmise that this association is driven by the ability of organisms with relatively high GC3 content (and high DNA flexibility) to repress gene expression via torsion-based stalling of RNA polymerase (42) in a large complement of environmentally inducible genes. In fact, in our study of the dapL gene, we also find an organism with unusually robust environmental tolerances, the algae *C. reinhardtii*, has unusually high DNA flexibility for its given protein space. In this same dapL genetic study, we find that several thermophiles are characterized by stiffer DNA, perhaps because its phosphate linkages are more thermodynamically stable (although this could also make DNA more prone to denaturing).

DapL is an enzyme involved in the anabolism of the *meso*-diaminopimelate (*m*-DAP) and lysine. Both *m*-DAP and lysine are cross linking amino acids in the peptidoglycan cell wall of most bacteria and lysine is one of the 20 common proteogenic amino acids. The dapL gene is well-represented in the three domains of life, yet is not ubiquitous as there are three other anabolic routes toward *m*-DAP and lysine (46). Thus, the dapL gene has undergone substantial sequence evolution, making it ideal for investigation of deep divergences in codon bias that may relate to cellular phenotype. As this enzyme synthesizes several critical components for cell wall and amino acid biosynthesis, additional future studies of *E. coli dap* mutants may serve as the basis for a tractable experimental system for future investigations linking GC3-related codon bias to DNA structural dynamics and phenotypic regulatory effects.

### Broader implications

Scientists in many areas of nucleic acid research are beginning to view the sequence-dependent backbone dynamics of nucleic acids as strong determinants for the fine-tuning of gene regulation (23,41–43,69). We have extended these ideas to an inquiry of the molecular evolutionary process by suggesting that evolution should likewise target backbone dynamics of the double helix to achieve its many effects on the packaging and regulation of genes in cell. It is now fast becoming evident that the genome can only be wholly understood as a biophysical molecule (70). This is especially true regarding its most highly functional and information dense regions (i.e. coding and *cis*-regulatory non-coding DNA). There is published evidence to suggest that the genomic packaging and unfolding as it is defined by both DNA backbone dynamics as well as the nucleobase sequence is targeted and shaped by evolution (26,33,40,55–56,71–75). We currently understand very little about how molecular evolution impacts the biophysical nature of genomes as well as their associations with architectural and regulatory proteins. Given the rapid development of next-generation sequencing and its application to the whole genome mapping of epigenetic and regulatory states, there is a critical need for a new theoretical molecular evolutionary framework and subsequent computational tools that can be applied to these new types of data. Over recent decades, molecular evolutionary studies have made considerable contributions to the fields of functional and comparative genomics. However, because these studies were largely enabled by advances in DNA sequencing technologies, researchers have primarily conducted only sequence-based analyses. Yet no hard rule of nature requires chemical evolution to be restricted entirely to digital information encoded only within the nucleobase sequence of DNA. Here we present novel evidence that sequence-based analog properties of DNA–protein and DNA self-interactions (42) are capable of heritably evolving under a regime of weak-to-moderate selection. Recent work suggests this new molecular evolutionary paradigm may extend even to epigenetic chemical modifications of both DNA (76,77) and probably also the many architectural proteins that interact with it. The packaging of DNA into the nucleus of the cell often requires that structural-based dynamics must often overlap (i.e. multiplex) with sequence-based genetic information contained in the genome. Over evolutionary timescales, this additional information is subject to both sequence-dependent ‘mutational’ impacts, as investigated in our work here, and also probably many sequence-independent ‘epimutational’ impacts as well. Both classes of these chemical changes potentially disrupt how DNA–protein and DNA self-interaction involved in gene regulation and local genome architecture are defined by local polymer biophysics. We have tried to show here that DNA polymer is not simply an inert carrier of genetic information, but rather is a dynamic partner of many other molecules in the cell, and thus capable of predetermining regulatory access to genes through its own physical chemistry. And this very quality of DNA may have sculpted gene and genome evolution over very deep geological timescales to some as yet unknown degree.

## SUPPLEMENTARY DATA

Supplementary Data are available at NAR Online.

SUPPLEMENTARY DATA
