# Supplementary material for: Synonymous codon bias and functional constraint on GC3-related DNA backbone dynamics in the prokaryotic nucleoid
Source: Nucleic Acids Res. 2014 Sep 8;42(17):10915–26. doi: 10.1093/nar/gku811 (PMC4176184; doi:10.1093/nar/gku811)
Supplement: SUPPLEMENTARY DATA [file supp_42_17_10915__index.html]

Synonymous codon bias and functional constraint on GC3-related DNA backbone dynamics in the prokaryotic nucleoid — Synonymous codon bias and functional constraint on GC3-related DNA backbone dynamics in the prokaryotic nucleoid — SUPPLEMENTARY DATA 

# Synonymous codon bias and functional constraint on GC3-related DNA backbone dynamics in the prokaryotic nucleoid

## SUPPLEMENTARY DATA

**Files in this Data Supplement:**

- SUPPLEMENTARY DATA
