# Supplementary material for: Synonymous codon bias and functional constraint on GC3-related DNA backbone dynamics in the prokaryotic nucleoid
Source: Nucleic Acids Res. 2014 Sep 8;42(17):10915–26. doi: 10.1093/nar/gku811 (PMC4176184; doi:10.1093/nar/gku811)
Supplement: SUPPLEMENTARY DATA [file supp_gku811_nar-01772-z-2014-File009.zip › NAR-01772-Z-2014.R1 Suppl files/SuppFigA.pdf]

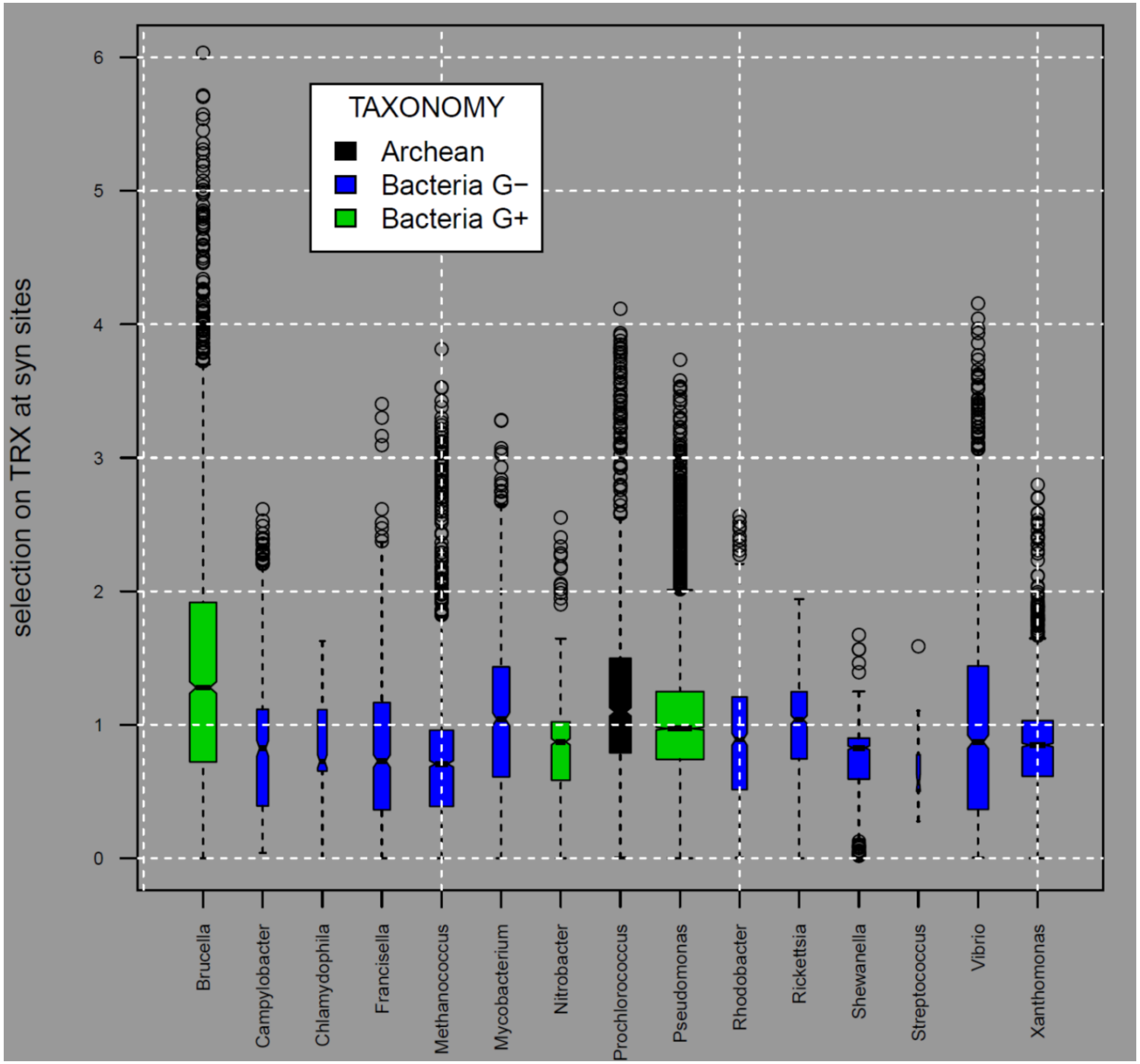

Supplemental Figure A. Boxplots of prokaryotic distributions of the selection metric targeting intrinsic DNA flexibility at synonymous sites. All 35000+ aligned gene sequences were obtained from the ATGC database (Novichkov et al. 2009).
