# Supplementary material for: Synonymous codon bias and functional constraint on GC3-related DNA backbone dynamics in the prokaryotic nucleoid
Source: Nucleic Acids Res. 2014 Sep 8;42(17):10915–26. doi: 10.1093/nar/gku811 (PMC4176184; doi:10.1093/nar/gku811)
Supplement: SUPPLEMENTARY DATA [file supp_gku811_nar-01772-z-2014-File009.zip › NAR-01772-Z-2014.R1 Suppl files/SuppFileA.pdf]

Supplementary File A - A simple and efficient Perl script for randomly repositioning mutations without altering base composition

```
#!usr/bin/perl

use List::Util qw( shuffle );

use Time::HiRes qw( gettimeofday );

$obsSHUFFLE = "n";    # to observe shuffling on seqs (y = yes n = no)

$shuffleINDELS = "n"; # to shuffle indels as well as subs

$windowSize = 100;    # size of window to shuffle within

$shufflecount  = 1000;  # number of shuffled sequences to produce


##########################################################################

##### The main script takes processes the given FASTA file      #####

##### and stores the sequences in a hash before passing them    #####

##### on to the shuffle function. Note that the FASTA file      #####

##### should only contain two sequences. The first sequence     #####

##### should be the query sequence, i.e. the sequence of        #####

##### interest containing the mutations. The second sequence    #####

##### should be the reference sequence.                         #####

##########################################################################


### check commandline arguments ###

die "Usage: $0 <fasta file> <output file name>\n" if @ARGV != 2;


### obtain sequences to work with in this implementation ###

$filename = @ARGV[0];

$output = @ARGV[1];

open(FILE, $filename) or die "Cannot open file";
```

```
%sequences = ();

$name = "";

$q = "";

$ref = "";


while($line = <FILE>){
	if($line =~ m/^\n/ || $line =~ m/^\s/){		### don't use empty lines ###


	} elsif($line =~ m/>/) {		### use sequence header as key ###


		if($q eq ""){
			$q = $line;
			$q =~ s/>//;
			$q =~ s/\n//;
			$name = $q;
		} else {
			$ref = $line;
			$ref =~ s/>//;
			$ref =~ s/\n//;
			$name = $ref;
		}


	} else {


		$tmp = $line;
		$tmp =~ s/\n//;


		$sequences{$name}
```

```
                                    = $sequences{$name} . $tmp;


  }
}
close(FILE);


### time how long it takes to do 1000 randomizations
### on one given sequence, as well as storing the resulting
### randomizations in a file
$timeOne = int( gettimeofday() * 1000);
open(STORE, $outputfile);
print STORE "original seqs\n";
print STORE $q . "\n";
print STORE $sequences{$q};
print STORE "\n";
print STORE $ref . "\n";
print STORE $sequences{$ref};
print STORE "\n\n";
print STORE "shuffled seqs\n";
for($n = 0; $n<$shufflecount; $n++){
        print STORE "$n\n";
   print STORE shiftMutations($sequences{$ref}, $sequences{$q}, 100),
                "\n";
}
close(STORE);
$timeTwo = int( gettimeofday() * 1000);


print $timeTwo-$timeOne, " msec\n";
```

```
######################################################################
##### This code is intended to shuffle substitution mutations      #####
##### by comparing two given sequences, the first of which                  #####
##### is used as an ancestral/reference sequence to identify        #####
##### mutations in the second sequence. Substitution                        #####
##### mutations are then relocated from the place they were                #####
##### found to a random location on a copy of the second                    #####
##### sequence. The nucleotide at the random location in the     #####
##### reference sequence is the same nucleotide in the                      #####
##### reference sequence as found at the location of the                    #####
##### substitution mutation. An additional option exists that      #####
##### also shuffles indels, given by the subroutine                         #####
##### 'shiftIndels'. The subroutine returns the randomized                  #####
##### sequence.
                    #####
######################################################################
sub shiftMutations {


        $reference = ($_[0]);                   ### the 'template' sequence
        $query = ($_[1]);                               ### the sequence containing mutations
        $shuffledSeq = ($_[1]);                 ### the sequence to which the changes
                                                                ###                     are made, based on the
query


        #$windowSize = uc($_[2]);                        ### the size of the window
```

```
#print "$windowSize\n";


### determine the length of the shortest sequence even
### though you would expect the two given sequences to be of
### the same length
$shortestLength;
if(length($reference) < length($query)){
	$shortestLength = length($reference);
} else {
	$shortestLength = length($query);
}



### traverse the entire length of the sequence by moving from
### window to window
$windowPos = 0;				### starting position of the current window
$endLocation = 0;			### ending position of the current window
$done = "false";
while($done ne "true"){

	### adjust the ending position relative to the window size
	### but use the remaining length of the sequence when it
	### is shorter than the given window size
	if( ($windowPos + $windowSize) < $shortestLength ){
		$endLocation = $windowPos + $windowSize;
	} else {
		$endLocation = $shortestLength;
		$done = "true";
```

```
}

@A_locations = ();                    ### locations of 'A's in window
@C_locations = ();                    ### locations of 'C's in window
@G_locations = ();                    ### locations of 'G's in window
@T_locations = ();                    ### locations of 'T's in window


### fill the nucLocations collection with all the individual
### nucleotide locations found in the reference sequence
### within the current window, disregarding indels
for($i = $windowPos; $i<$endLocation; $i++){
        $nucleotide = substr($reference, $i, 1);
        if(substr($query, $i, 1) !~ m/-/
                && ($nucleotide eq substr($query, $i, 1))){

                if( $nucleotide eq "A" ){
                        push(@A_locations, $i);
                } elsif( $nucleotide eq "C" ){
                        push(@C_locations, $i);
                } elsif( $nucleotide eq "G" ){
                        push(@G_locations, $i);
                } elsif( $nucleotide eq "T" ){
                        push(@T_locations, $i);
                }
                if( $nucleotide eq "a" ){
                        push(@A_locations, $i);
                } elsif( $nucleotide eq "c" ){
```

```
                push(@C_locations, $i);
            } elsif( $nucleotide eq "g" ){
                push(@G_locations, $i);
            } elsif( $nucleotide eq "t" ){
                push(@T_locations, $i);
            }
        }
}

### randomize the nucleotide locations
@A_locations = shuffle(@A_locations);
@C_locations = shuffle(@C_locations);
@G_locations = shuffle(@G_locations);
@T_locations = shuffle(@T_locations);



for($i = $windowPos; $i<$endLocation; $i++){

        ### swap mutations only if you find a substitution mutation
        if( substr($reference, $i, 1) ne substr($query, $i, 1)
            && substr($reference, $i, 1) !~ m/-/
            && substr($query, $i, 1) !~ m/-/){

            $referenceBase = substr($reference, $i, 1);
            $mutatedBase = substr($query, $i, 1);

            ### remove and return first element of shuffled
            ### nucleotide locations to determine where to
```

```
			### replace the mutation
			$newLocation;
			if($referenceBase eq "A"){
				$newLocation = shift(@A_locations);
			} elsif($referenceBase eq "C"){
				$newLocation = shift(@C_locations);
			} elsif($referenceBase eq "G"){
				$newLocation = shift(@G_locations);
			} elsif($referenceBase eq "T"){
				$newLocation = shift(@T_locations);
			}
			if($referenceBase eq "a"){
				$newLocation = shift(@A_locations);
			} elsif($referenceBase eq "c"){
				$newLocation = shift(@C_locations);
			} elsif($referenceBase eq "g"){
				$newLocation = shift(@G_locations);
			} elsif($referenceBase eq "t"){
				$newLocation = shift(@T_locations);
			}

			### perform the actual swap
			$baseToSwap = substr($query, $newLocation, 1);

if ($obsSHUFFLE ne "y"){
  substr($shuffledSeq, $newLocation, 1, ($mutatedBase));
				}
if ($obsSHUFFLE eq "y"){
```

```
if ($baseToSwap eq "a" || $baseToSwap eq "c" || $baseToSwap eq "g" || $$baseToSwap eq "t"){
  substr($shuffledSeq, $newLocation, 1, (uc $mutatedBase));  # to observe shuffling on sequences
  }
if ($baseToSwap eq "A" || $baseToSwap eq "C" || $baseToSwap eq "G" || $baseToSwap eq "T"){
  substr($shuffledSeq, $newLocation, 1, (lc $mutatedBase));
  }
}


substr($shuffledSeq, $i, 1, ($baseToSwap));


                  }
              }
              ### move to next window
              $windowPos += $windowSize;
    }



    #print substr($reference, 0, $windowSize), "\n\n";
    #print substr($query, 0, $windowSize), "\n\n";
    #print substr($shuffledSeq, 0, $windowSize), "\n\n";

    ### shuffle indels around as well
    if ($shuffleINDELS eq "y"){
$shuffledSeq = shiftIndels($reference, $shuffledSeq, $windowSize);
    }
    #print substr($shuffledSeq, 0, $windowSize), "\n";
```

```
	return $shuffledSeq;
}



######################################################################
##### This subroutine shuffles around indels. It handles             #####
##### insertions by identifying '-'s in the reference                #####
##### sequence, extracting the corresponding 'word' in the           #####
##### query sequence, concatinating the surrounding sequence #####
##### ends, and inserting the found 'word' at a new random           #####
##### location within the current window. Deletions, on the      #####
##### other hand, are dealt with by identifying '-'s in the          #####
##### query sequence, determining the length of the gap,             #####
##### replacing the '-'s in the query sequence with the              #####
##### corresponding nucleotides from the reference sequence, #####
##### and then replacing nucleotides on the query sequence at  #####
##### a random location with a gap of the same length.               #####
######################################################################
sub shiftIndels {

	$reference = $_[0];		### the 'template' sequence
	$query = $_[1];			### the sequence containing mutations
	$indelShiftSeq = $_[1];		### the sequence to which the changes
						###		are made

	#$windowSize = $_[2];		### the window size

	### determine the length of the shortest sequence even
```

```
### though you would expect the two given sequences to be of
### the same length
$shortestLength;
if(length($reference) < length($query)){
	$shortestLength = length($reference);
} else {
	$shortestLength = length($query);
}

### traverse the entire length of the sequence by moving from
### window to window
$windowPos = 0;				### starting postition of current window
$endLocation = 0;			### ending position of current window
$done = "false";
while($done ne "true"){

	### adjust the ending position relative to the window size
	### but use the remaining length of the sequence when it
	### is shorter than the given window size
	if( ($windowPos + $windowSize) < $shortestLength ){
		$endLocation = $windowPos + $windowSize;
	} else {
		$endLocation = $shortestLength;
		$done = "true";
	}

	for($i = $windowPos; $i<$endLocation; $i++){
```

```
### handle insertions, seens as '-' in the reference sequence
### making sure that the current '-' is not part of a larger,
### previously handled insertion
if( substr($reference, $i, 1) eq "-"
        && substr($reference, $i-1, 1) ne "-"){

        ### determine length of the insertion
        $nextBase = substr($reference, $i+1, 1);
        $length = 1;
        while($nextBase eq "-"){
                $length++;
                $nextBase = substr($reference, $i+$length, 1);
        }
        $insertion = substr($query, $i, $length);

        ### concatenate sequence around insertion
        $indelShiftSeq = substr($indelShiftSeq, 0, $i)
                . substr($indelShiftSeq, $i+$length);

        ### place insertion at random different location
        ### that is not the current position but within the
        ### current window
        $newLocation = int(rand($windowSize-$length))
                + $windowPos;
        while($newLocation == $i
                && (substr($query, $newLocation, 1) ne "-")){
                $newLocation = int(rand($windowSize-$length))
                        + $windowPos;
```

```
        }
        $indelShiftSeq = substr($indelShiftSeq, 0, $newLocation)
                . $insertion . substr($indelShiftSeq, $newLocation);
}

### handle deletions, seen as '-' in the query sequence
### making sure that the current '-' is not part of a larger,
### previously handled deletion
elsif( substr($query, $i, 1) eq "-"
        && substr($query, $i-1, 1) ne "-"){

        ### determine length of the deletion
        $nextBase = substr($query, $i+1, 1);
        $length = 1;
        while($nextBase eq "-"){
                $length++;
                $nextBase = substr($query, $i+$length, 1);
        }
        $deletion = substr($reference, $i, $length);

        ### replace gap with deleted sequence
        substr($indelShiftSeq, $i, $length, $deletion);

        ### create a new gap of the same length at a random position
        $newLocation = int(rand($windowSize-$length))
                + $windowPos;
        while($newLocation == $i
                && (substr($reference, $newLocation, $length) =~ m/-/)){
```

```
                    $newLocation = int(rand($windowSize-$length))
                        + $windowPos;
                }

                $gapSequence = "-";
                for($j = 1; $j<$length; $j++){
                    $gapSequence = $gapSequence . "-";
                }

                substr($indelShiftSeq, $newLocation, $length, $gapSequence);
            }
        }
        ### move to next window
        $windowPos += $windowSize;
    }

    return $indelShiftSeq;

}
```
