# Supplementary material for: Synonymous codon bias and functional constraint on GC3-related DNA backbone dynamics in the prokaryotic nucleoid
Source: Nucleic Acids Res. 2014 Sep 8;42(17):10915–26. doi: 10.1093/nar/gku811 (PMC4176184; doi:10.1093/nar/gku811)
Supplement: SUPPLEMENTARY DATA [file supp_gku811_nar-01772-z-2014-File009.zip › NAR-01772-Z-2014.R1 Suppl files/SuppFileB_caption.pdf]

Supplementary File B - The intrinsic DNA flexibility and GC3 of the diaminopimelate aminotransferase (DapL) gene from all species represented in Figure 6A. The plots below are read identically to those in Figure 4. Plots include observed (red), minimum (green), maximum (blue) and average synonymous (black)intrinsic flexibility TRX scores and respective trends in overall GC content (black), GC content in each reading frame (gray) and GC3 (green).

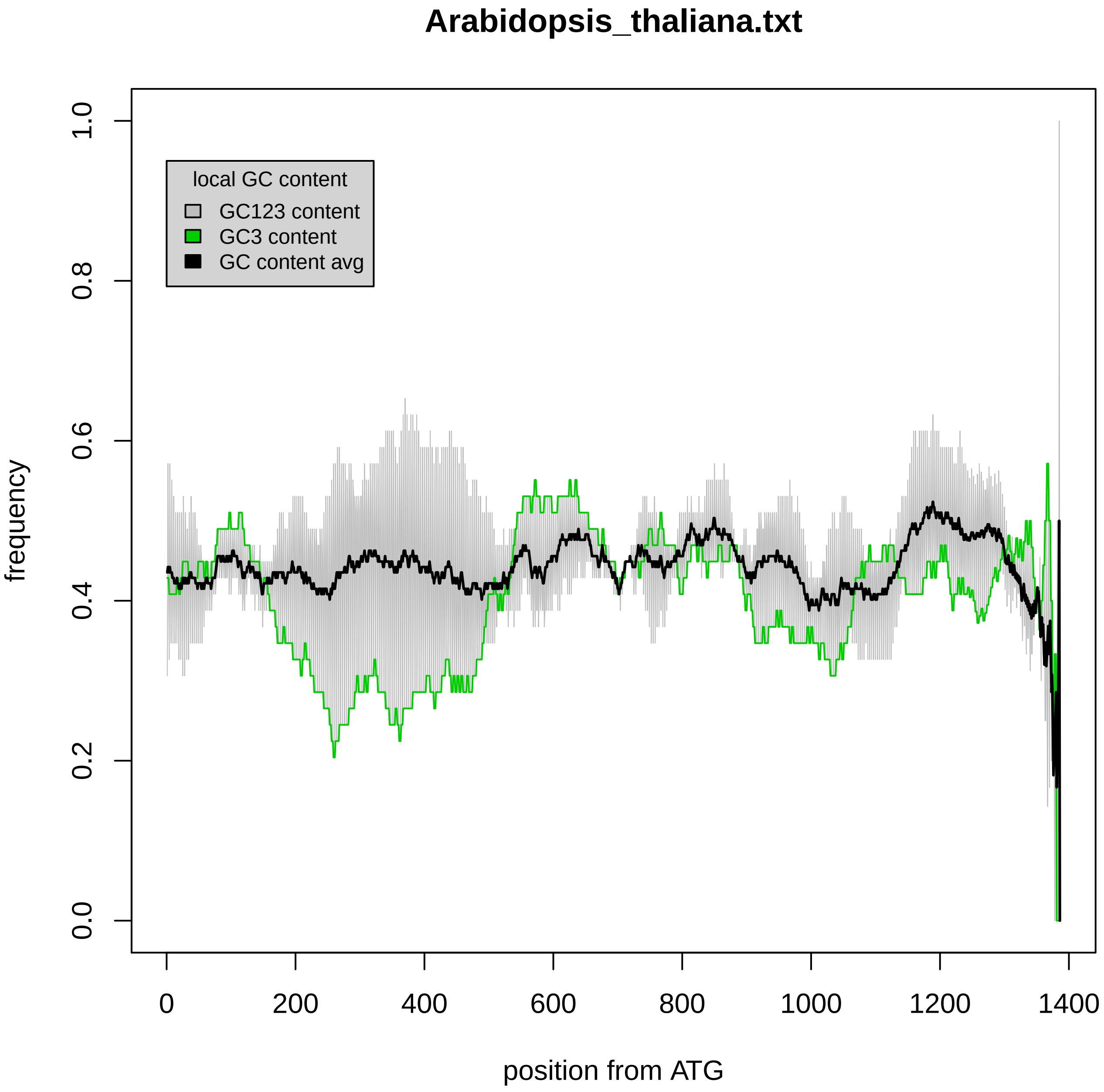
Arabidopsis_thaliana.txt
local GC content
GC123 content
GC3 content
GC content avg
frequency
0.0
0.2
0.4
0.6
0.8
1.0
0
200
400
600
800
1000
1200
1400
position from ATG

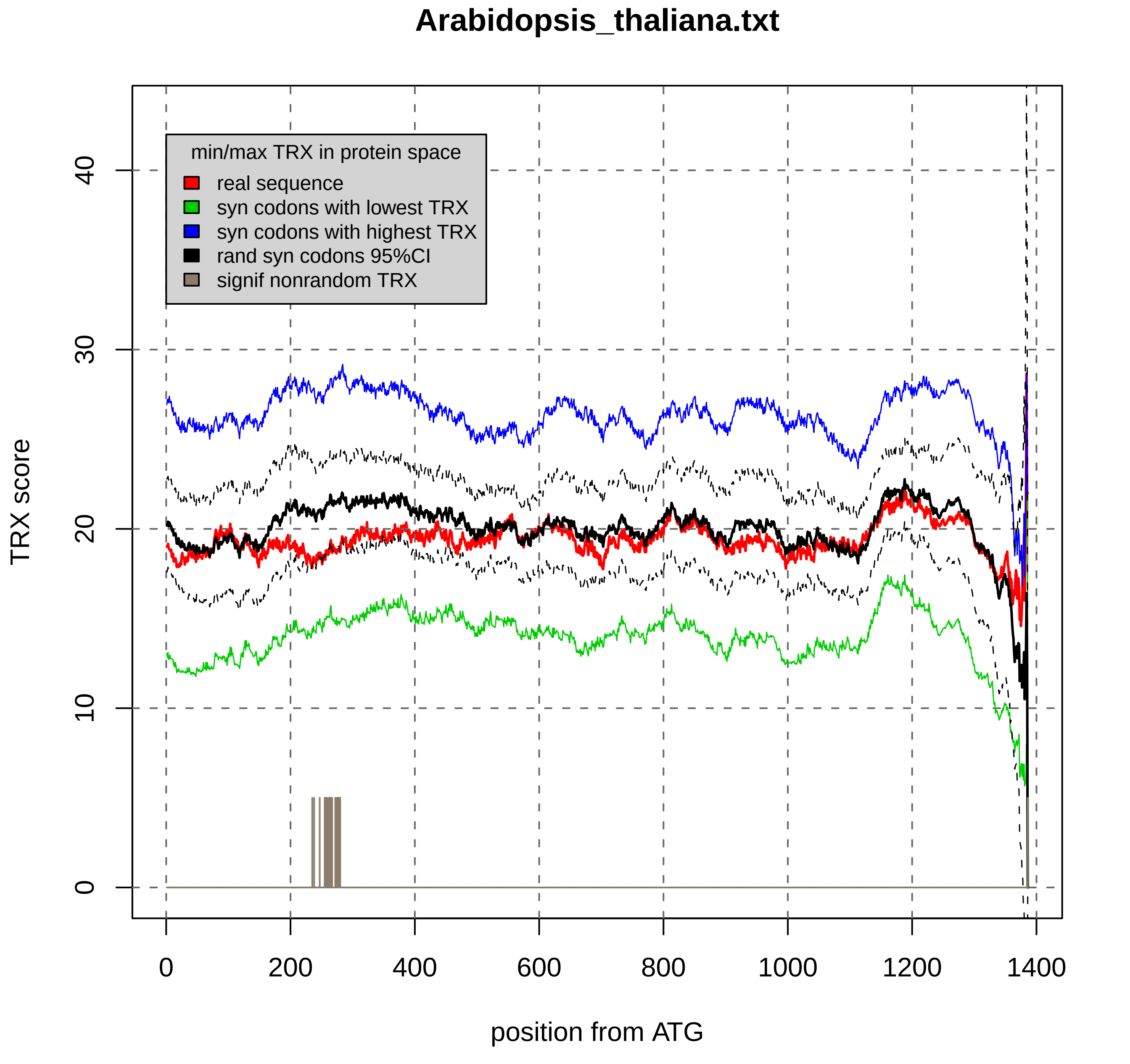

Arabidopsis_thaliana.txt
min/max TRX in protein space
real sequence
syn codons with lowest TRX
syn codons with highest TRX
rand syn codons 95%CI
signif nonrandom TRX
TRX score
0
10
20
30
40
0
200
400
600
800
1000
1200
1400
position from ATG

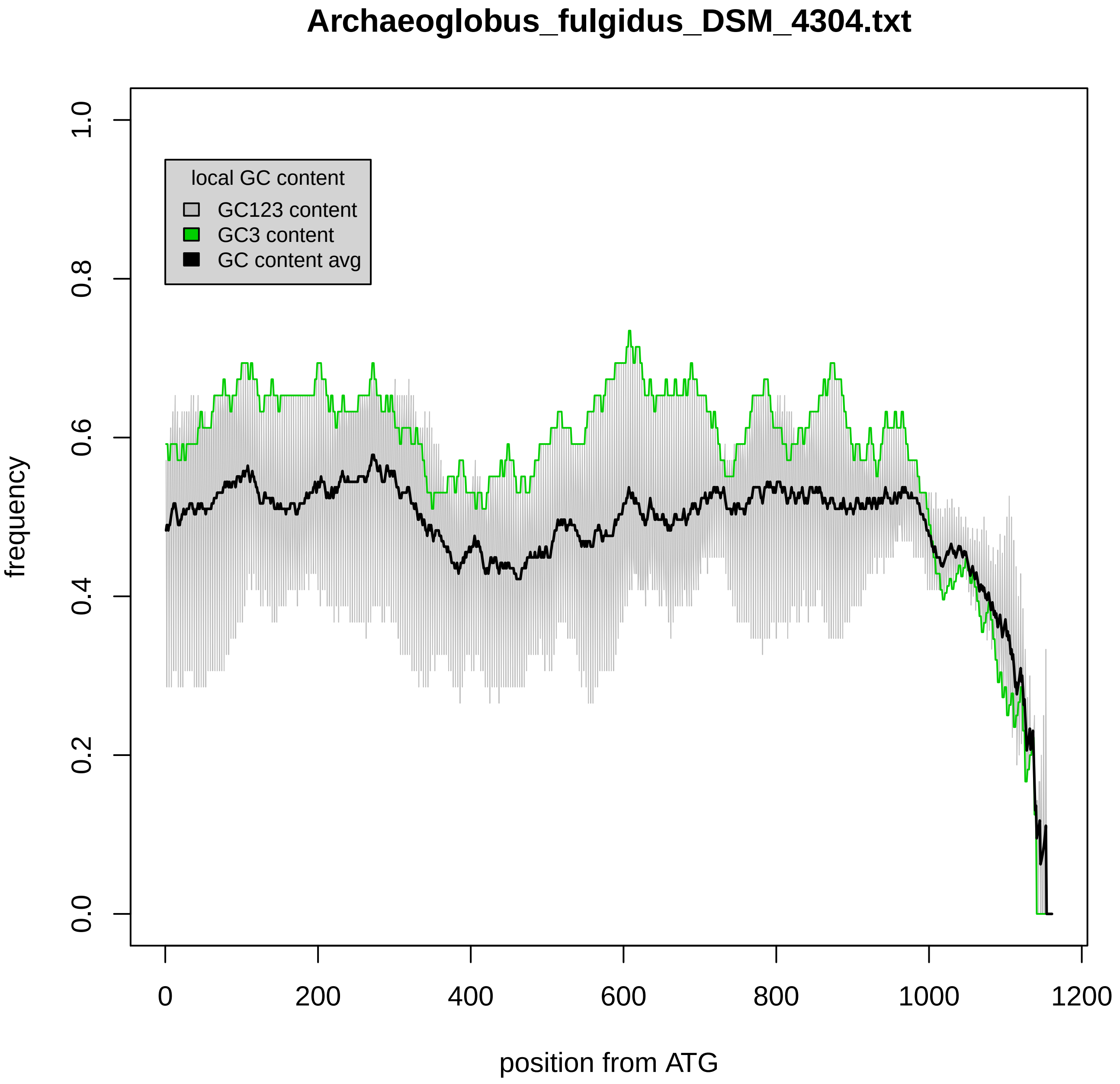

Archaeoglobus_fulgidus_DSM_4304.txt
local GC content
GC123 content
GC3 content
GC content avg
frequency
position from ATG
0.0
0.2
0.4
0.6
0.8
1.0
0
200
400
600
800
1000
1200

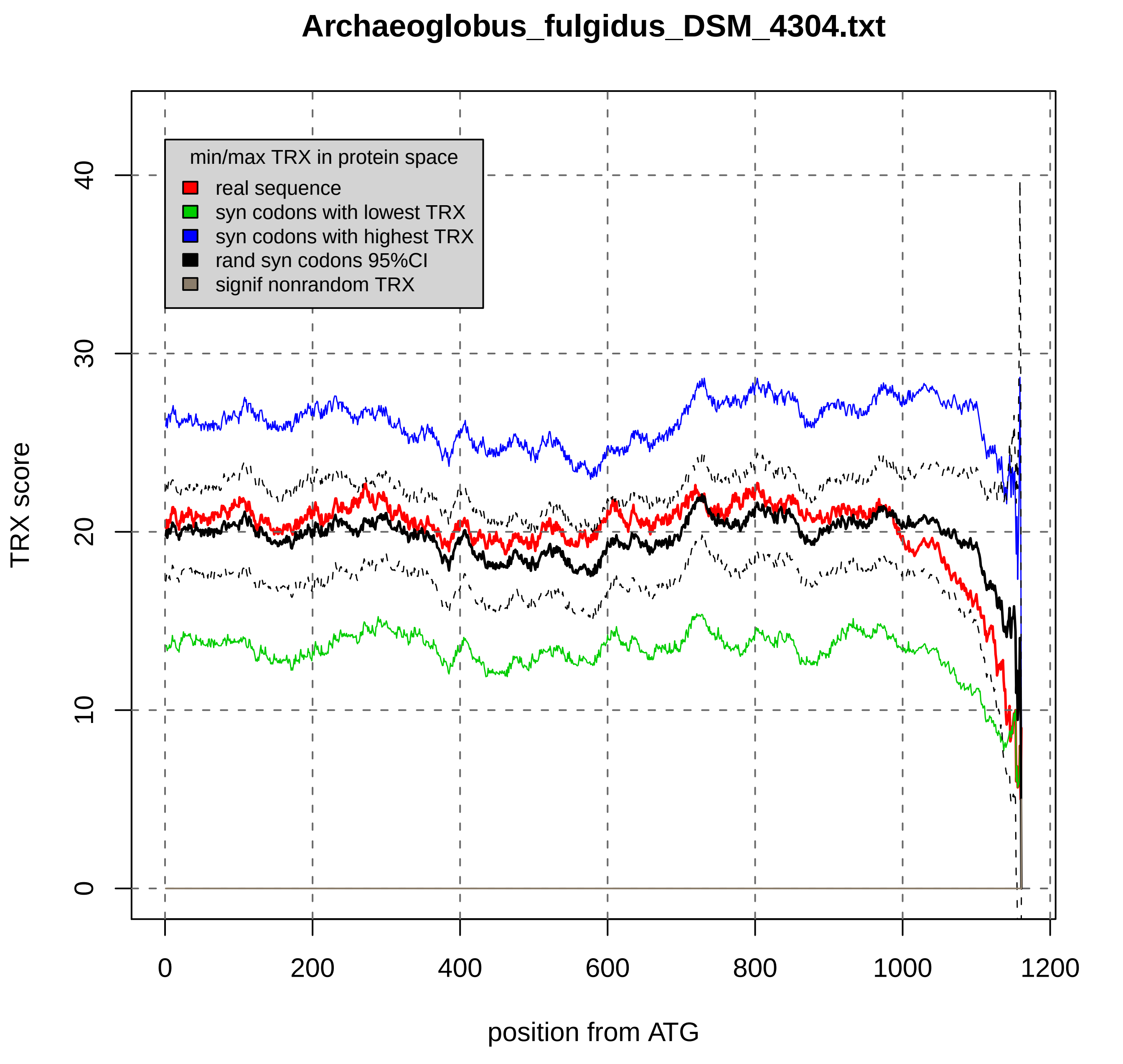

Archaeoglobus_fulgidus_DSM_4304.txt
min/max TRX in protein space
real sequence
syn codons with lowest TRX
syn codons with highest TRX
rand syn codons 95%CI
signif nonrandom TRX
TRX score
0
10
20
30
40
0
200
400
600
800
1000
1200
position from ATG

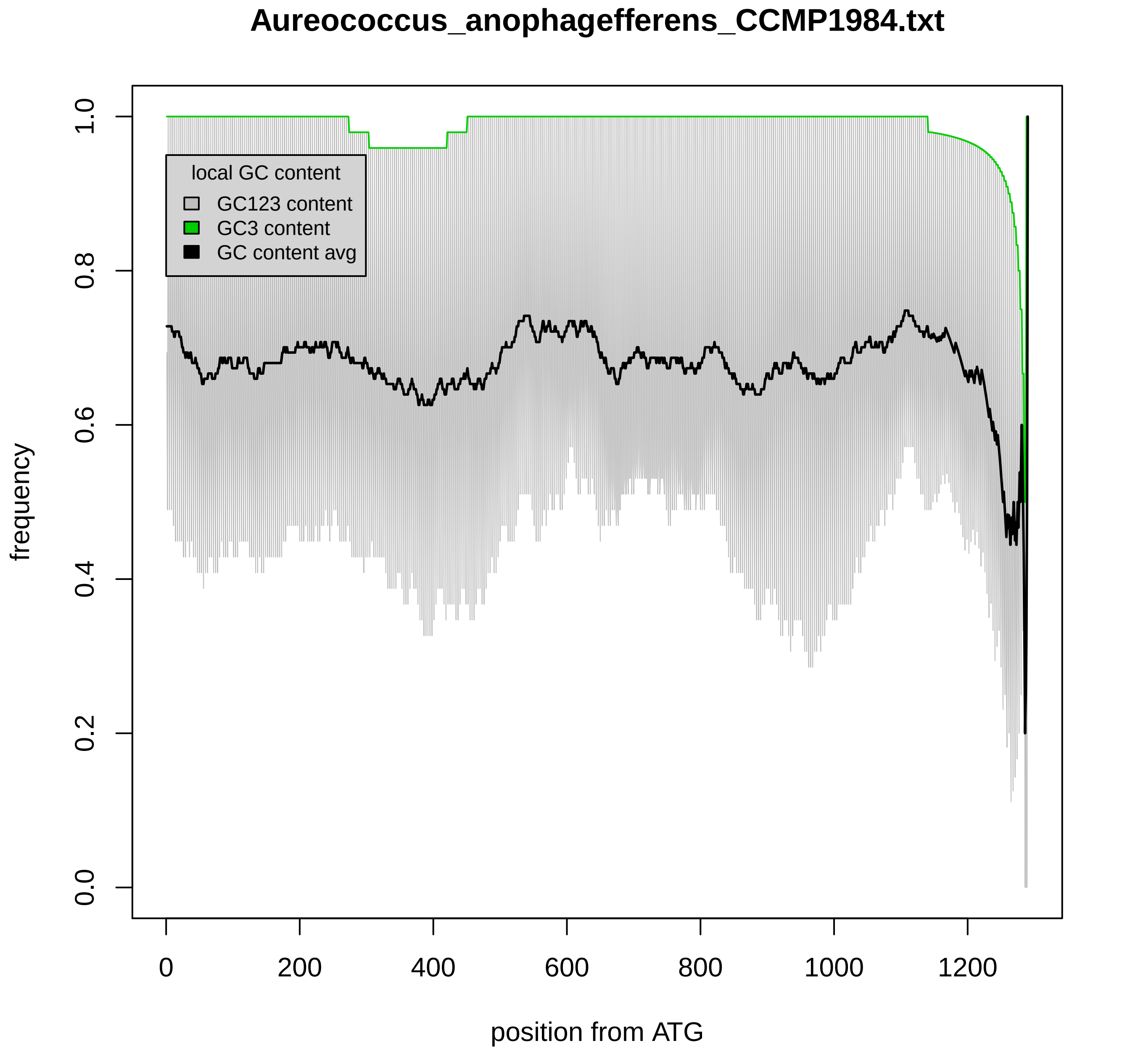

Aureococcus_anophagefferens_CCMP1984.txt
local GC content
GC123 content
GC3 content
GC content avg
frequency
position from ATG
0.0
0.2
0.4
0.6
0.8
1.0
0
200
400
600
800
1000
1200

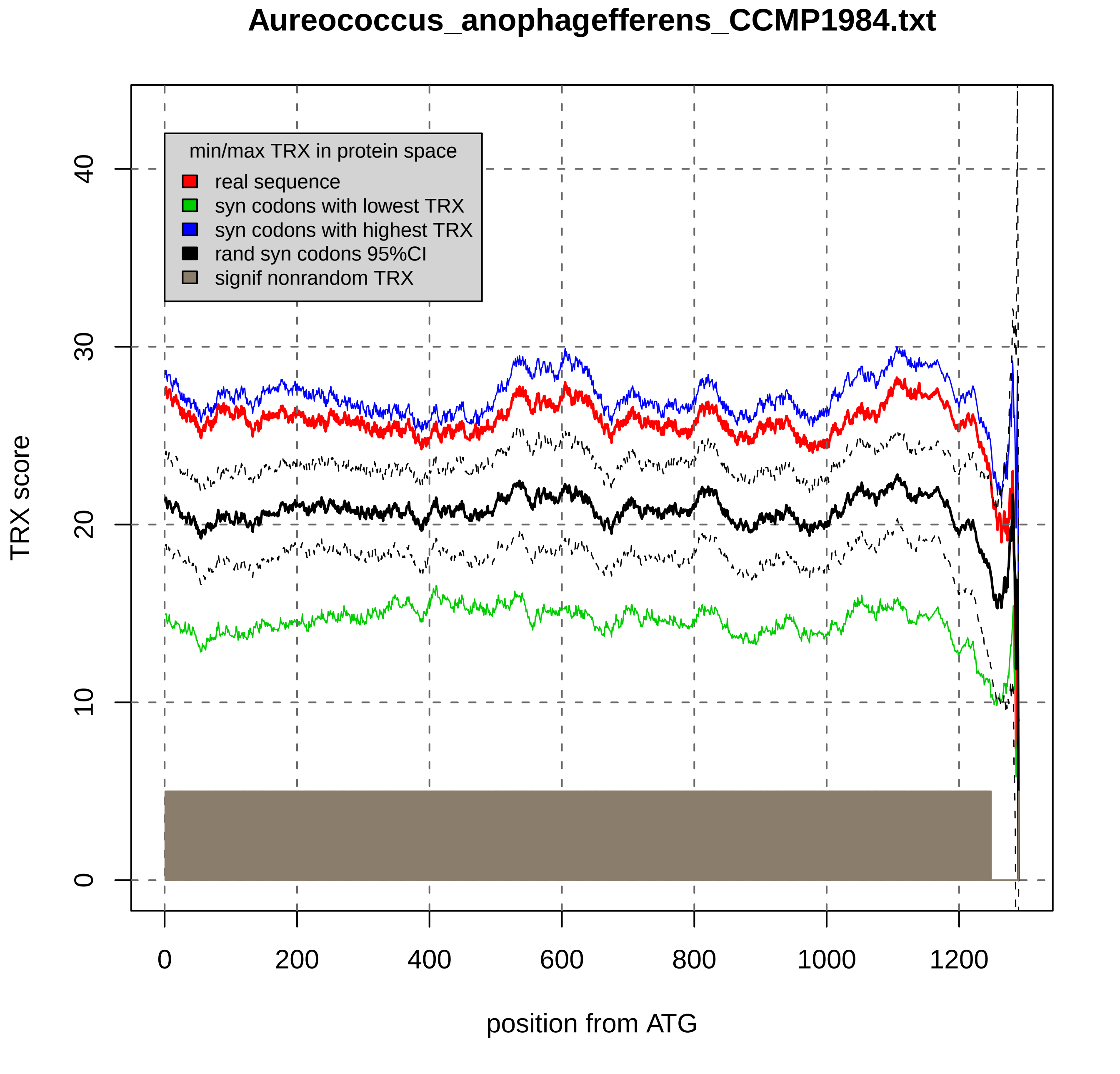
Aureococcus_anophagefferens_CCMP1984.txt
min/max TRX in protein space
real sequence
syn codons with lowest TRX
syn codons with highest TRX
rand syn codons 95%CI
signif nonrandom TRX
TRX score
position from ATG
0
10
20
30
40
0
200
400
600
800
1000
1200

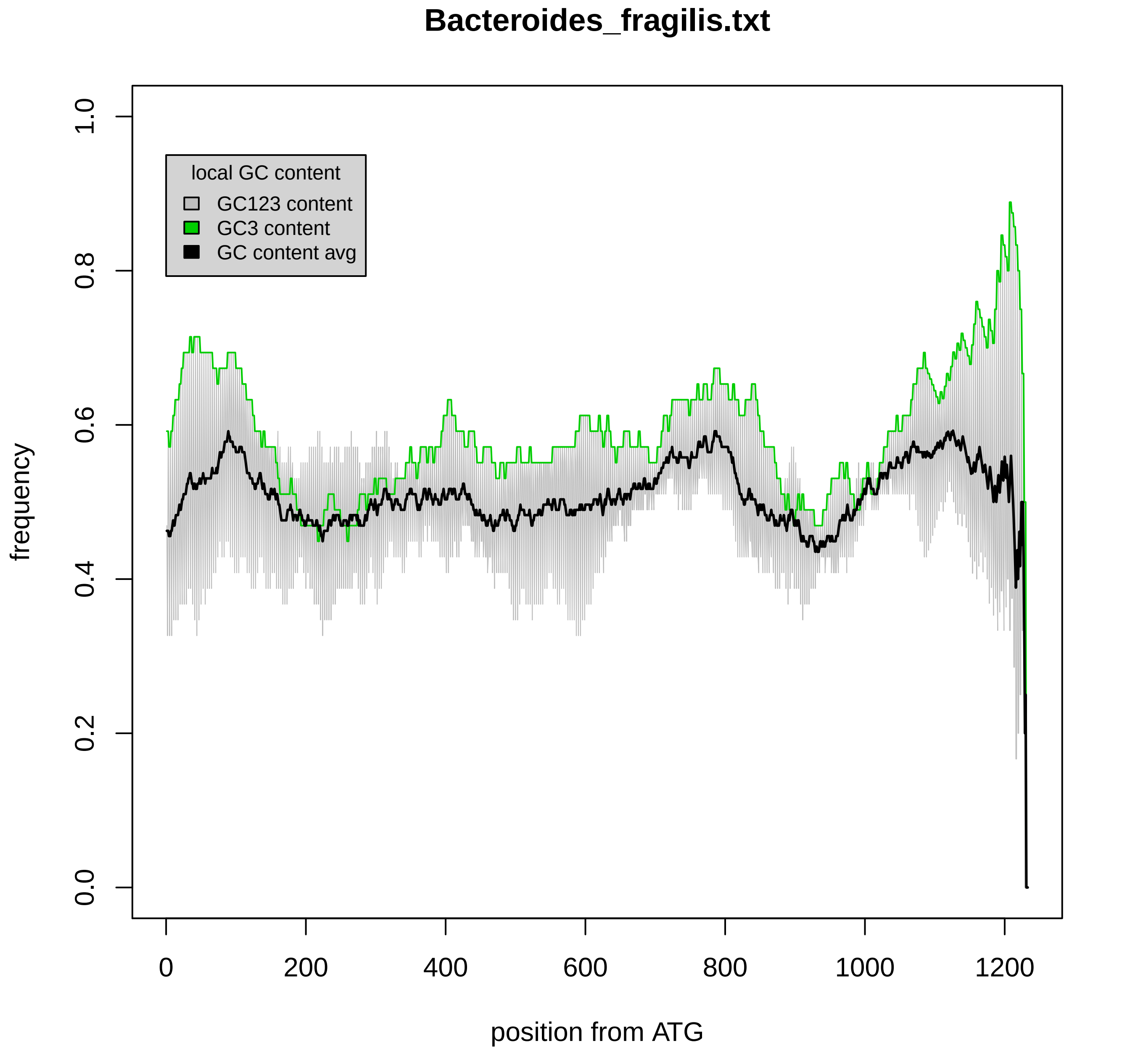

Bacteroides_fragilis.txt
local GC content
GC123 content
GC3 content
GC content avg
frequency
0.0
0.2
0.4
0.6
0.8
1.0
0
200
400
600
800
1000
1200
position from ATG

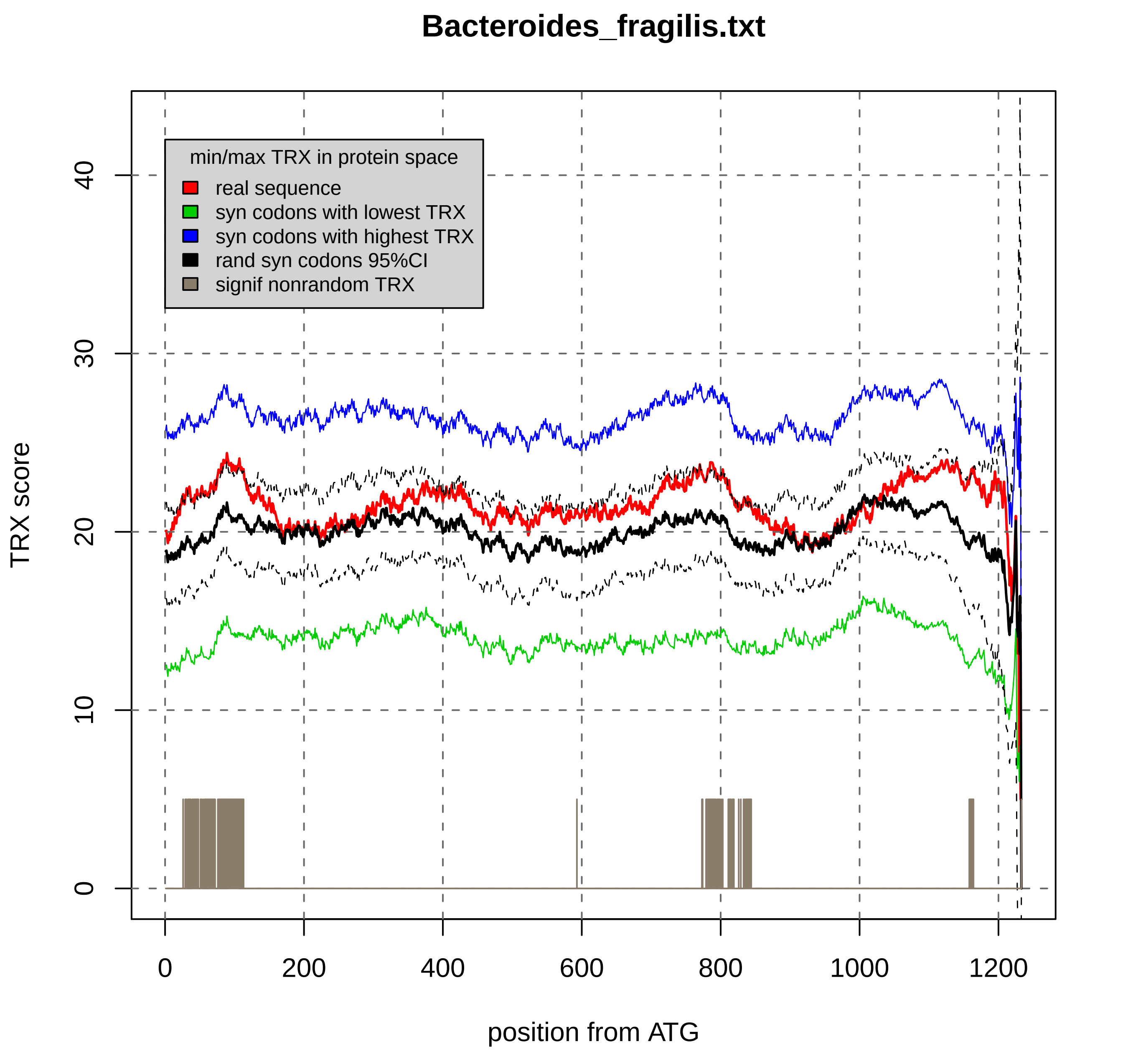
Bacteroides_fragilis.txt
min/max TRX in protein space
real sequence
syn codons with lowest TRX
syn codons with highest TRX
rand syn codons 95%CI
signif nonrandom TRX
TRX score
0
10
20
30
40
position from ATG
0
200
400
600
800
1000
1200

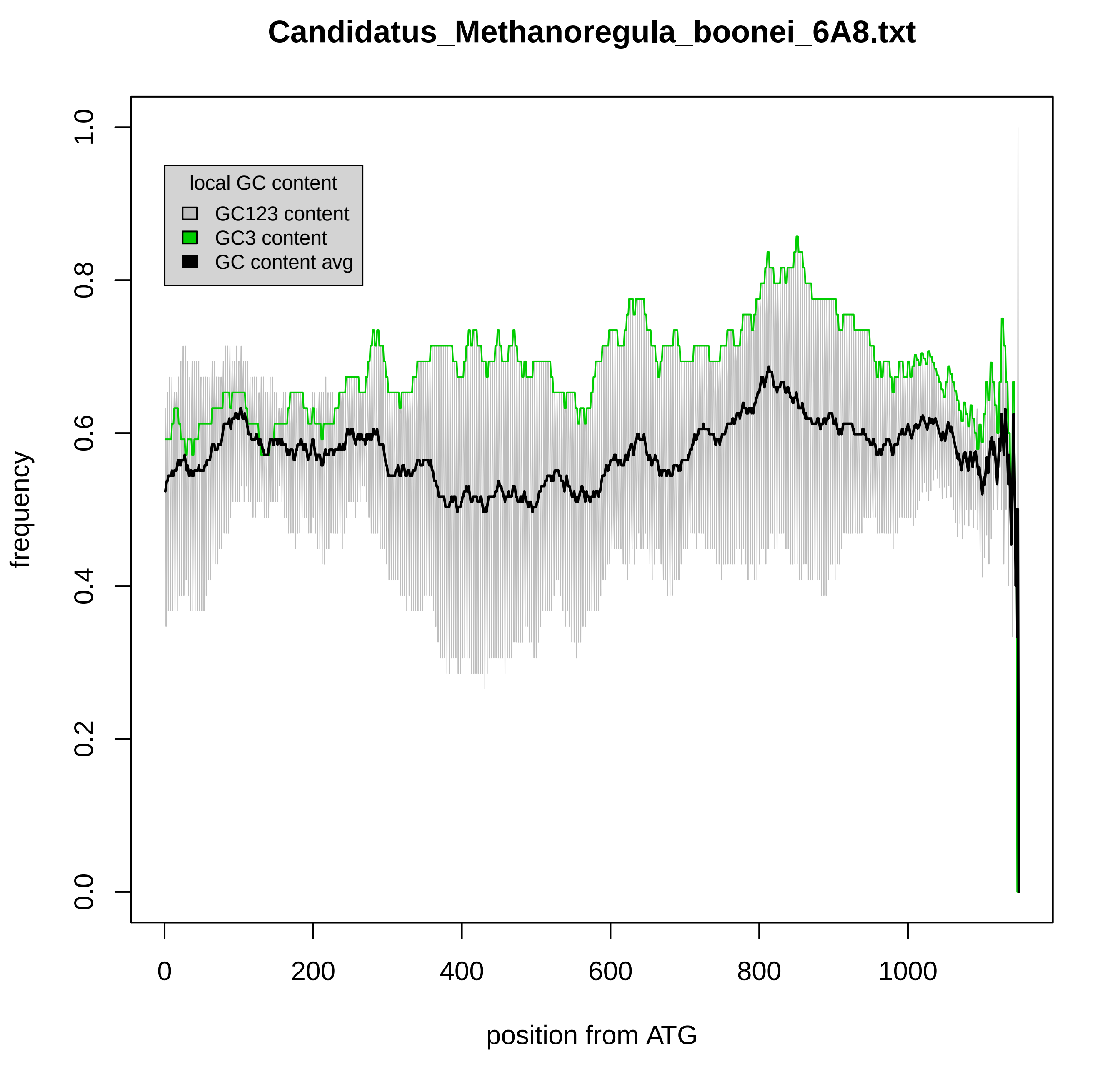

Candidatus_Methanoregula_boonei_6A8.txt
local GC content
GC123 content
GC3 content
GC content avg
frequency
position from ATG
0.0
0.2
0.4
0.6
0.8
1.0
0
200
400
600
800
1000

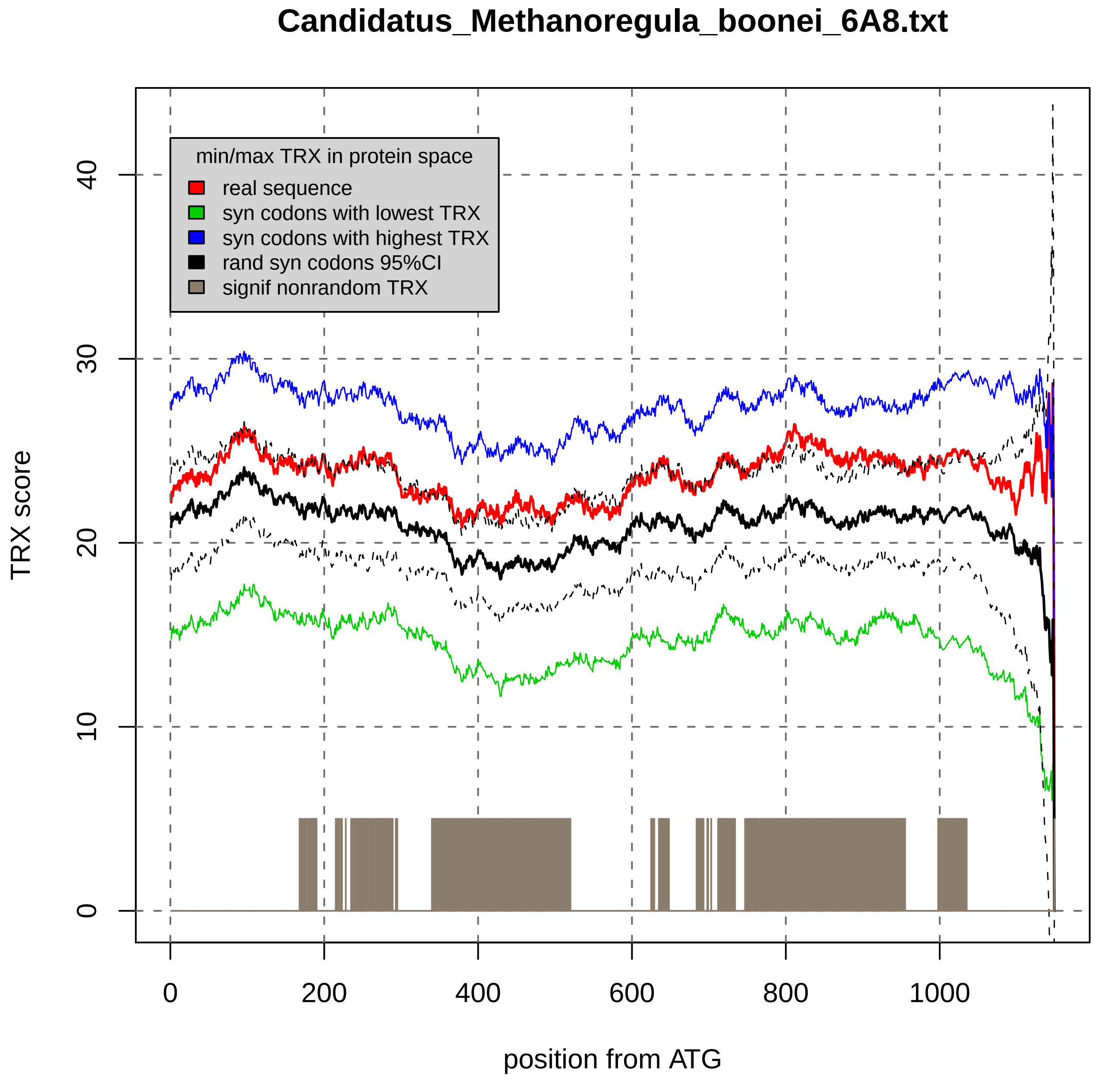

Candidatus_Methanoregula_boonei_6A8.txt
min/max TRX in protein space
real sequence
syn codons with lowest TRX
syn codons with highest TRX
rand syn codons 95%CI
signif nonrandom TRX
TRX score
position from ATG
0
10
20
30
40
0
200
400
600
800
1000

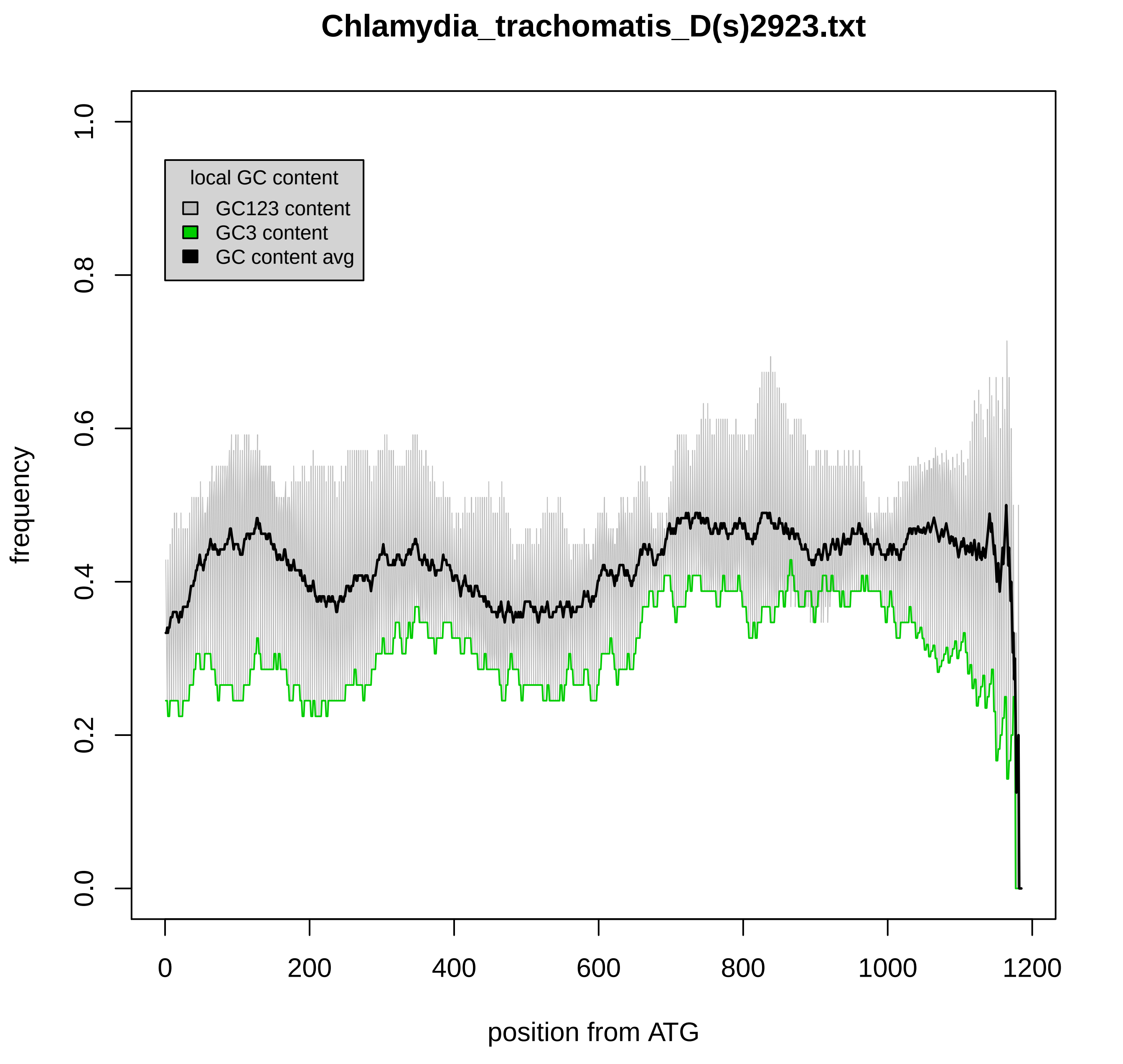
Chlamydia_trachomatis_D(s)2923.txt
local GC content
GC123 content
GC3 content
GC content avg
frequency
position from ATG
0.0
0.2
0.4
0.6
0.8
1.0
0
200
400
600
800
1000
1200

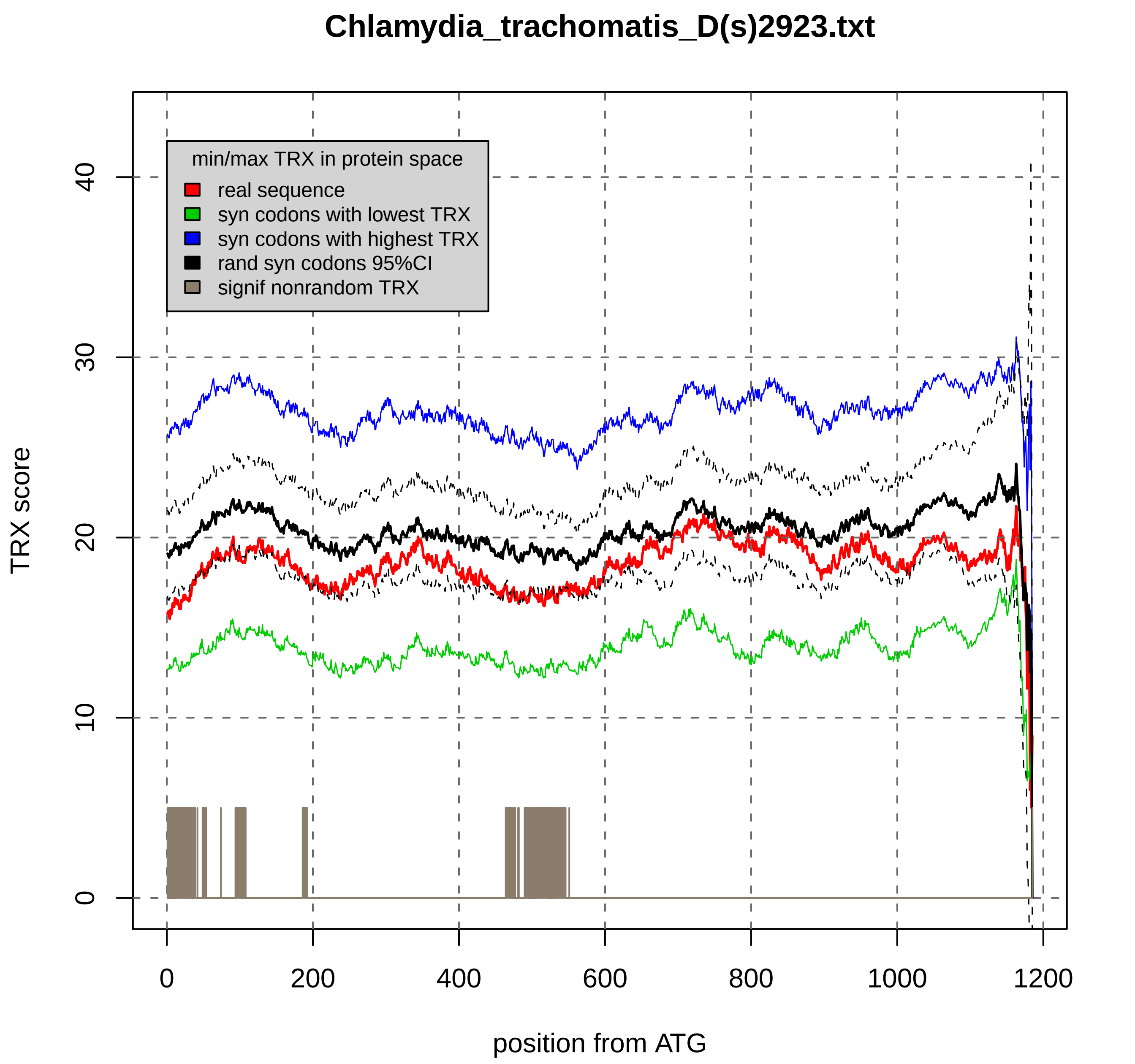
Chlamydia_trachomatis_D(s)2923.txt
min/max TRX in protein space
real sequence
syn codons with lowest TRX
syn codons with highest TRX
rand syn codons 95%CI
signif nonrandom TRX
TRX score
position from ATG
0
10
20
30
40
0
200
400
600
800
1000
1200

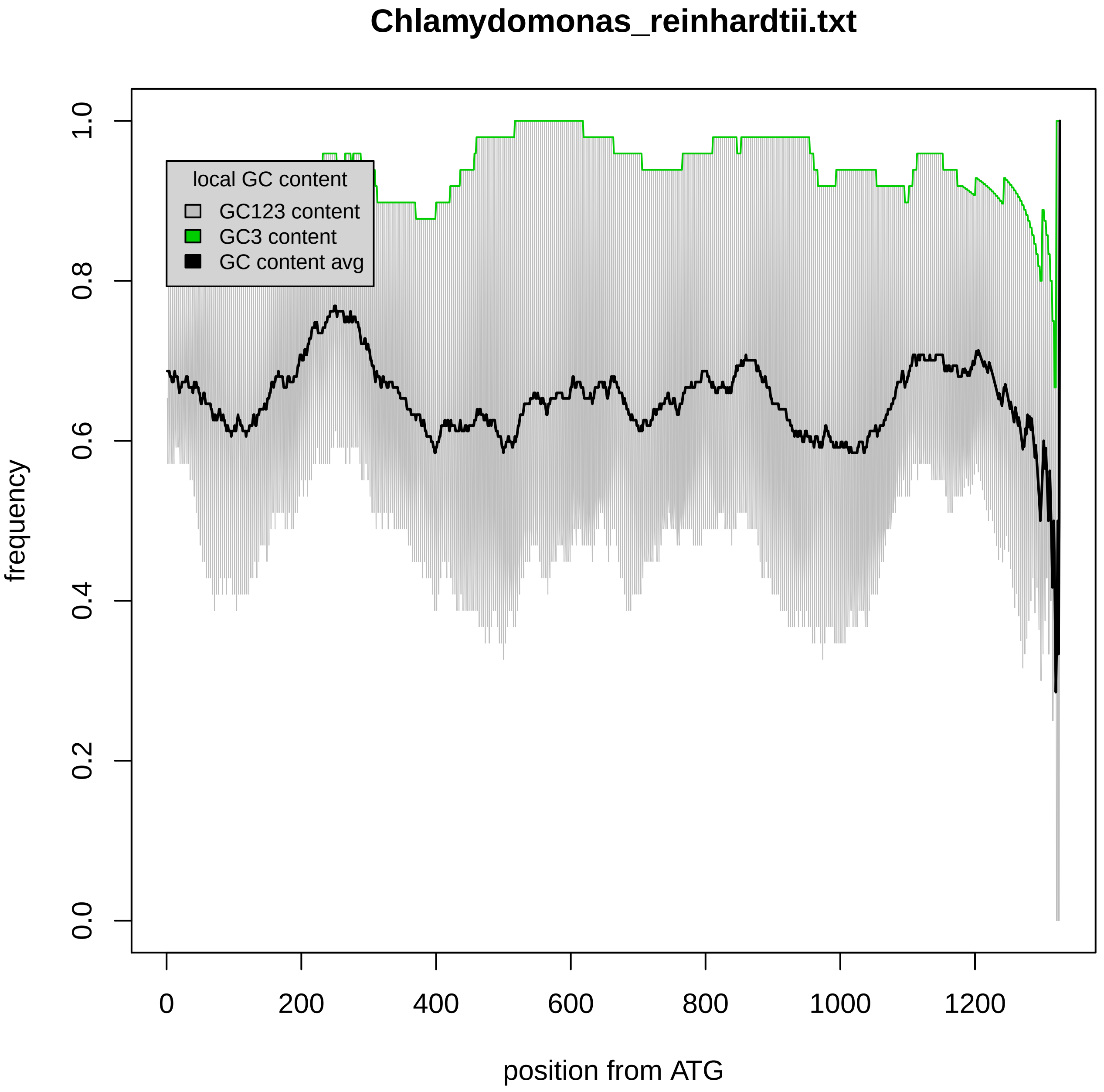

Chlamydomonas_reinhardtii.txt
local GC content
GC123 content
GC3 content
GC content avg
frequency
0.0
0.2
0.4
0.6
0.8
1.0
0
200
400
600
800
1000
1200
position from ATG

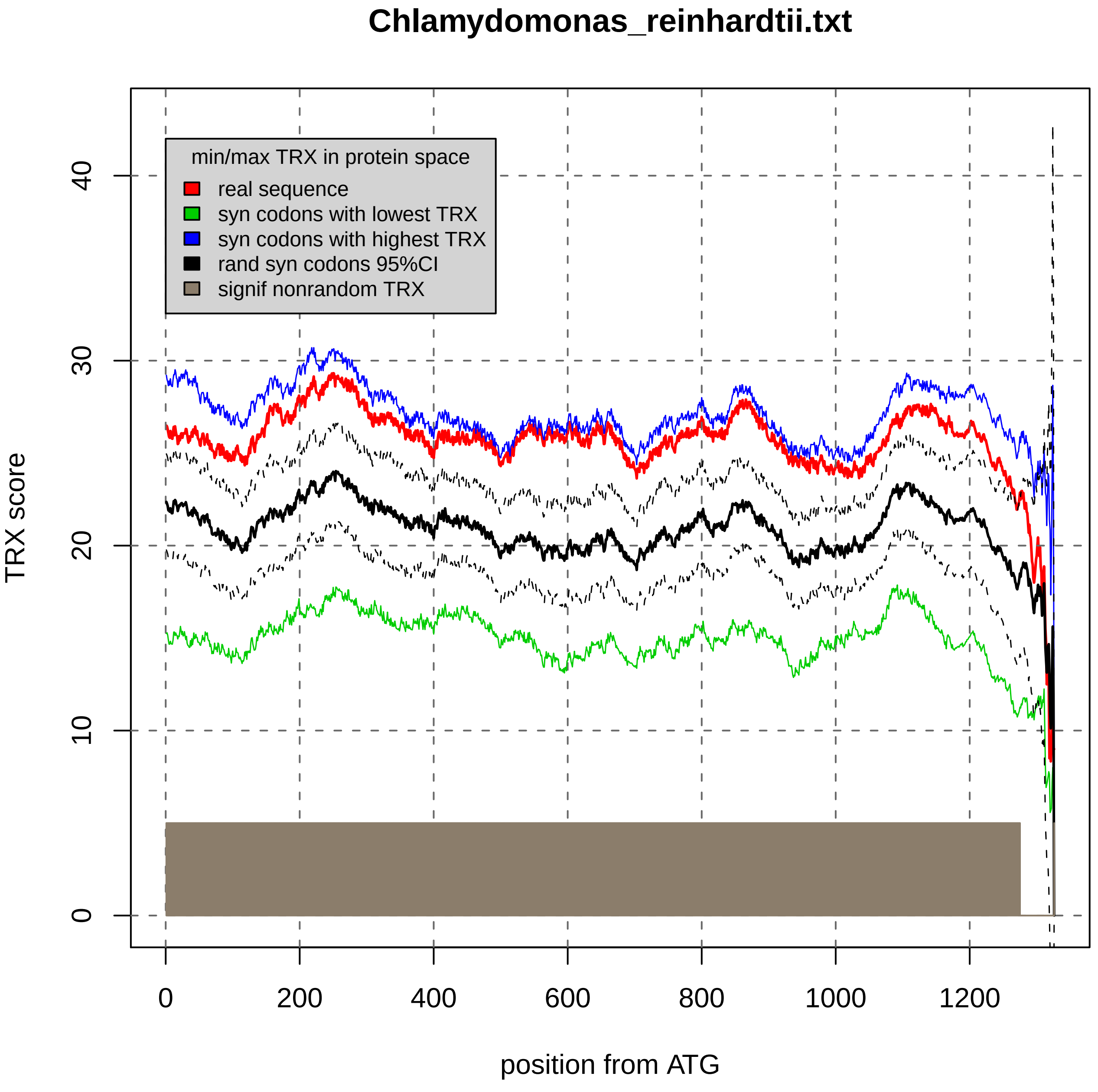

Chlamydomonas_reinhardtii.txt
min/max TRX in protein space
real sequence
syn codons with lowest TRX
syn codons with highest TRX
rand syn codons 95%CI
signif nonrandom TRX
TRX score
0
10
20
30
40
0
200
400
600
800
1000
1200
position from ATG

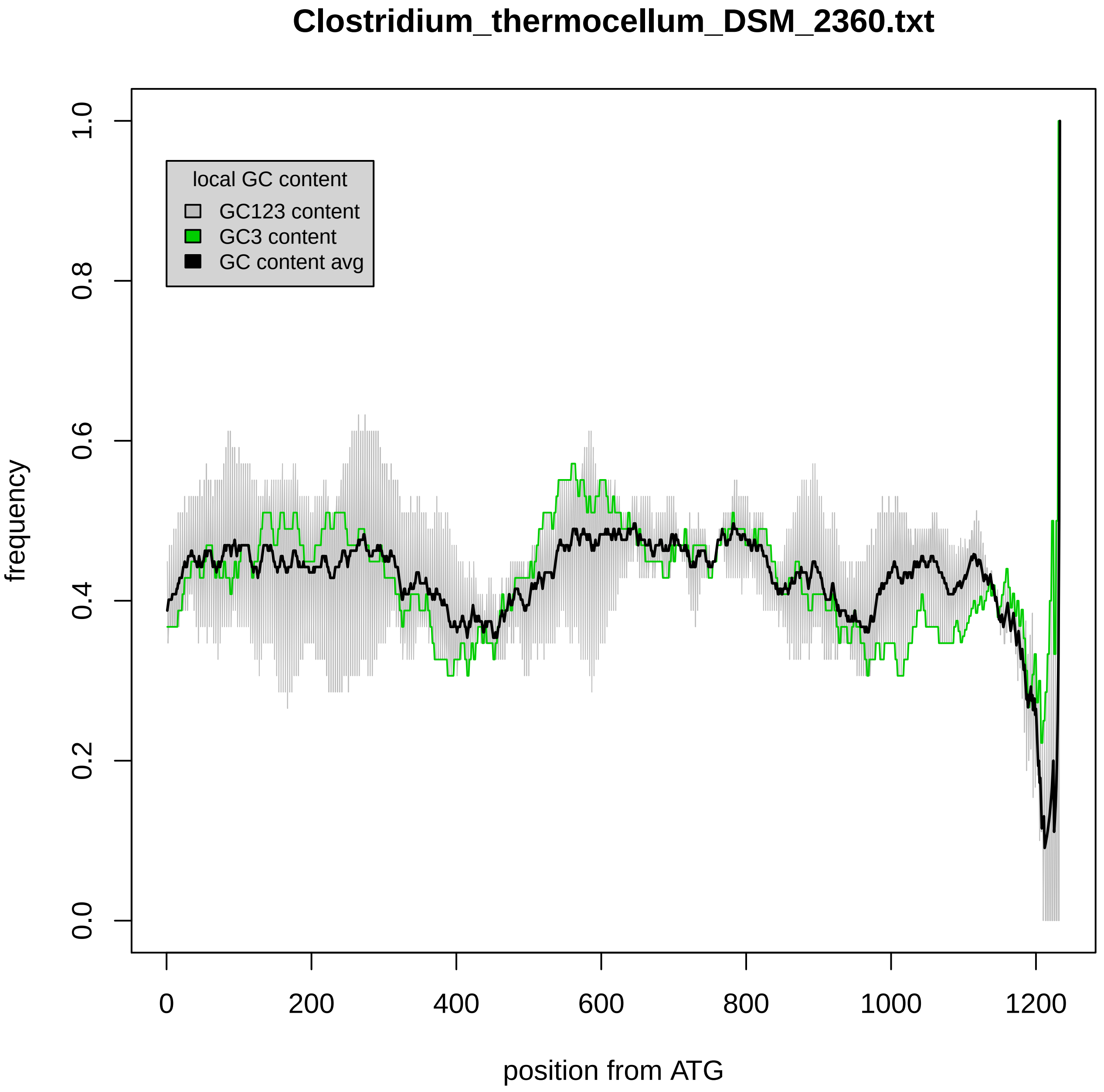

Clostridium_thermocellum_DSM_2360.txt
local GC content
GC123 content
GC3 content
GC content avg
frequency
position from ATG
0.0
0.2
0.4
0.6
0.8
1.0
0
200
400
600
800
1000
1200

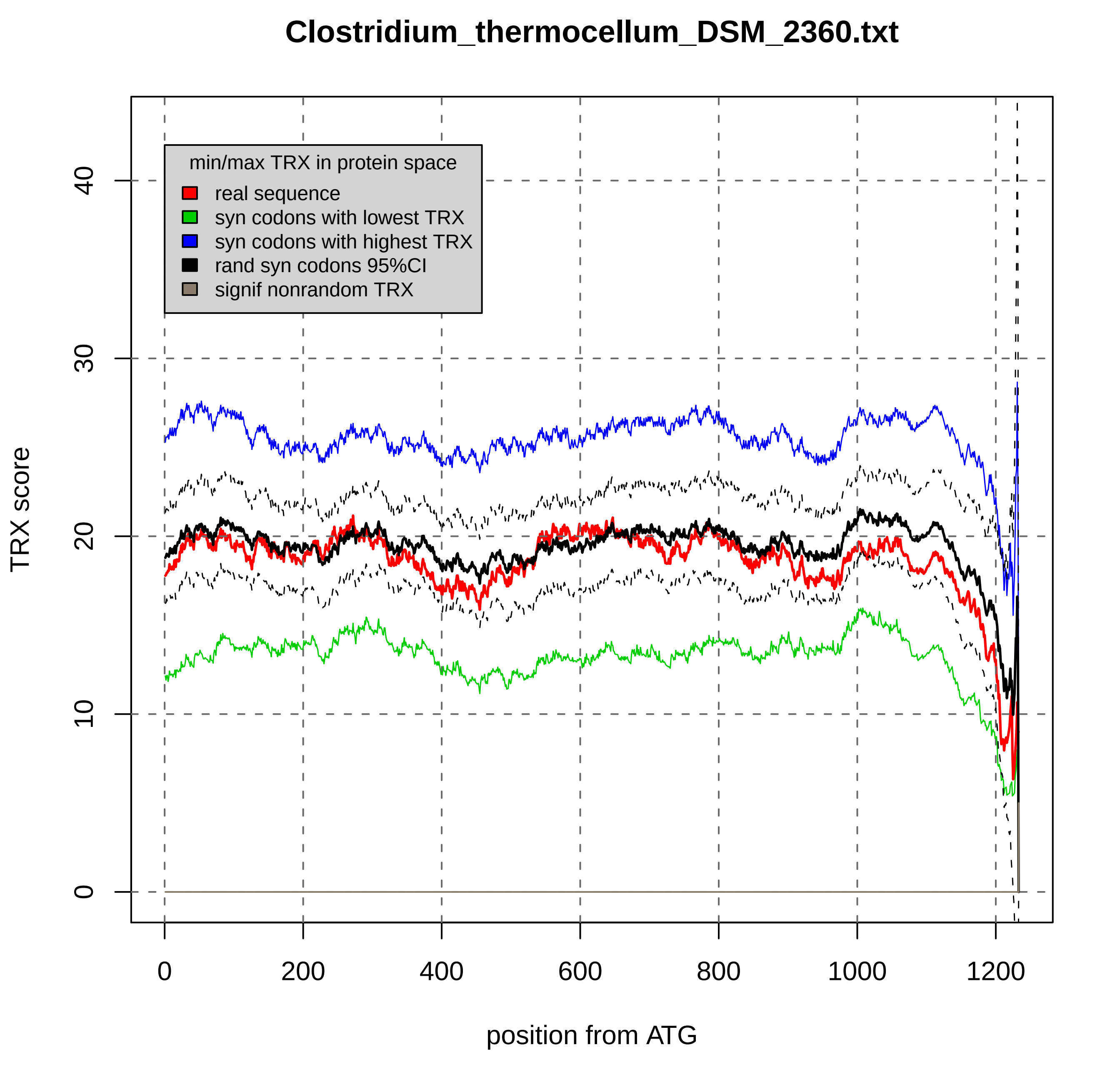

Clostridium_thermocellum_DSM_2360.txt
min/max TRX in protein space
real sequence
syn codons with lowest TRX
syn codons with highest TRX
rand syn codons 95%CI
signif nonrandom TRX
TRX score
0
10
20
30
40
0
200
400
600
800
1000
1200
position from ATG

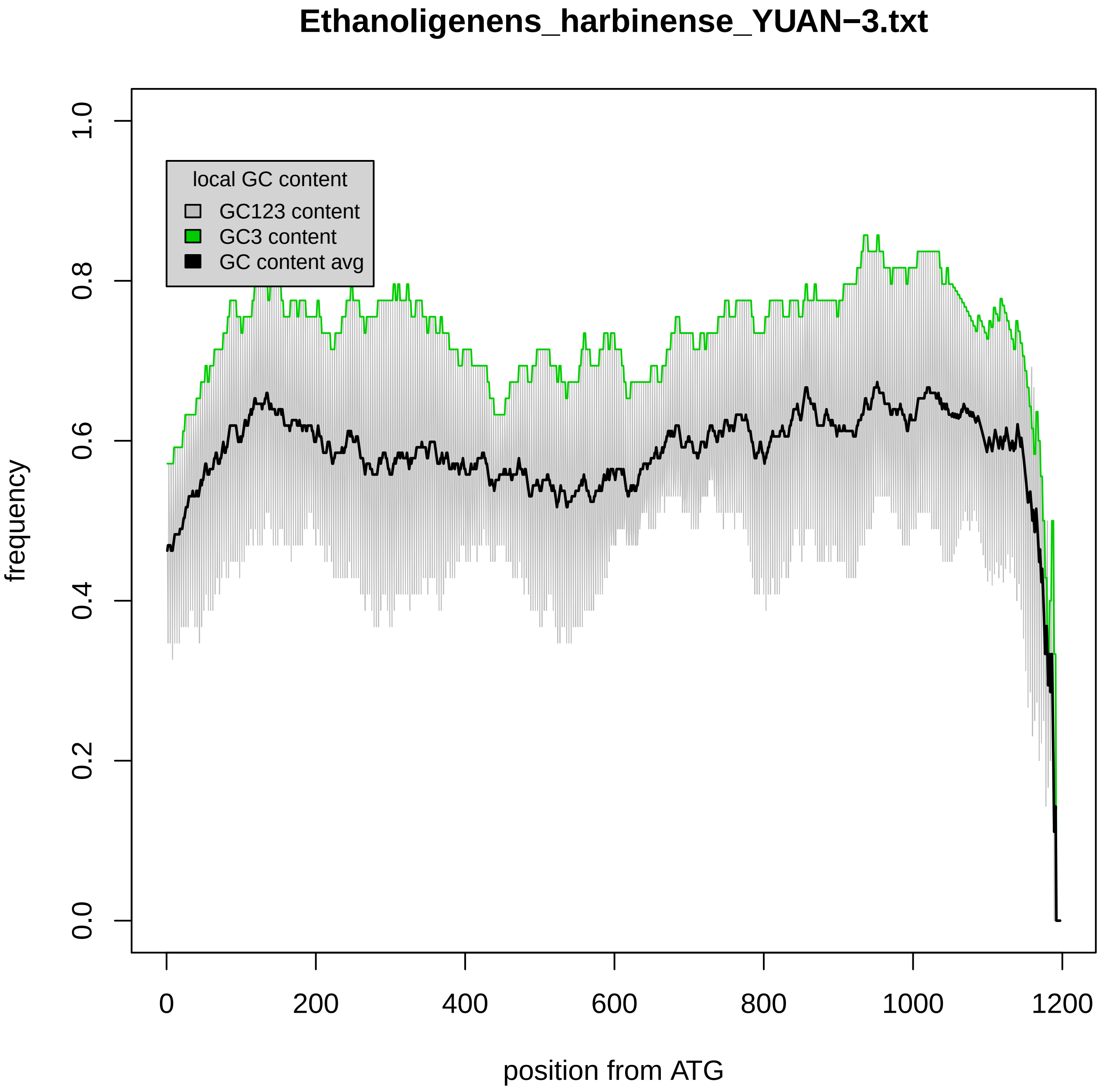
Ethanoligenens_harbinense_YUAN−3.txt
local GC content
GC123 content
GC3 content
GC content avg
frequency
position from ATG
0.0
0.2
0.4
0.6
0.8
1.0
0
200
400
600
800
1000
1200

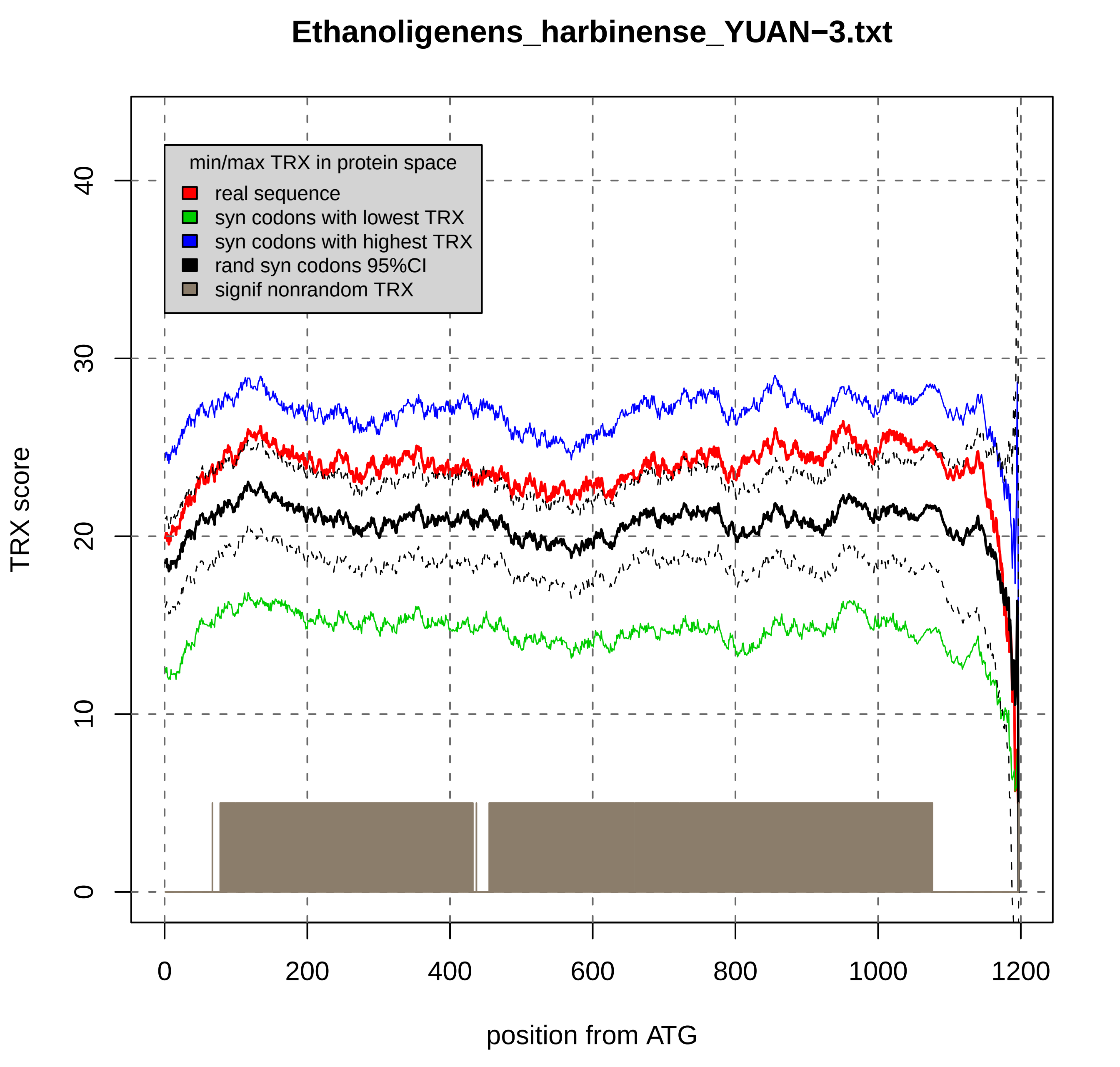
Ethanoligenens_harbinense_YUAN−3.txt
min/max TRX in protein space
real sequence
syn codons with lowest TRX
syn codons with highest TRX
rand syn codons 95%CI
signif nonrandom TRX
TRX score
0
10
20
30
40
0
200
400
600
800
1000
1200
position from ATG

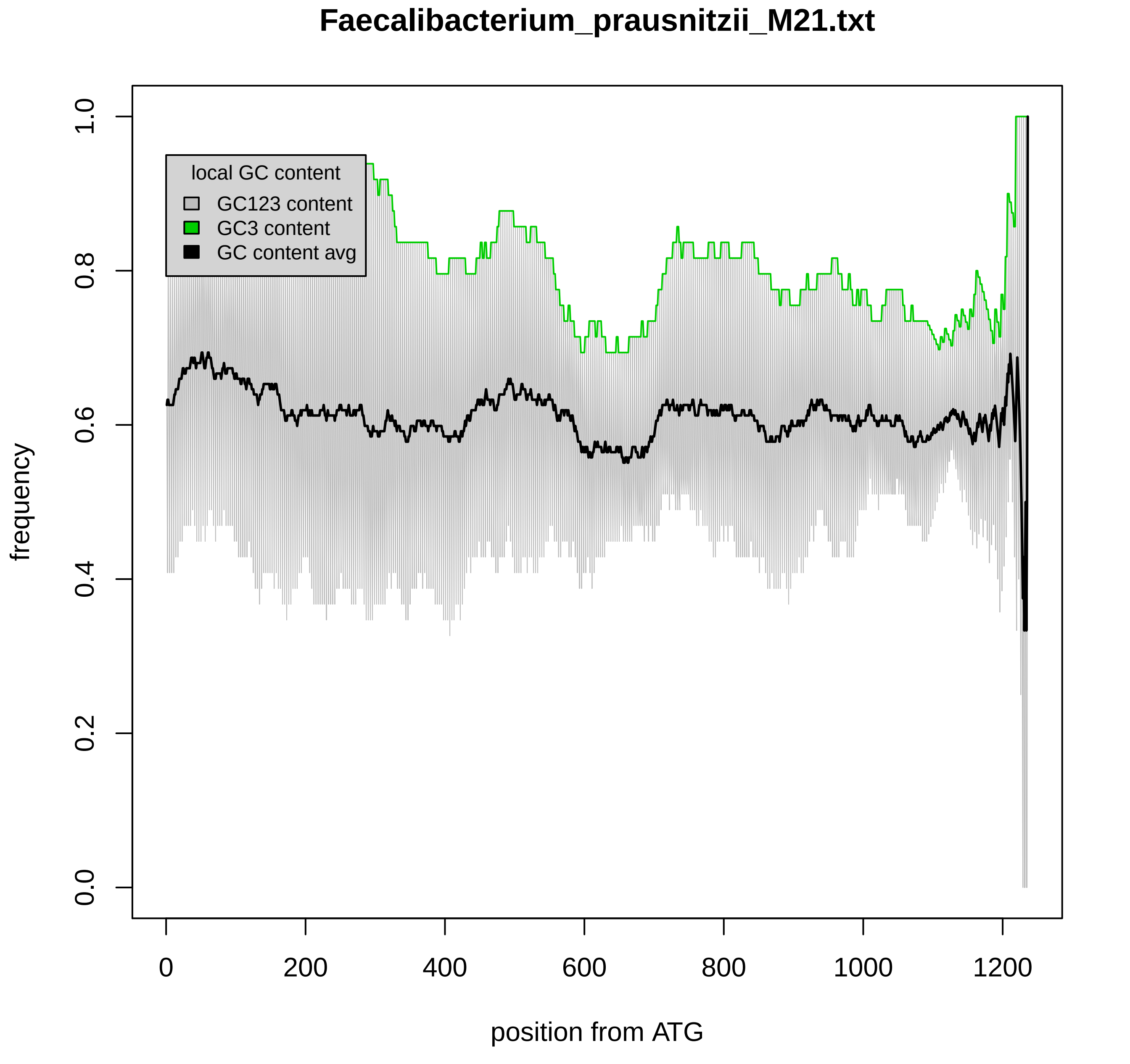
Faecalibacterium_prausnitzii_M21.txt
local GC content
GC123 content
GC3 content
GC content avg
frequency
position from ATG
0.0
0.2
0.4
0.6
0.8
1.0
0
200
400
600
800
1000
1200

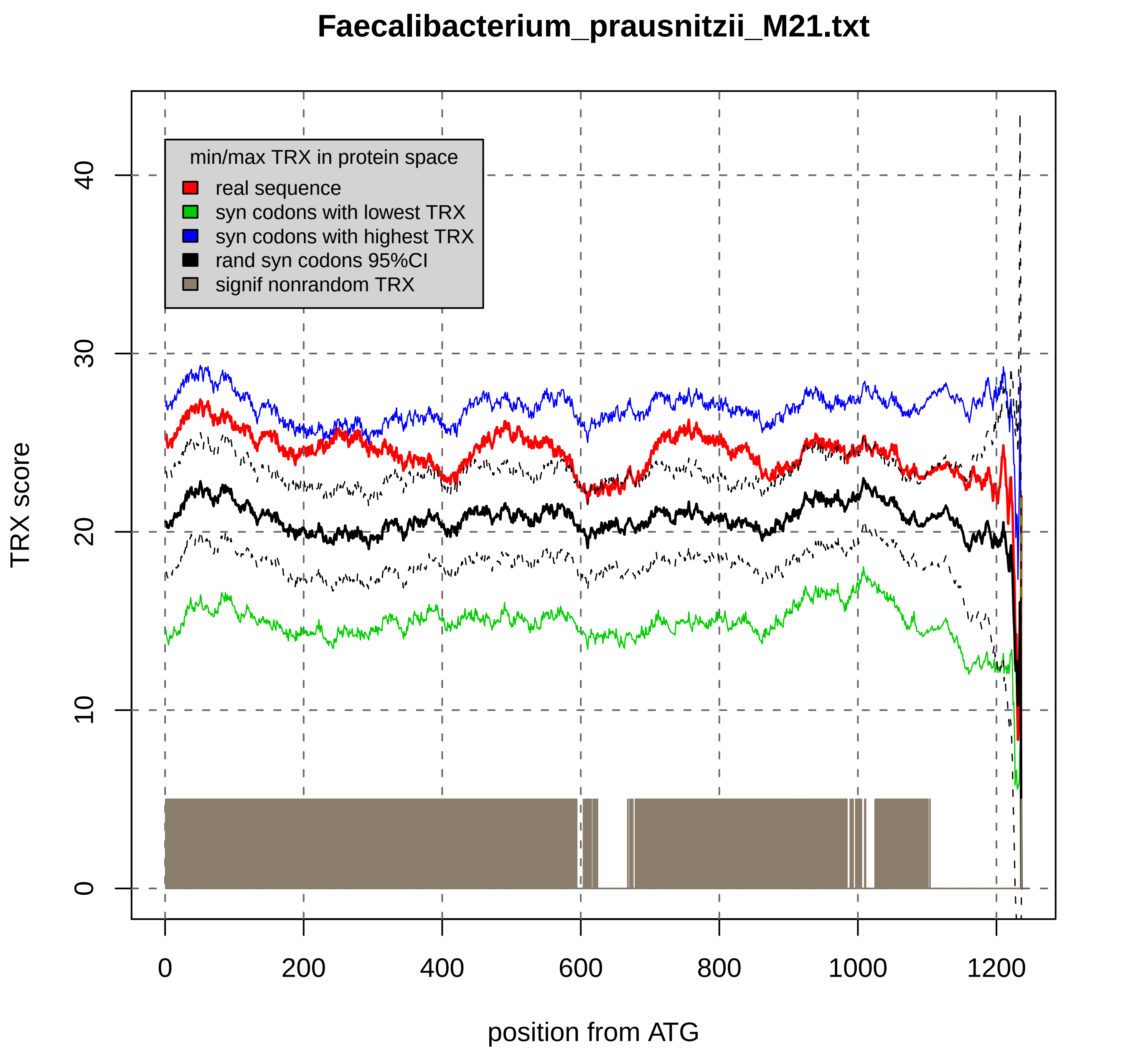
Faecalibacterium_prausnitzii_M21.txt
min/max TRX in protein space
real sequence
syn codons with lowest TRX
syn codons with highest TRX
rand syn codons 95%CI
signif nonrandom TRX
TRX score
position from ATG
0
10
20
30
40
0
200
400
600
800
1000
1200

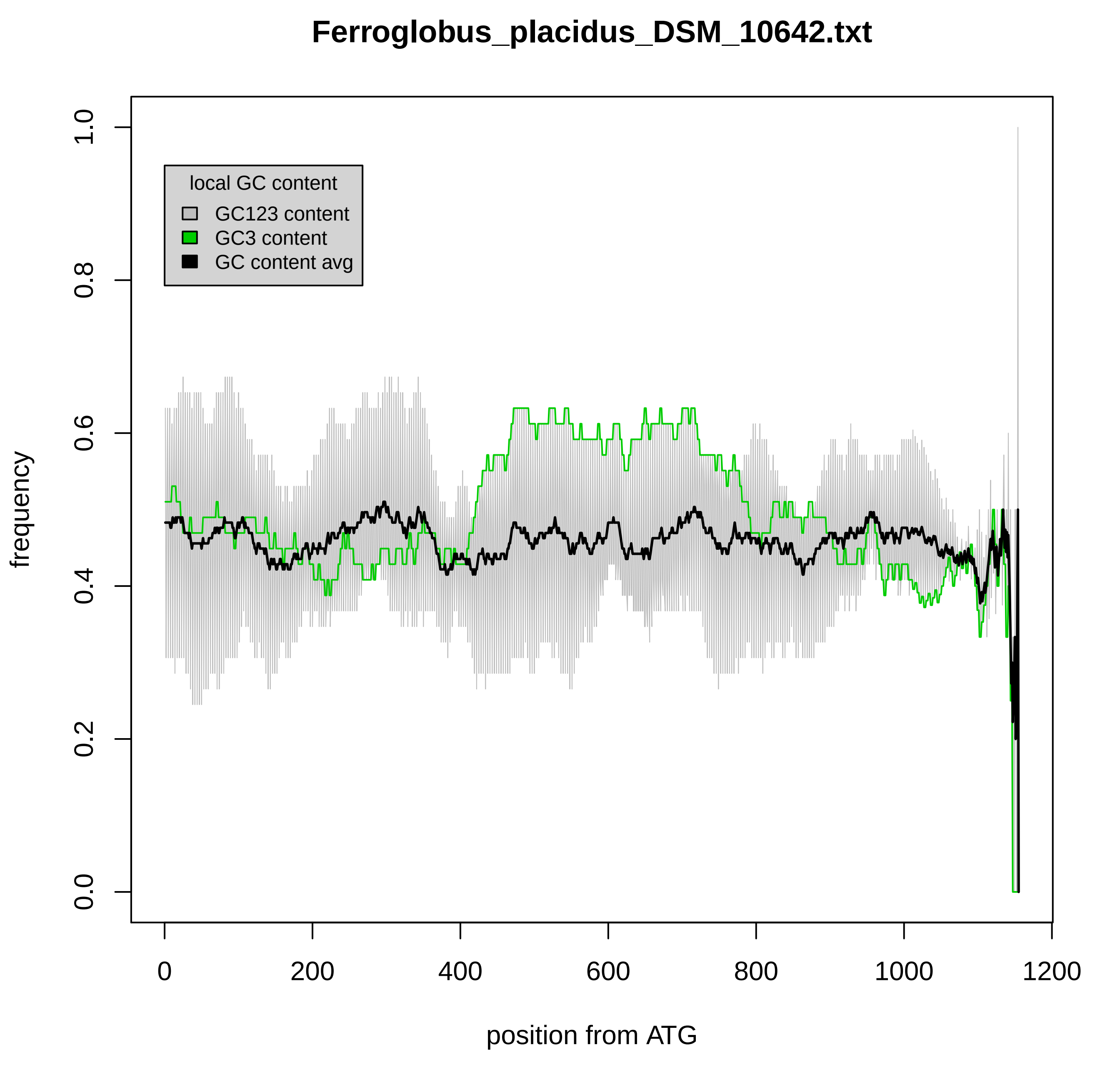
Ferroglobus_placidus_DSM_10642.txt
local GC content
GC123 content
GC3 content
GC content avg
frequency
position from ATG
0.0
0.2
0.4
0.6
0.8
1.0
0
200
400
600
800
1000
1200

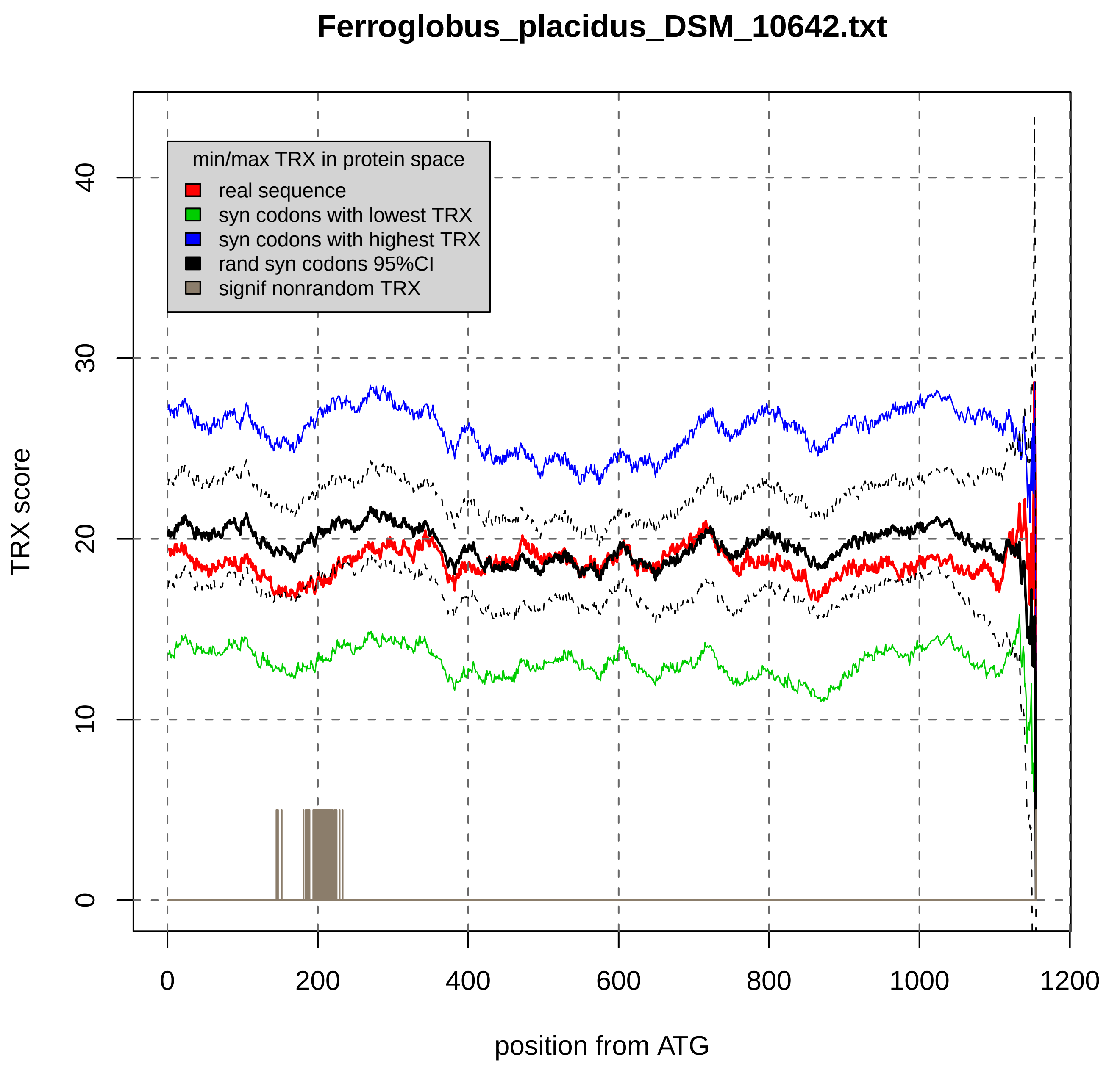
Ferroglobus_placidus_DSM_10642.txt
min/max TRX in protein space
real sequence
syn codons with lowest TRX
syn codons with highest TRX
rand syn codons 95%CI
signif nonrandom TRX
TRX score
position from ATG
0
10
20
30
40
0
200
400
600
800
1000
1200

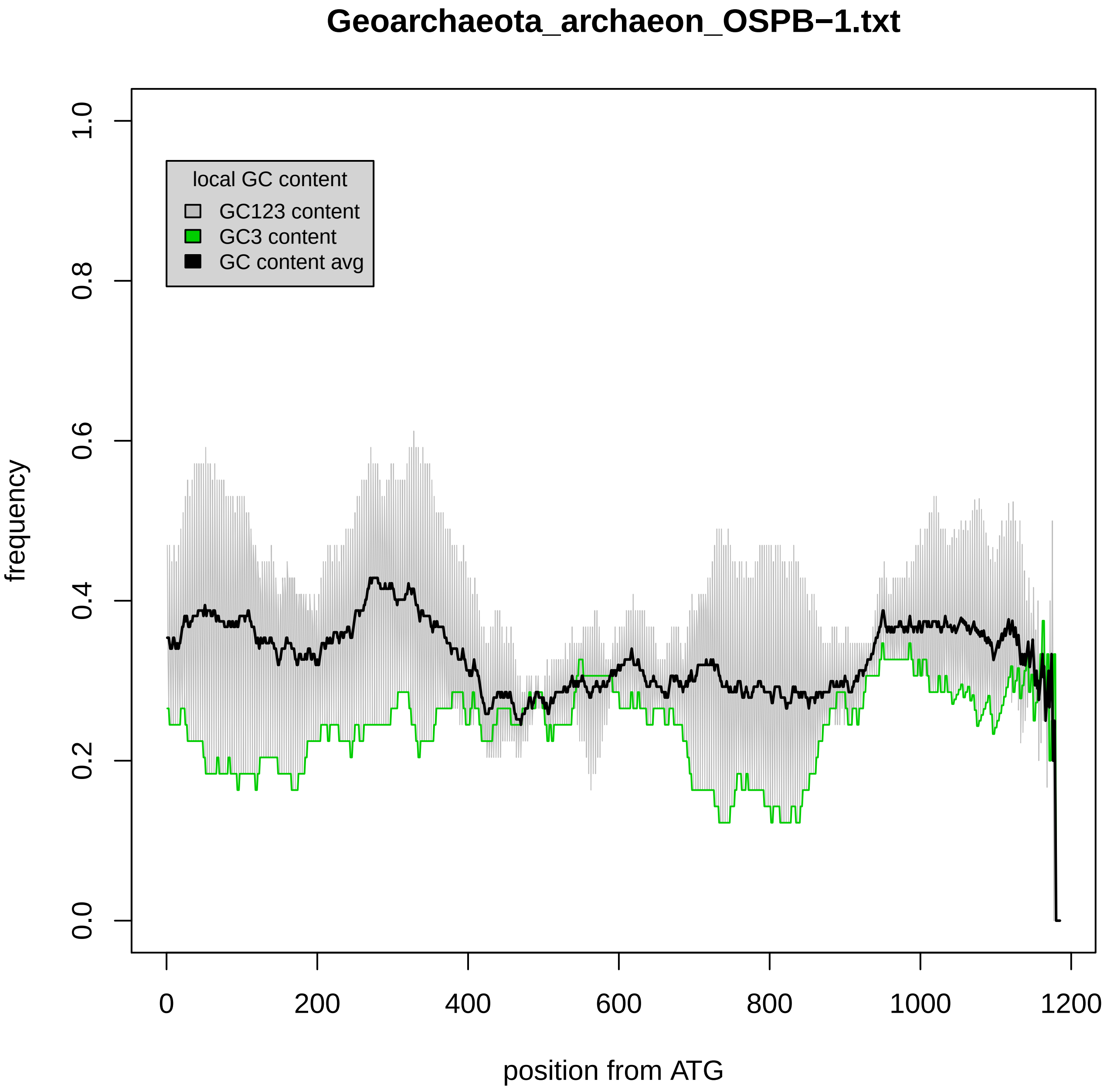
Geoarchaeota_archaeon_OSPB−1.txt
local GC content
GC123 content
GC3 content
GC content avg
frequency
0.0
0.2
0.4
0.6
0.8
1.0
position from ATG
0
200
400
600
800
1000
1200

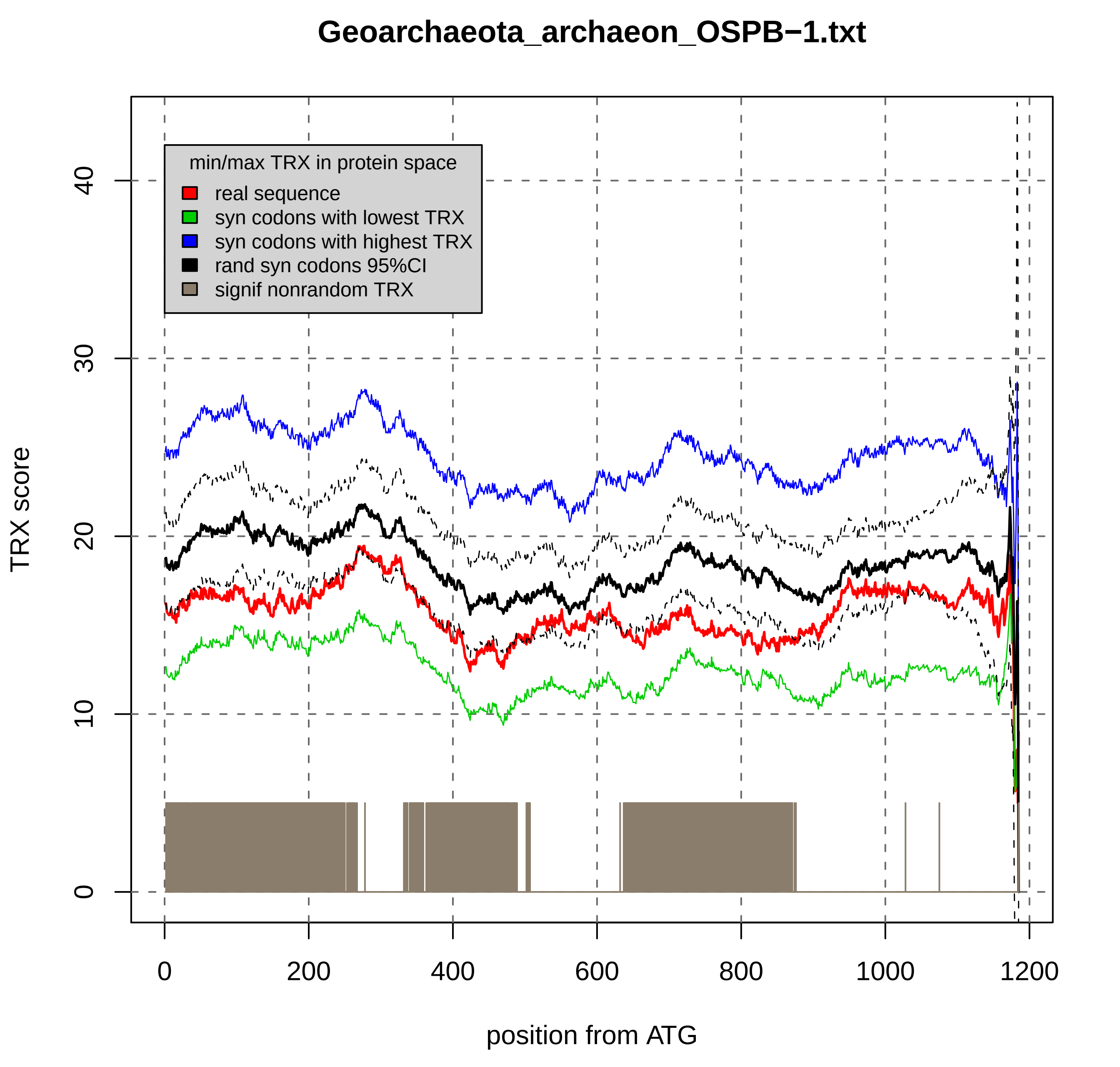
Geoarchaeota_archaeon_OSPB−1.txt
min/max TRX in protein space
real sequence
syn codons with lowest TRX
syn codons with highest TRX
rand syn codons 95%CI
signif nonrandom TRX
TRX score
position from ATG
0
10
20
30
40
0
200
400
600
800
1000
1200

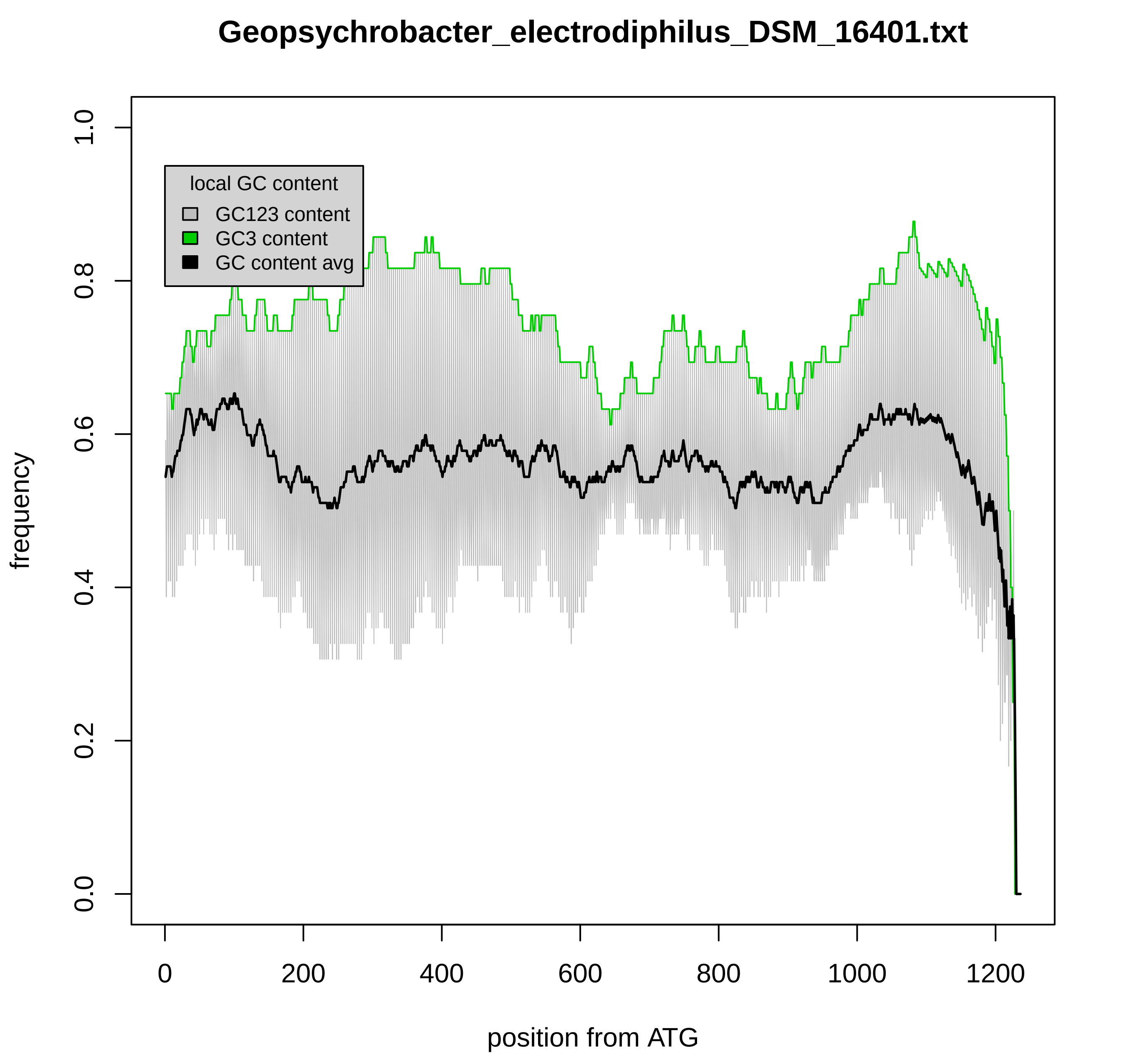

Geopsychrobacter_electrodiphilus_DSM_16401.txt
local GC content
GC123 content
GC3 content
GC content avg
frequency
position from ATG
0.0
0.2
0.4
0.6
0.8
1.0
0
200
400
600
800
1000
1200

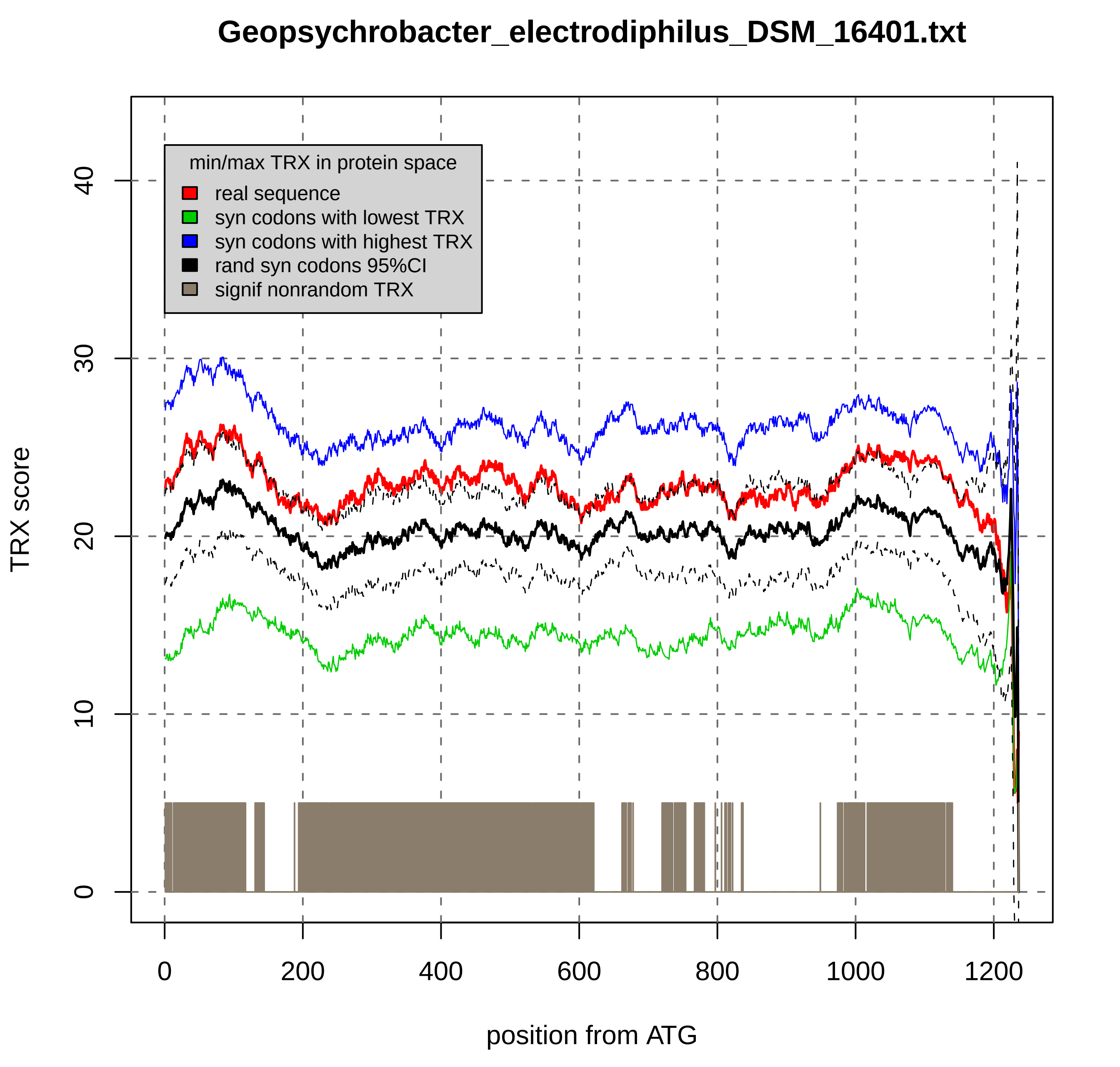

Geopsychrobacter_electrodiphilus_DSM_16401.txt
min/max TRX in protein space
real sequence
syn codons with lowest TRX
syn codons with highest TRX
rand syn codons 95%CI
signif nonrandom TRX
TRX score
0
10
20
30
40
0
200
400
600
800
1000
1200
position from ATG

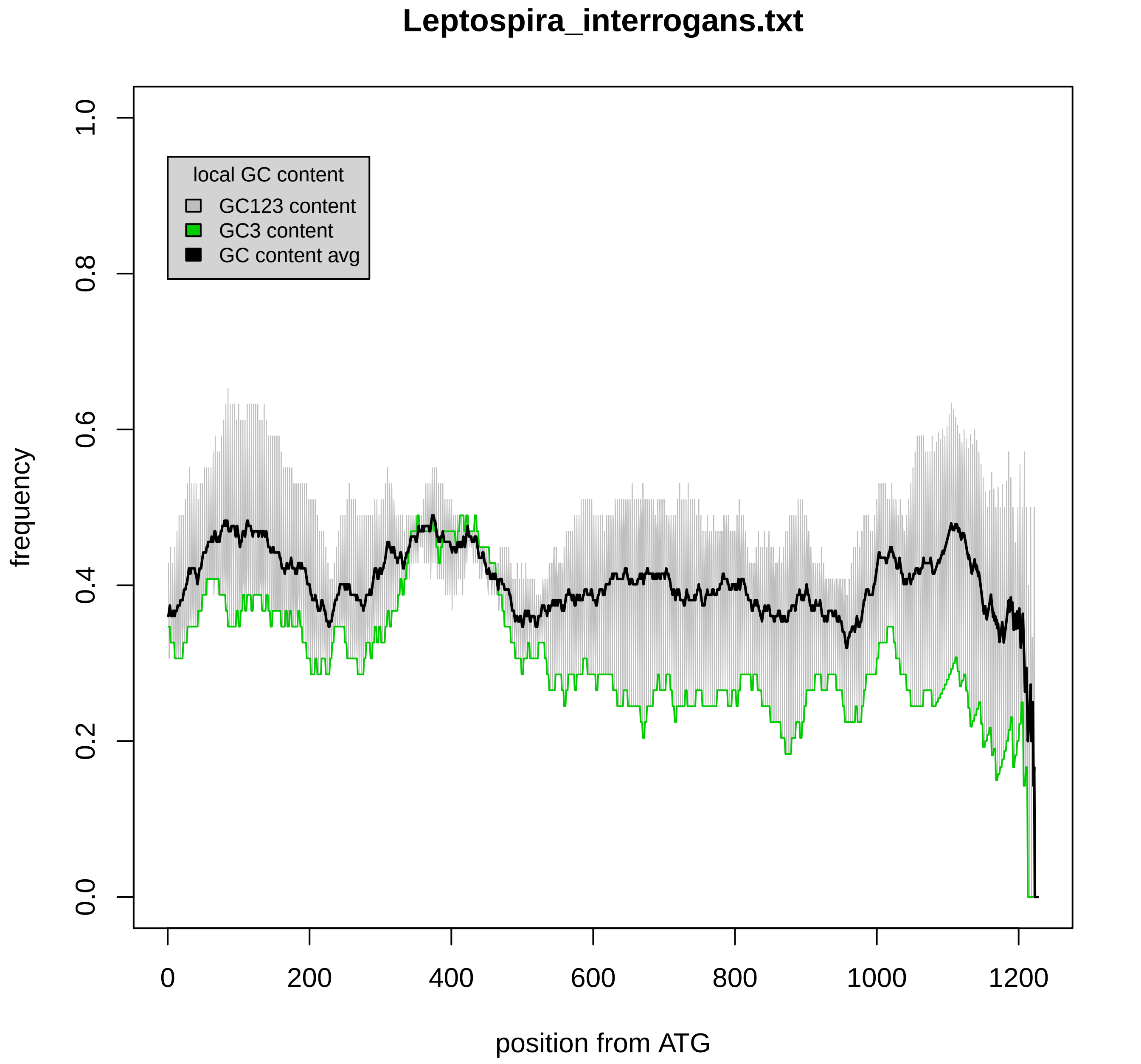
Leptospira_interrogans.txt
local GC content
GC123 content
GC3 content
GC content avg
frequency
0.0
0.2
0.4
0.6
0.8
1.0
0
200
400
600
800
1000
1200
position from ATG

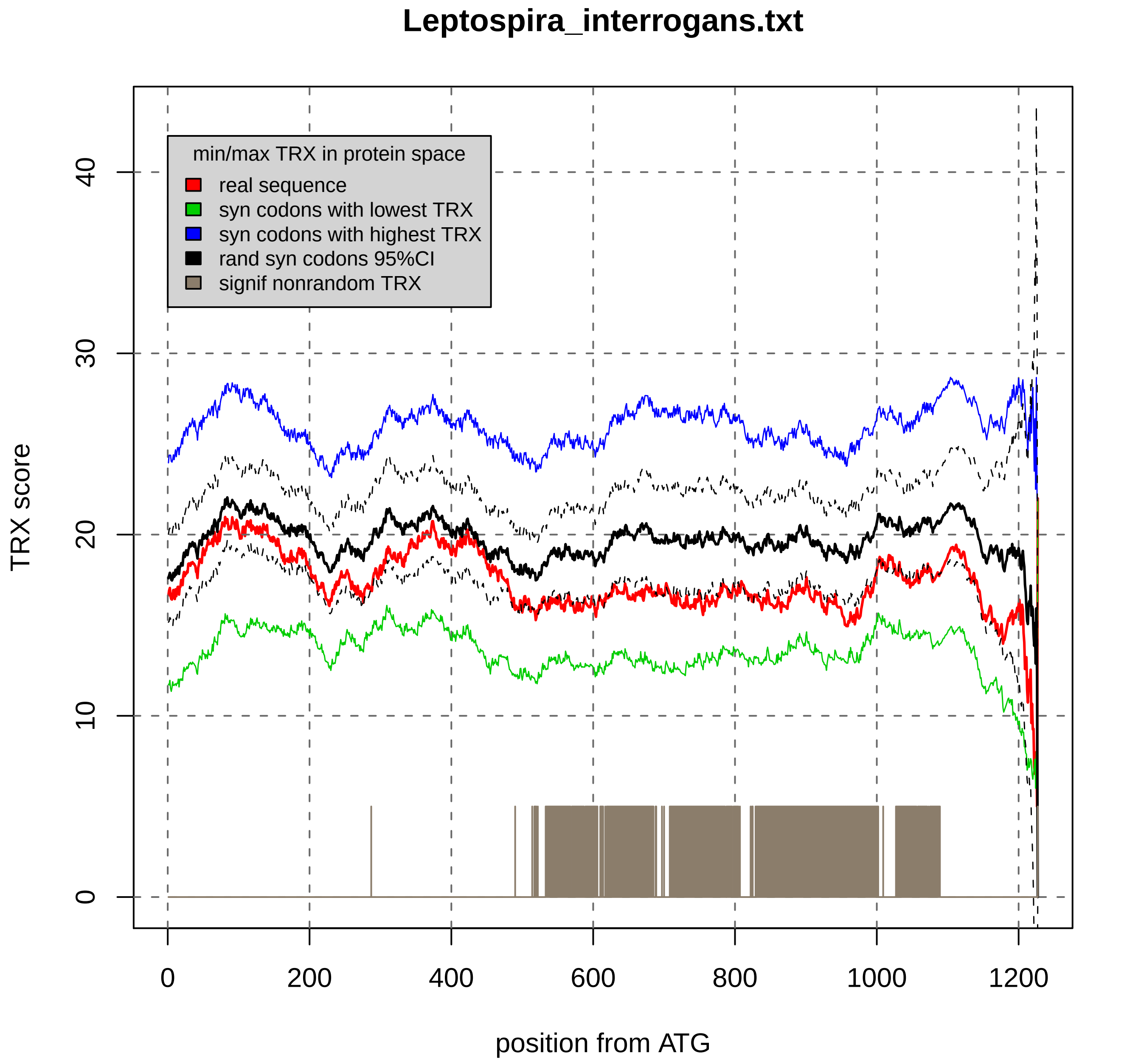
Leptospira_interrogans.txt
min/max TRX in protein space
real sequence
syn codons with lowest TRX
syn codons with highest TRX
rand syn codons 95%CI
signif nonrandom TRX
TRX score
position from ATG
0
10
20
30
40
0
200
400
600
800
1000
1200

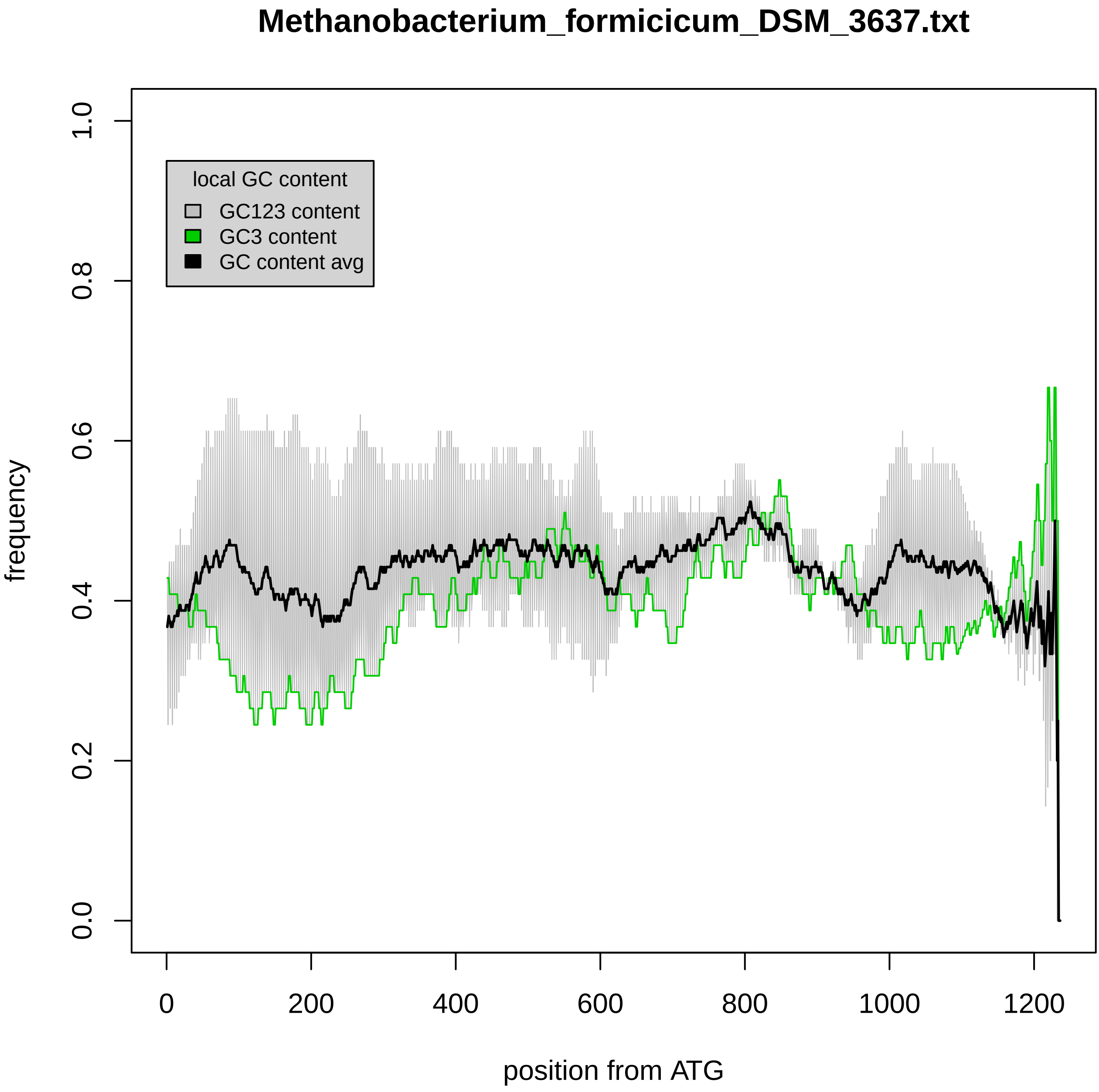

Methanobacterium_formicicum_DSM_3637.txt
local GC content
GC123 content
GC3 content
GC content avg
frequency
0.0
0.2
0.4
0.6
0.8
1.0
0
200
400
600
800
1000
1200
position from ATG

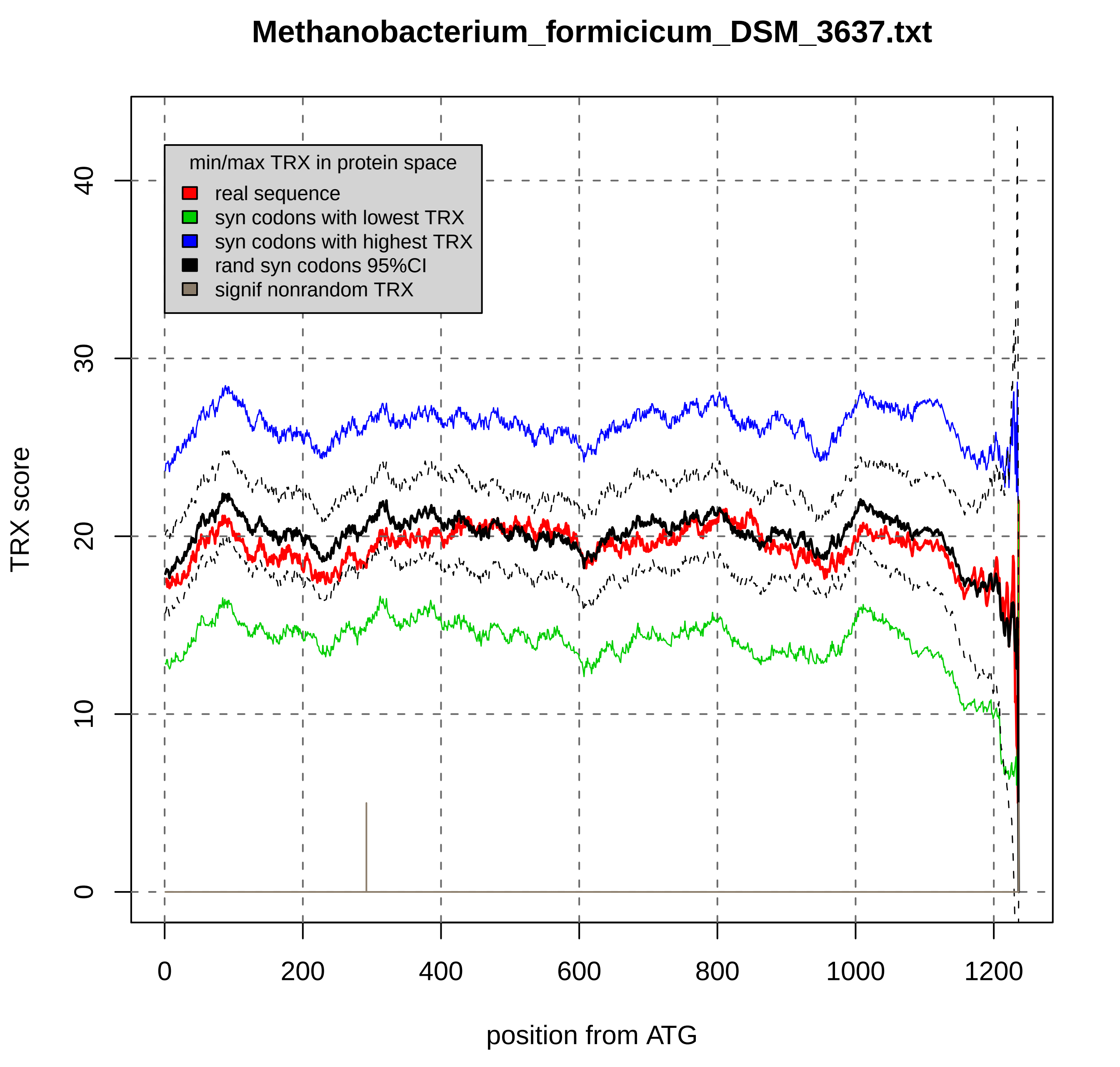

Methanobacterium_formicicum_DSM_3637.txt
min/max TRX in protein space
real sequence
syn codons with lowest TRX
syn codons with highest TRX
rand syn codons 95%CI
signif nonrandom TRX
TRX score
position from ATG
0
10
20
30
40
0
200
400
600
800
1000
1200

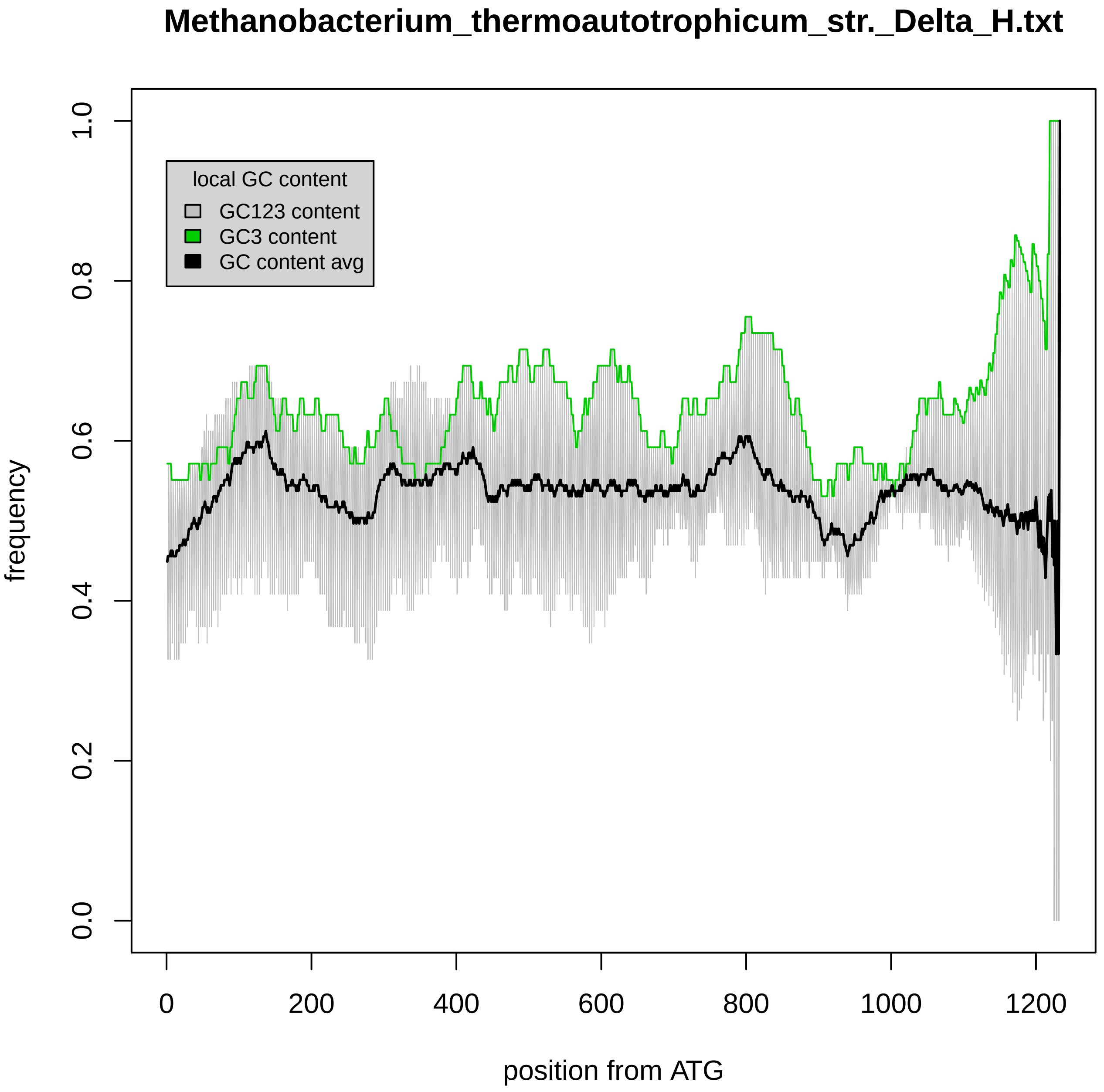

Methanobacterium_thermoautotrophicum_str._Delta_H.txt
local GC content
GC123 content
GC3 content
GC content avg
frequency
position from ATG
0.0
0.2
0.4
0.6
0.8
1.0
0
200
400
600
800
1000
1200

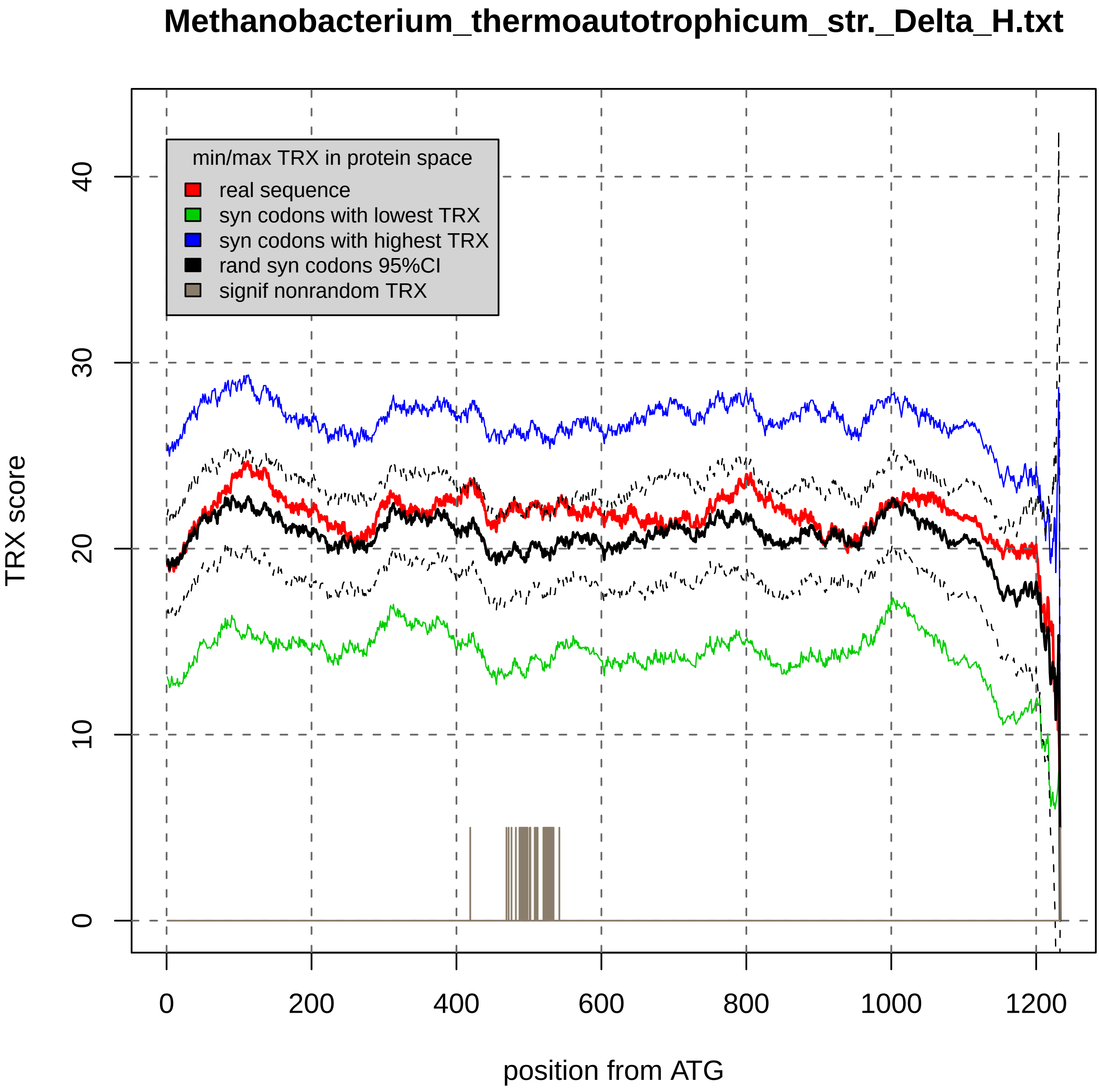
Methanobacterium_thermoautotrophicum_str._Delta_H.txt
min/max TRX in protein space
real sequence
syn codons with lowest TRX
syn codons with highest TRX
rand syn codons 95%CI
signif nonrandom TRX
TRX score
position from ATG
0
10
20
30
40
0
200
400
600
800
1000
1200

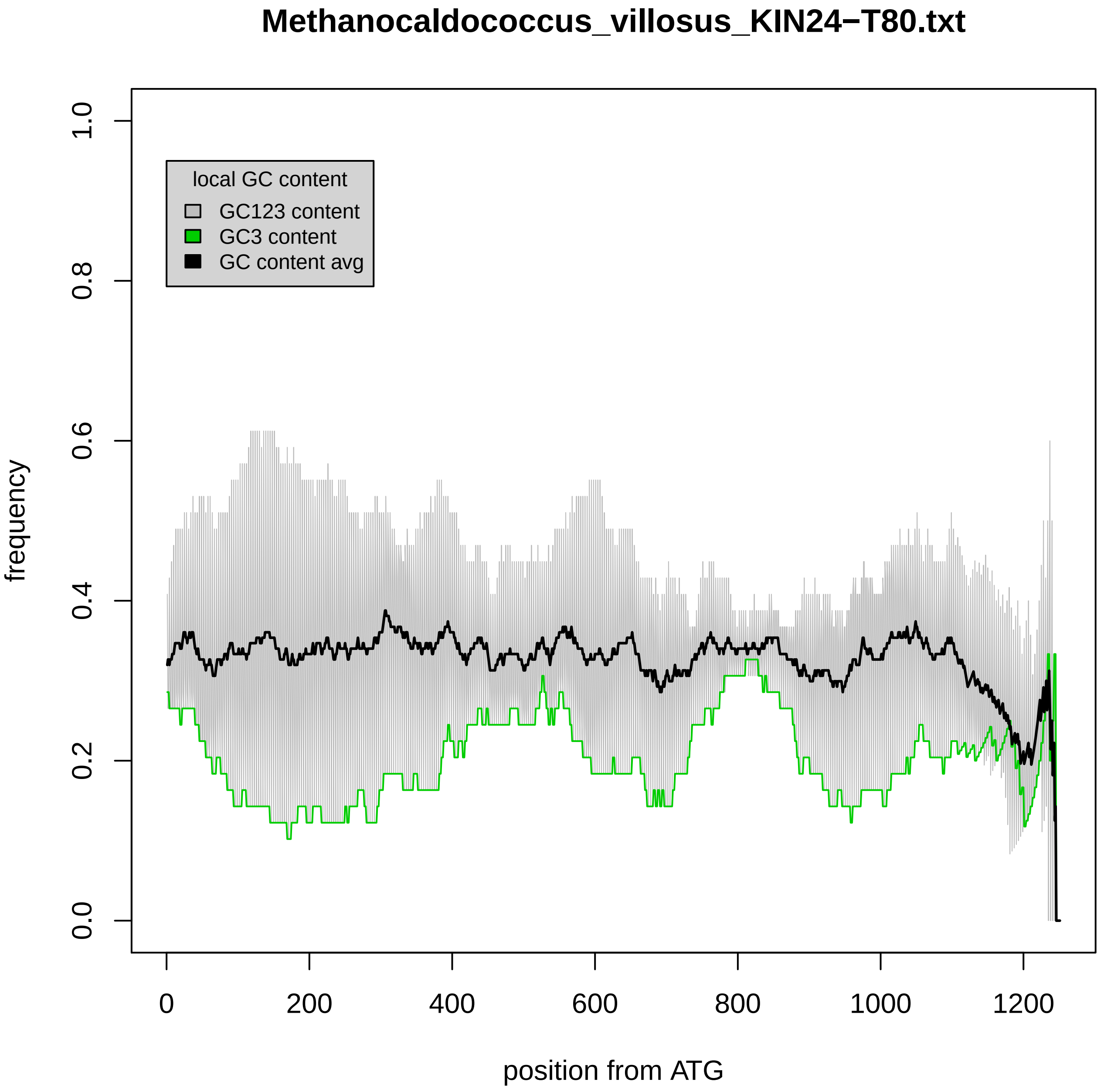

Methanocaldococcus_villosus_KIN24−T80.txt
local GC content
GC123 content
GC3 content
GC content avg
frequency
position from ATG
0.0
0.2
0.4
0.6
0.8
1.0
0
200
400
600
800
1000
1200

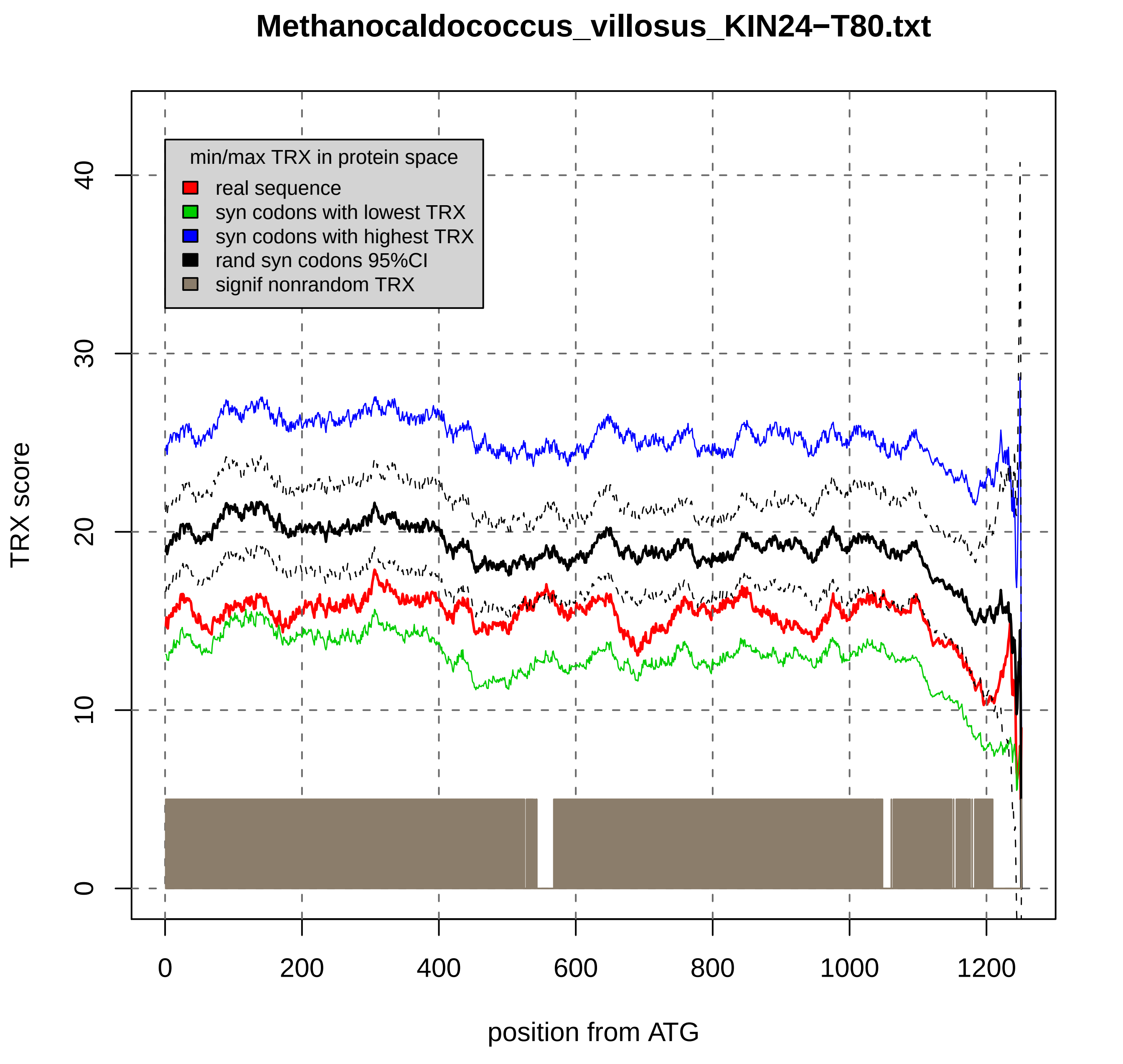

Methanocaldococcus_villosus_KIN24−T80.txt
min/max TRX in protein space
real sequence
syn codons with lowest TRX
syn codons with highest TRX
rand syn codons 95%CI
signif nonrandom TRX
TRX score
0
10
20
30
40
0
200
400
600
800
1000
1200
position from ATG

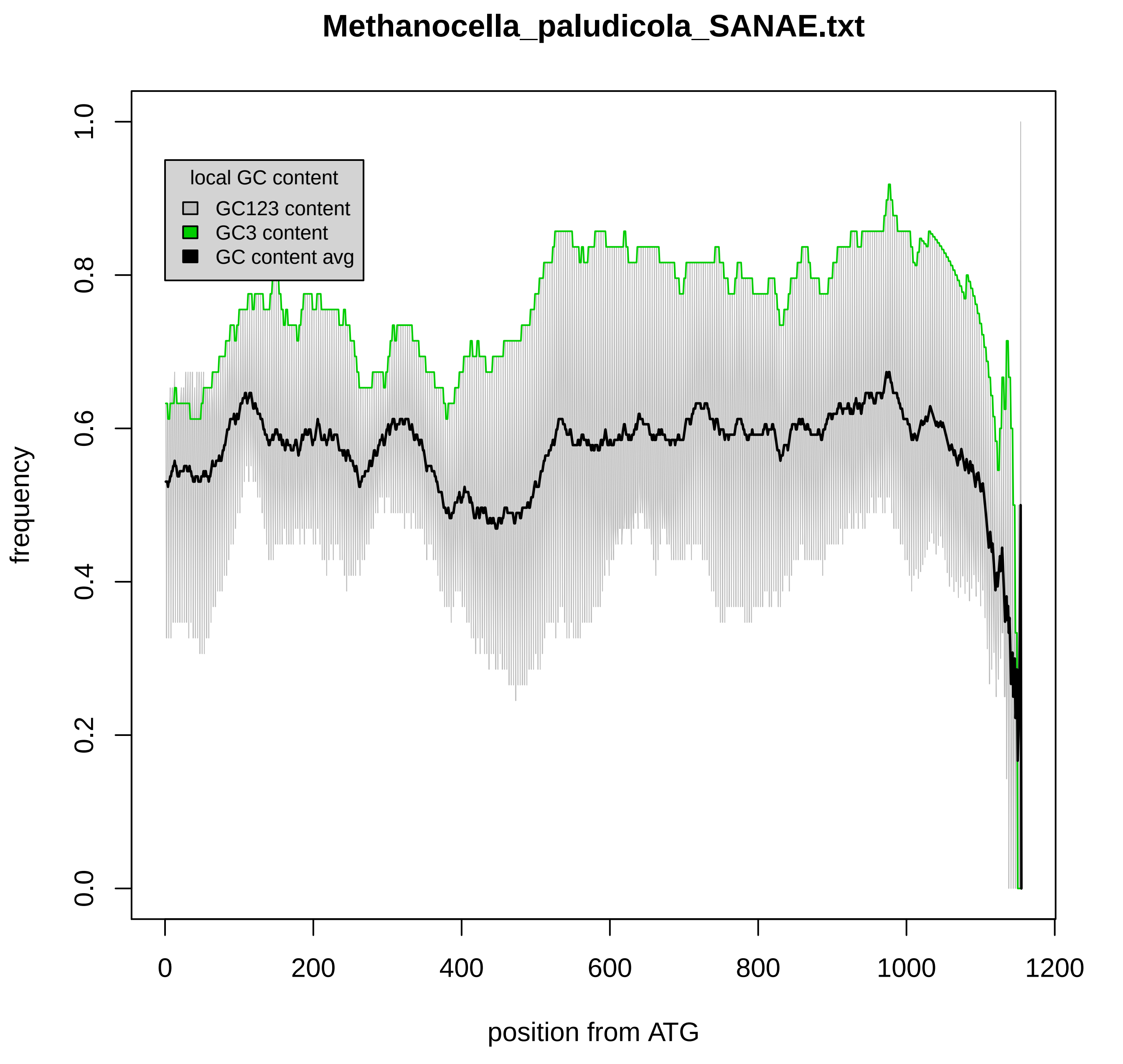

Methanocella_paludicola_SANAE.txt
local GC content
GC123 content
GC3 content
GC content avg
frequency
position from ATG
0.0
0.2
0.4
0.6
0.8
1.0
0
200
400
600
800
1000
1200

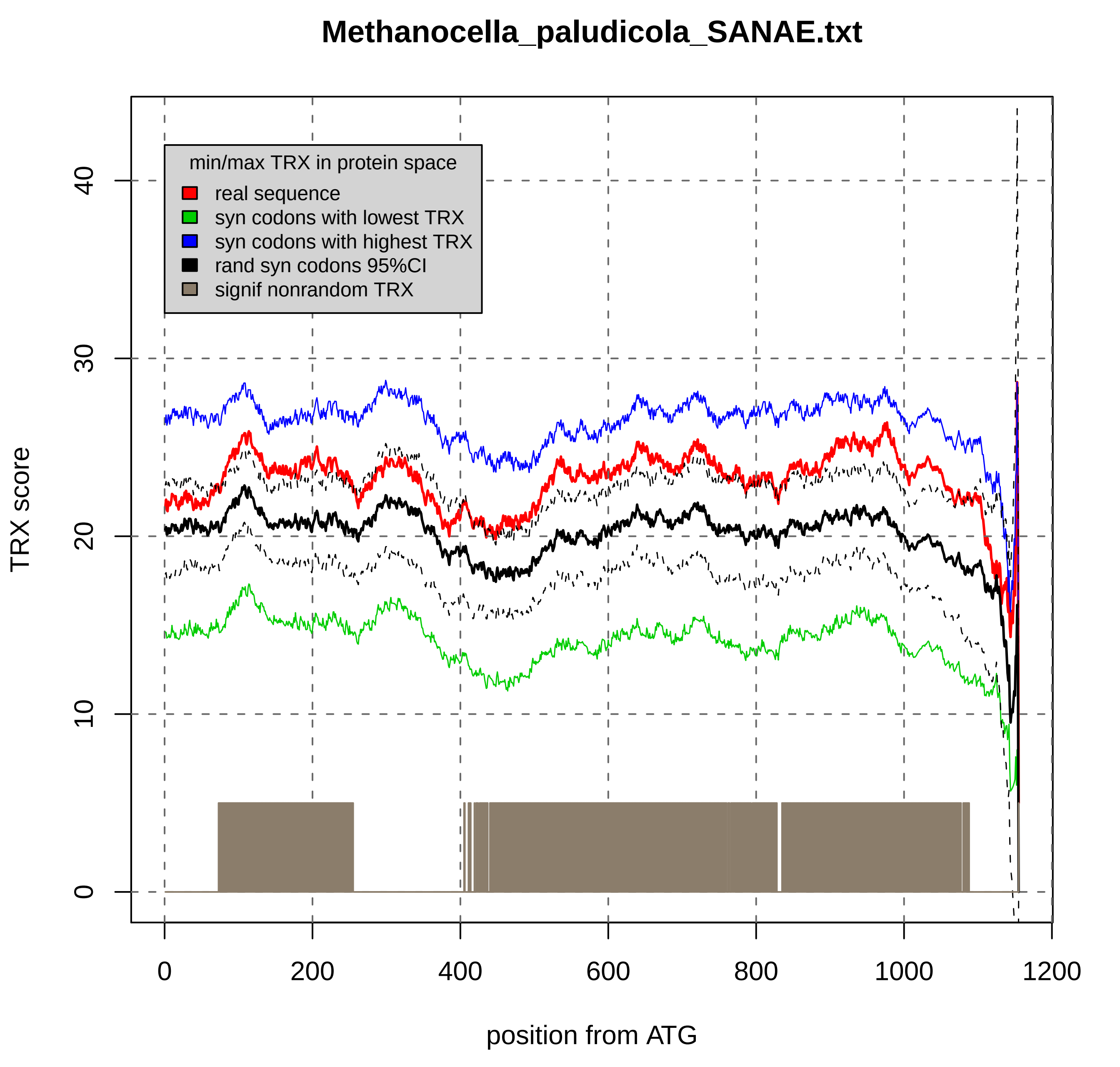
Methanocella_paludicola_SANAE.txt
min/max TRX in protein space
real sequence
syn codons with lowest TRX
syn codons with highest TRX
rand syn codons 95%CI
signif nonrandom TRX
TRX score
0
10
20
30
40
0
200
400
600
800
1000
1200
position from ATG

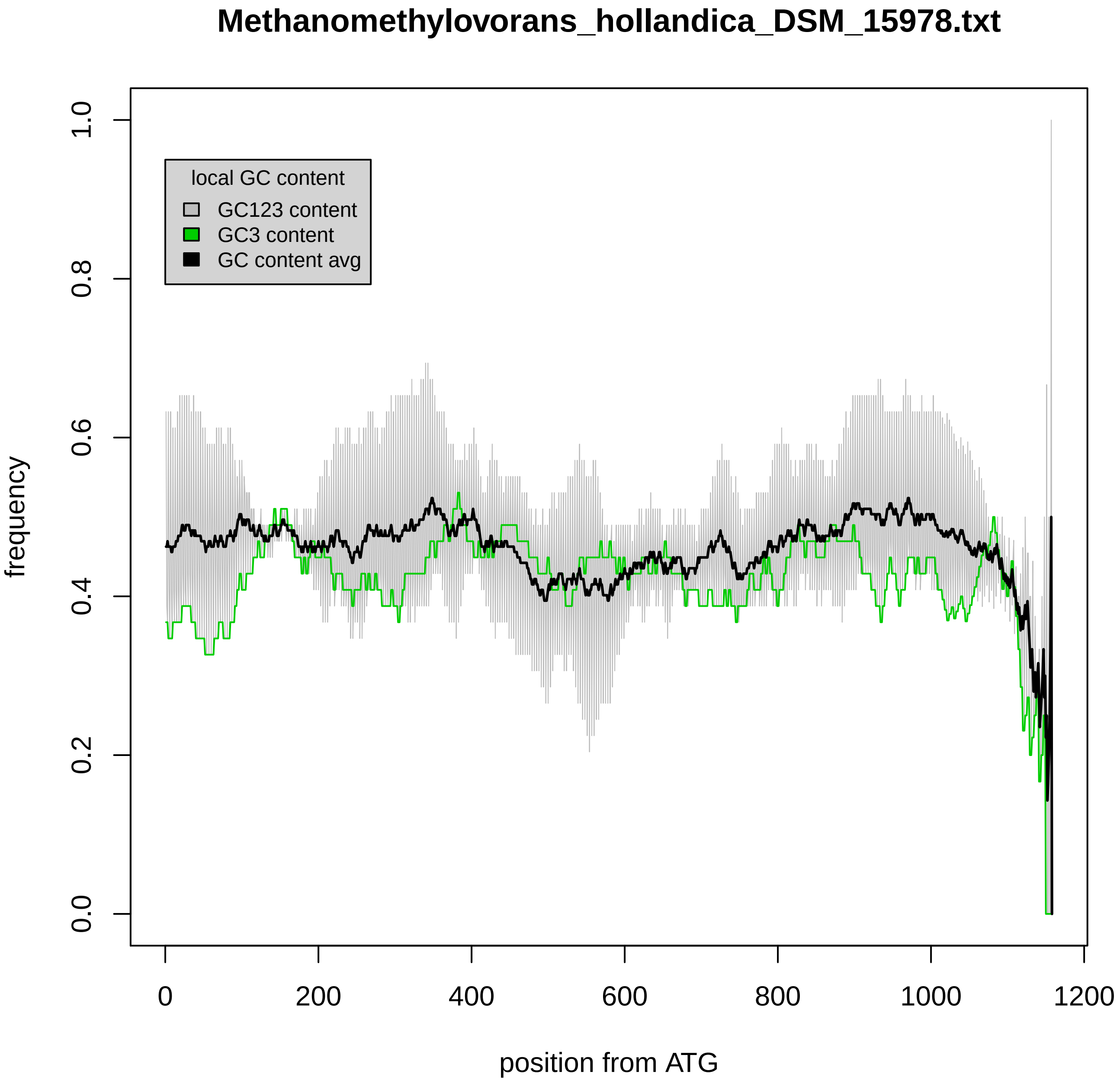
Methanomethylovorans_hollandica_DSM_15978.txt
local GC content
GC123 content
GC3 content
GC content avg
frequency
0.0
0.2
0.4
0.6
0.8
1.0
0
200
400
600
800
1000
1200
position from ATG

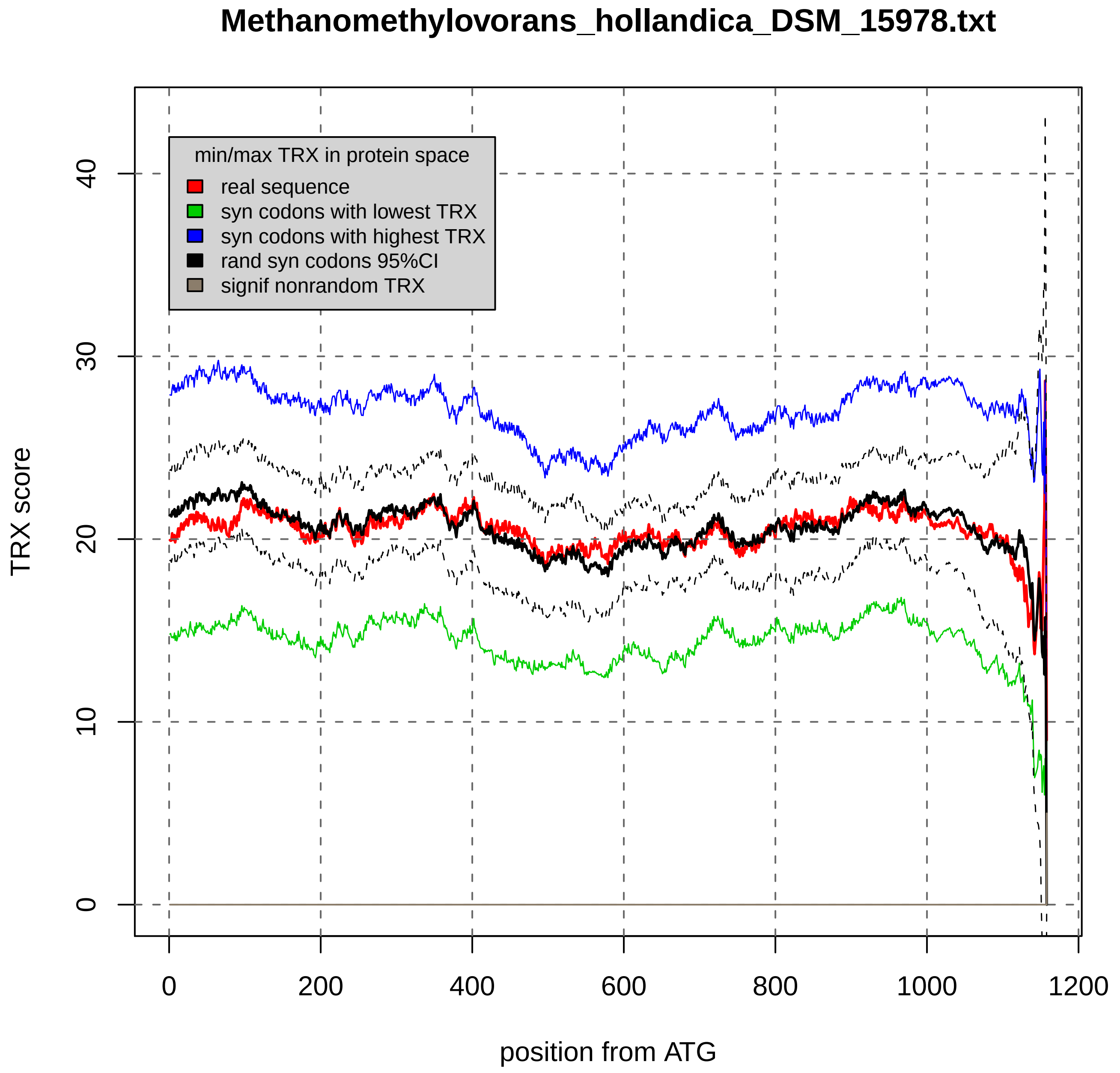

Methanomethylovorans_hollandica_DSM_15978.txt
min/max TRX in protein space
real sequence
syn codons with lowest TRX
syn codons with highest TRX
rand syn codons 95%CI
signif nonrandom TRX
TRX score
position from ATG
0
10
20
30
40
0
200
400
600
800
1000
1200

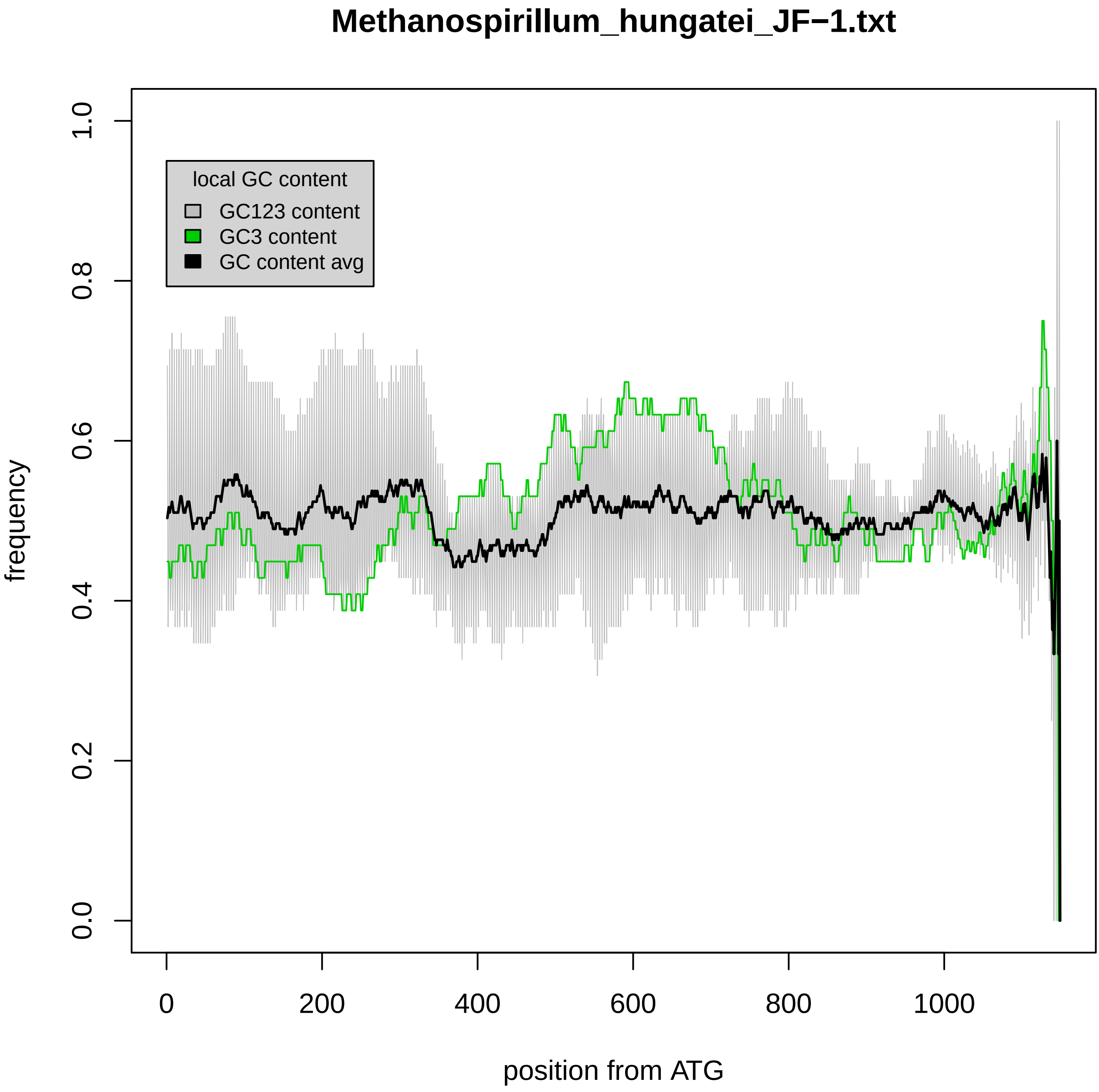
Methanospirillum_hungatei_JF−1.txt
local GC content
GC123 content
GC3 content
GC content avg
frequency
position from ATG
0.0
0.2
0.4
0.6
0.8
1.0
0
200
400
600
800
1000

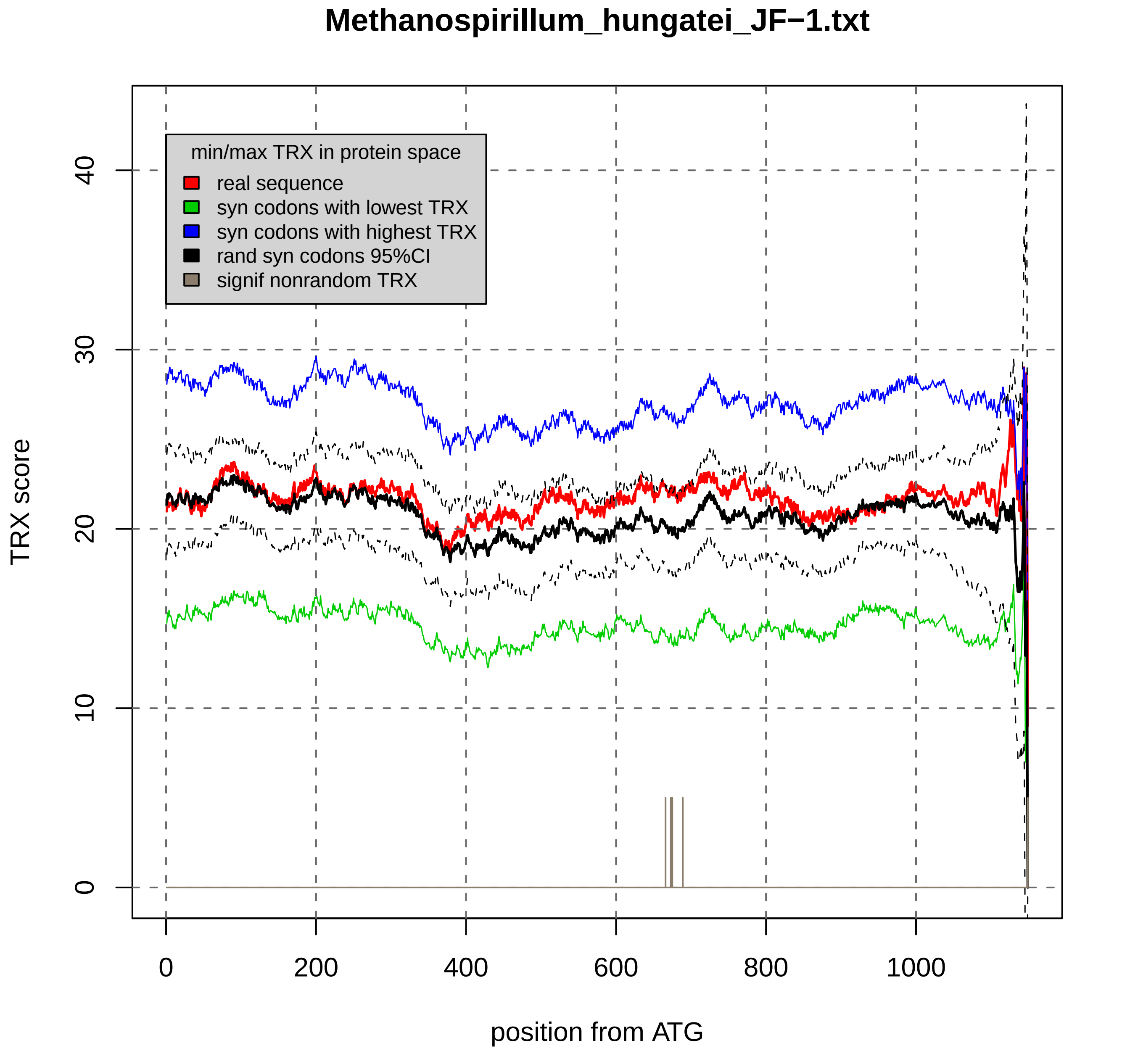
Methanospirillum_hungatei_JF−1.txt
min/max TRX in protein space
real sequence
syn codons with lowest TRX
syn codons with highest TRX
rand syn codons 95%CI
signif nonrandom TRX
TRX score
position from ATG
0
10
20
30
40
0
200
400
600
800
1000

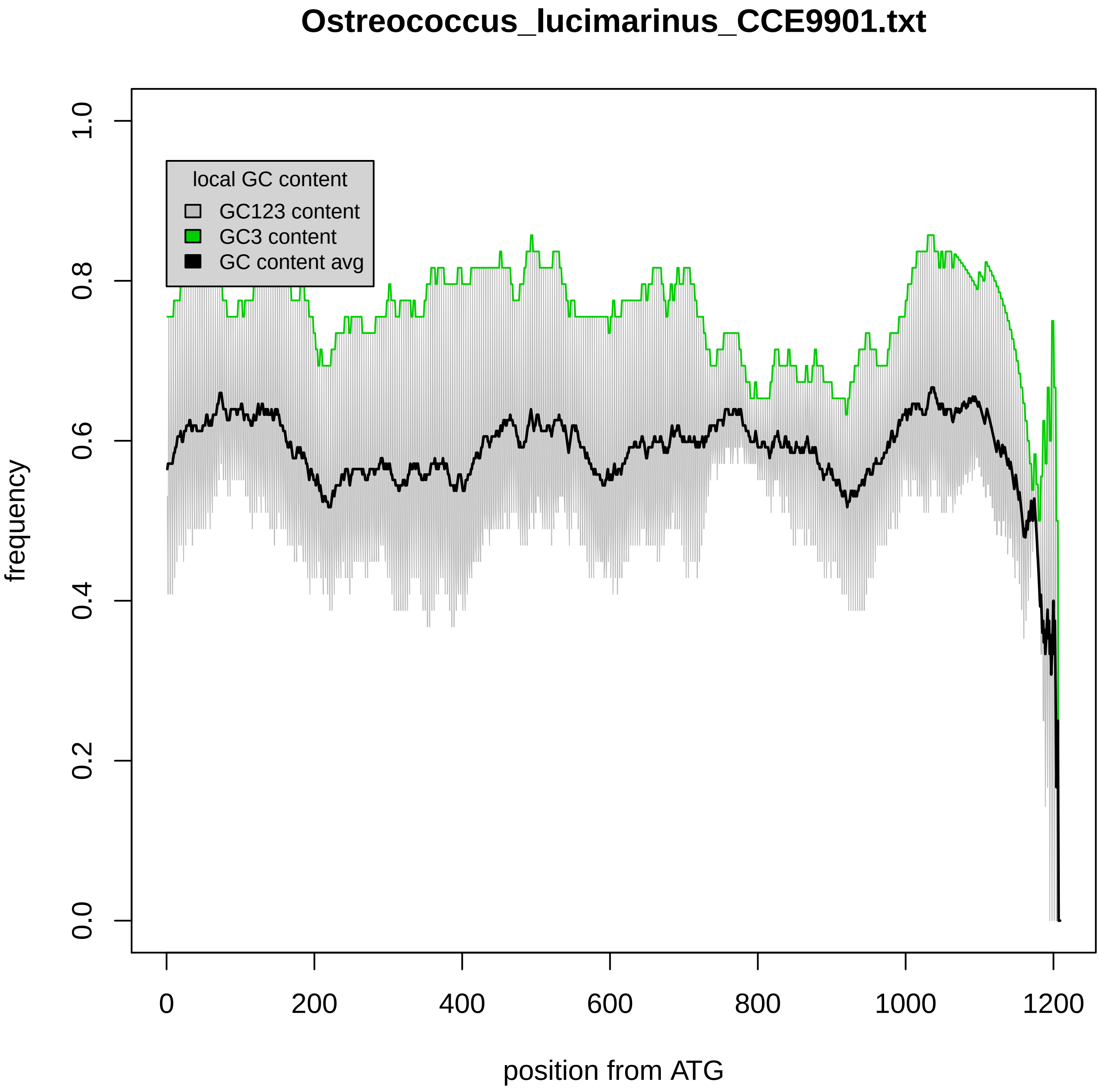

Ostreococcus_lucimarinus_CCE9901.txt
local GC content
GC123 content
GC3 content
GC content avg
frequency
position from ATG
0.0
0.2
0.4
0.6
0.8
1.0
0
200
400
600
800
1000
1200

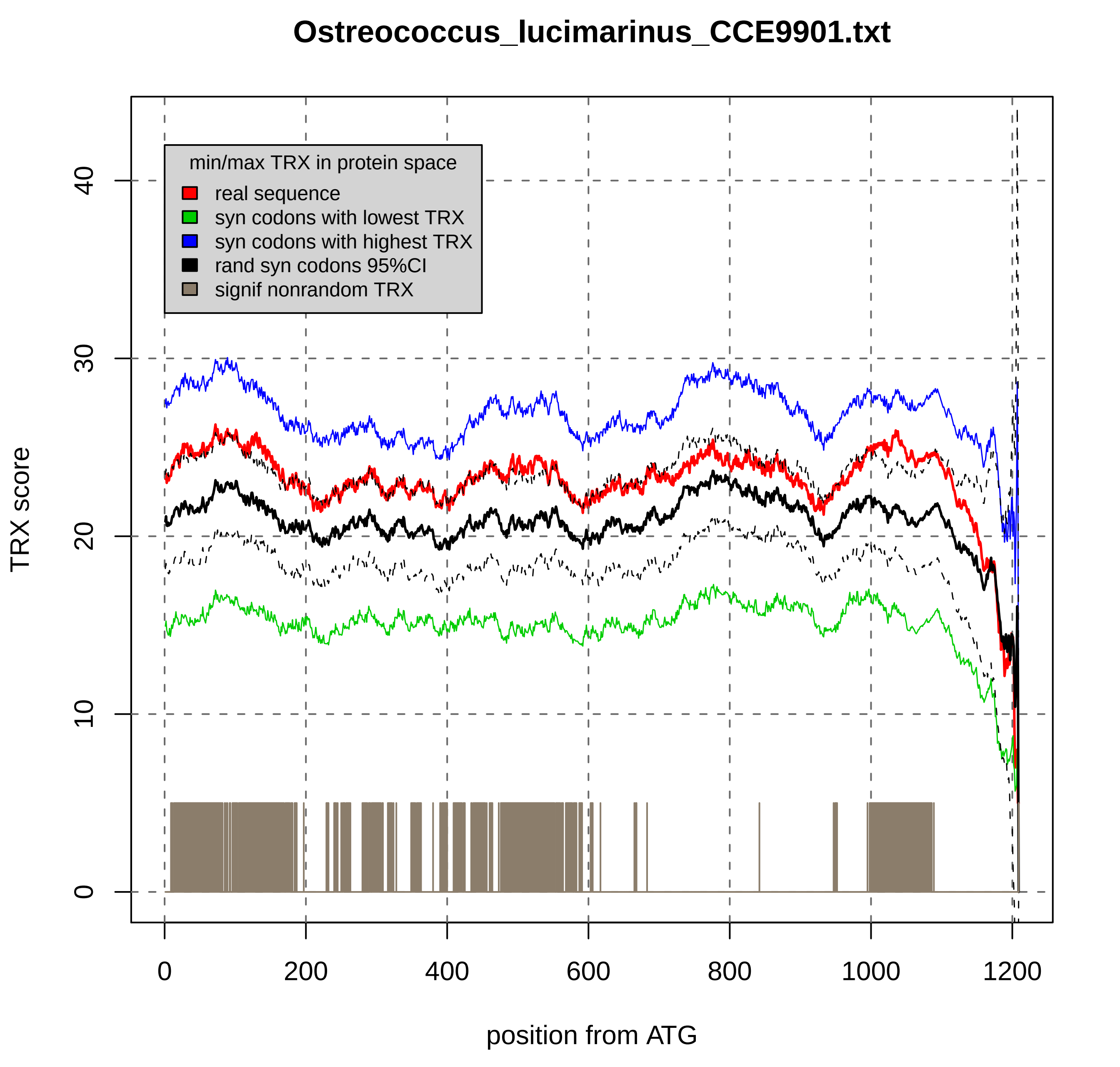

Ostreococcus_lucimarinus_CCE9901.txt
min/max TRX in protein space
real sequence
syn codons with lowest TRX
syn codons with highest TRX
rand syn codons 95%CI
signif nonrandom TRX
TRX score
0
10
20
30
40
0
200
400
600
800
1000
1200
position from ATG

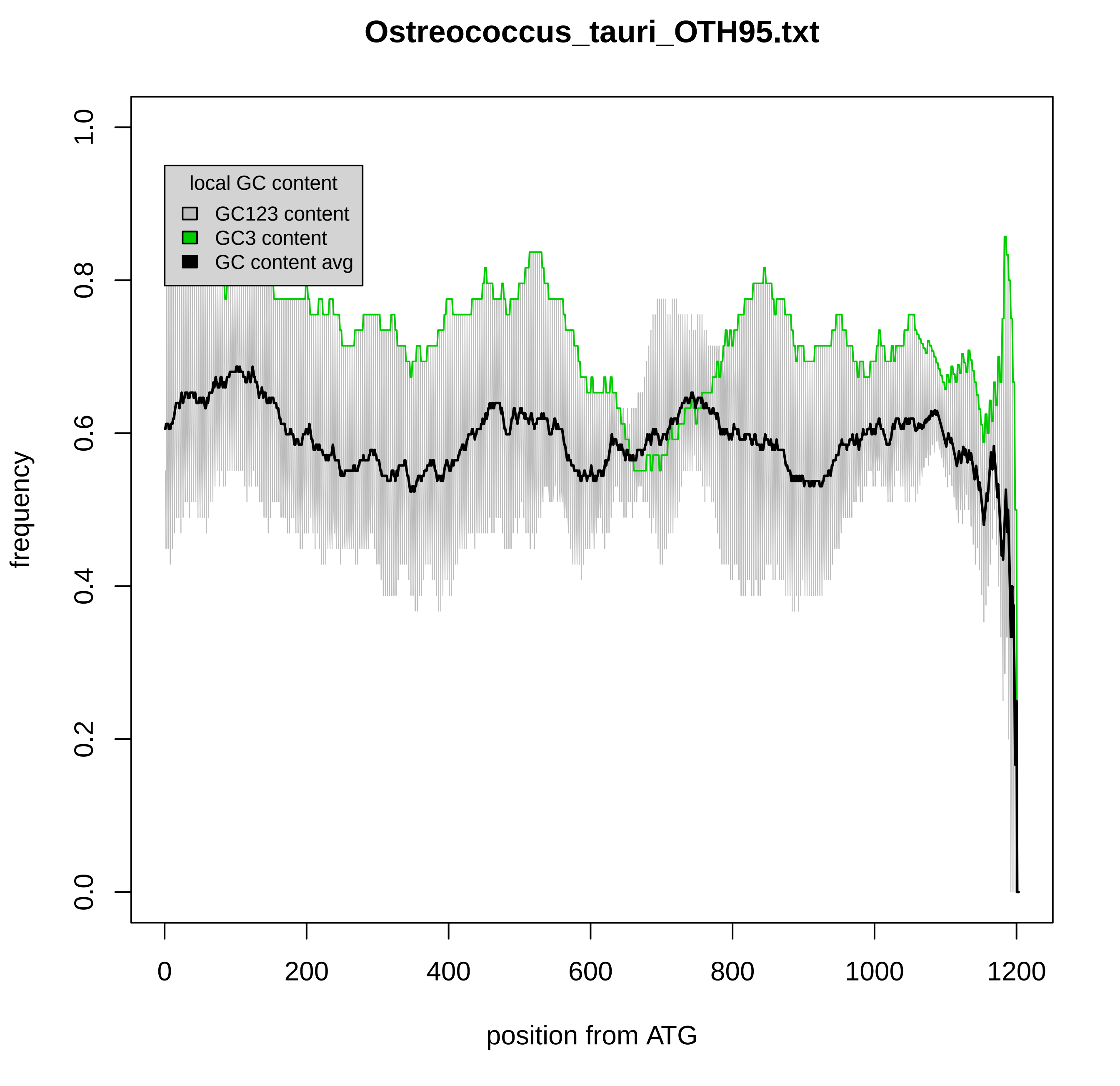

Ostreococcus_tauri_OTH95.txt
local GC content
GC123 content
GC3 content
GC content avg
frequency
position from ATG
0
200
400
600
800
1000
1200
0.0
0.2
0.4
0.6
0.8
1.0

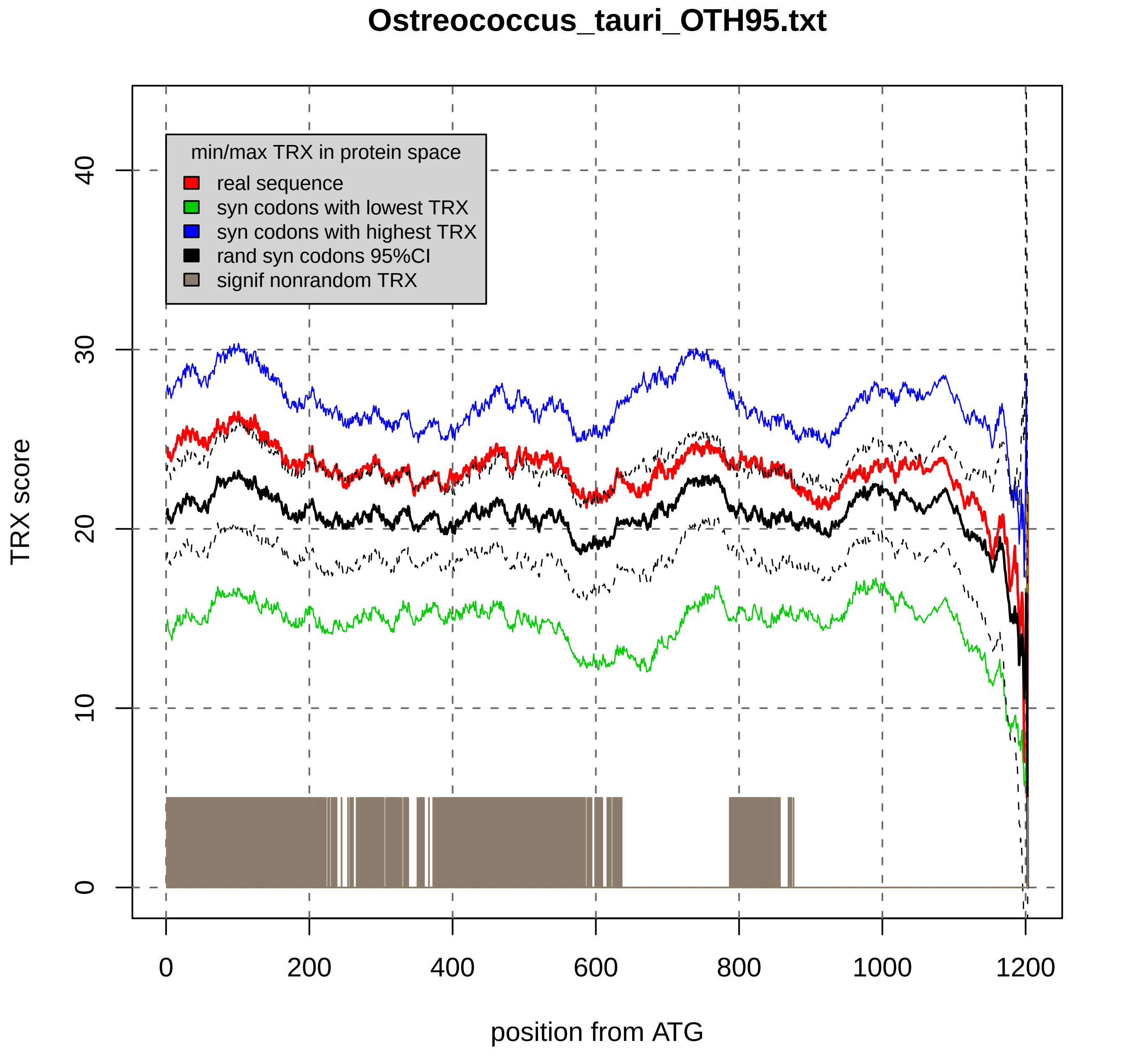

Ostreococcus_tauri_OTH95.txt
min/max TRX in protein space
real sequence
syn codons with lowest TRX
syn codons with highest TRX
rand syn codons 95%CI
signif nonrandom TRX
TRX score
position from ATG
0
10
20
30
40
0
200
400
600
800
1000
1200

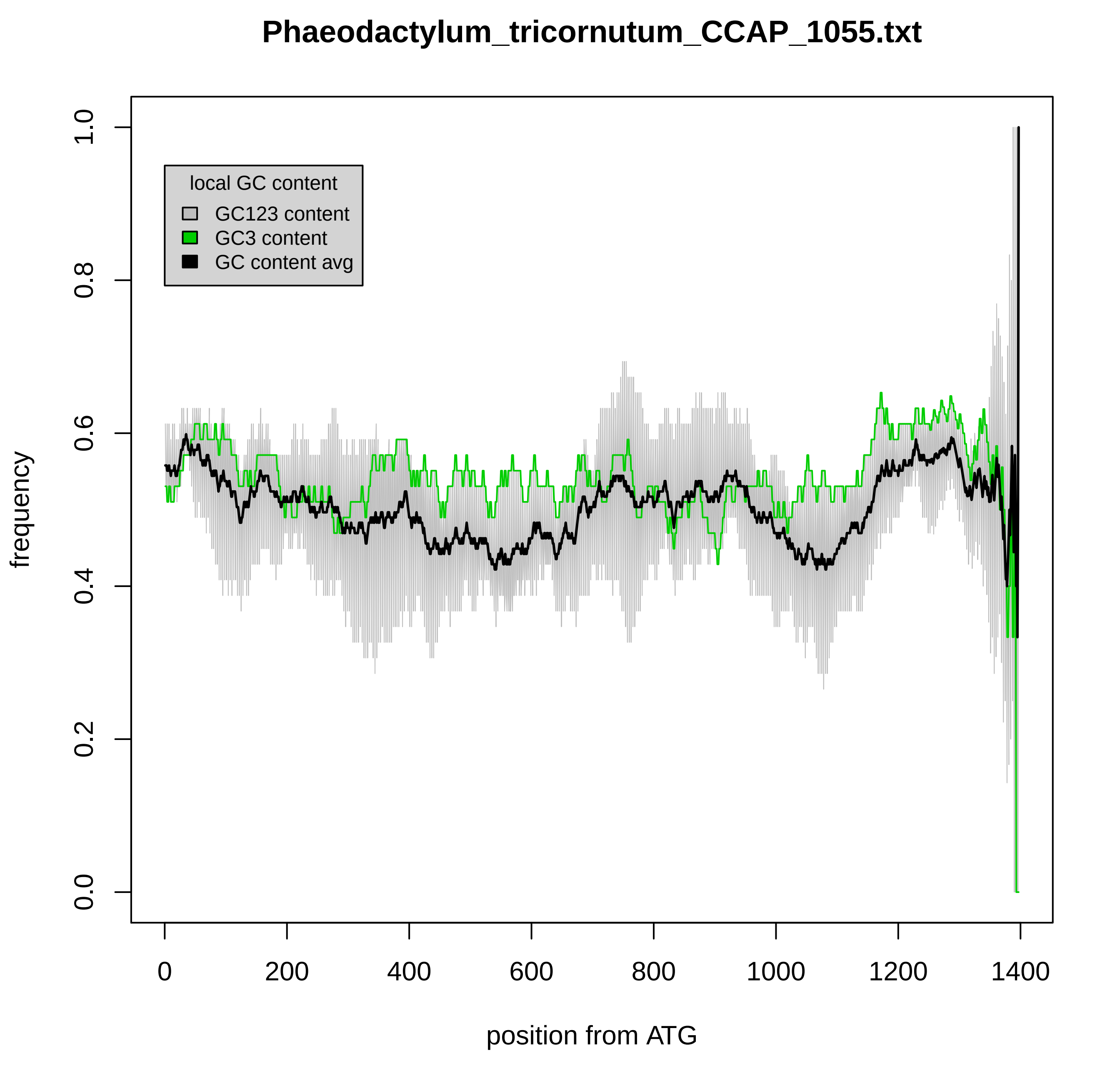
Phaeodactylum_tricornutum_CCAP_1055.txt
local GC content
GC123 content
GC3 content
GC content avg
frequency
position from ATG
0.0
0.2
0.4
0.6
0.8
1.0
0
200
400
600
800
1000
1200
1400

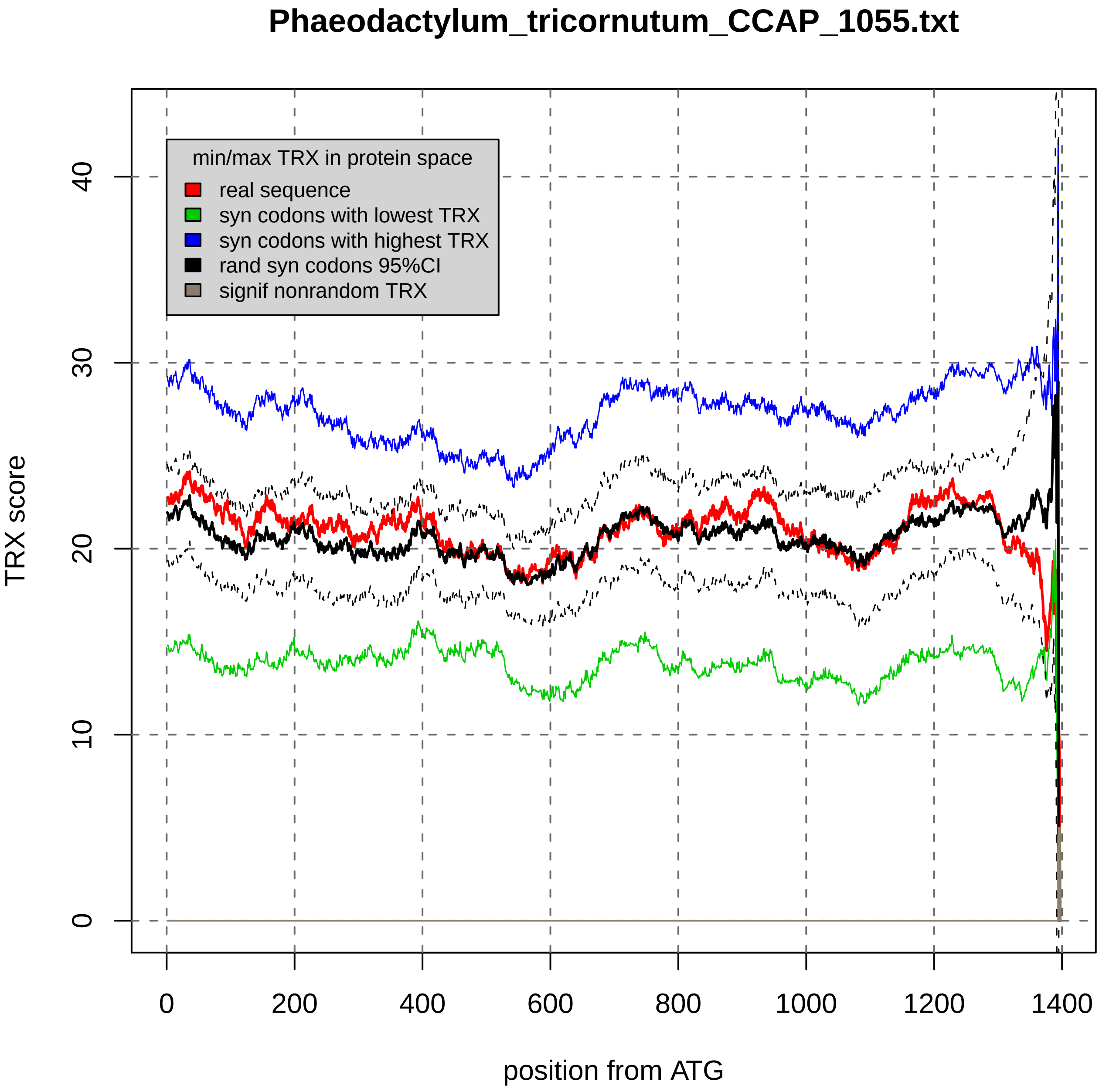
Phaeodactylum_tricornutum_CCAP_1055.txt
min/max TRX in protein space
real sequence
syn codons with lowest TRX
syn codons with highest TRX
rand syn codons 95%CI
signif nonrandom TRX
TRX score
position from ATG
0
10
20
30
40
0
200
400
600
800
1000
1200
1400

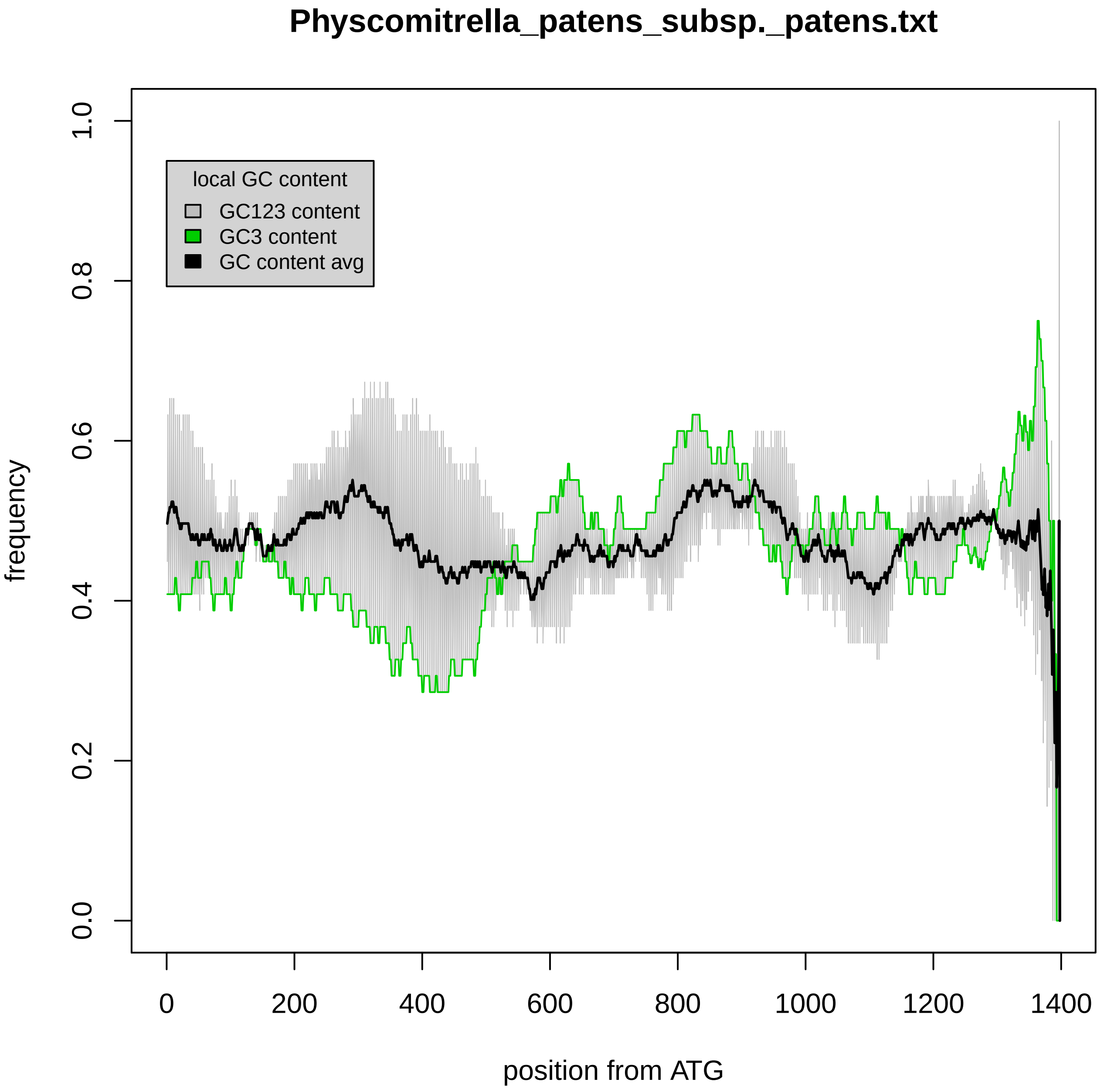
Physcomitrella_patens_subsp._patens.txt
local GC content
GC123 content
GC3 content
GC content avg
frequency
position from ATG
0.0
0.2
0.4
0.6
0.8
1.0
0
200
400
600
800
1000
1200
1400

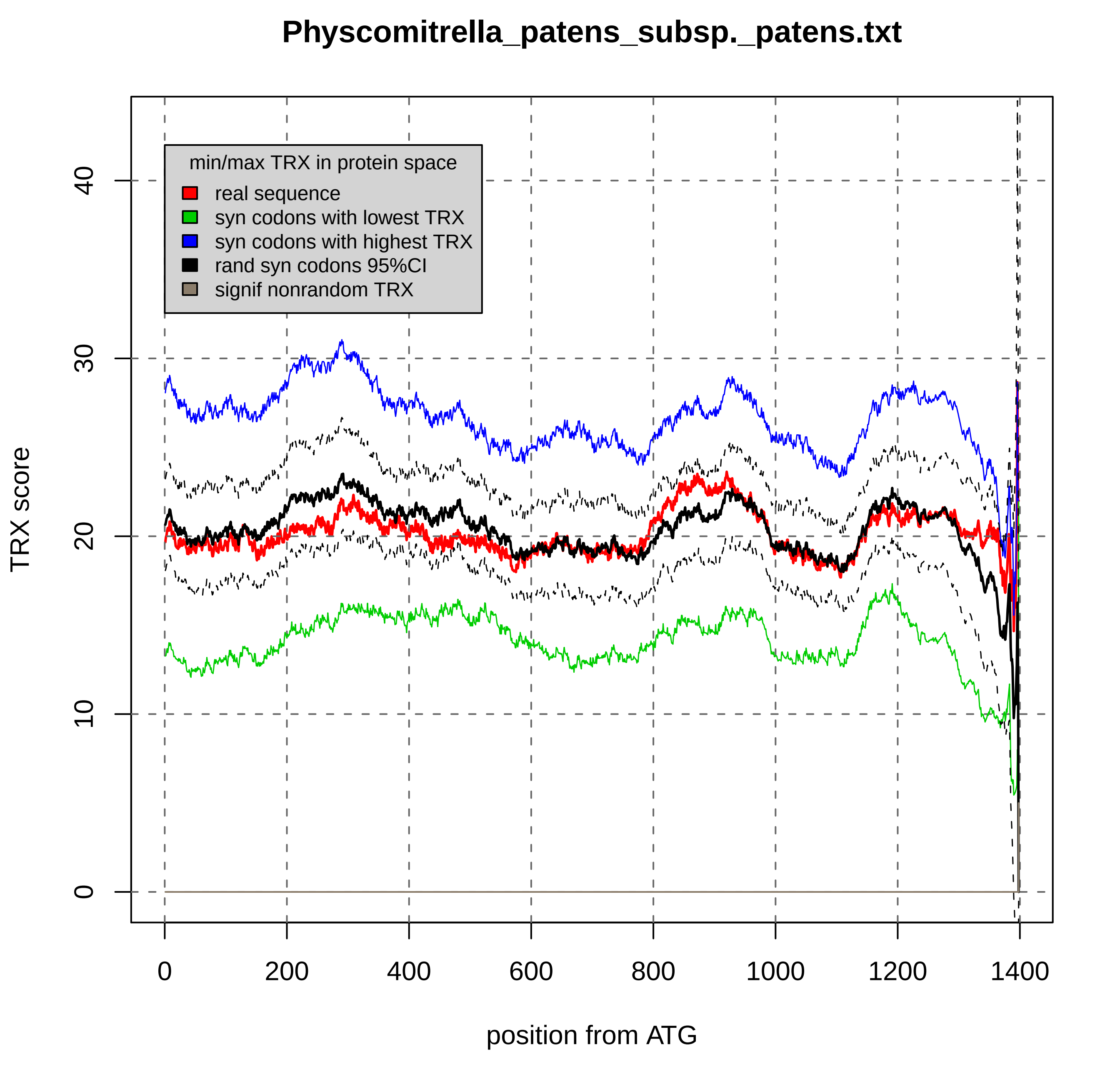

Physcomitrella_patens_subsp._patens.txt
min/max TRX in protein space
real sequence
syn codons with lowest TRX
syn codons with highest TRX
rand syn codons 95%CI
signif nonrandom TRX
TRX score
position from ATG
0
10
20
30
40
0
200
400
600
800
1000
1200
1400

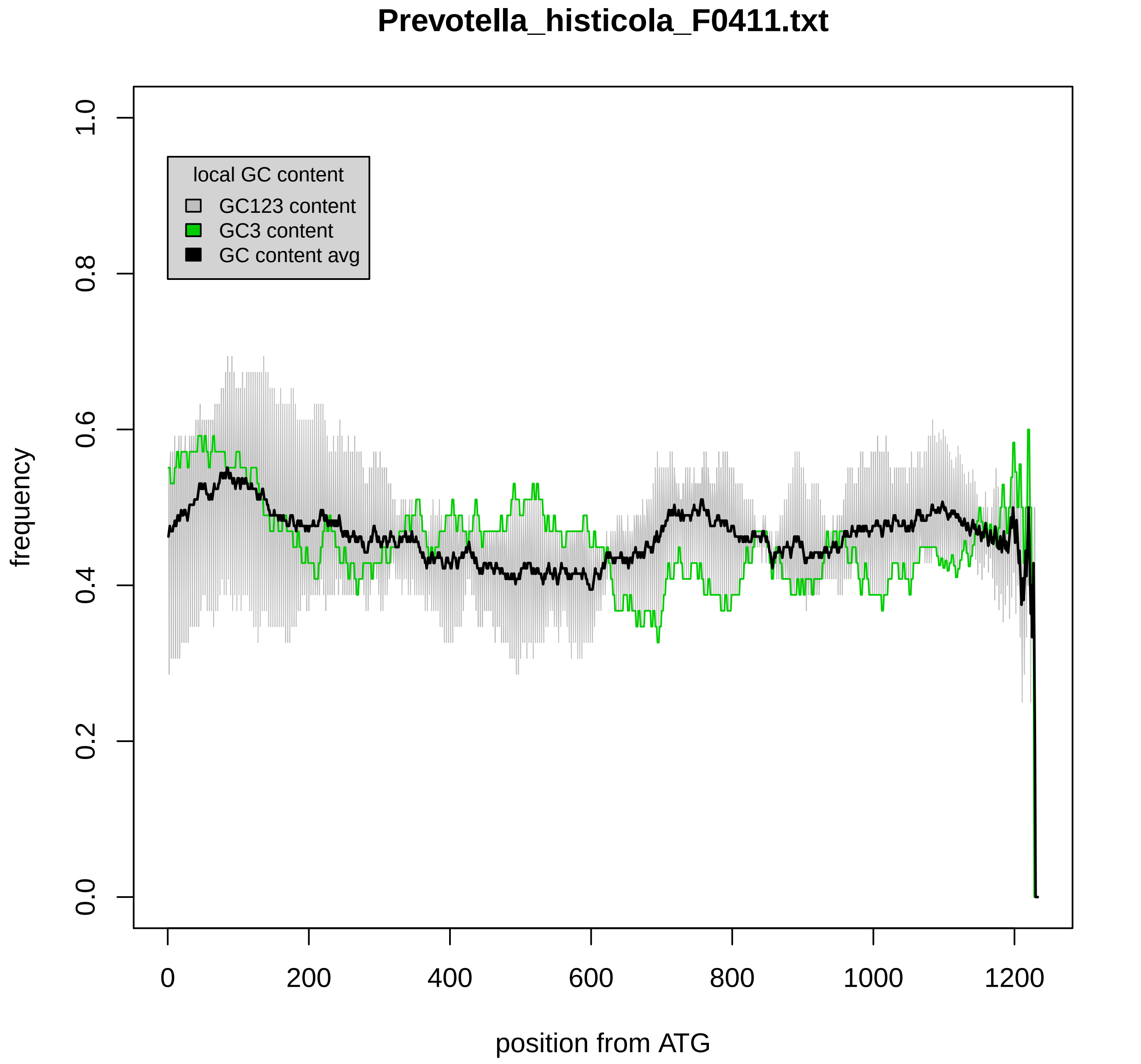

Prevotella_histicola_F0411.txt
local GC content
GC123 content
GC3 content
GC content avg
frequency
position from ATG
0.0
0.2
0.4
0.6
0.8
1.0
0
200
400
600
800
1000
1200

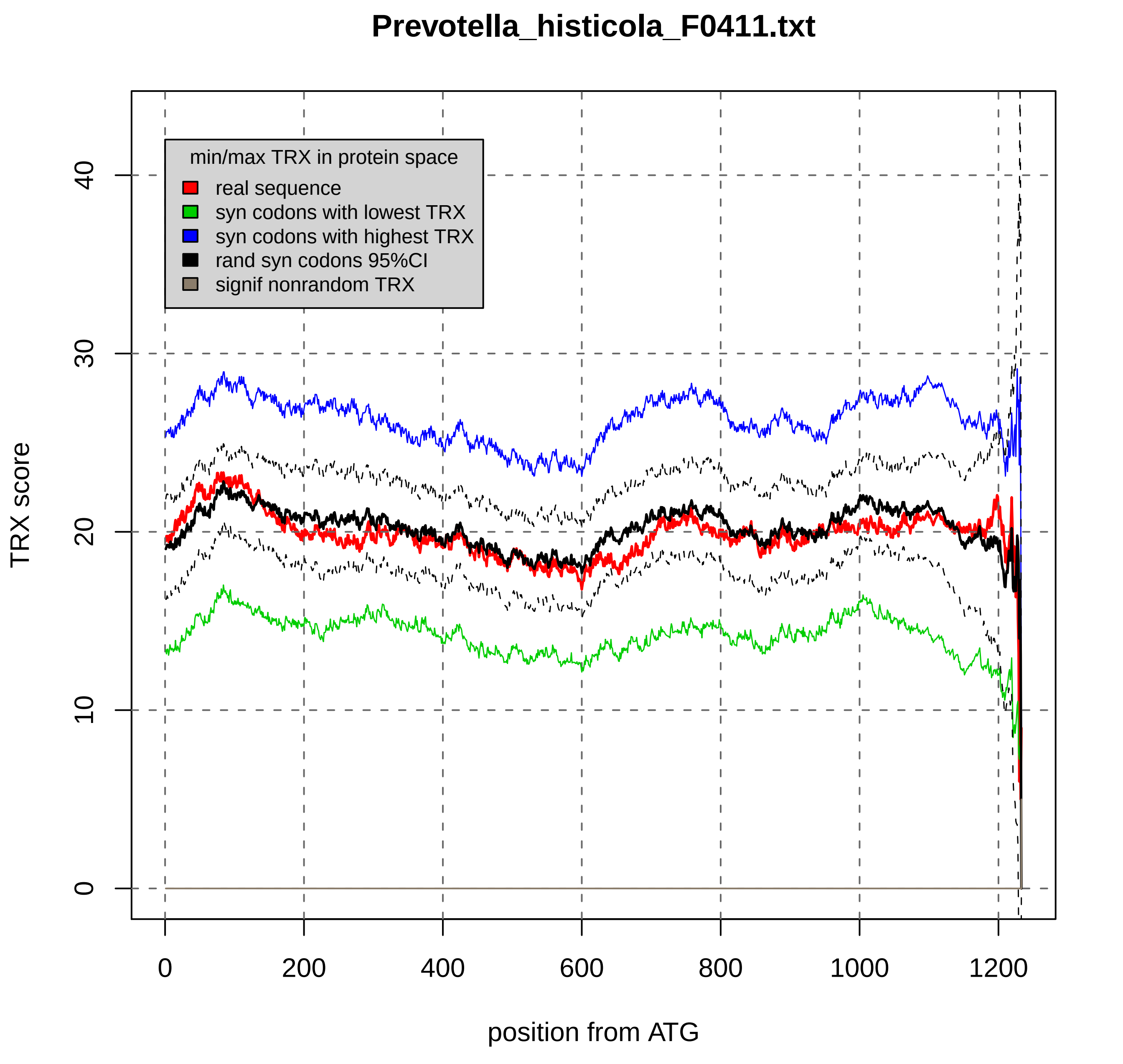
Prevotella_histicola_F0411.txt
min/max TRX in protein space
real sequence
syn codons with lowest TRX
syn codons with highest TRX
rand syn codons 95%CI
signif nonrandom TRX
TRX score
position from ATG
0
10
20
30
40
0
200
400
600
800
1000
1200

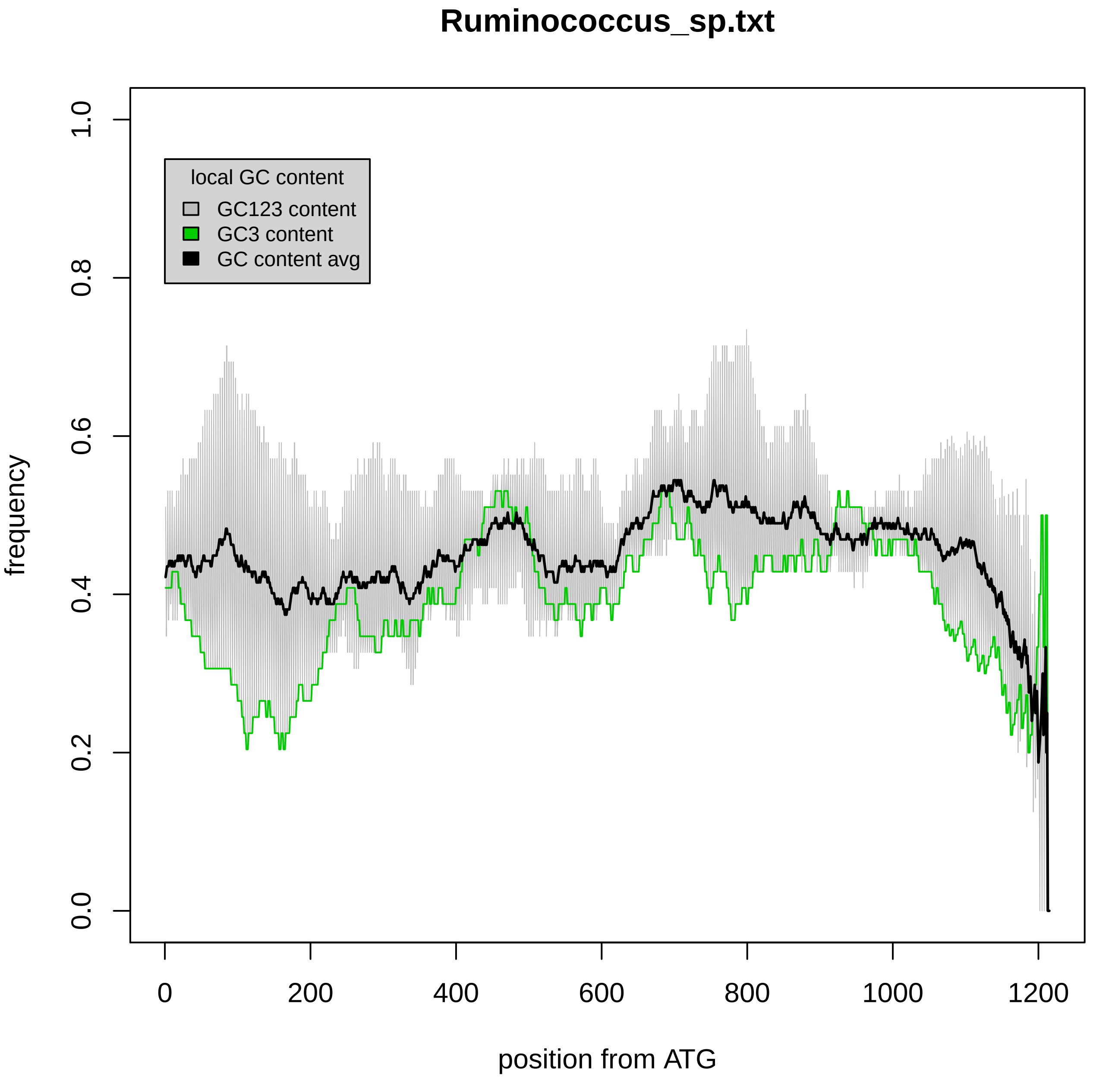
Ruminococcus_sp.txt
local GC content
GC123 content
GC3 content
GC content avg
frequency
position from ATG
0.0
0.2
0.4
0.6
0.8
1.0
0
200
400
600
800
1000
1200

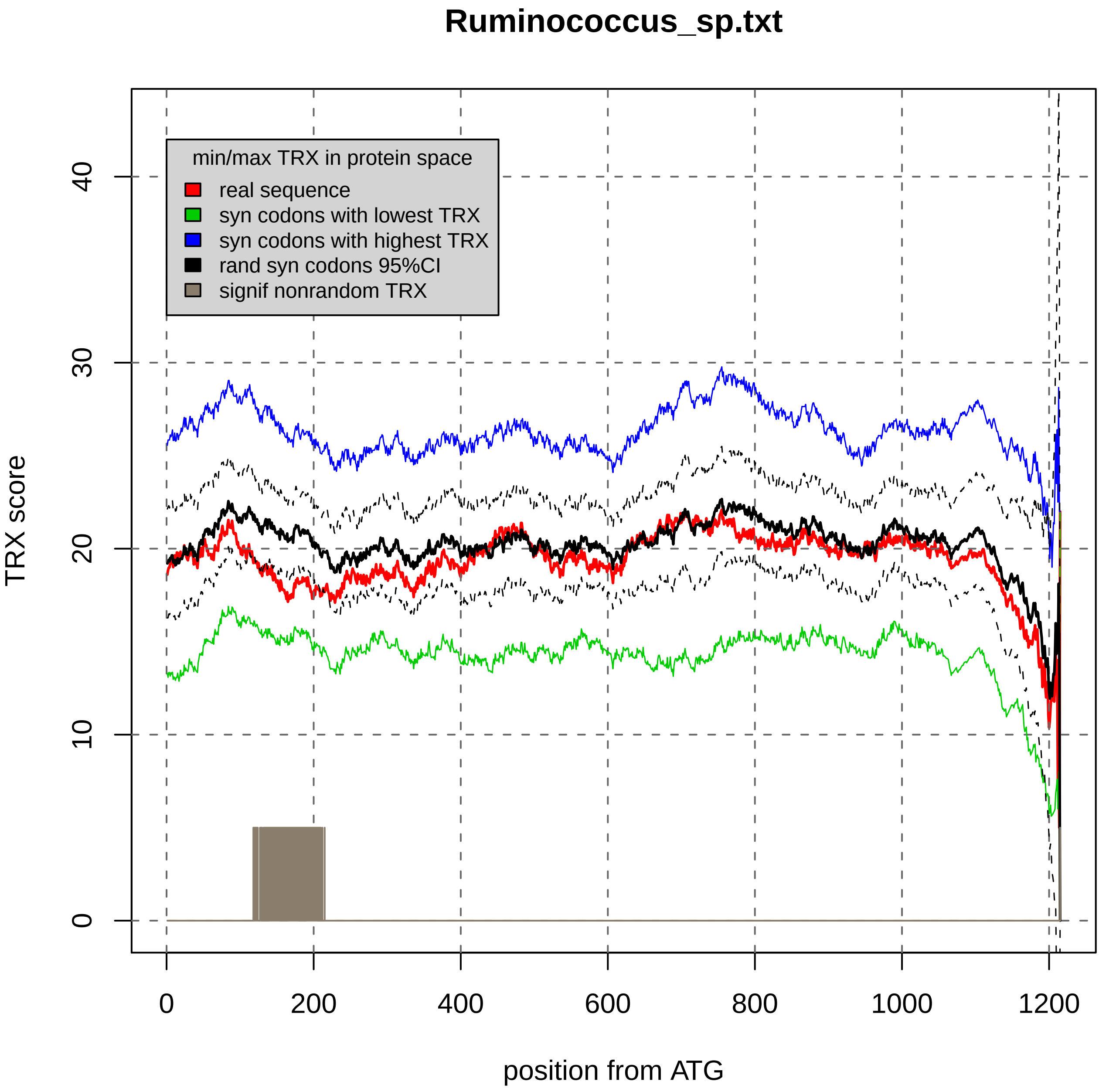

Ruminococcus_sp.txt
min/max TRX in protein space
real sequence
syn codons with lowest TRX
syn codons with highest TRX
rand syn codons 95%CI
signif nonrandom TRX
TRX score
position from ATG
0
10
20
30
40
0
200
400
600
800
1000
1200

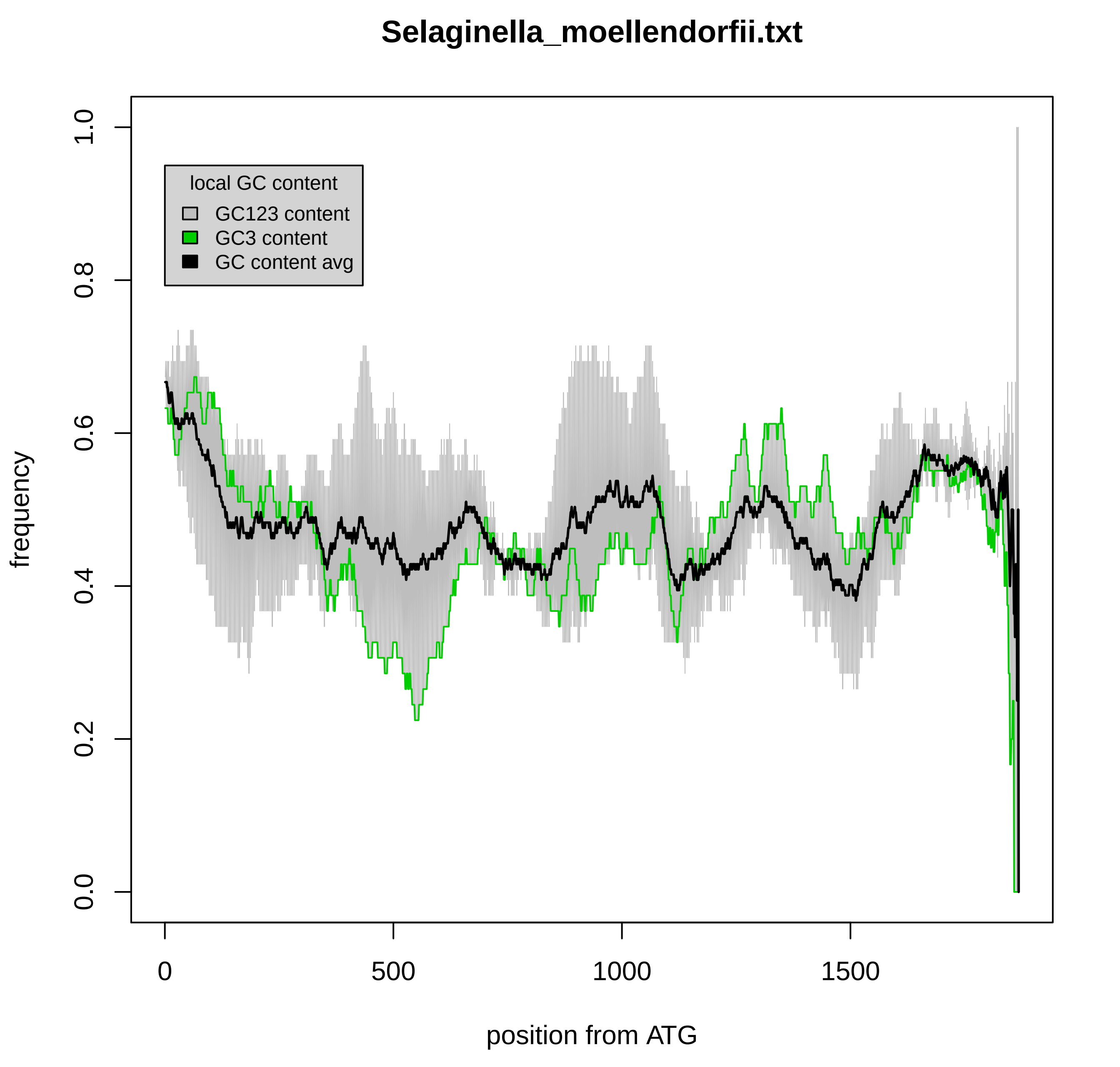

Selaginella_moellendorfii.txt
local GC content
GC123 content
GC3 content
GC content avg
frequency
0.0
0.2
0.4
0.6
0.8
1.0
0
500
1000
1500
position from ATG

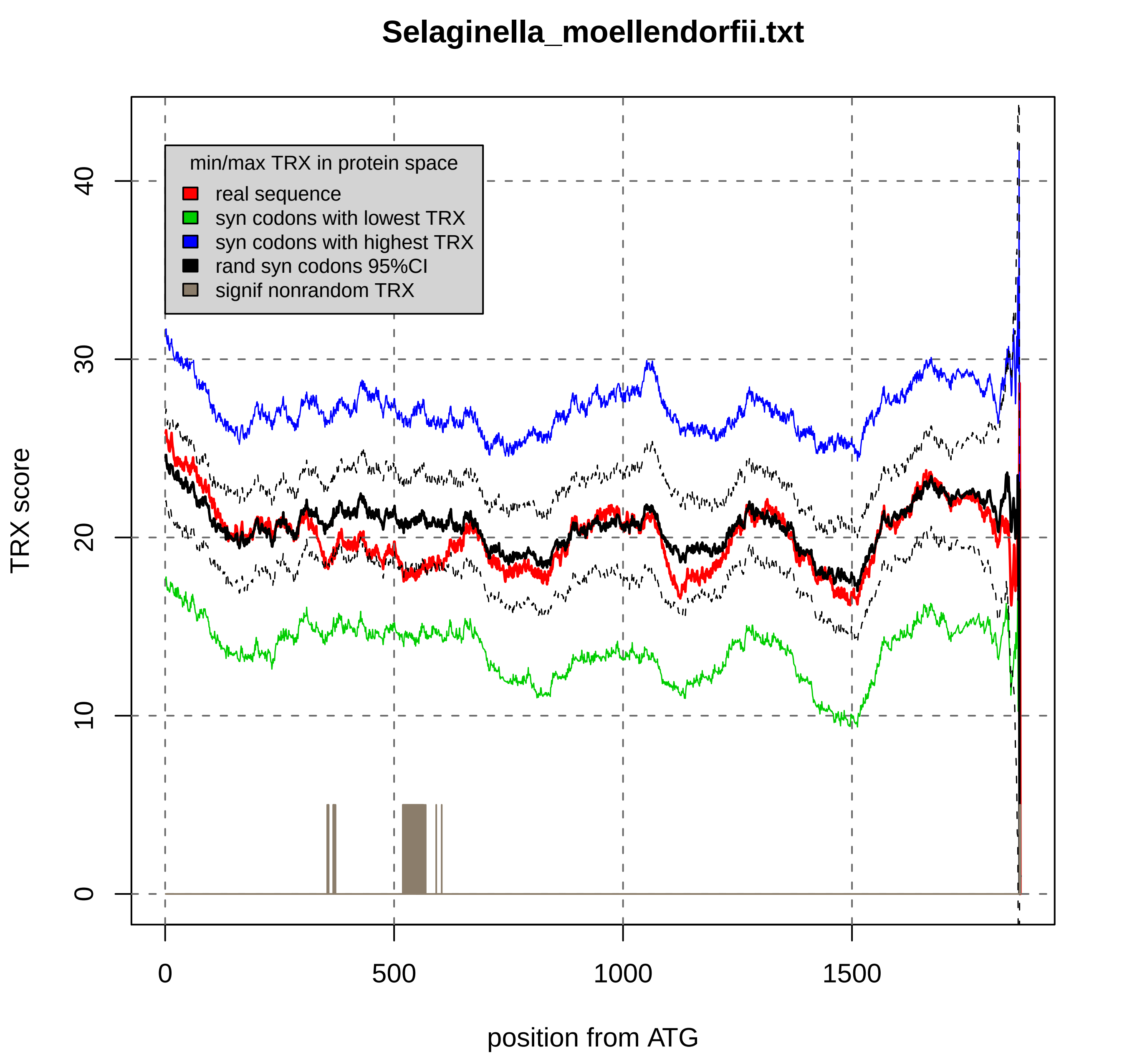

Selaginella_moellendorfii.txt
min/max TRX in protein space
real sequence
syn codons with lowest TRX
syn codons with highest TRX
rand syn codons 95%CI
signif nonrandom TRX
TRX score
0
10
20
30
40
0
500
1000
1500
position from ATG

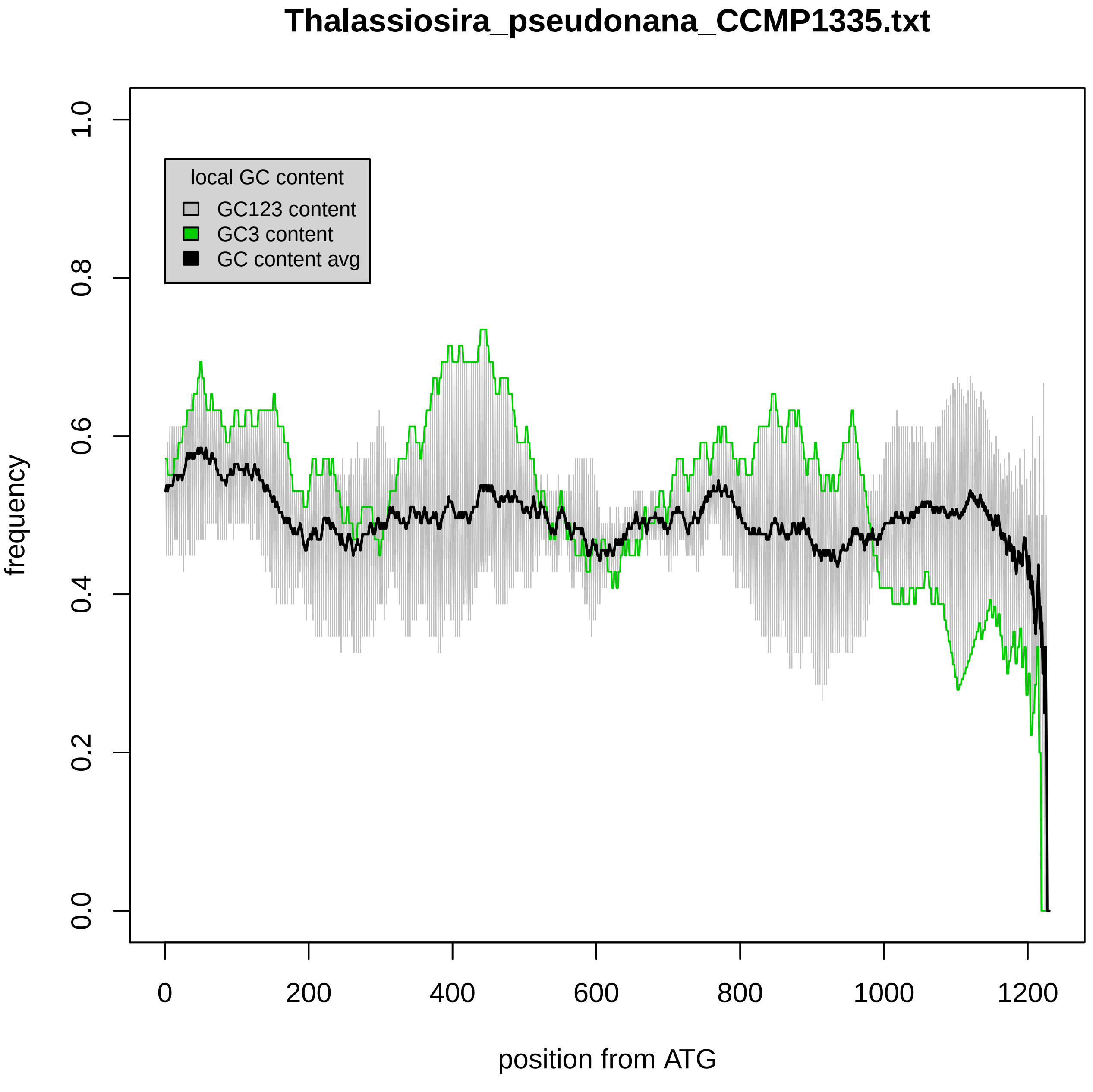

Thalassiosira_pseudonana_CCMP1335.txt
local GC content
GC123 content
GC3 content
GC content avg
frequency
0.0
0.2
0.4
0.6
0.8
1.0
0
200
400
600
800
1000
1200
position from ATG

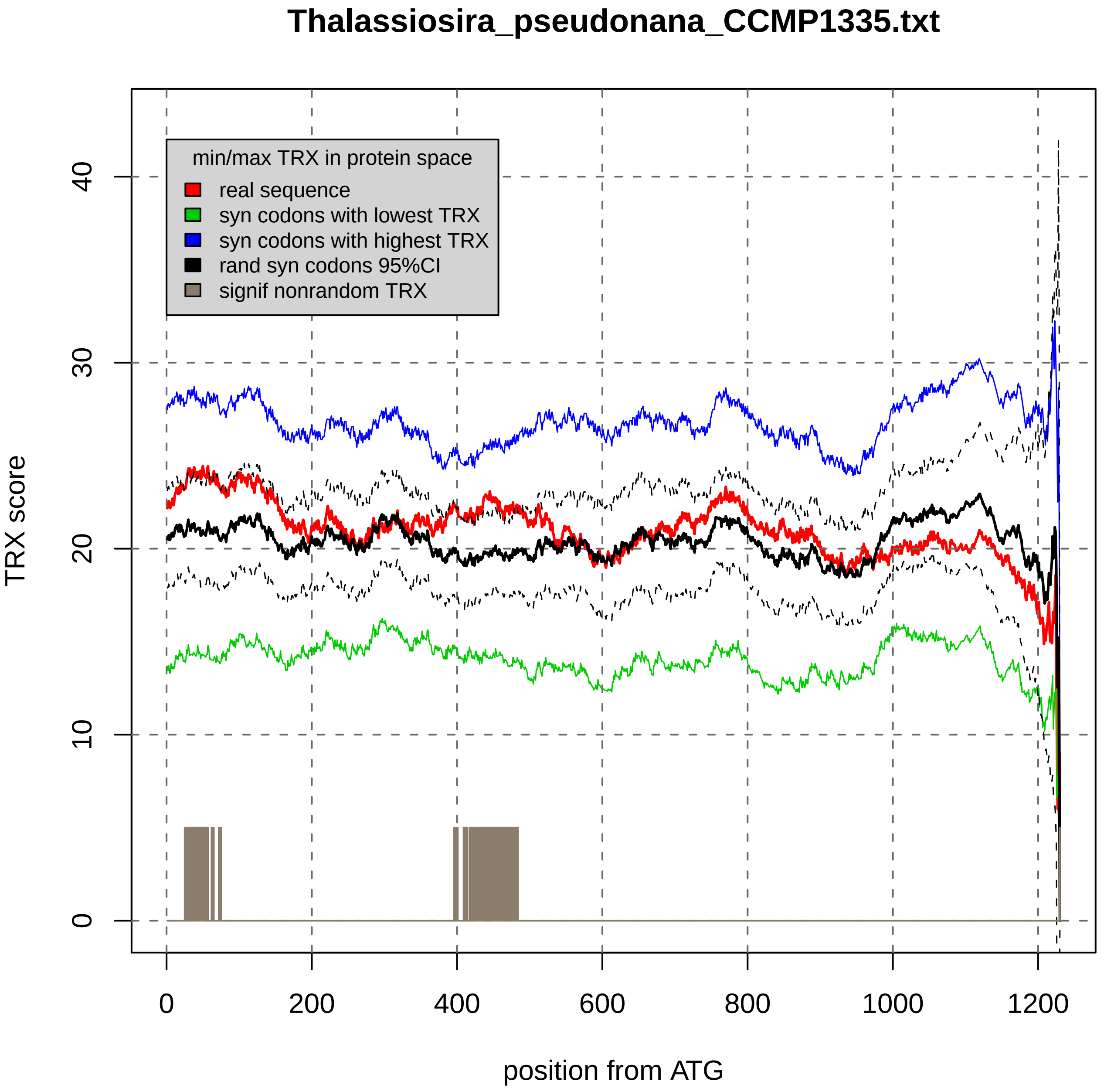
Thalassiosira_pseudonana_CCMP1335.txt
min/max TRX in protein space
real sequence
syn codons with lowest TRX
syn codons with highest TRX
rand syn codons 95%CI
signif nonrandom TRX
TRX score
position from ATG
0
10
20
30
40
0
200
400
600
800
1000
1200

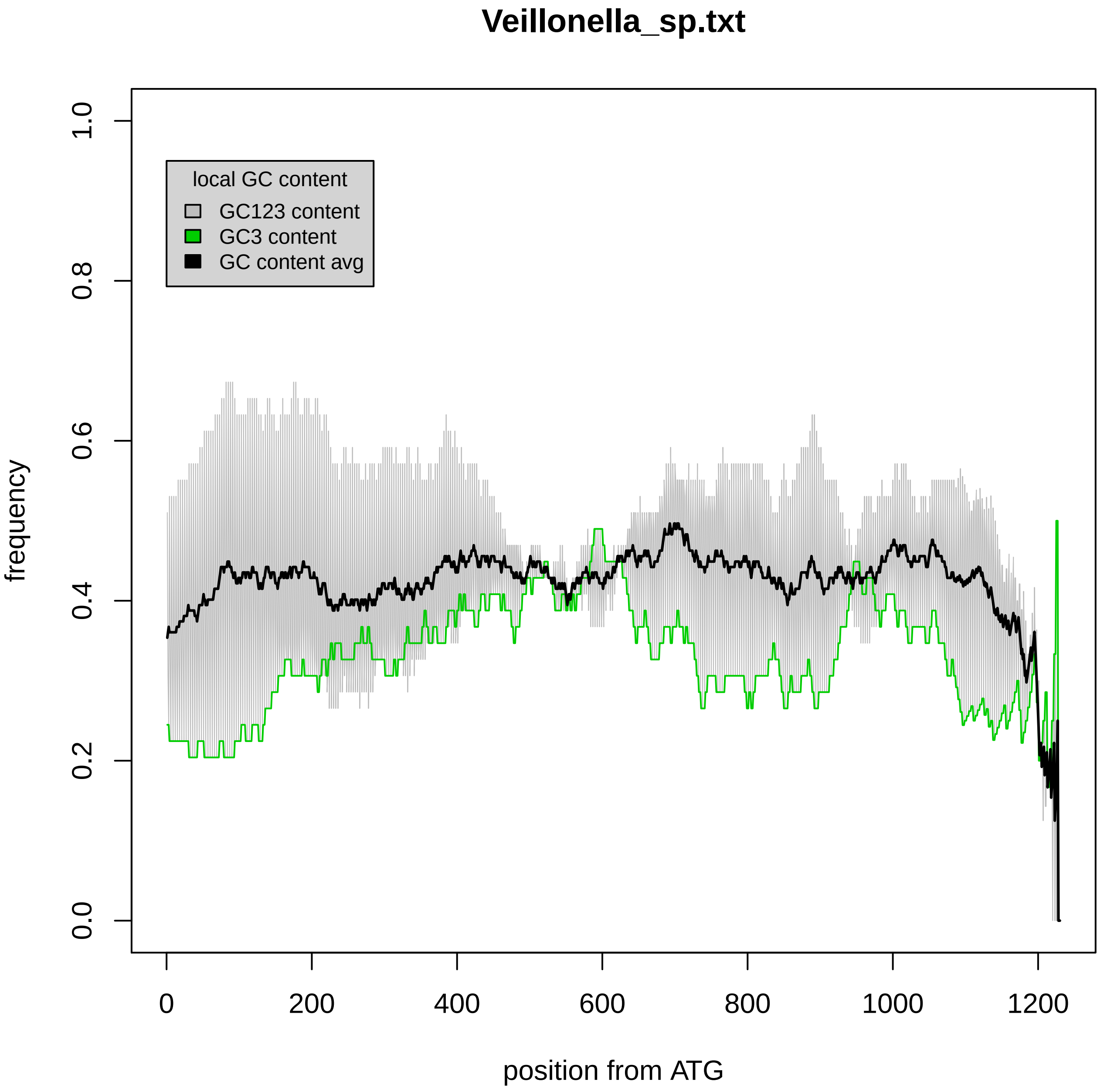

Veillonella_sp.txt
local GC content
GC123 content
GC3 content
GC content avg
frequency
0.0
0.2
0.4
0.6
0.8
1.0
0
200
400
600
800
1000
1200
position from ATG

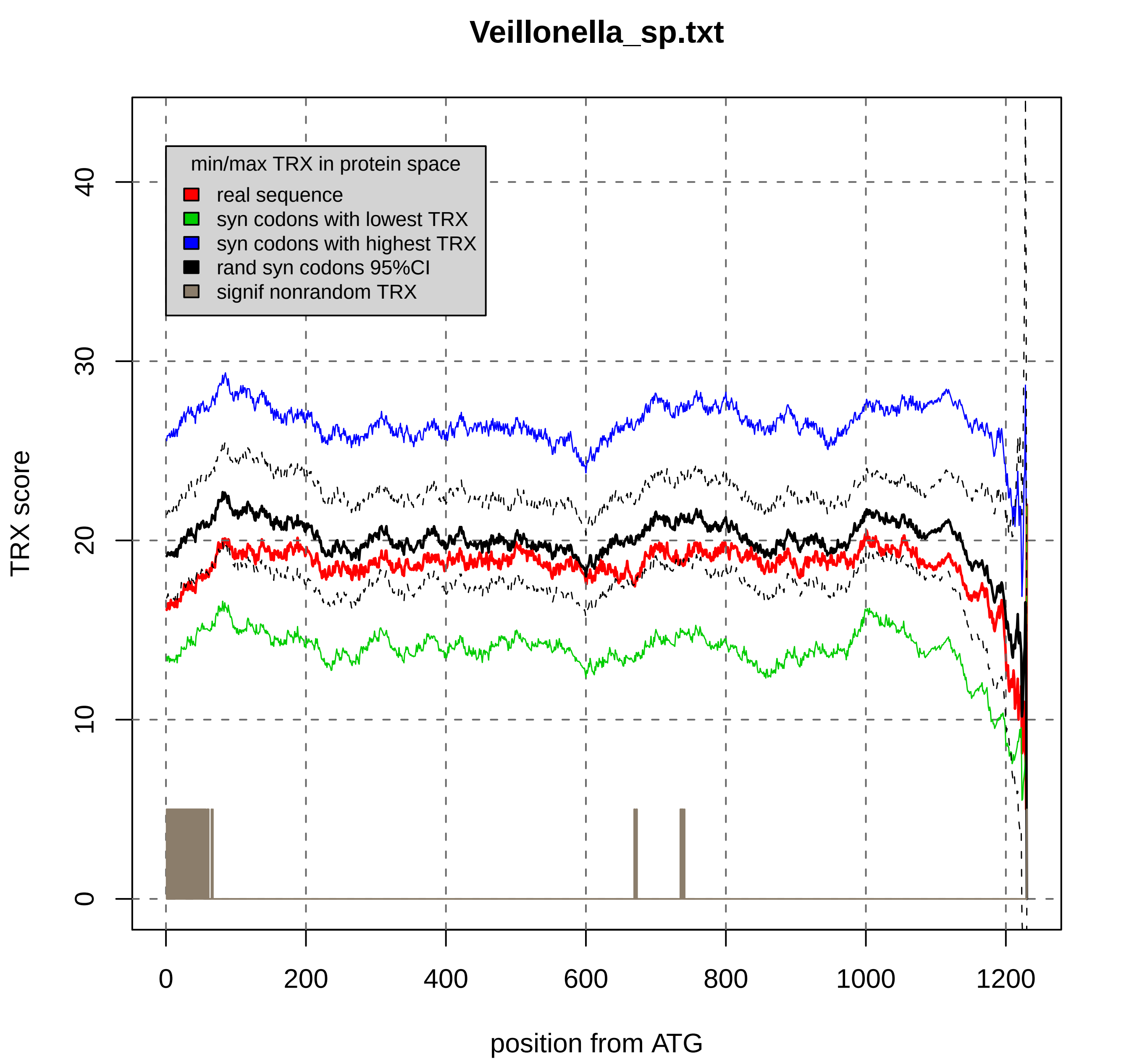
Veillonella_sp.txt
min/max TRX in protein space
real sequence
syn codons with lowest TRX
syn codons with highest TRX
rand syn codons 95%CI
signif nonrandom TRX
TRX score
position from ATG
0
10
20
30
40
0
200
400
600
800
1000
1200

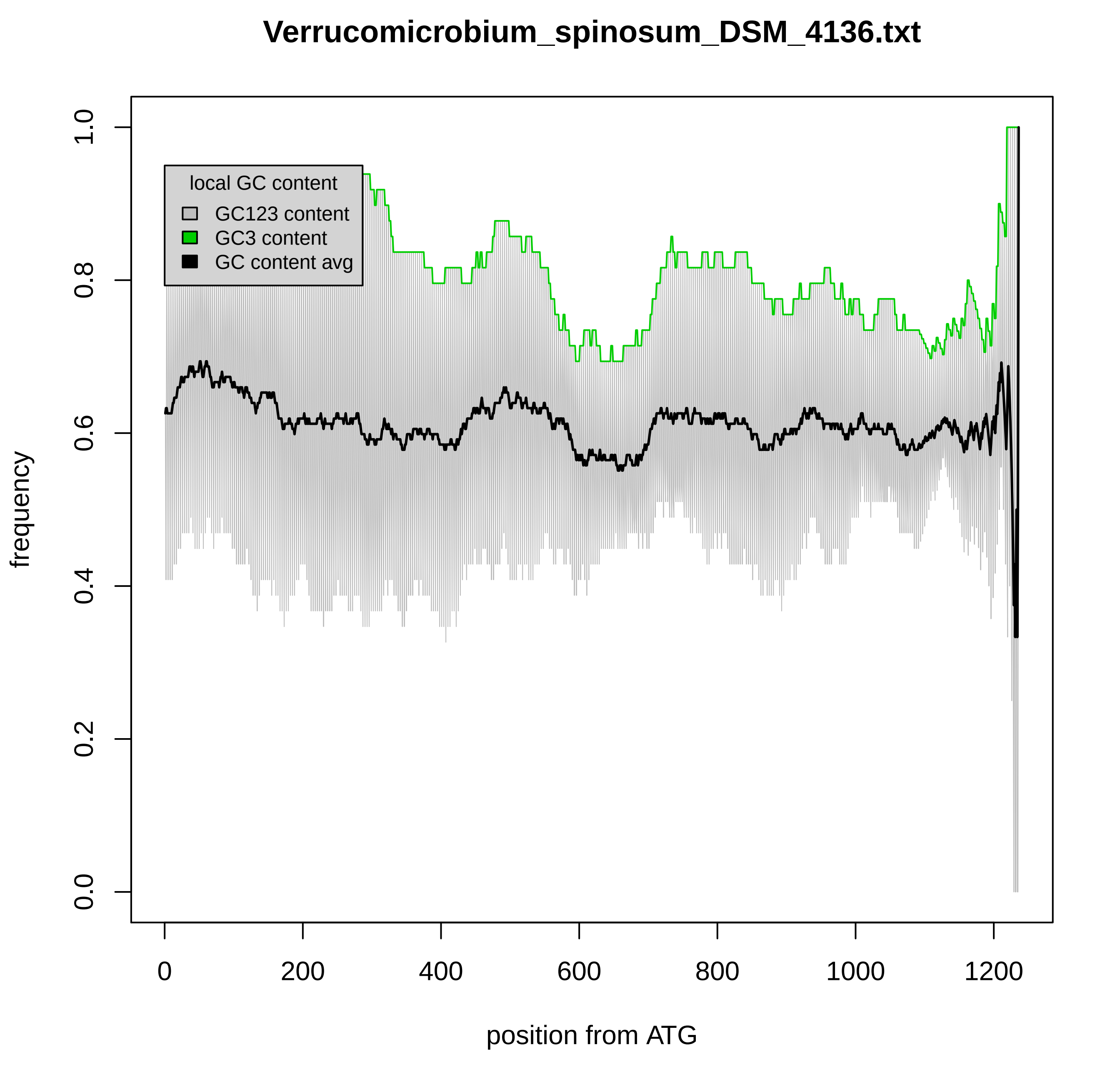
Verrucomicrobium_spinosum_DSM_4136.txt
local GC content
GC123 content
GC3 content
GC content avg
frequency
position from ATG
0.0
0.2
0.4
0.6
0.8
1.0
0
200
400
600
800
1000
1200

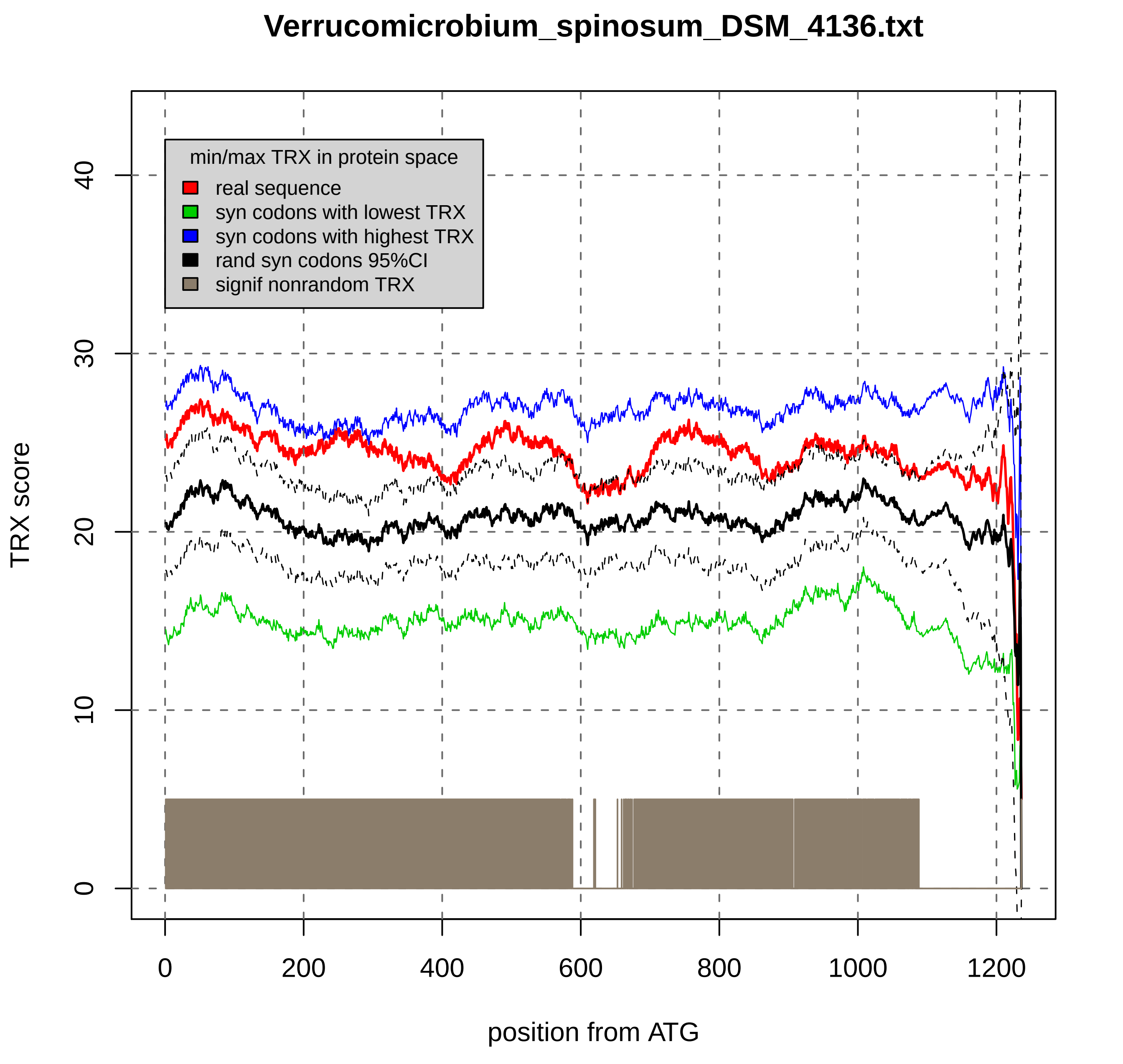
Verrucomicrobium_spinosum_DSM_4136.txt
min/max TRX in protein space
real sequence
syn codons with lowest TRX
syn codons with highest TRX
rand syn codons 95%CI
signif nonrandom TRX
TRX score
0
10
20
30
40
0
200
400
600
800
1000
1200
position from ATG
