# Supplementary material for: Synonymous codon bias and functional constraint on GC3-related DNA backbone dynamics in the prokaryotic nucleoid
Source: Nucleic Acids Res. 2014 Sep 8;42(17):10915–26. doi: 10.1093/nar/gku811 (PMC4176184; doi:10.1093/nar/gku811)
Supplement: SUPPLEMENTARY DATA [file supp_gku811_nar-01772-z-2014-File009.zip › NAR-01772-Z-2014.R1 Suppl files/SuppFileC_caption.pdf]

Supplementary File C - The intrinsic DNA flexibility and GC3 of 20 representative genes from the Carsonella genome. The plots below are read identically to those in Figure 5. Plots include observed (red), minimum (green), maximum (blue) and average synonymous (black)intrinsic flexibility TRX scores and respective trends in overall GC content (black), GC content in each reading frame (gray) and GC3 (green).

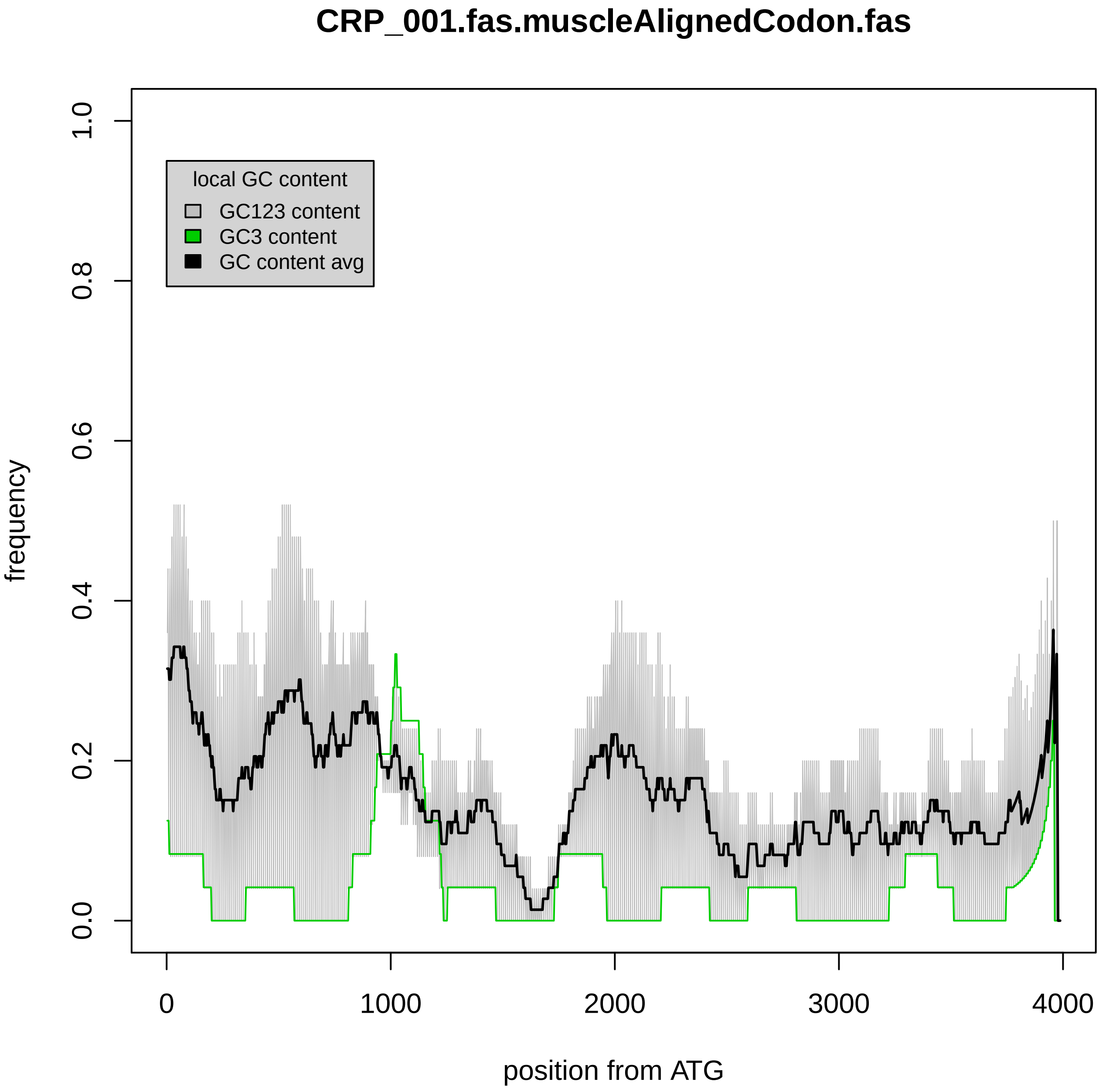

CRP_001.fas.muscleAlignedCodon.fas
local GC content
GC123 content
GC3 content
GC content avg
frequency
0.0
0.2
0.4
0.6
0.8
1.0
0
1000
2000
3000
4000
position from ATG

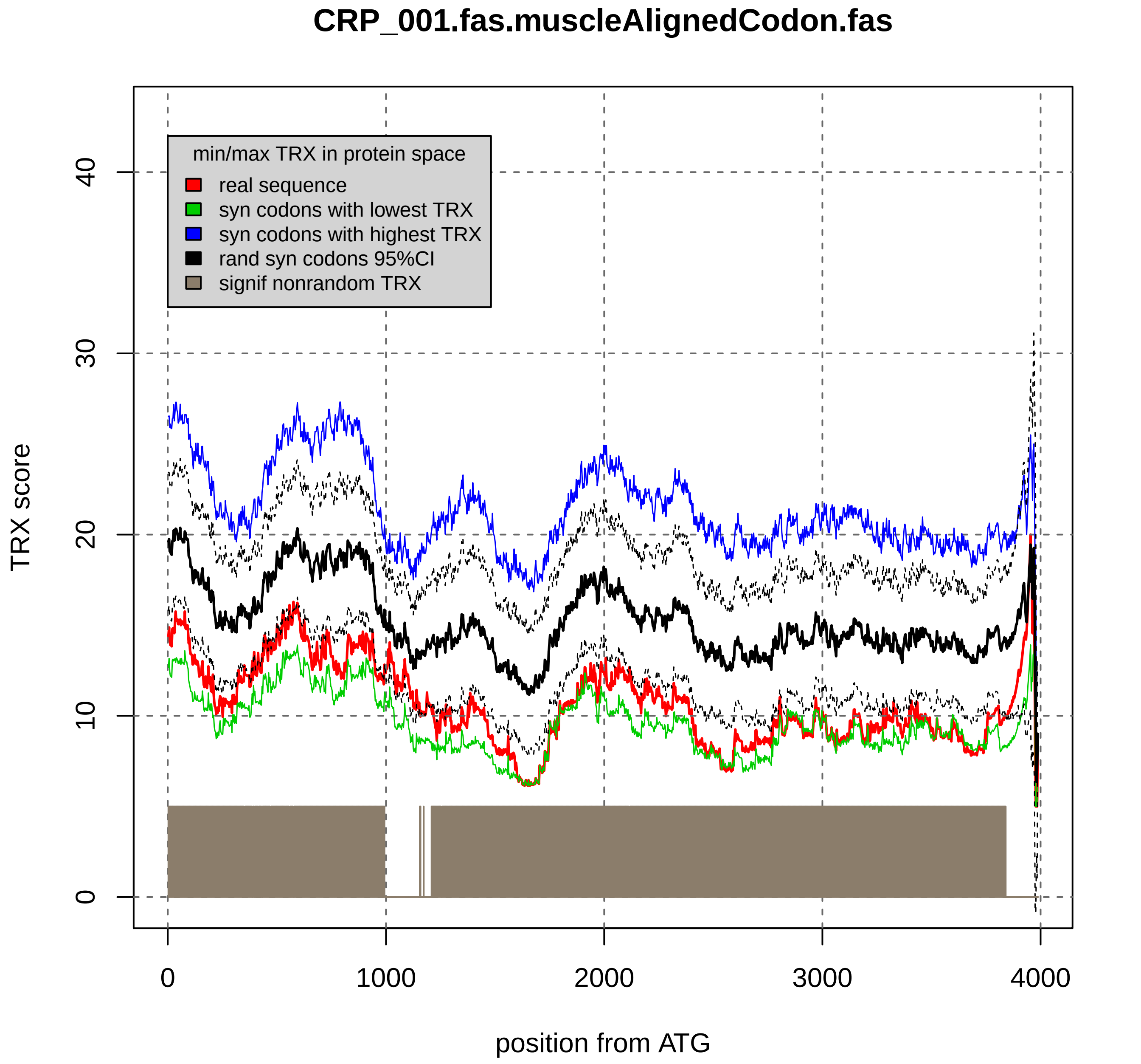
CRP_001.fas.muscleAlignedCodon.fas
min/max TRX in protein space
real sequence
syn codons with lowest TRX
syn codons with highest TRX
rand syn codons 95%CI
signif nonrandom TRX
TRX score
position from ATG
0
10
20
30
40
0
1000
2000
3000
4000

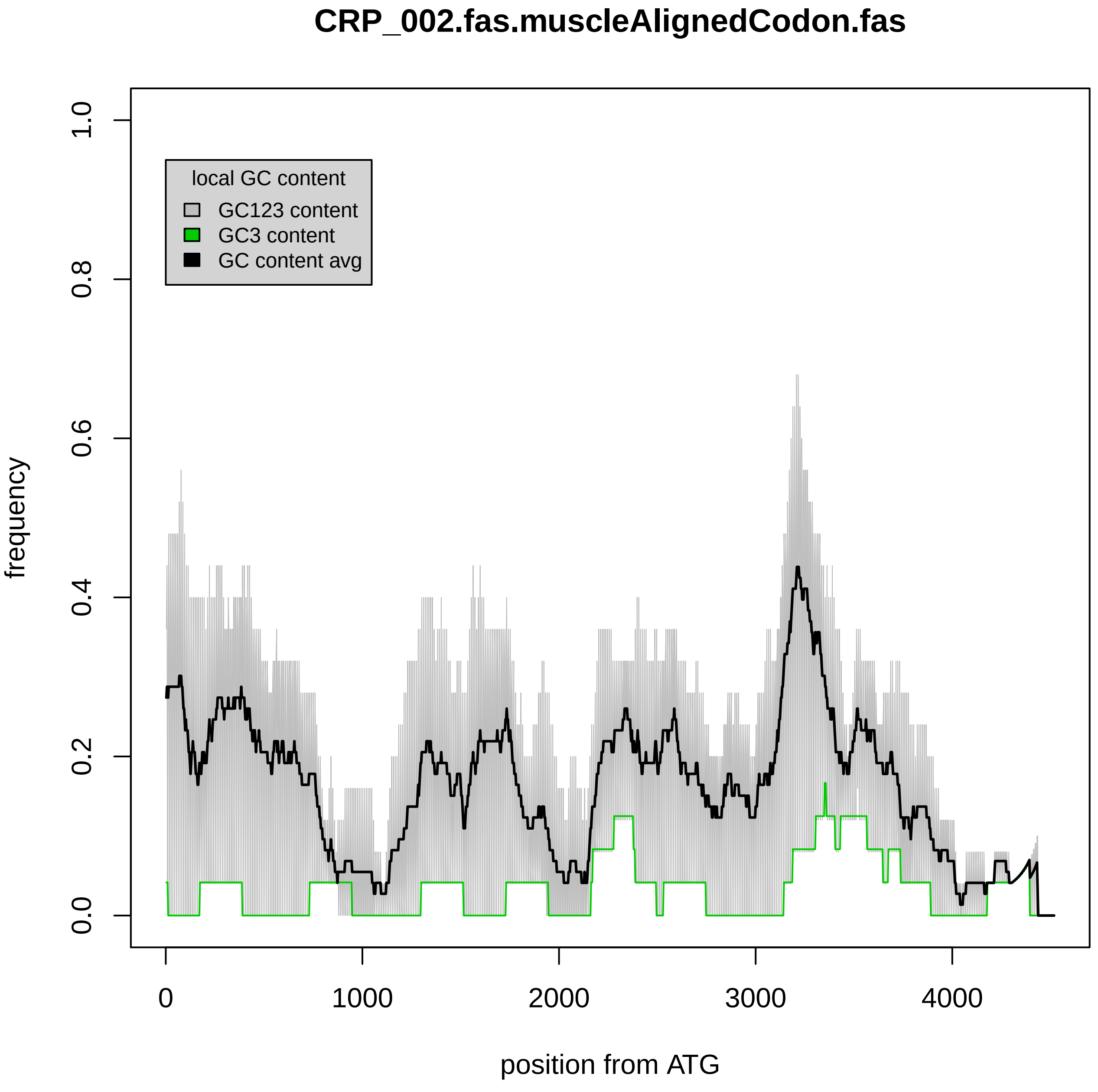
CRP_002.fas.muscleAlignedCodon.fas
local GC content
GC123 content
GC3 content
GC content avg
frequency
0.0
0.2
0.4
0.6
0.8
1.0
0
1000
2000
3000
4000
position from ATG

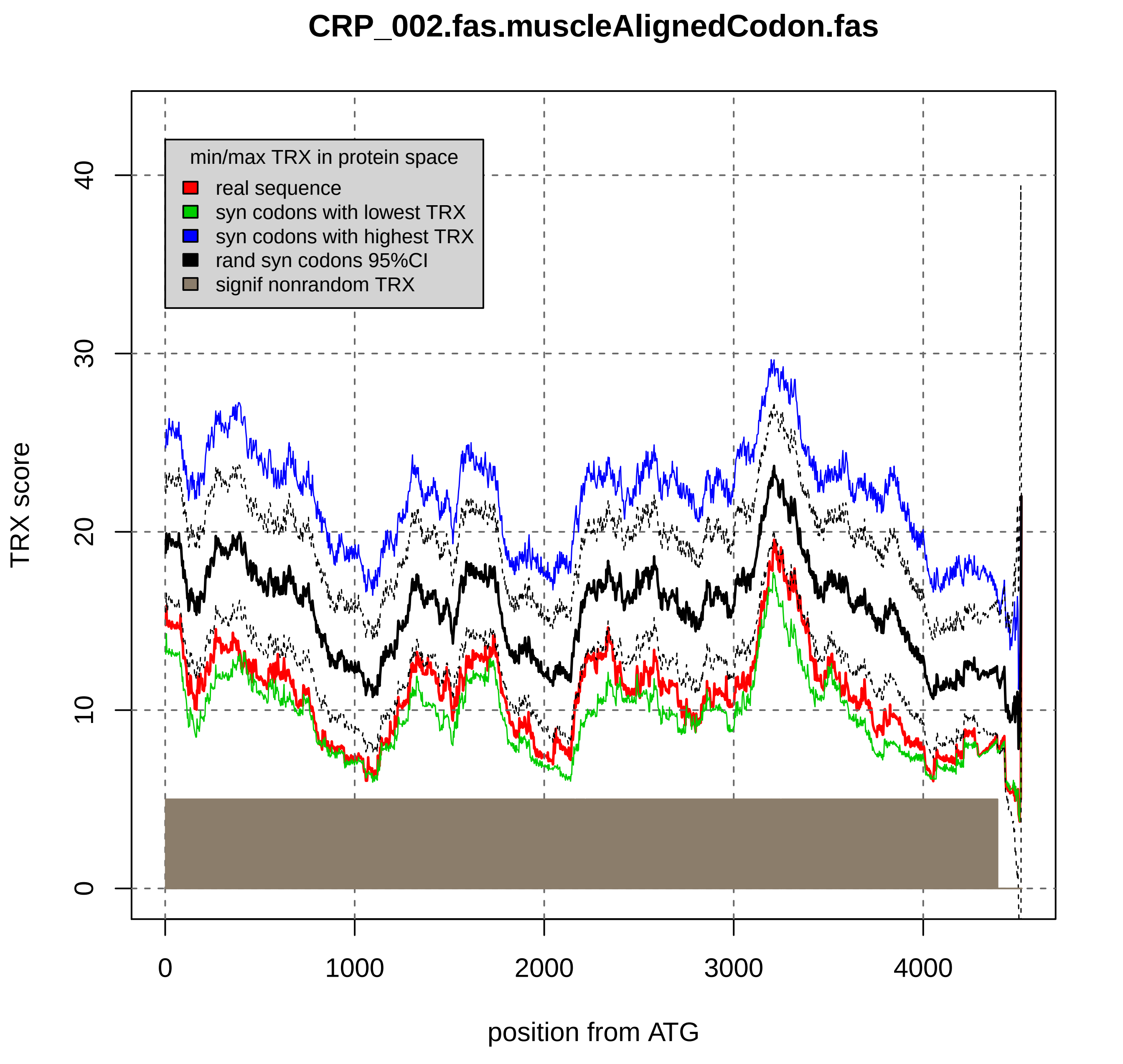

CRP_002.fas.muscleAlignedCodon.fas
min/max TRX in protein space
real sequence
syn codons with lowest TRX
syn codons with highest TRX
rand syn codons 95%CI
signif nonrandom TRX
TRX score
0
10
20
30
40
0
1000
2000
3000
4000
position from ATG

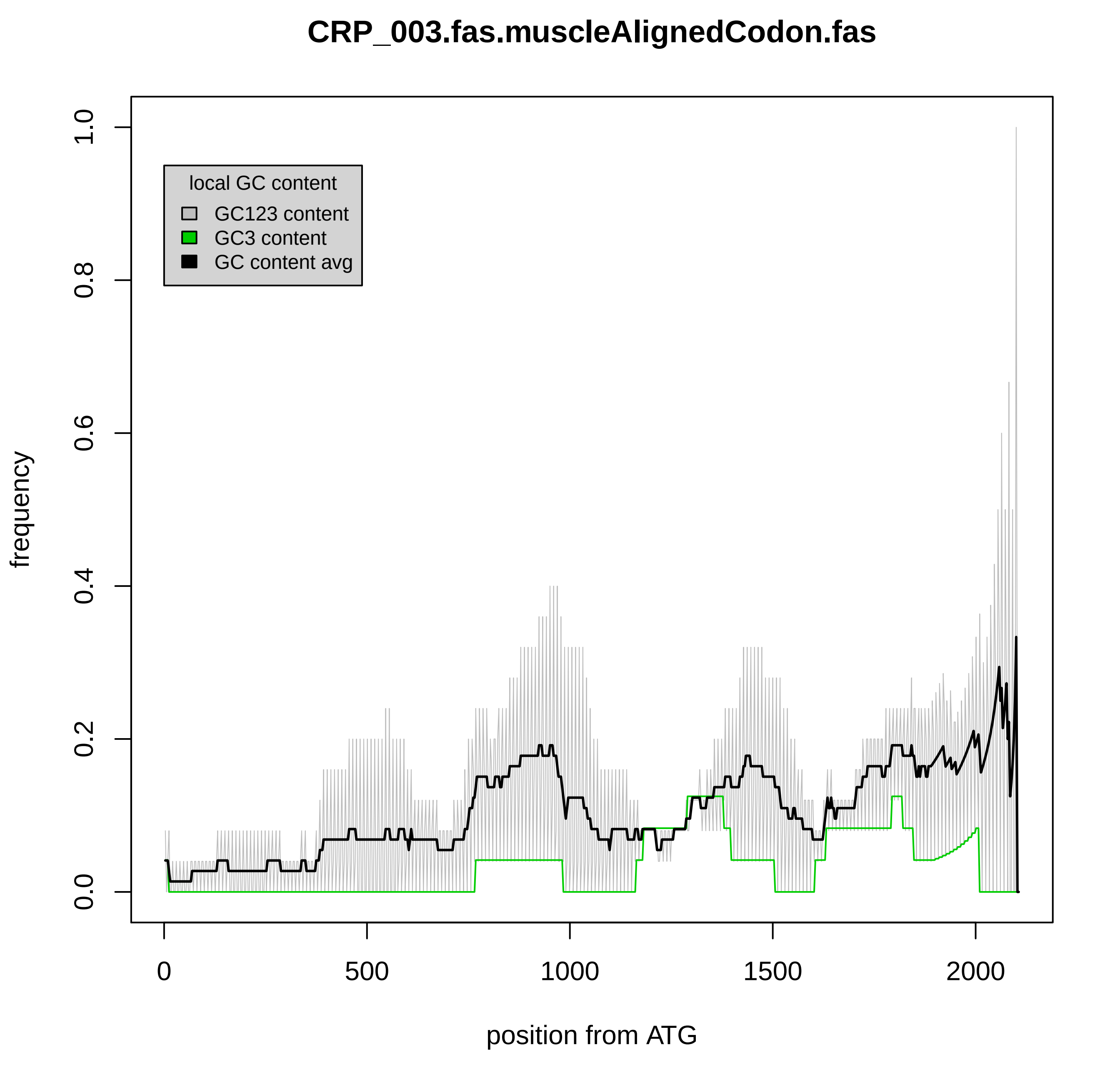

CRP_003.fas.muscleAlignedCodon.fas
local GC content
GC123 content
GC3 content
GC content avg
frequency
0.0
0.2
0.4
0.6
0.8
1.0
0
500
1000
1500
2000
position from ATG

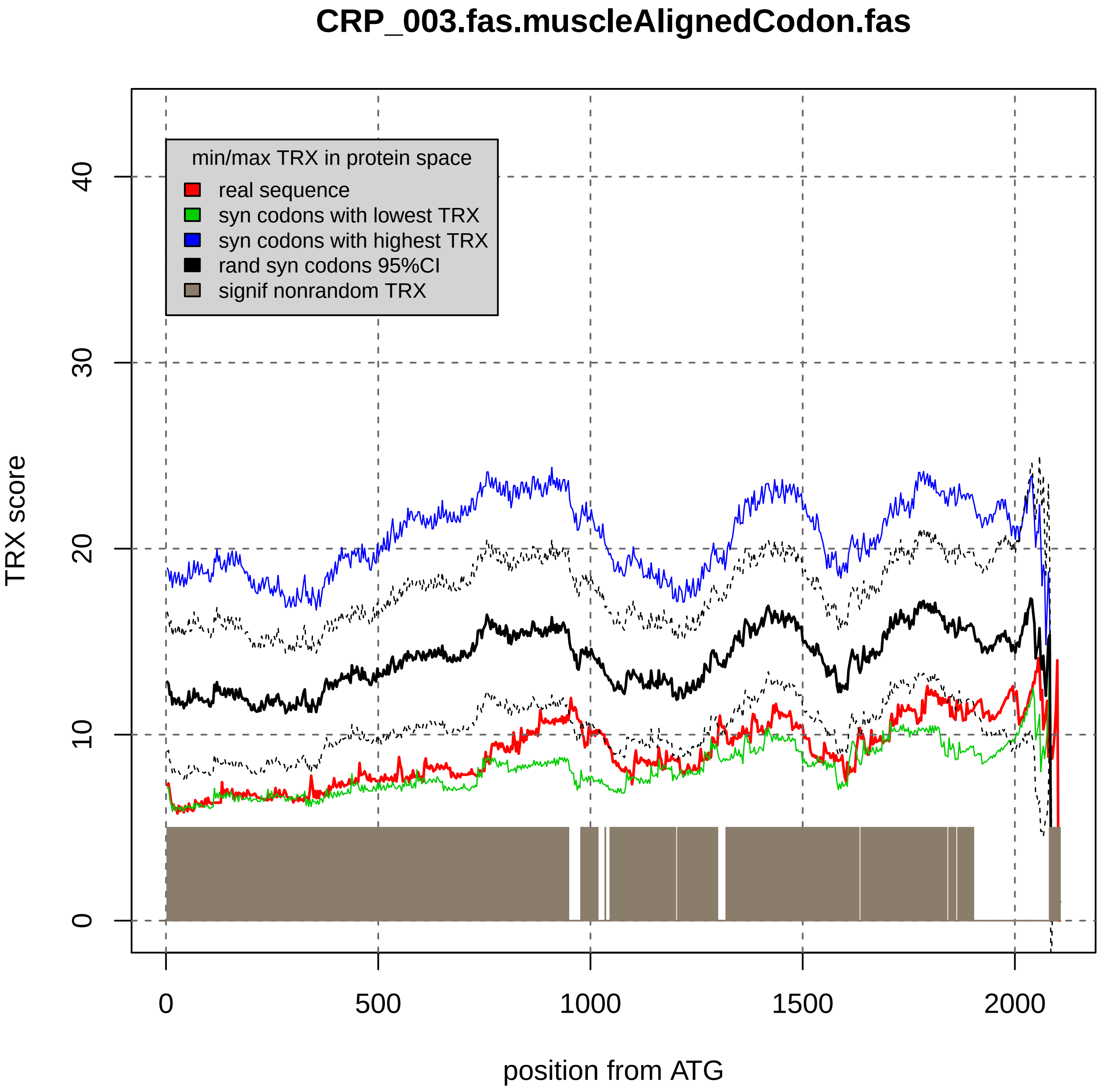
CRP_003.fas.muscleAlignedCodon.fas
min/max TRX in protein space
real sequence
syn codons with lowest TRX
syn codons with highest TRX
rand syn codons 95%CI
signif nonrandom TRX
TRX score
position from ATG
0
10
20
30
40
0
500
1000
1500
2000

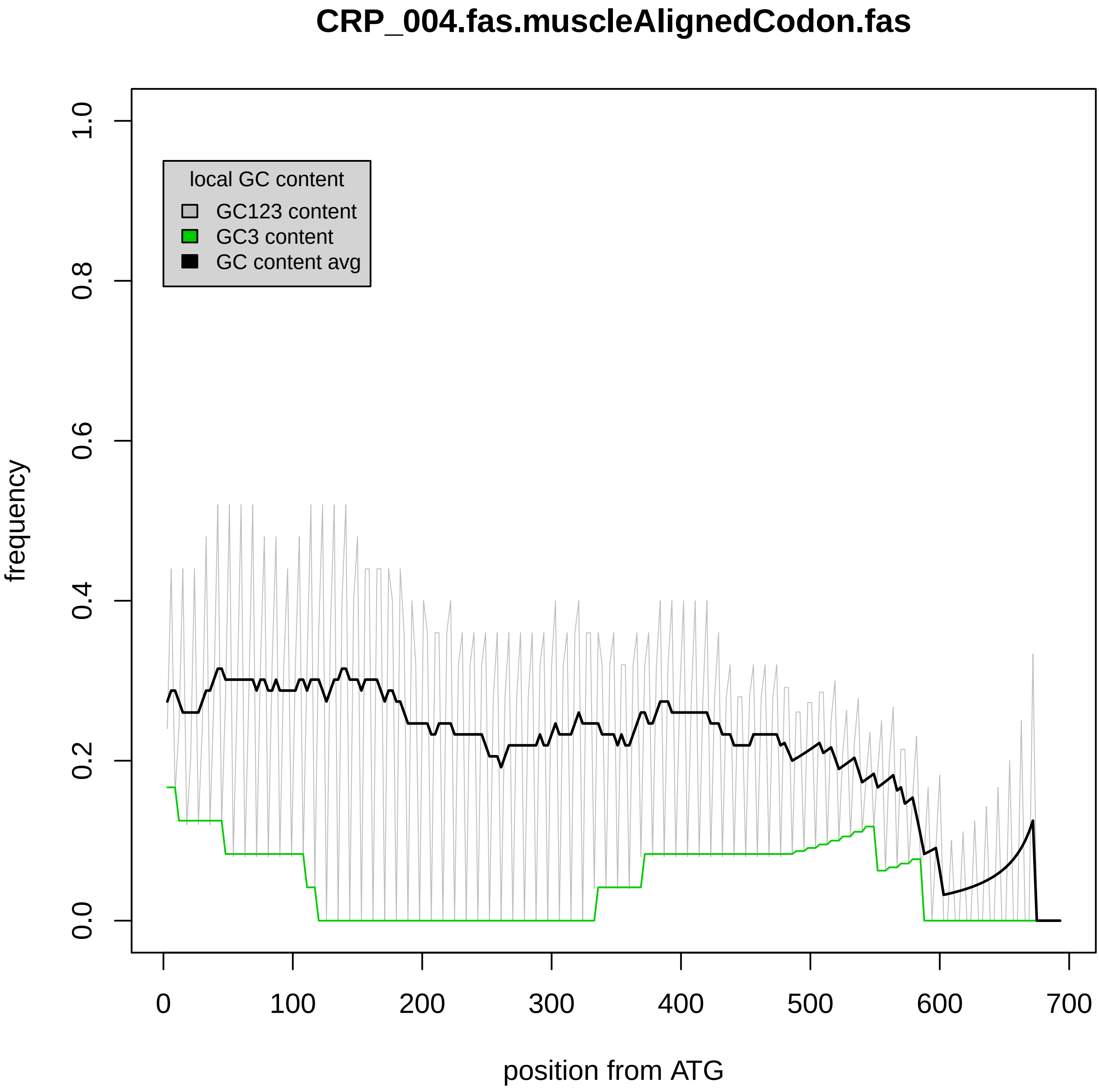
CRP_004.fas.muscleAlignedCodon.fas
local GC content
GC123 content
GC3 content
GC content avg
frequency
0.0
0.2
0.4
0.6
0.8
1.0
0
100
200
300
400
500
600
700
position from ATG

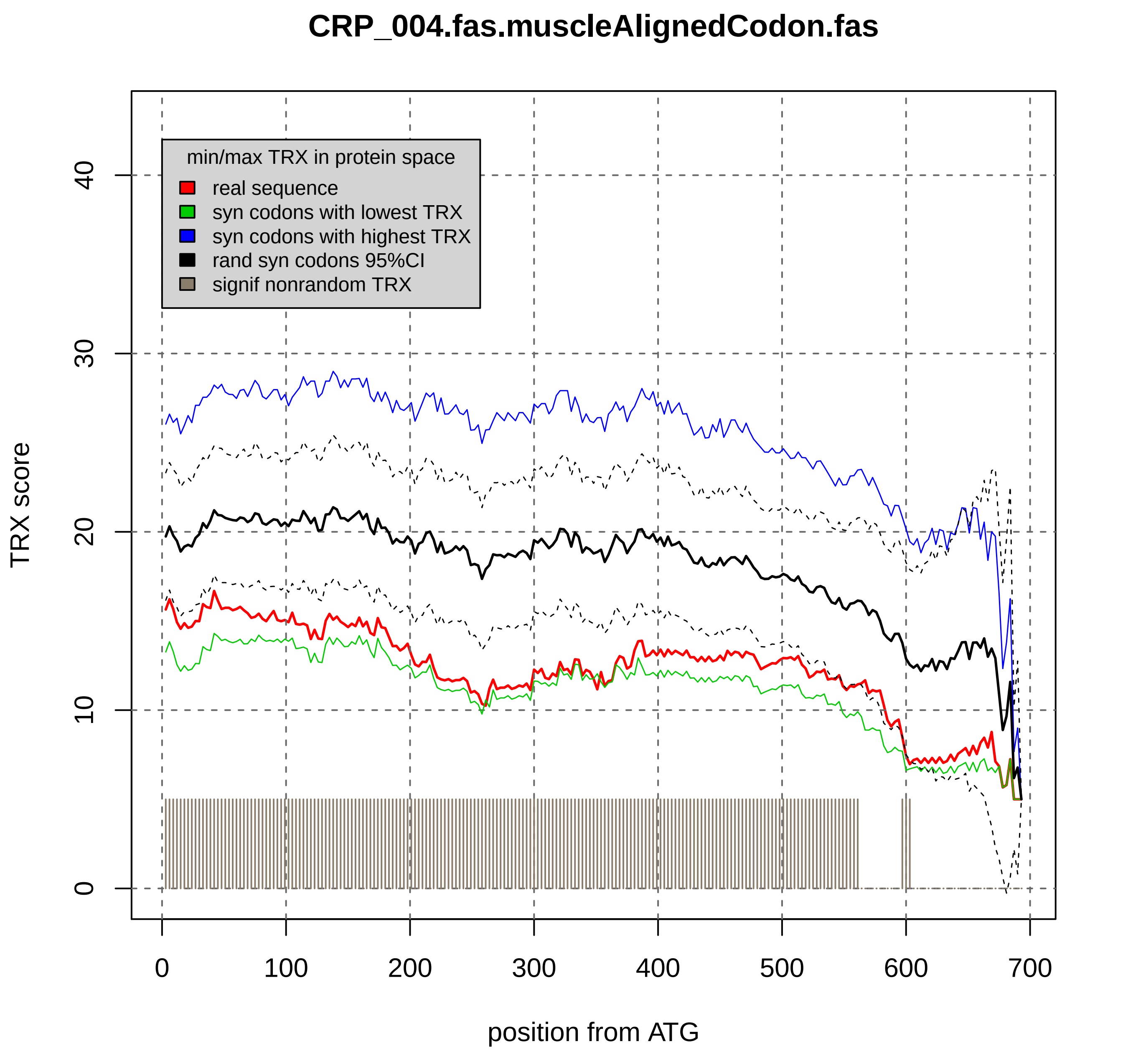

CRP_004.fas.muscleAlignedCodon.fas
min/max TRX in protein space
real sequence
syn codons with lowest TRX
syn codons with highest TRX
rand syn codons 95%CI
signif nonrandom TRX
TRX score
0
10
20
30
40
position from ATG
0
100
200
300
400
500
600
700

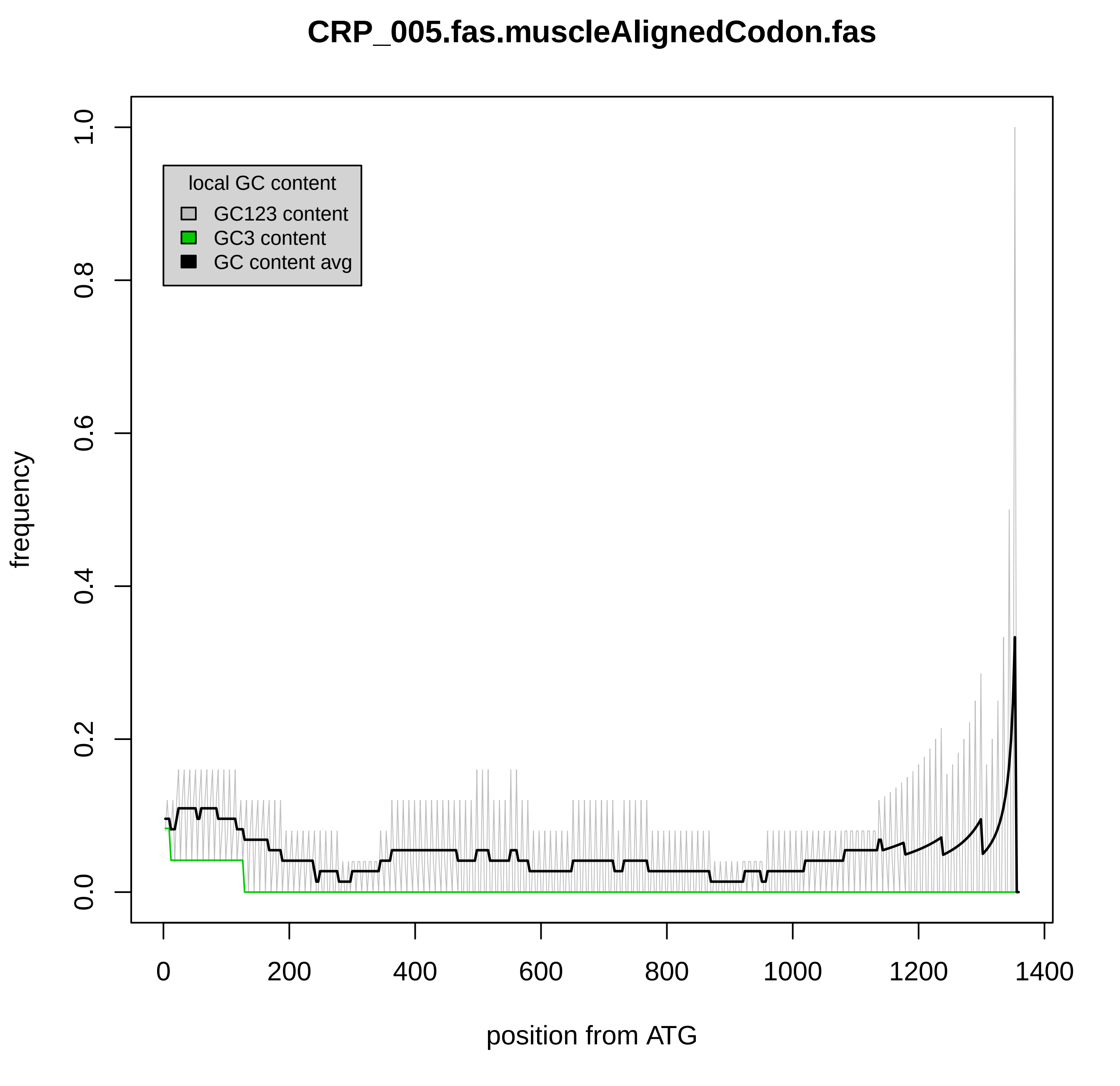

CRP_005.fas.muscleAlignedCodon.fas
local GC content
GC123 content
GC3 content
GC content avg
frequency
0.0
0.2
0.4
0.6
0.8
1.0
position from ATG
0
200
400
600
800
1000
1200
1400

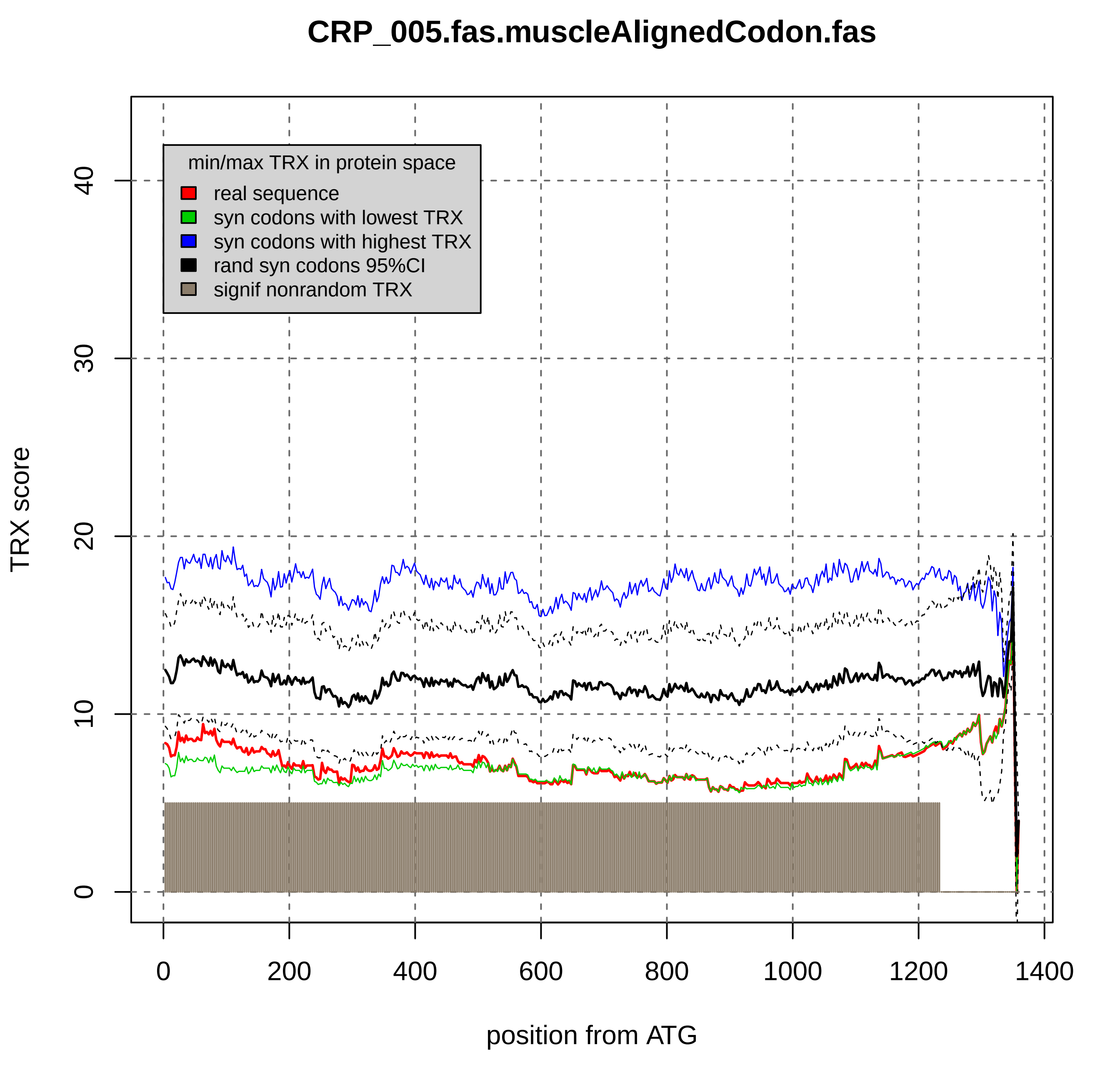
CRP_005.fas.muscleAlignedCodon.fas
min/max TRX in protein space
real sequence
syn codons with lowest TRX
syn codons with highest TRX
rand syn codons 95%CI
signif nonrandom TRX
TRX score
position from ATG
0
10
20
30
40
0
200
400
600
800
1000
1200
1400

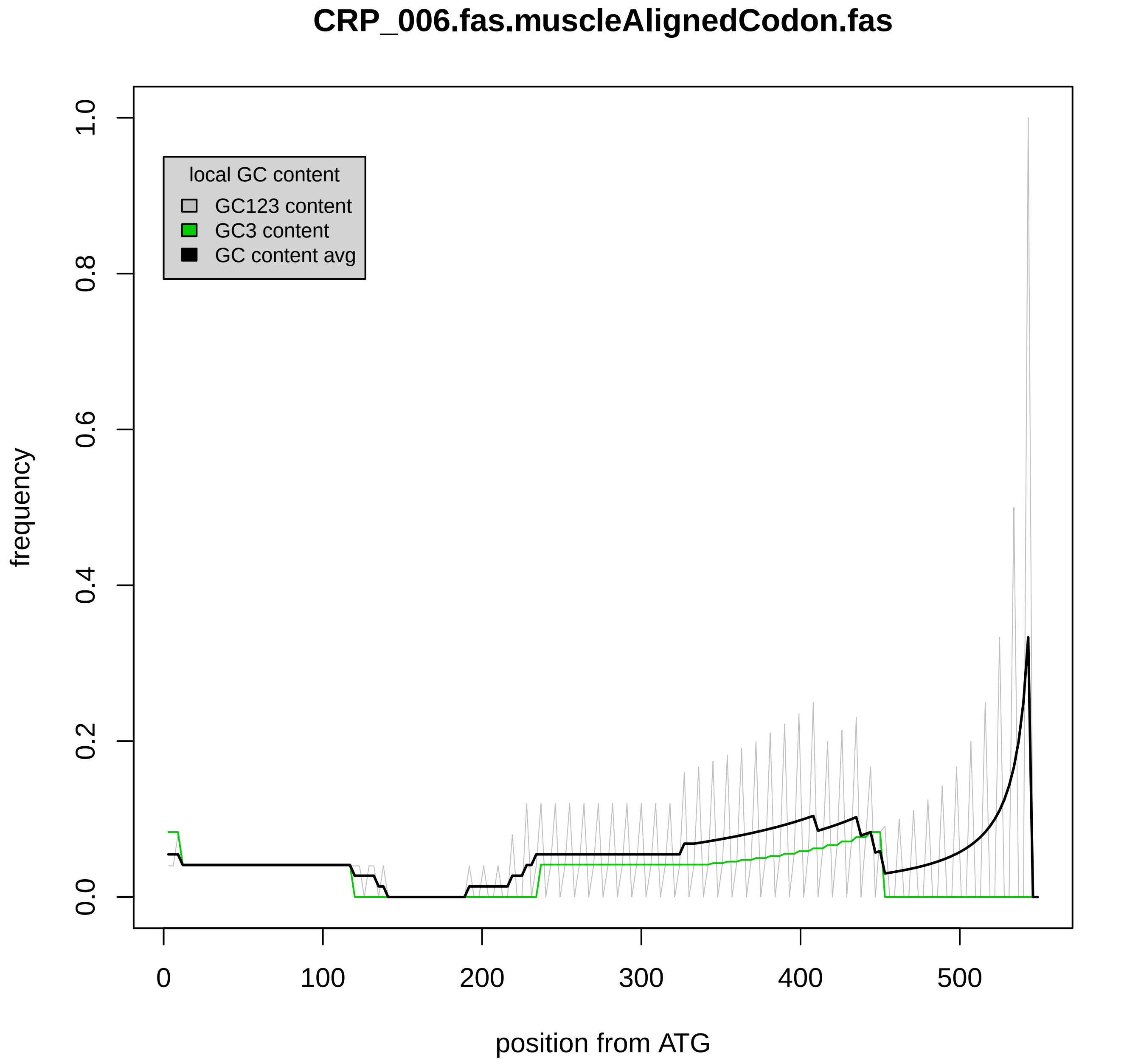
CRP_006.fas.muscleAlignedCodon.fas
local GC content
GC123 content
GC3 content
GC content avg
frequency
0.0
0.2
0.4
0.6
0.8
1.0
0
100
200
300
400
500
position from ATG

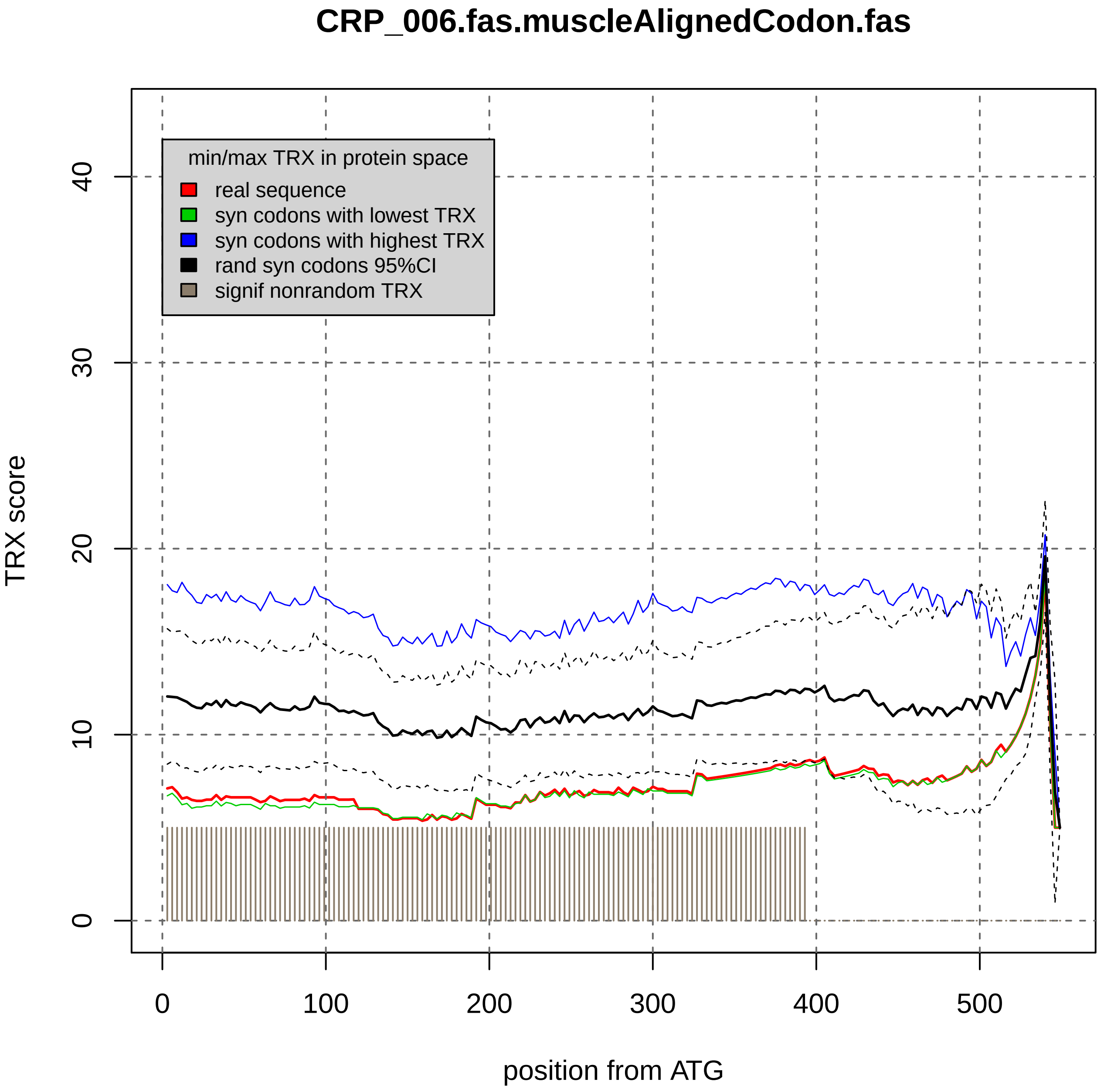

CRP_006.fas.muscleAlignedCodon.fas
min/max TRX in protein space
real sequence
syn codons with lowest TRX
syn codons with highest TRX
rand syn codons 95%CI
signif nonrandom TRX
TRX score
0
10
20
30
40
0
100
200
300
400
500
position from ATG

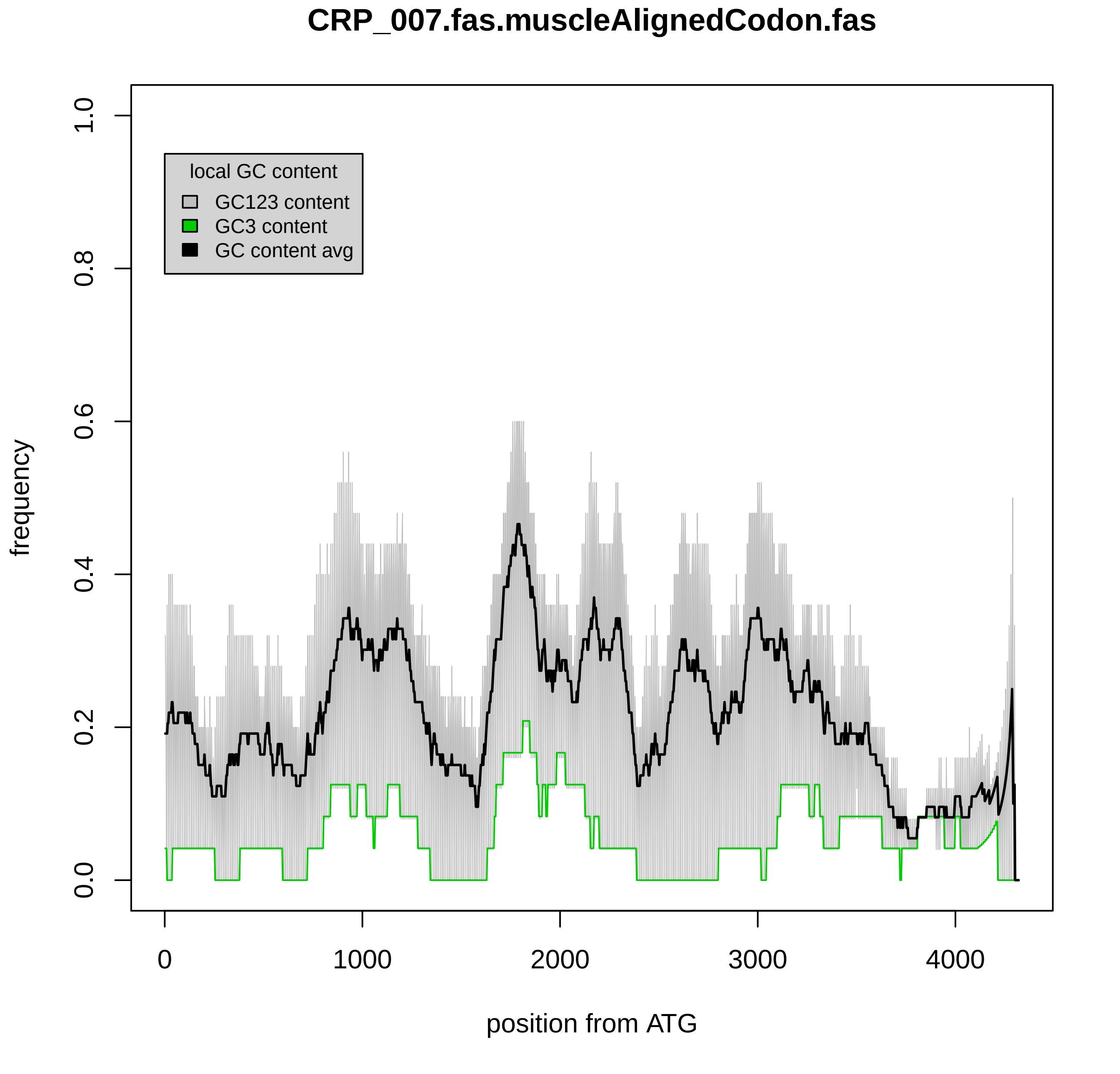
CRP_007.fas.muscleAlignedCodon.fas
local GC content
GC123 content
GC3 content
GC content avg
frequency
0.0
0.2
0.4
0.6
0.8
1.0
0
1000
2000
3000
4000
position from ATG

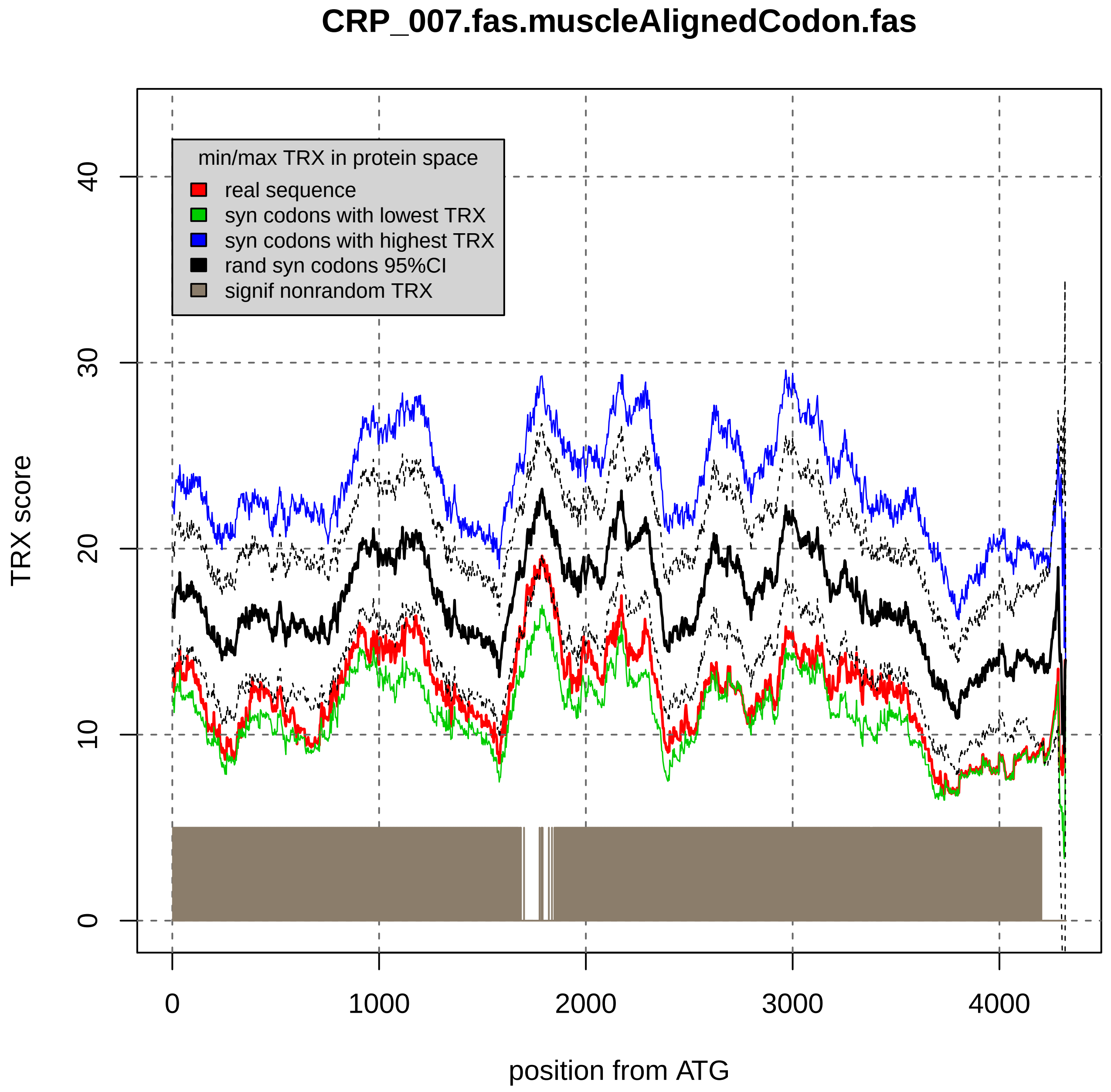

CRP_007.fas.muscleAlignedCodon.fas
min/max TRX in protein space
real sequence
syn codons with lowest TRX
syn codons with highest TRX
rand syn codons 95%CI
signif nonrandom TRX
TRX score
position from ATG
0
10
20
30
40
0
1000
2000
3000
4000

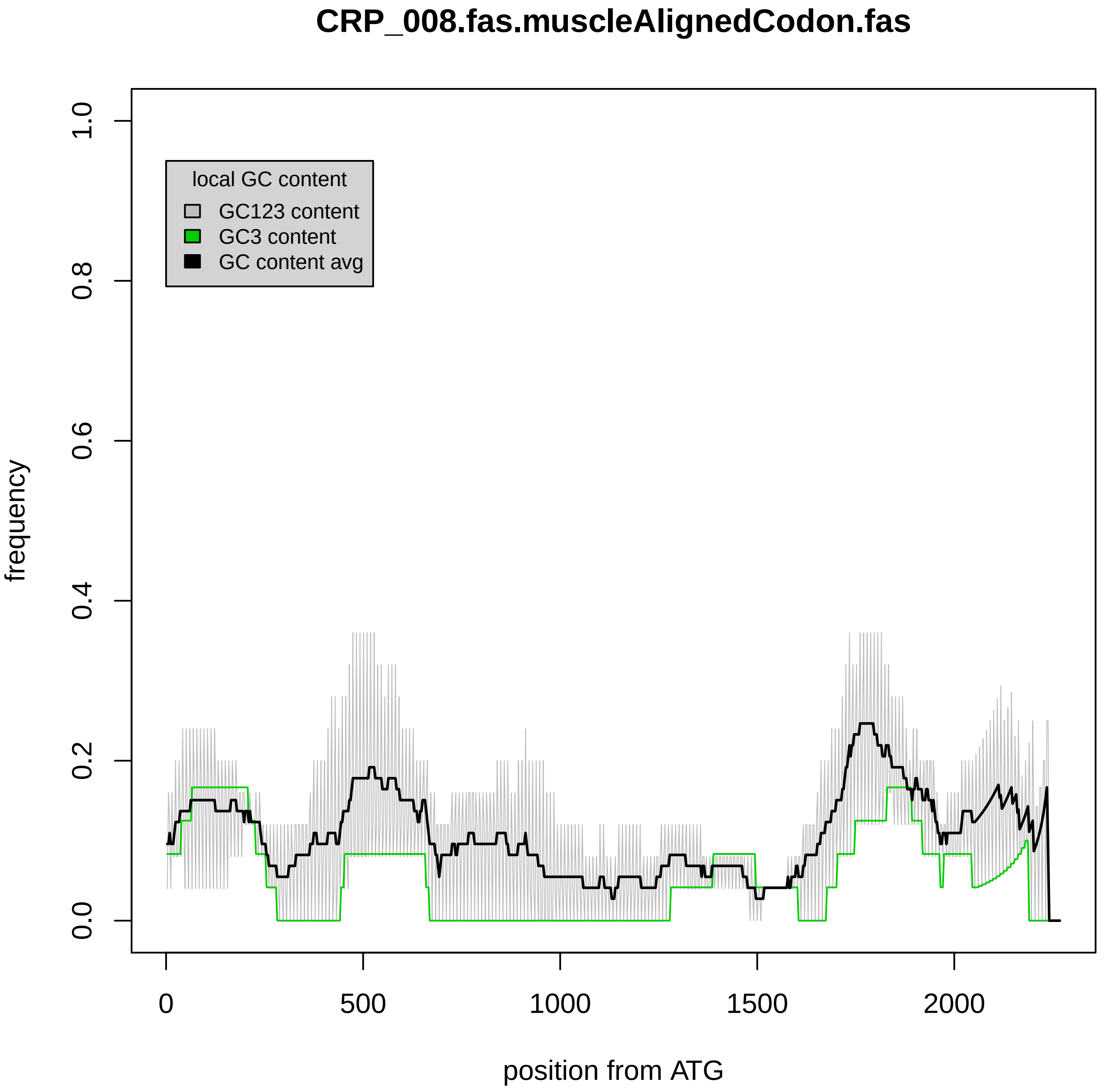

CRP_008.fas.muscleAlignedCodon.fas
local GC content
GC123 content
GC3 content
GC content avg
frequency
0.0
0.2
0.4
0.6
0.8
1.0
0
500
1000
1500
2000
position from ATG

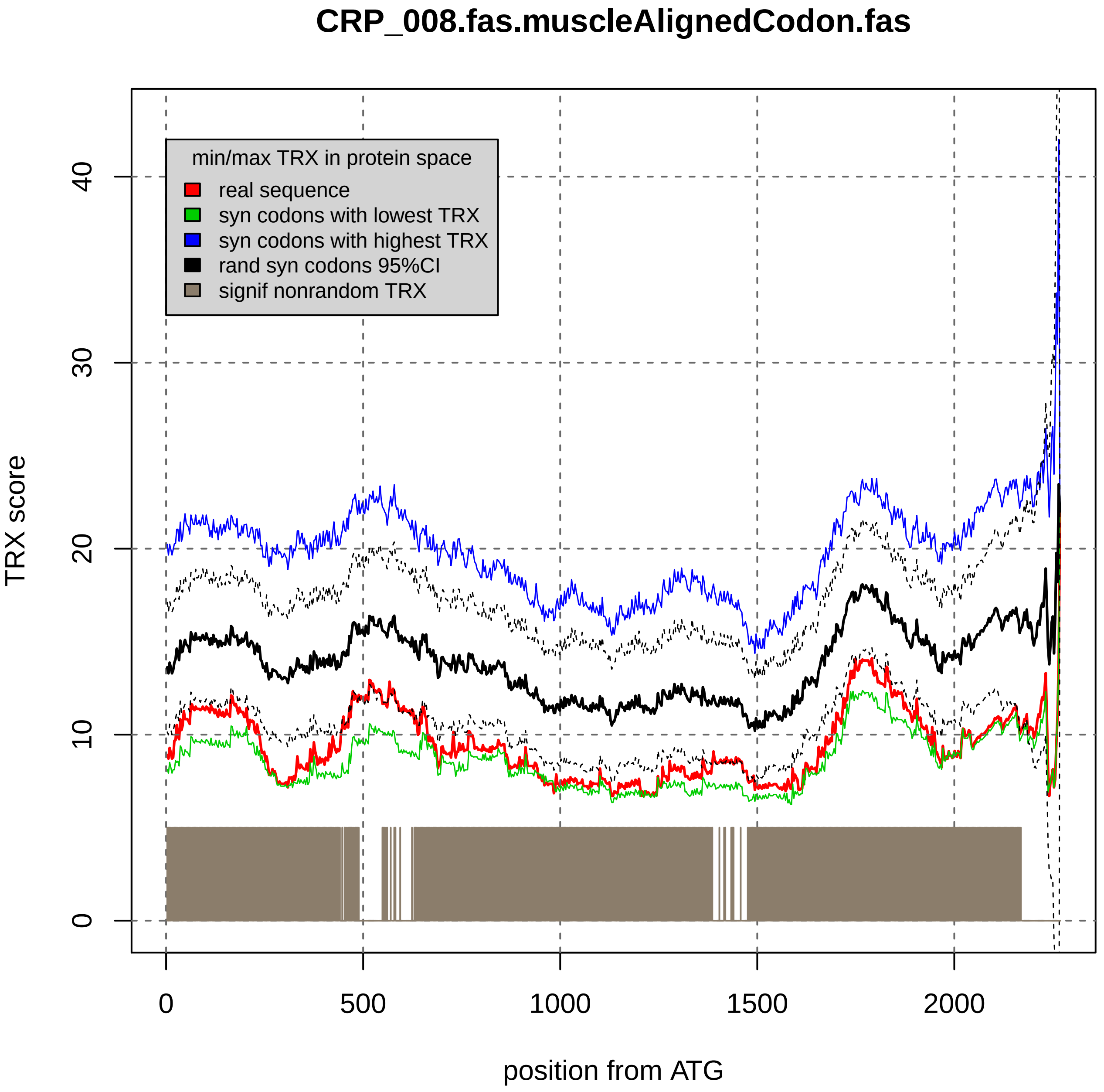

CRP_008.fas.muscleAlignedCodon.fas
min/max TRX in protein space
real sequence
syn codons with lowest TRX
syn codons with highest TRX
rand syn codons 95%CI
signif nonrandom TRX
TRX score
0
10
20
30
40
0
500
1000
1500
2000
position from ATG

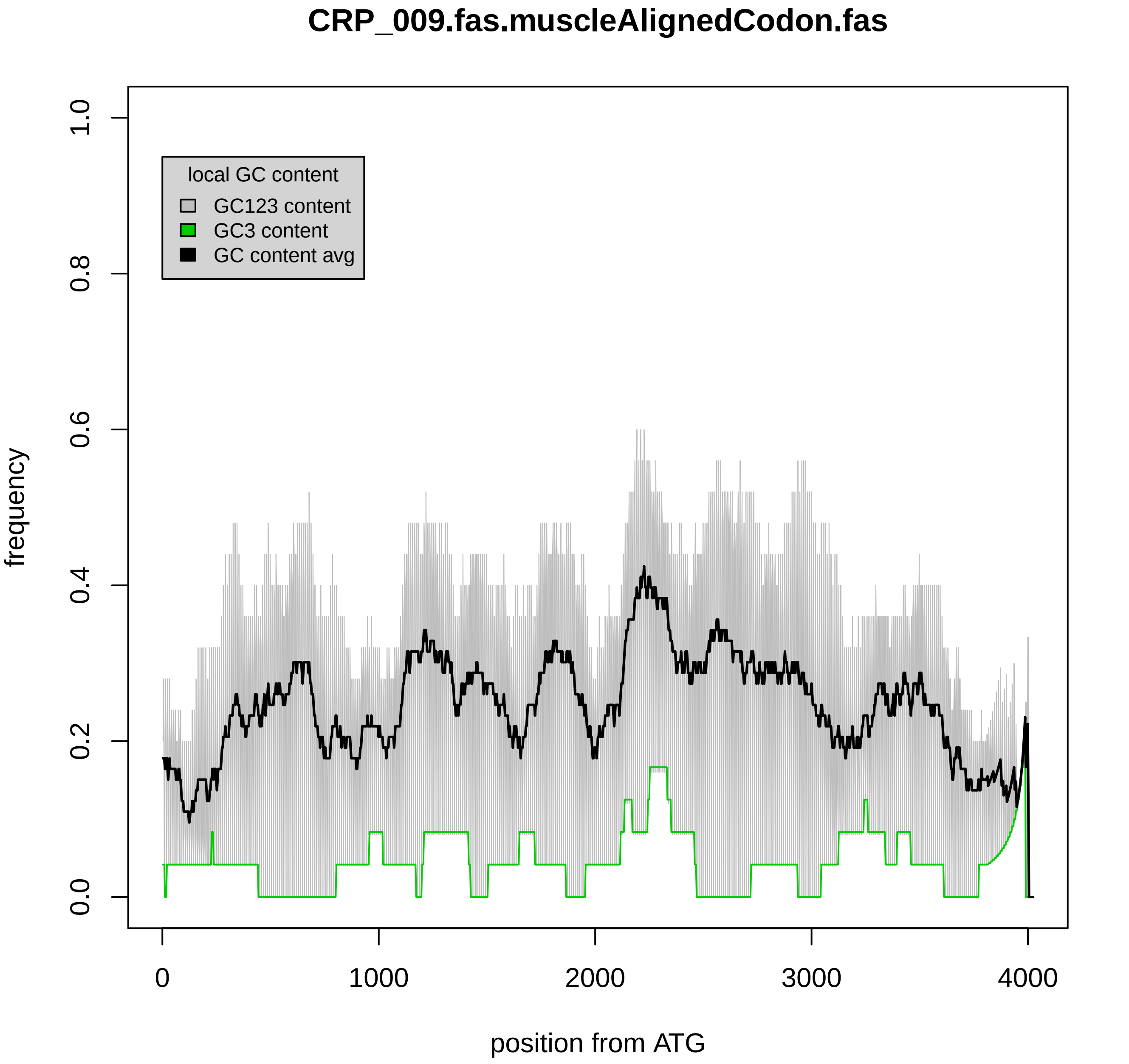
CRP_009.fas.muscleAlignedCodon.fas
local GC content
GC123 content
GC3 content
GC content avg
frequency
0.0
0.2
0.4
0.6
0.8
1.0
0
1000
2000
3000
4000
position from ATG

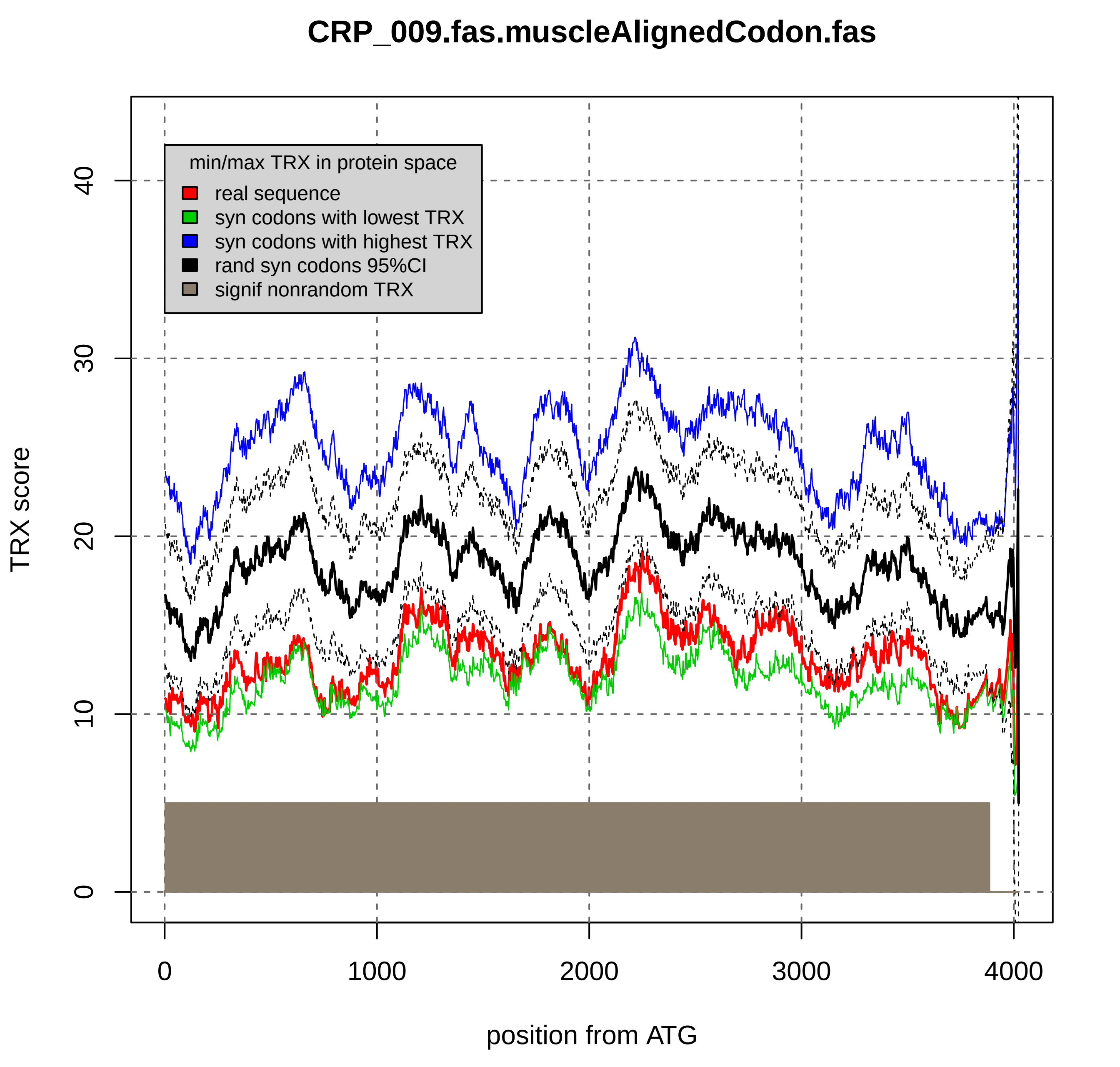
CRP_009.fas.muscleAlignedCodon.fas
min/max TRX in protein space
real sequence
syn codons with lowest TRX
syn codons with highest TRX
rand syn codons 95%CI
signif nonrandom TRX
TRX score
position from ATG
0
10
20
30
40
0
1000
2000
3000
4000

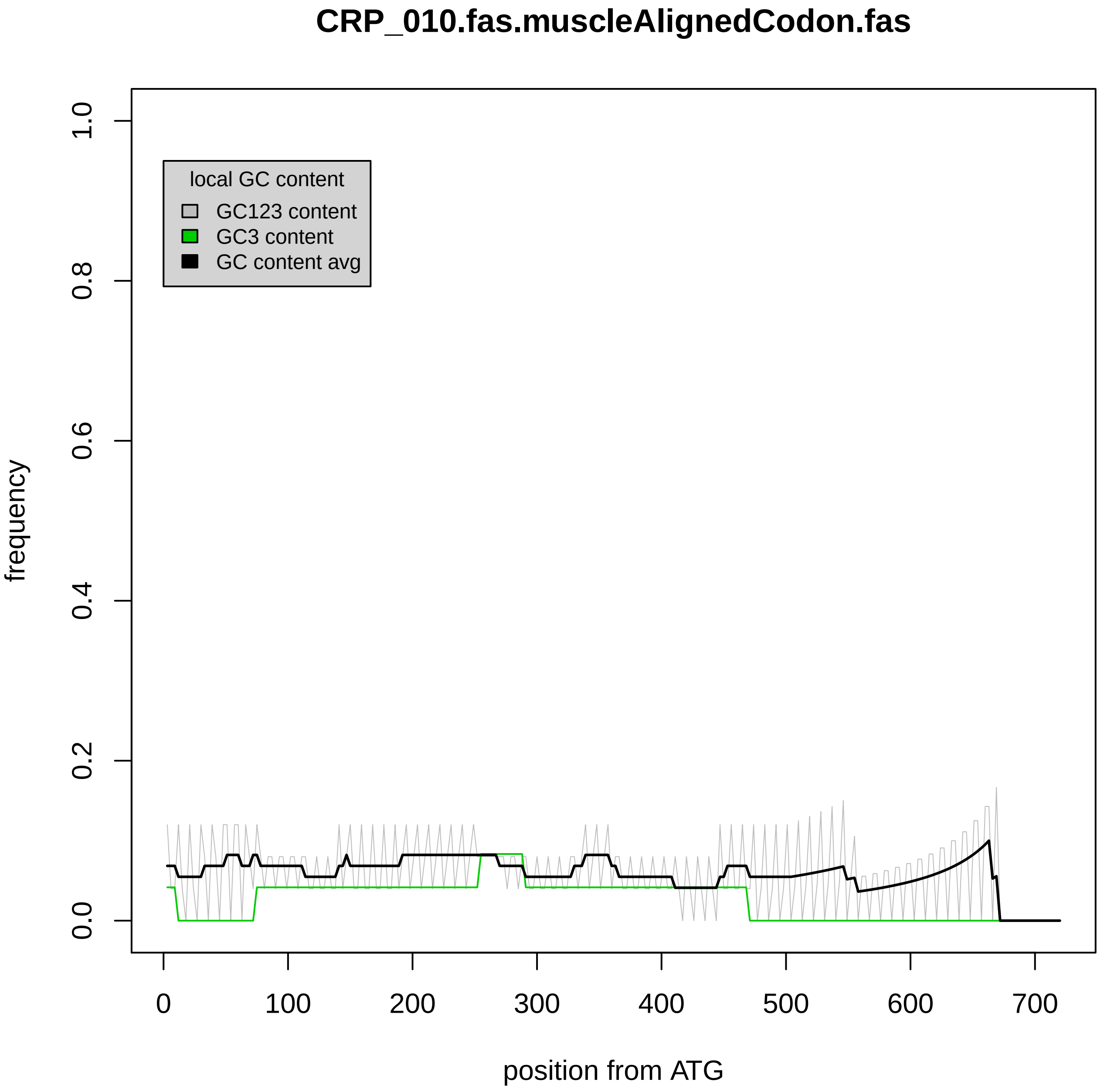
CRP_010.fas.muscleAlignedCodon.fas
local GC content
GC123 content
GC3 content
GC content avg
frequency
0.0
0.2
0.4
0.6
0.8
1.0
position from ATG
0
100
200
300
400
500
600
700

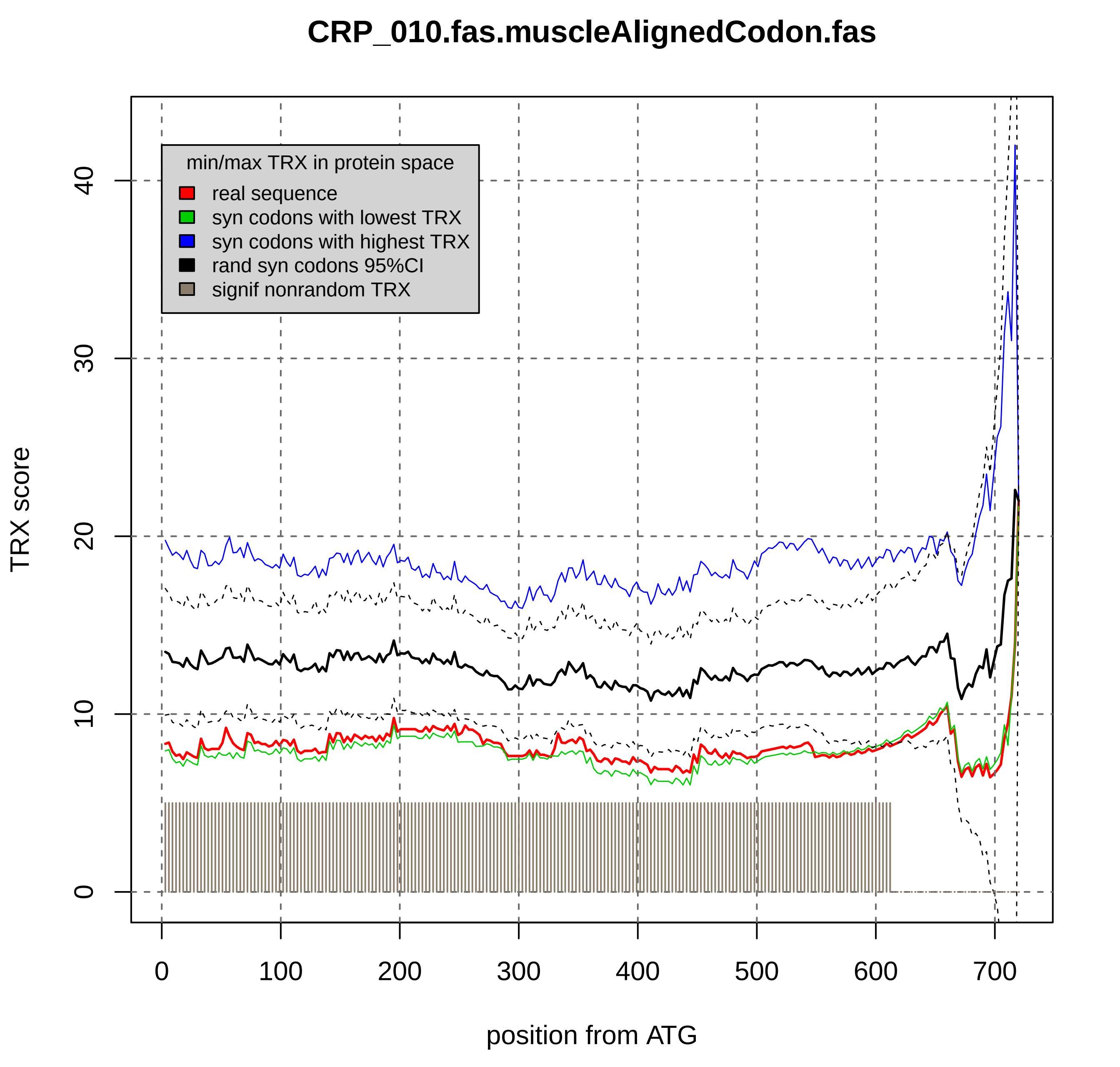
CRP_010.fas.muscleAlignedCodon.fas
min/max TRX in protein space
real sequence
syn codons with lowest TRX
syn codons with highest TRX
rand syn codons 95%CI
signif nonrandom TRX
TRX score
0
10
20
30
40
0
100
200
300
400
500
600
700
position from ATG

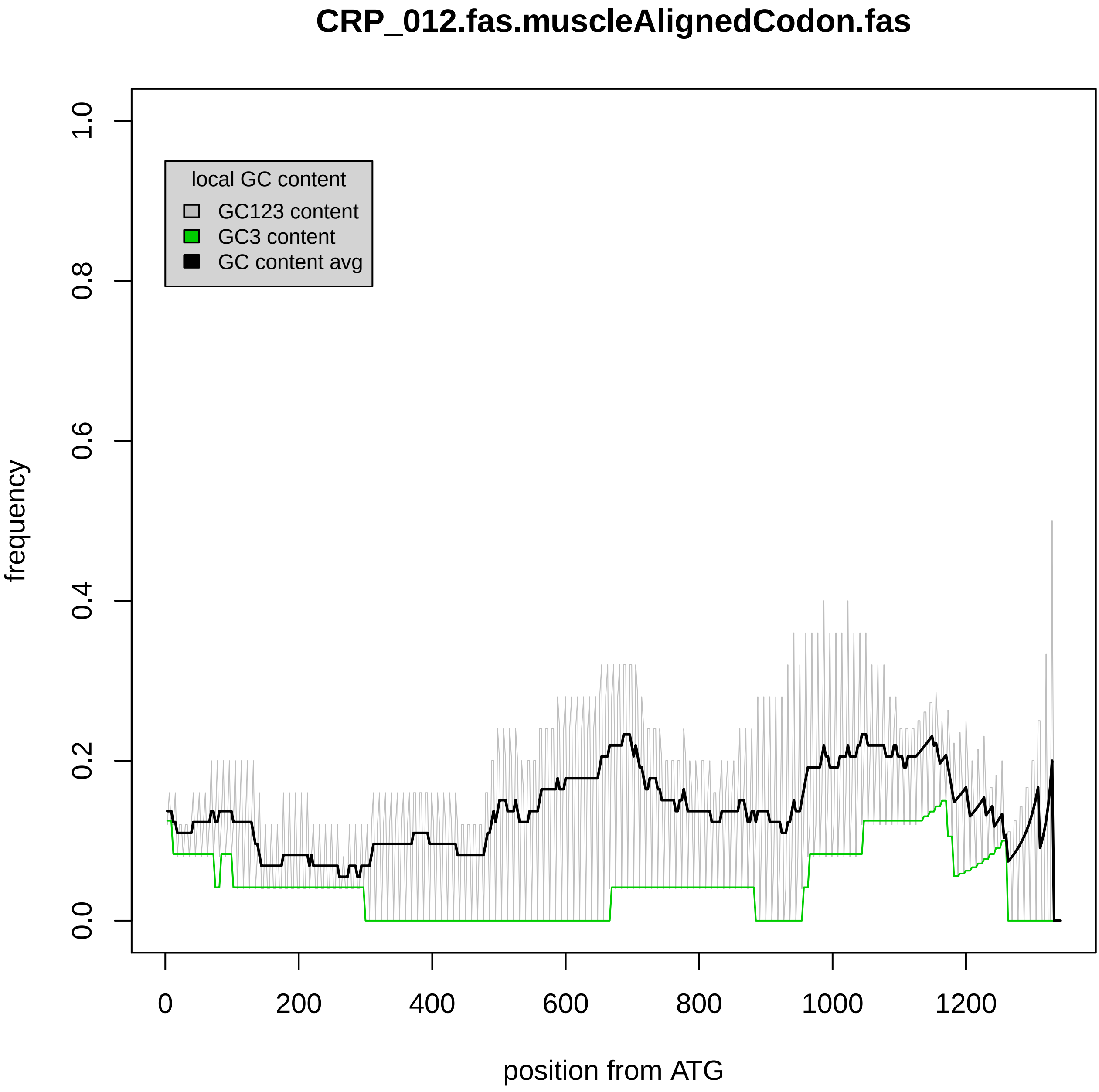

CRP_012.fas.muscleAlignedCodon.fas
local GC content
GC123 content
GC3 content
GC content avg
frequency
0.0
0.2
0.4
0.6
0.8
1.0
0
200
400
600
800
1000
1200
position from ATG

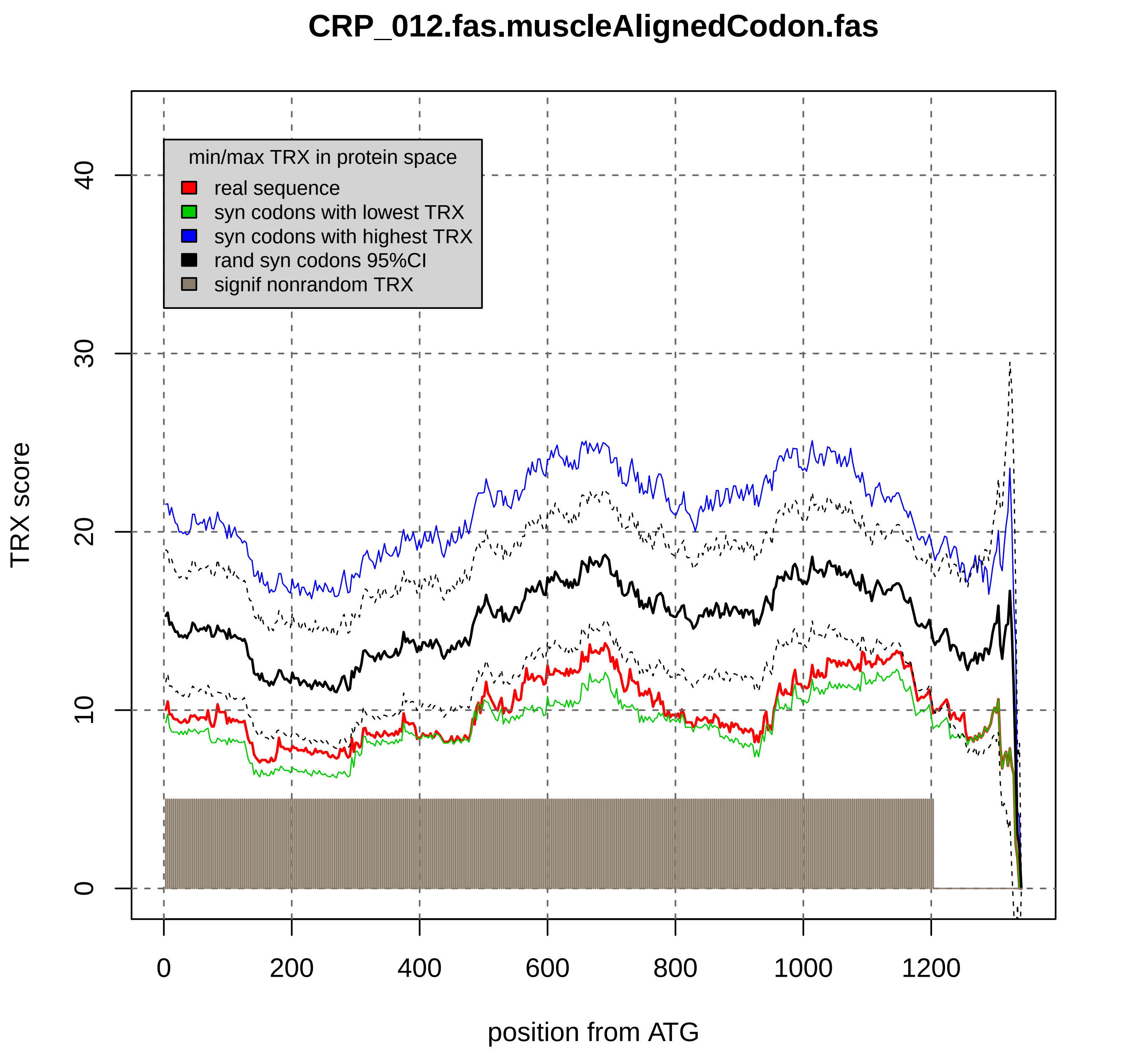

CRP_012.fas.muscleAlignedCodon.fas
min/max TRX in protein space
real sequence
syn codons with lowest TRX
syn codons with highest TRX
rand syn codons 95%CI
signif nonrandom TRX
TRX score
position from ATG
0
10
20
30
40
0
200
400
600
800
1000
1200

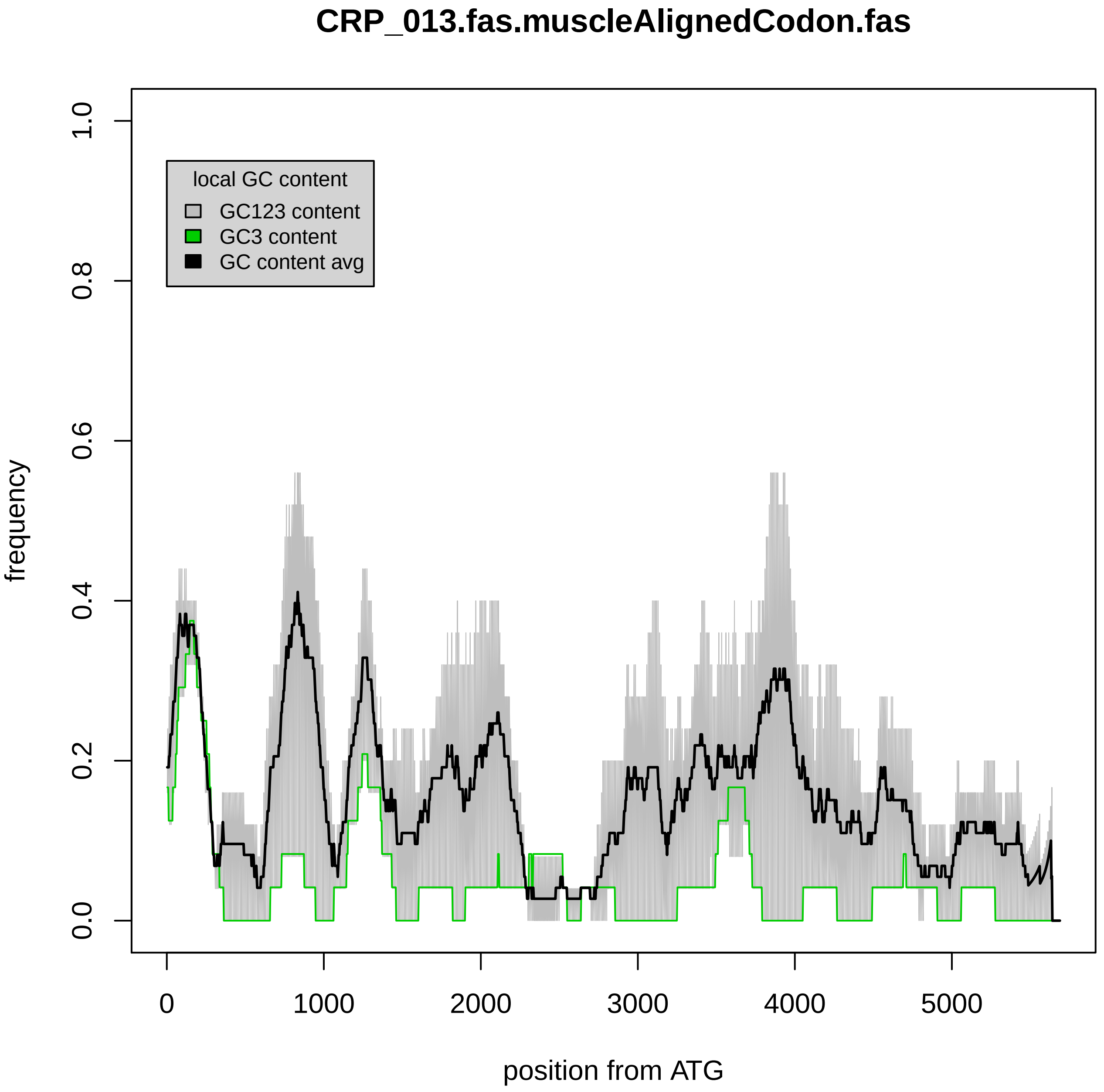
CRP_013.fas.muscleAlignedCodon.fas
local GC content
GC123 content
GC3 content
GC content avg
frequency
0.0
0.2
0.4
0.6
0.8
1.0
0
1000
2000
3000
4000
5000
position from ATG

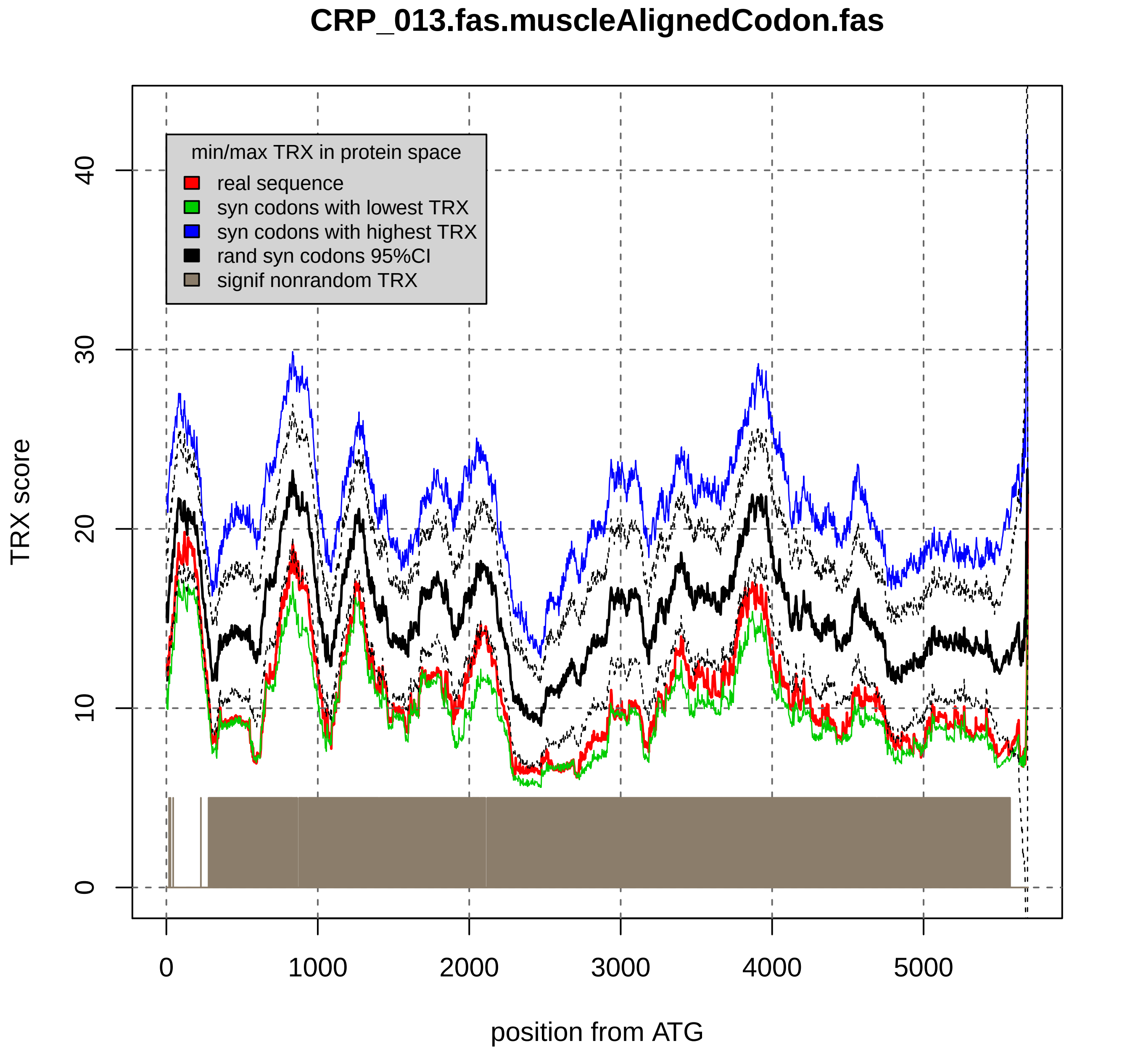

CRP_013.fas.muscleAlignedCodon.fas
min/max TRX in protein space
real sequence
syn codons with lowest TRX
syn codons with highest TRX
rand syn codons 95%CI
signif nonrandom TRX
TRX score
0
10
20
30
40
0
1000
2000
3000
4000
5000
position from ATG

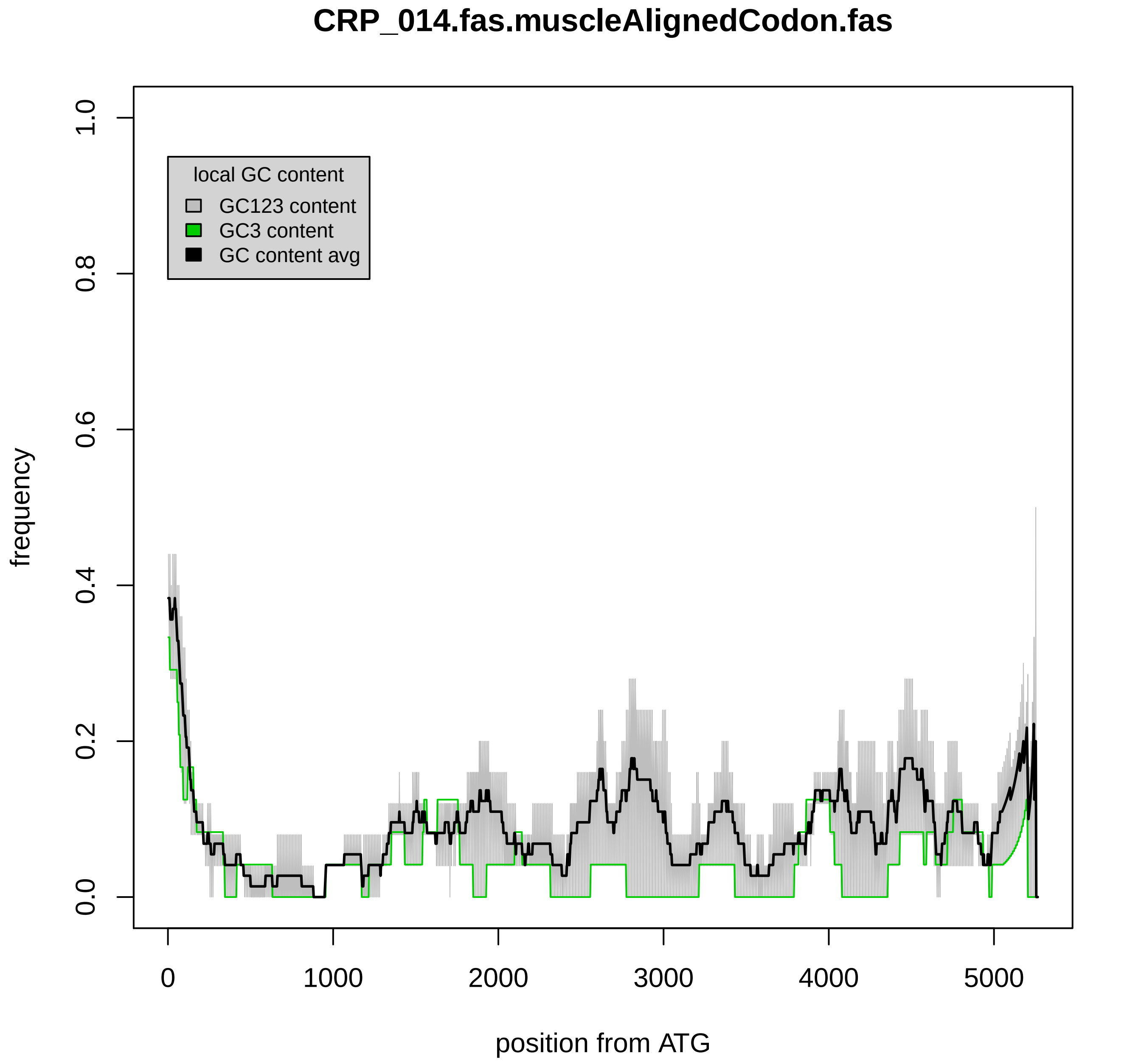
CRP_014.fas.muscleAlignedCodon.fas
local GC content
GC123 content
GC3 content
GC content avg
frequency
0.0
0.2
0.4
0.6
0.8
1.0
0
1000
2000
3000
4000
5000
position from ATG

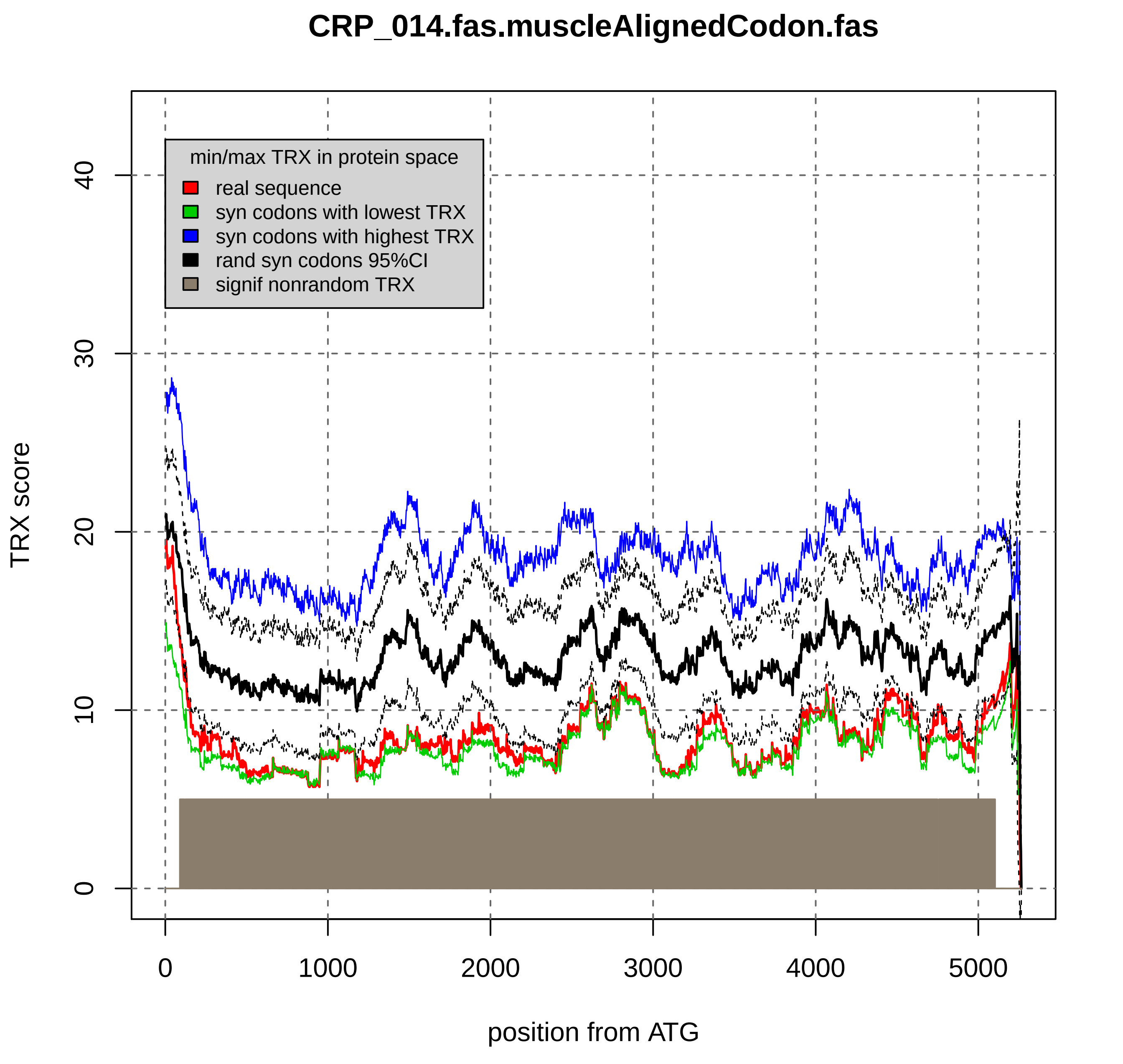

CRP_014.fas.muscleAlignedCodon.fas
min/max TRX in protein space
real sequence
syn codons with lowest TRX
syn codons with highest TRX
rand syn codons 95%CI
signif nonrandom TRX
TRX score
0
10
20
30
40
0
1000
2000
3000
4000
5000
position from ATG

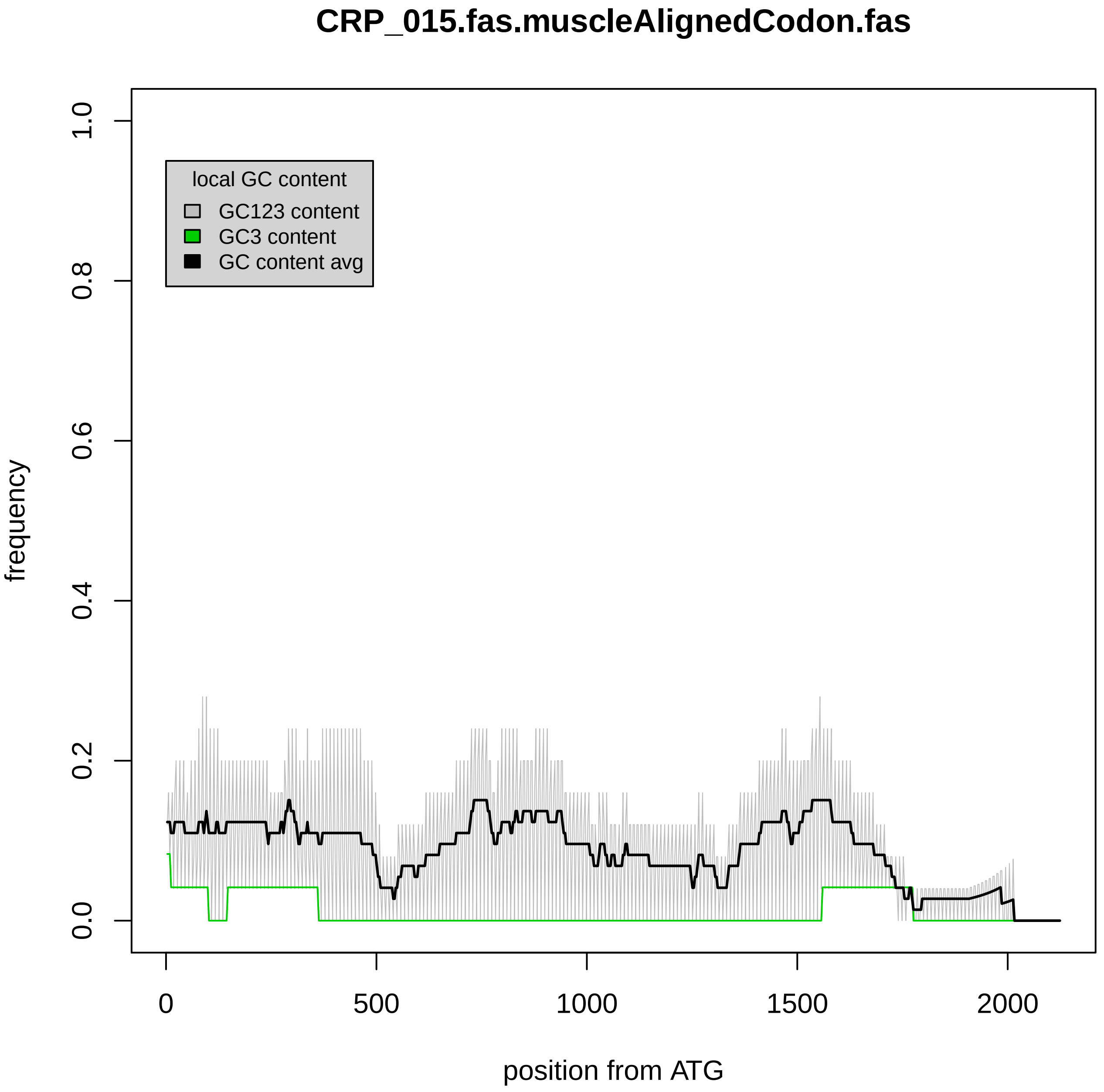
CRP_015.fas.muscleAlignedCodon.fas
local GC content
GC123 content
GC3 content
GC content avg
frequency
0.0
0.2
0.4
0.6
0.8
1.0
0
500
1000
1500
2000
position from ATG

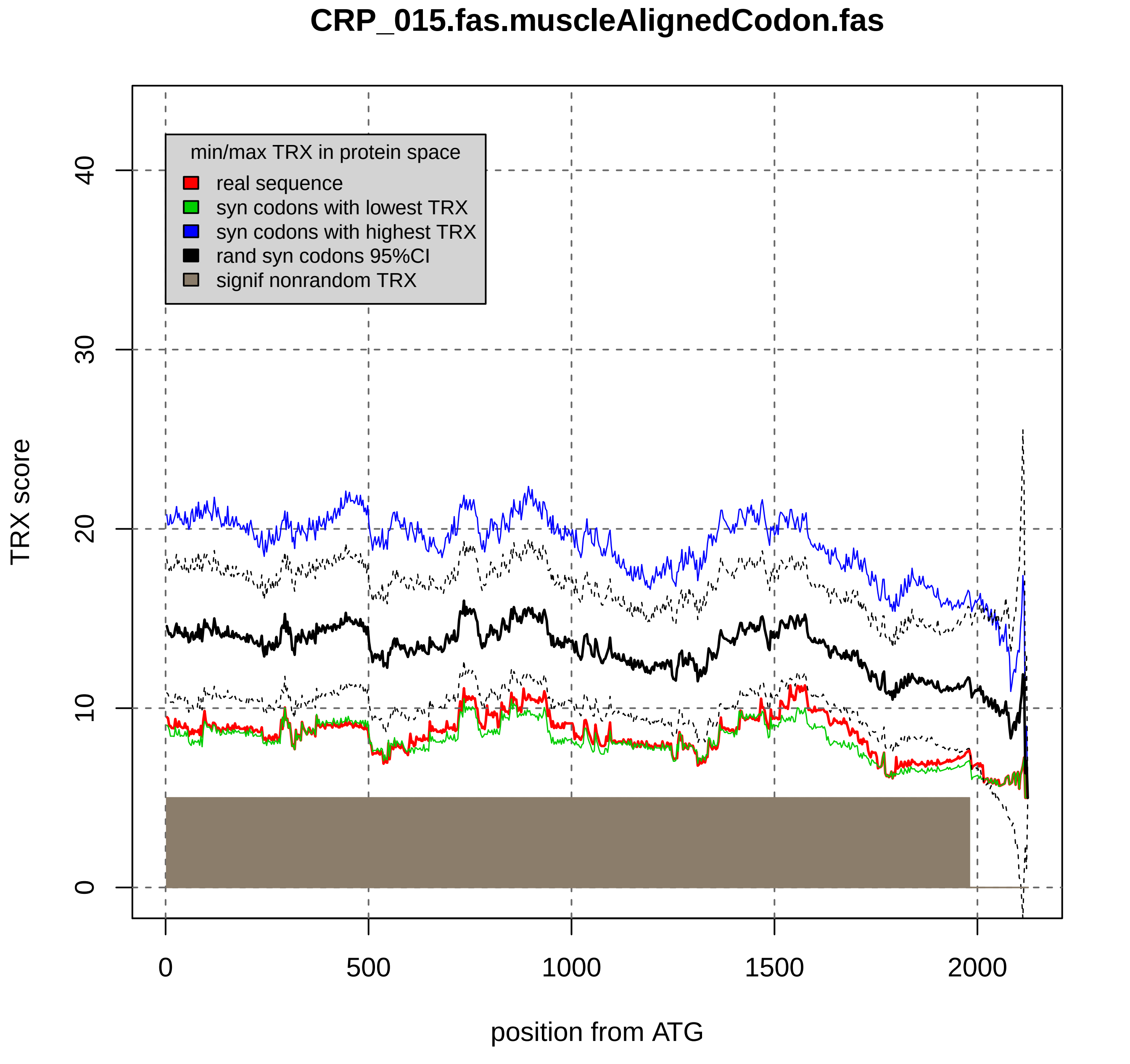

CRP_015.fas.muscleAlignedCodon.fas
min/max TRX in protein space
real sequence
syn codons with lowest TRX
syn codons with highest TRX
rand syn codons 95%CI
signif nonrandom TRX
TRX score
0
10
20
30
40
0
500
1000
1500
2000
position from ATG

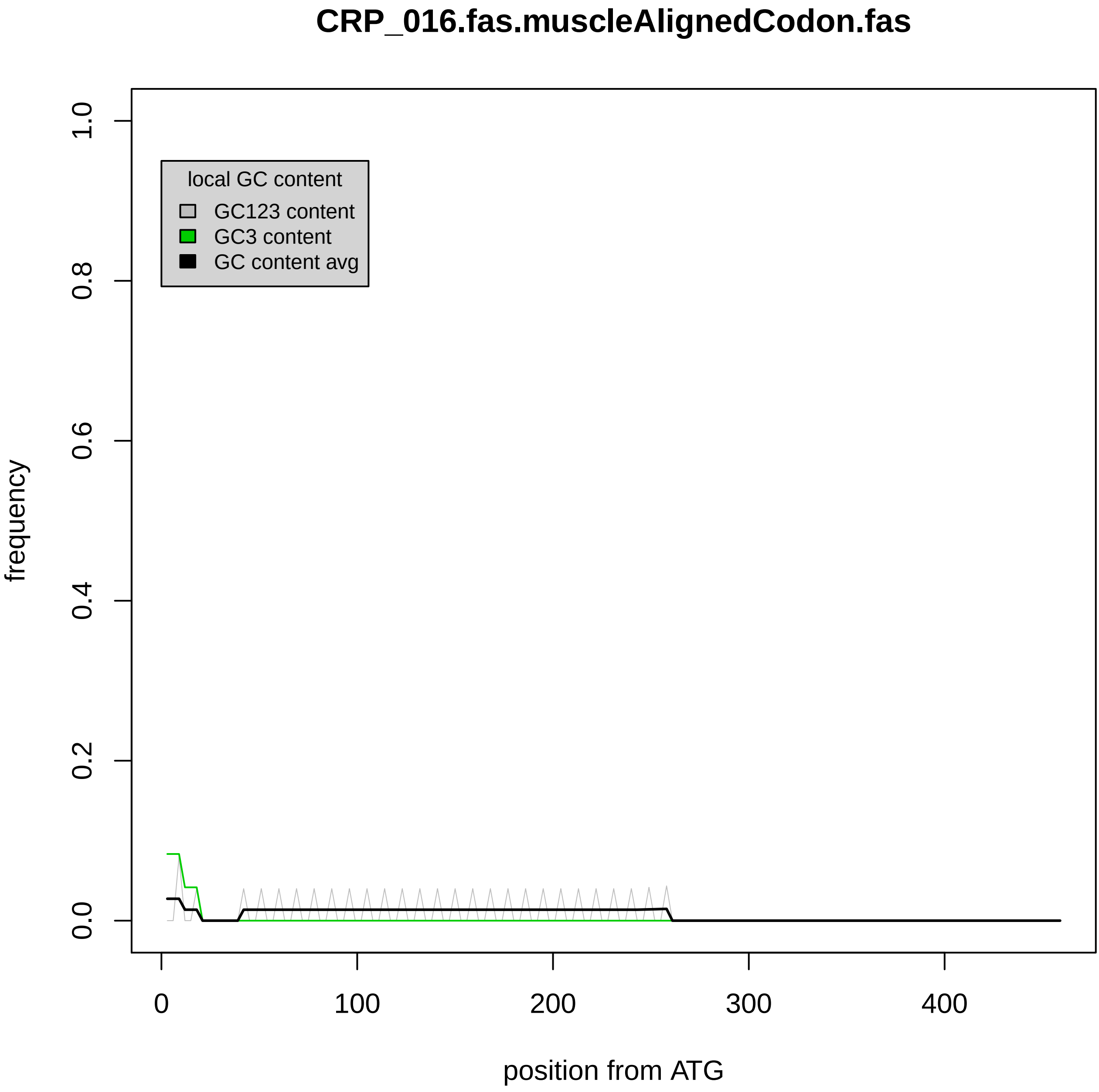

CRP_016.fas.muscleAlignedCodon.fas
local GC content
GC123 content
GC3 content
GC content avg
frequency
0.0
0.2
0.4
0.6
0.8
1.0
0
100
200
300
400
position from ATG

CRP_016.fas.muscleAlignedCodon.fas

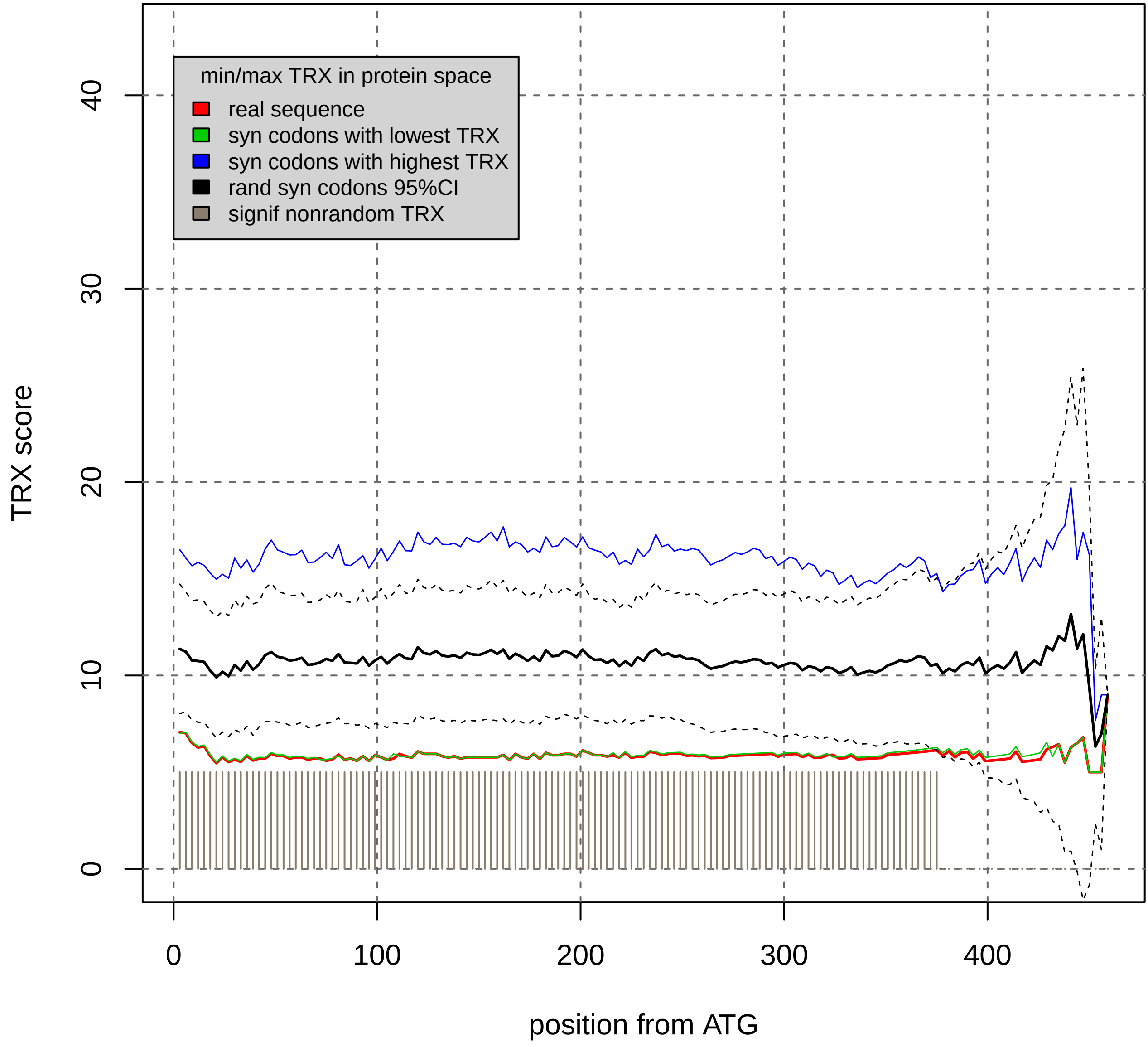

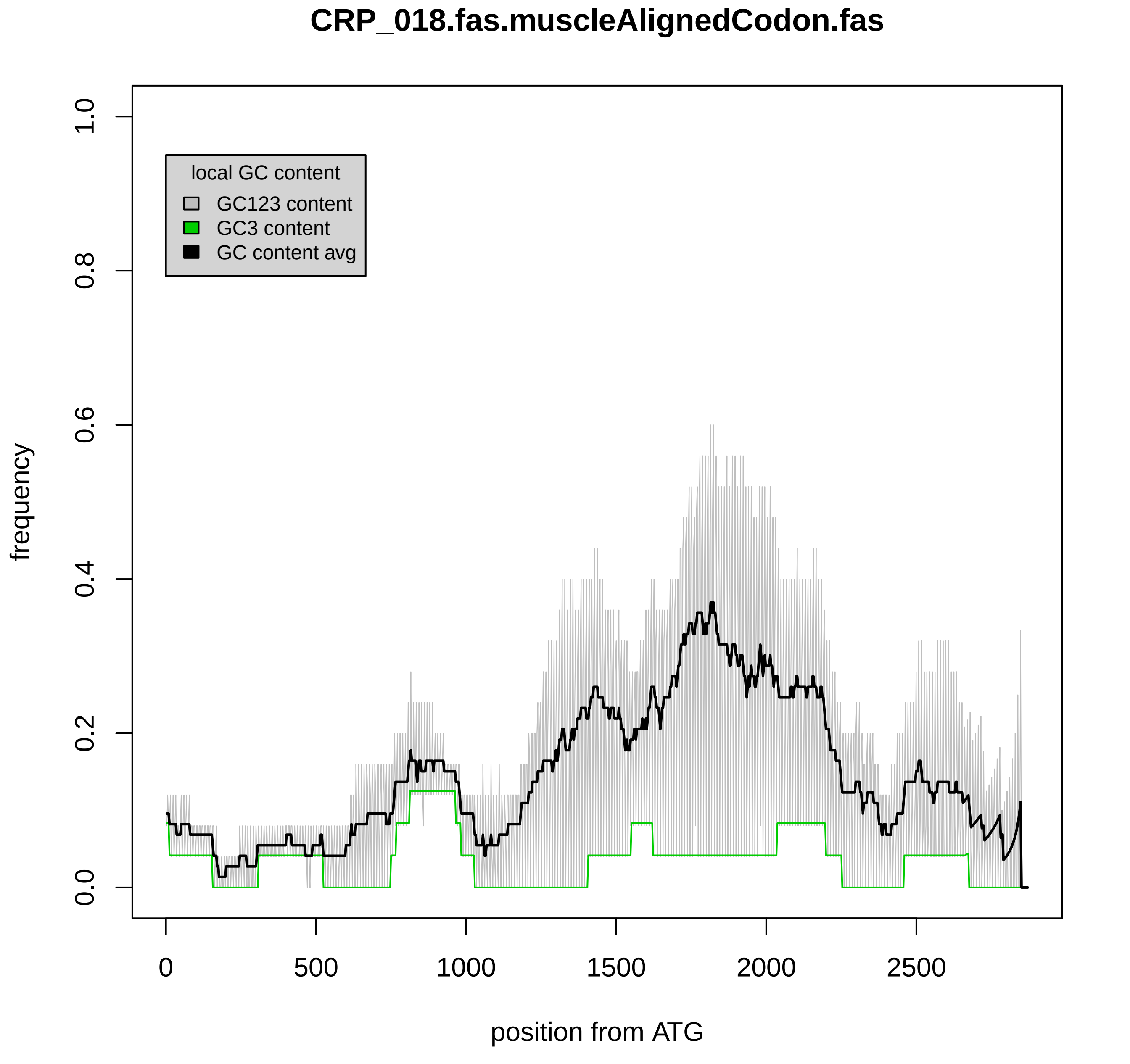
CRP_018.fas.muscleAlignedCodon.fas
local GC content
GC123 content
GC3 content
GC content avg
frequency
0.0
0.2
0.4
0.6
0.8
1.0
0
500
1000
1500
2000
2500
position from ATG

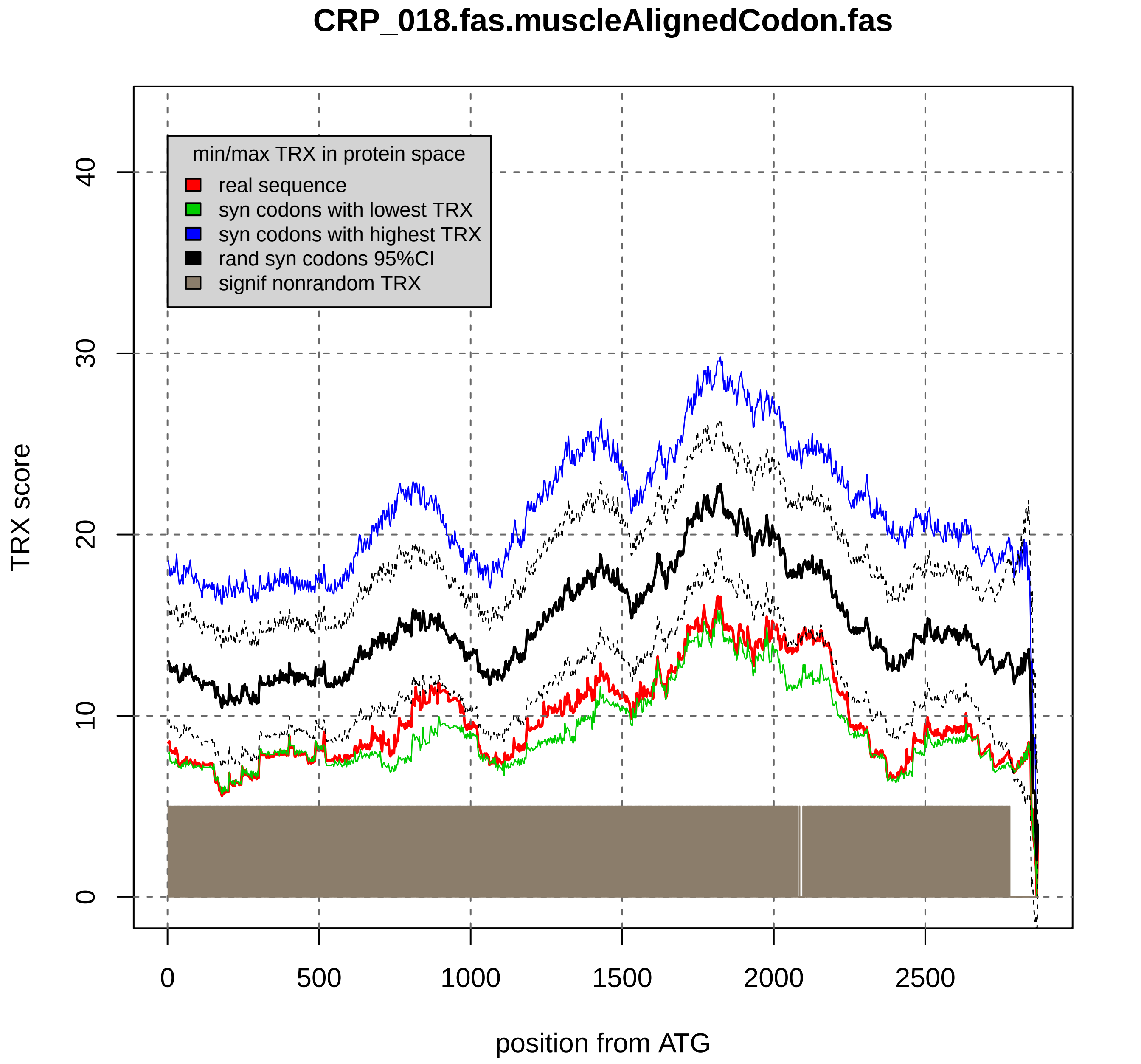

CRP_018.fas.muscleAlignedCodon.fas
min/max TRX in protein space
real sequence
syn codons with lowest TRX
syn codons with highest TRX
rand syn codons 95%CI
signif nonrandom TRX
TRX score
0
10
20
30
40
0
500
1000
1500
2000
2500
position from ATG

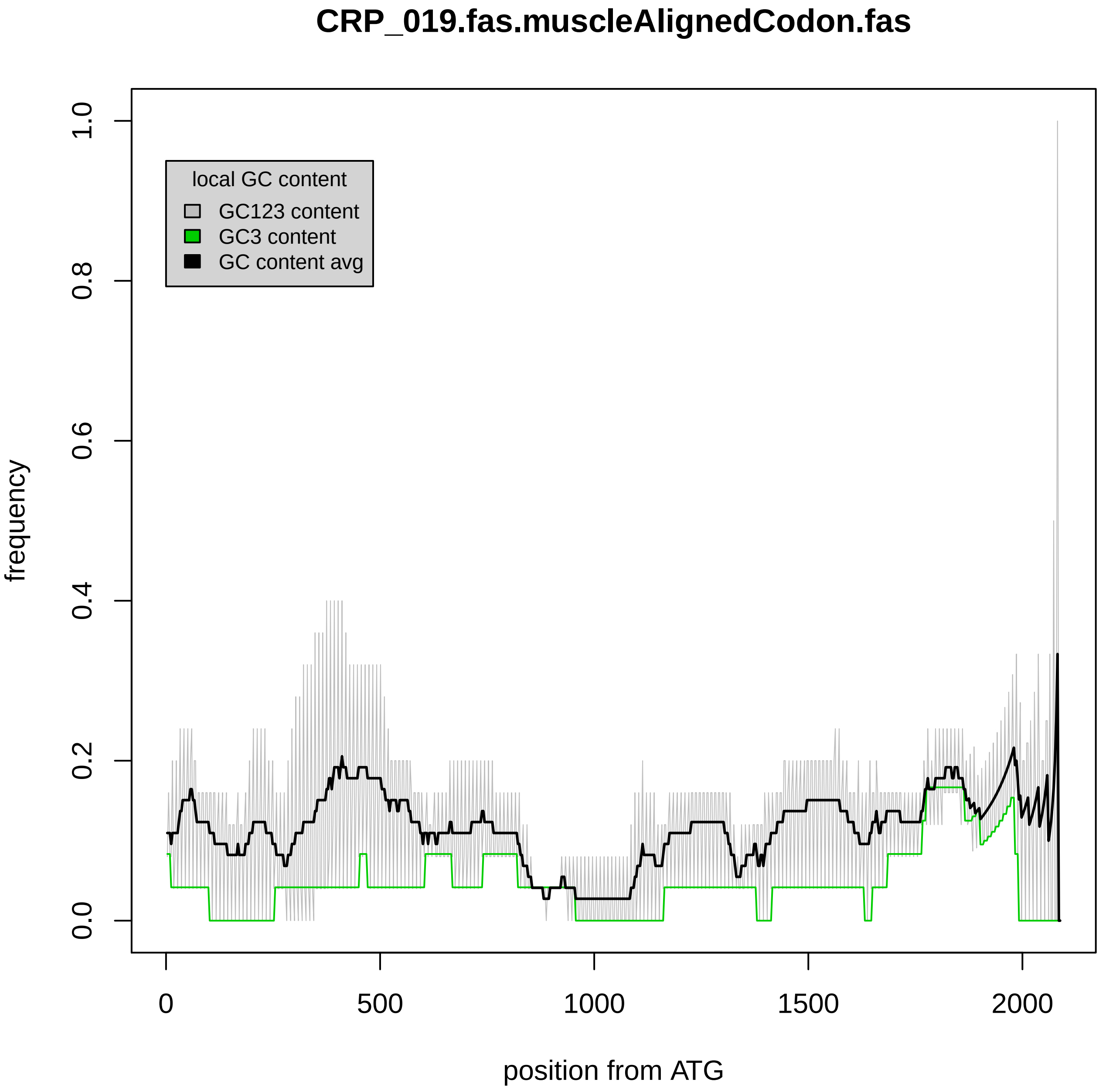

CRP_019.fas.muscleAlignedCodon.fas
local GC content
GC123 content
GC3 content
GC content avg
frequency
0.0
0.2
0.4
0.6
0.8
1.0
0
500
1000
1500
2000
position from ATG

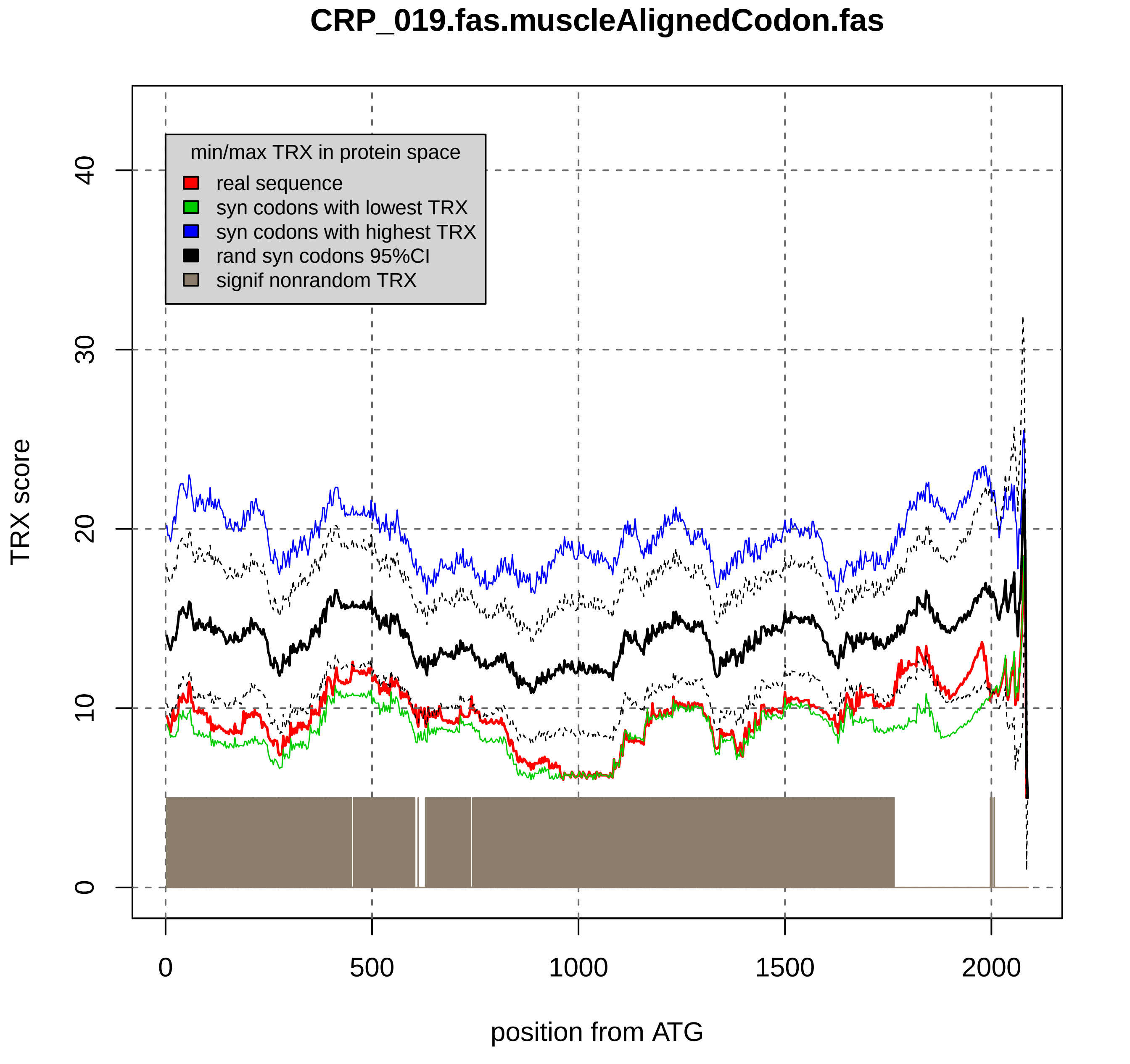

CRP_019.fas.muscleAlignedCodon.fas
min/max TRX in protein space
real sequence
syn codons with lowest TRX
syn codons with highest TRX
rand syn codons 95%CI
signif nonrandom TRX
TRX score
0
10
20
30
40
0
500
1000
1500
2000
position from ATG

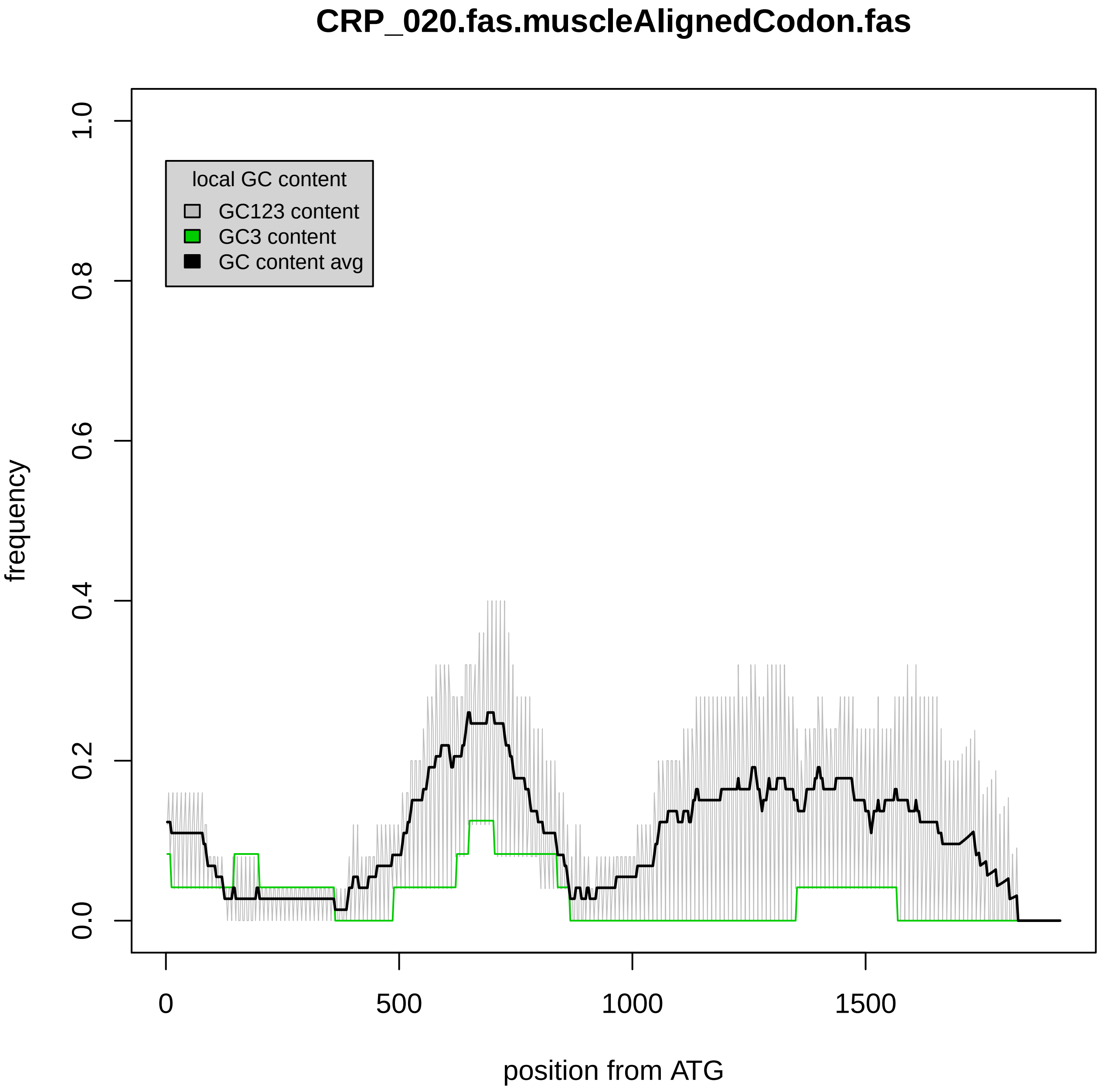
CRP_020.fas.muscleAlignedCodon.fas
local GC content
GC123 content
GC3 content
GC content avg
frequency
0.0
0.2
0.4
0.6
0.8
1.0
0
500
1000
1500
position from ATG

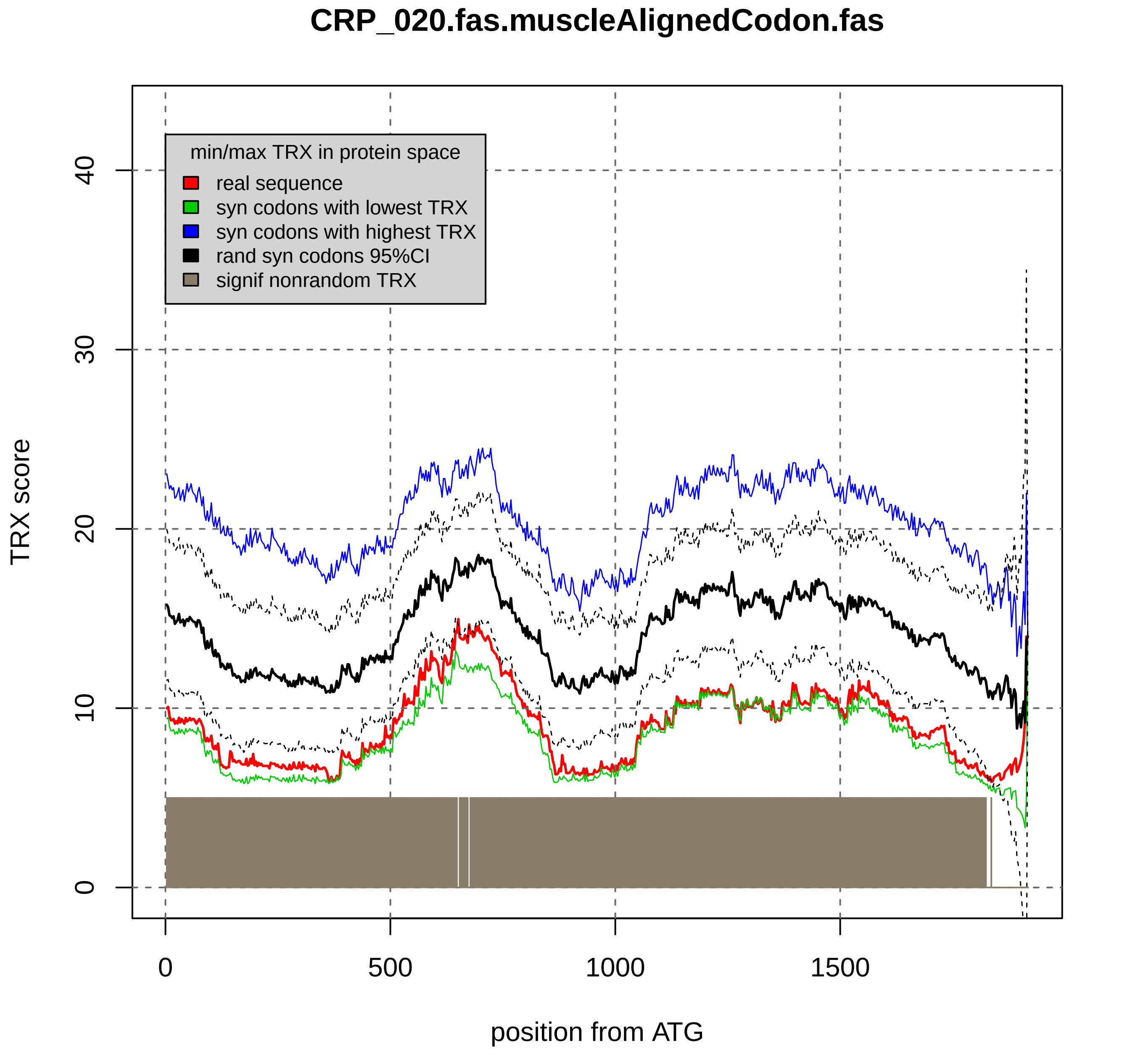

CRP_020.fas.muscleAlignedCodon.fas
min/max TRX in protein space
real sequence
syn codons with lowest TRX
syn codons with highest TRX
rand syn codons 95%CI
signif nonrandom TRX
TRX score
0
10
20
30
40
0
500
1000
1500
position from ATG

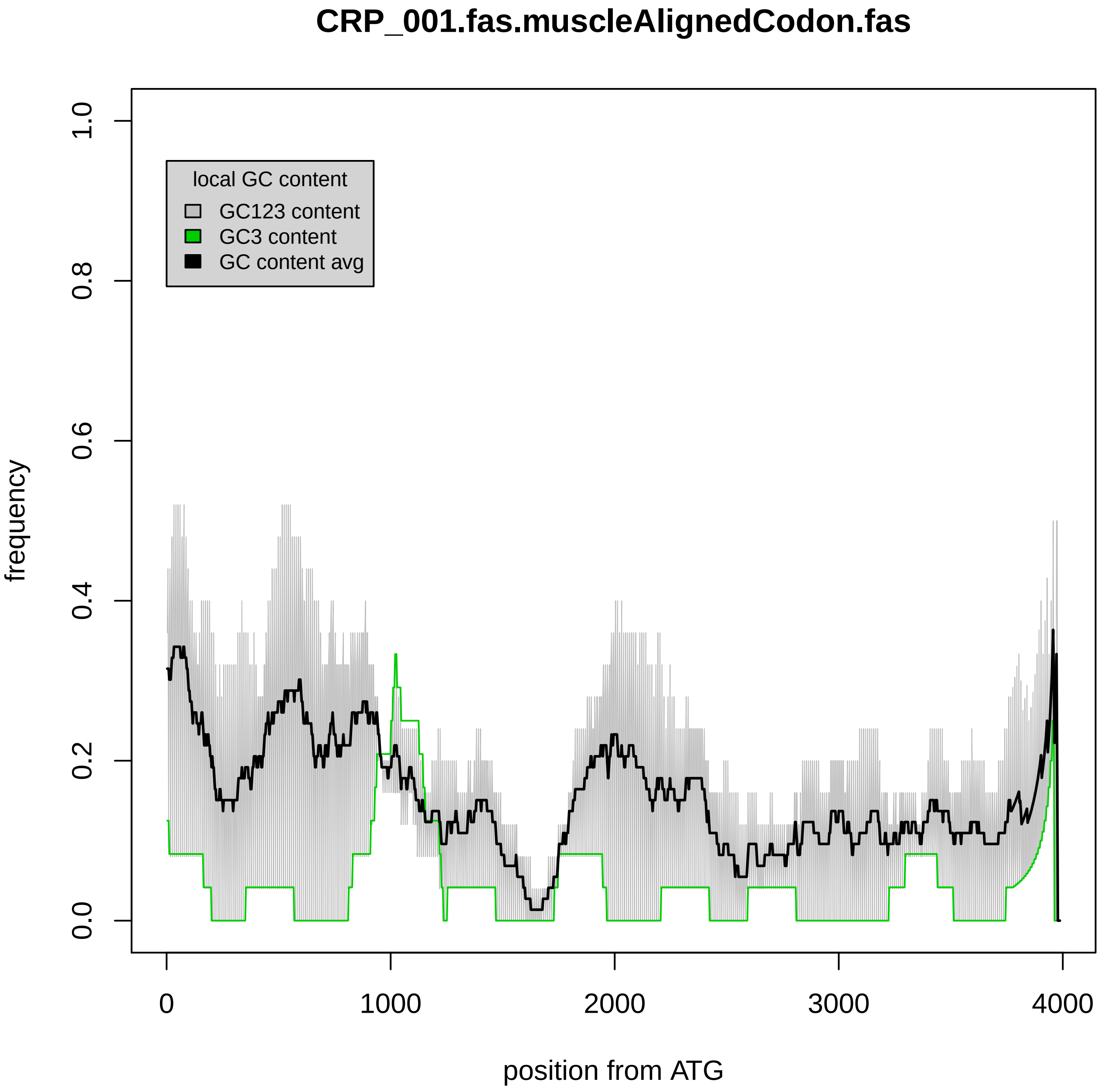

CRP_001.fas.muscleAlignedCodon.fas
local GC content
GC123 content
GC3 content
GC content avg
frequency
0.0
0.2
0.4
0.6
0.8
1.0
0
1000
2000
3000
4000
position from ATG

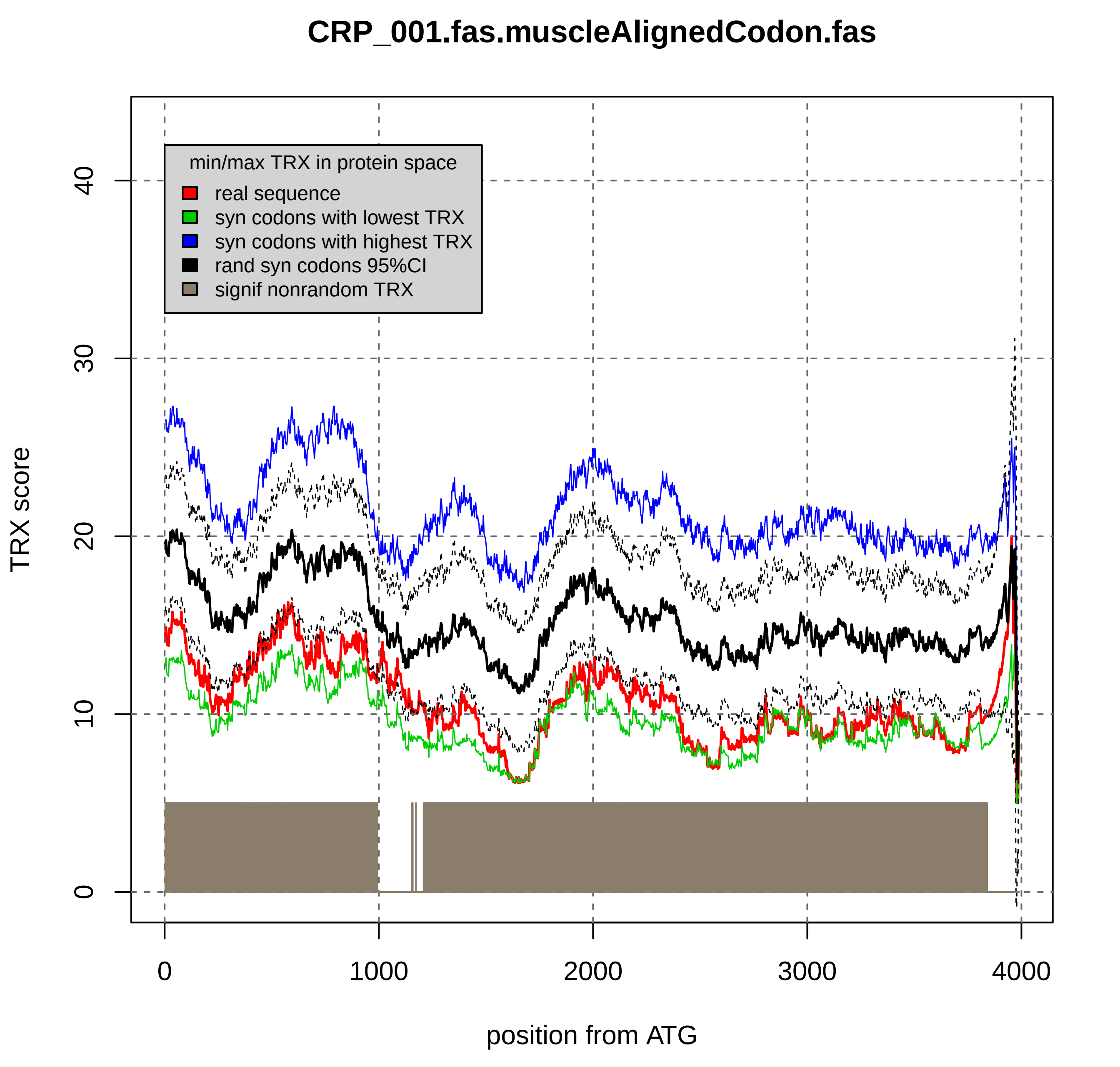

CRP_001.fas.muscleAlignedCodon.fas
min/max TRX in protein space
real sequence
syn codons with lowest TRX
syn codons with highest TRX
rand syn codons 95%CI
signif nonrandom TRX
TRX score
0
10
20
30
40
0
1000
2000
3000
4000
position from ATG

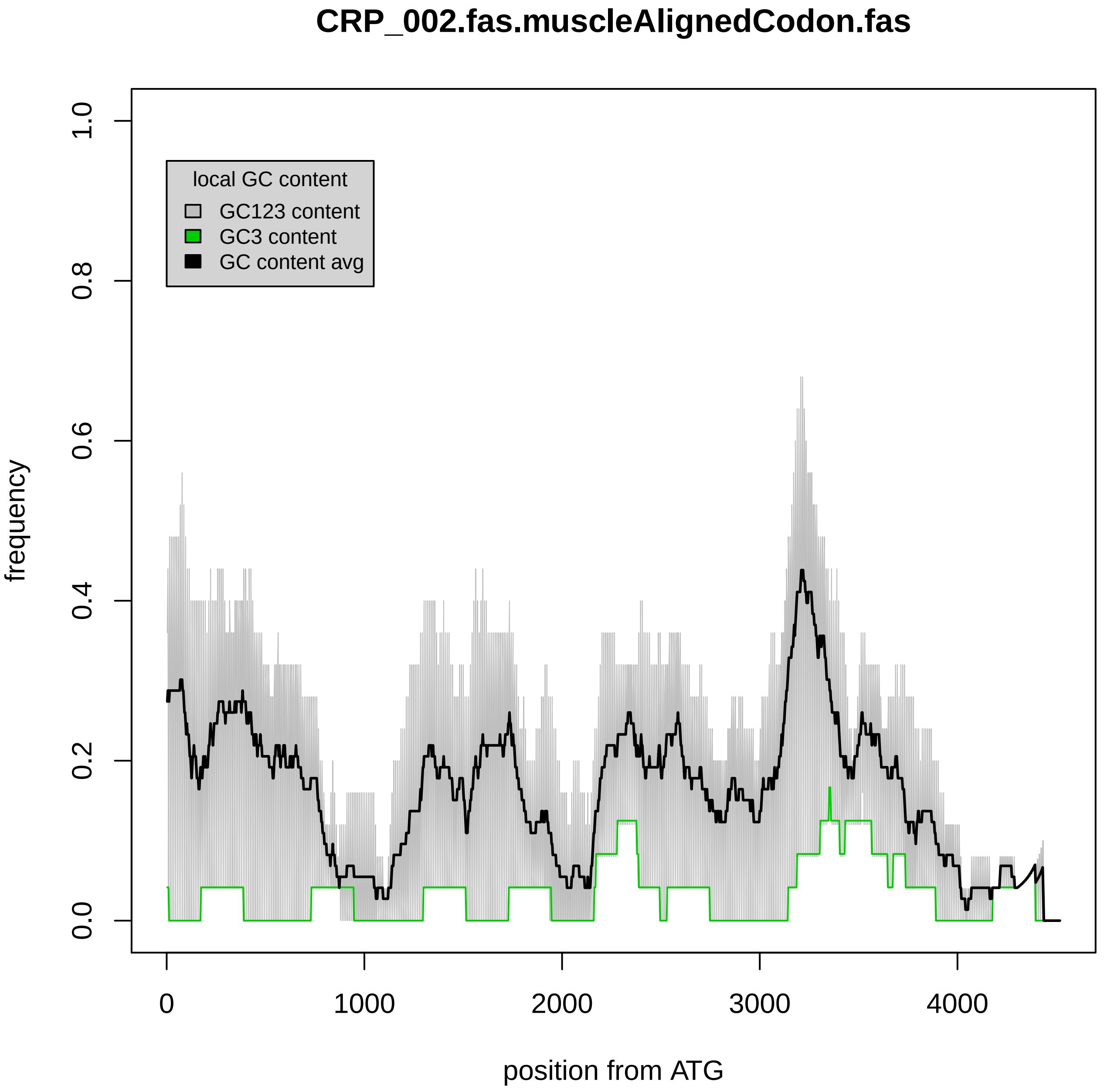
CRP_002.fas.muscleAlignedCodon.fas
local GC content
GC123 content
GC3 content
GC content avg
frequency
0.0
0.2
0.4
0.6
0.8
1.0
0
1000
2000
3000
4000
position from ATG

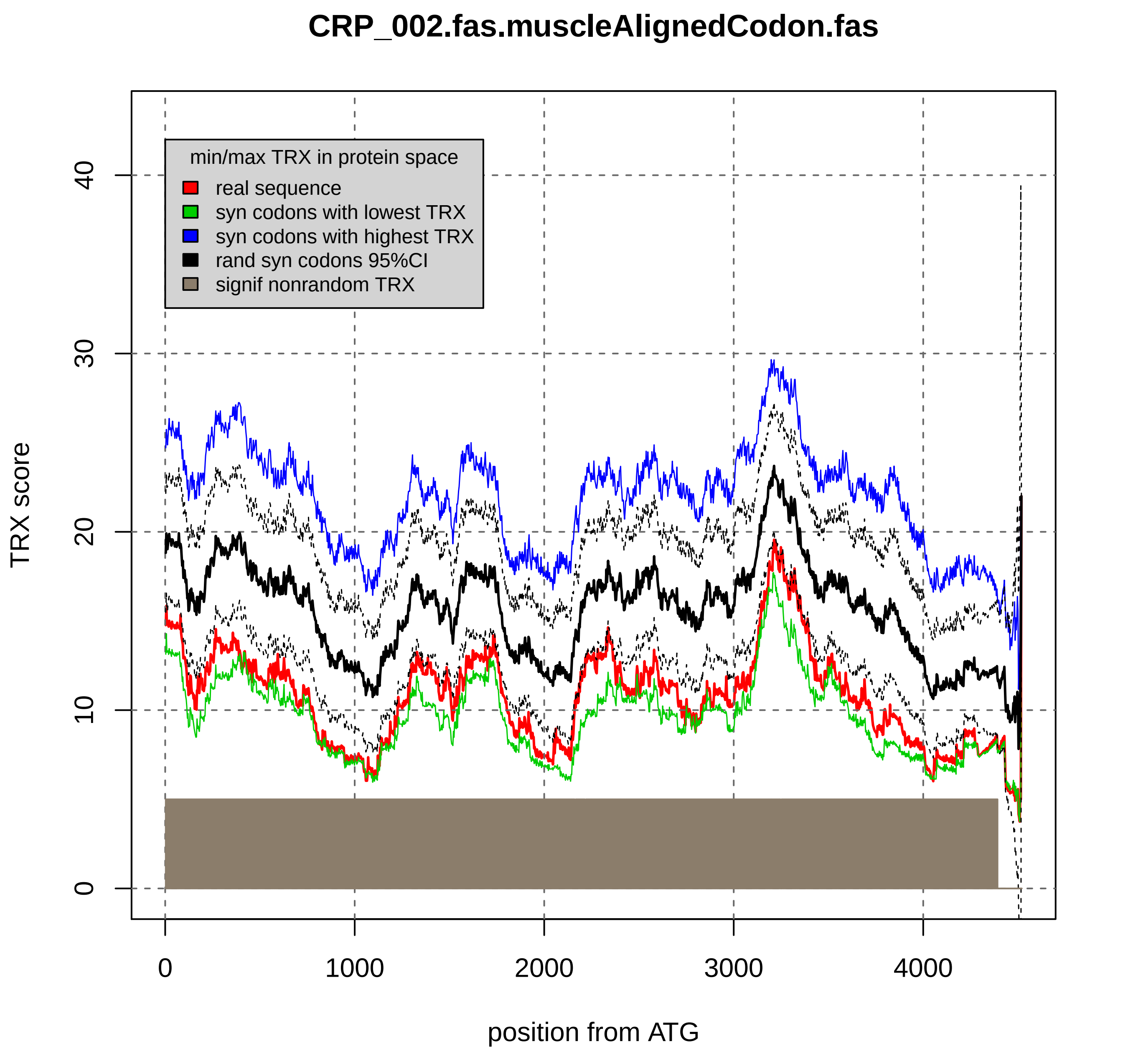

CRP_002.fas.muscleAlignedCodon.fas
min/max TRX in protein space
real sequence
syn codons with lowest TRX
syn codons with highest TRX
rand syn codons 95%CI
signif nonrandom TRX
TRX score
0
10
20
30
40
0
1000
2000
3000
4000
position from ATG

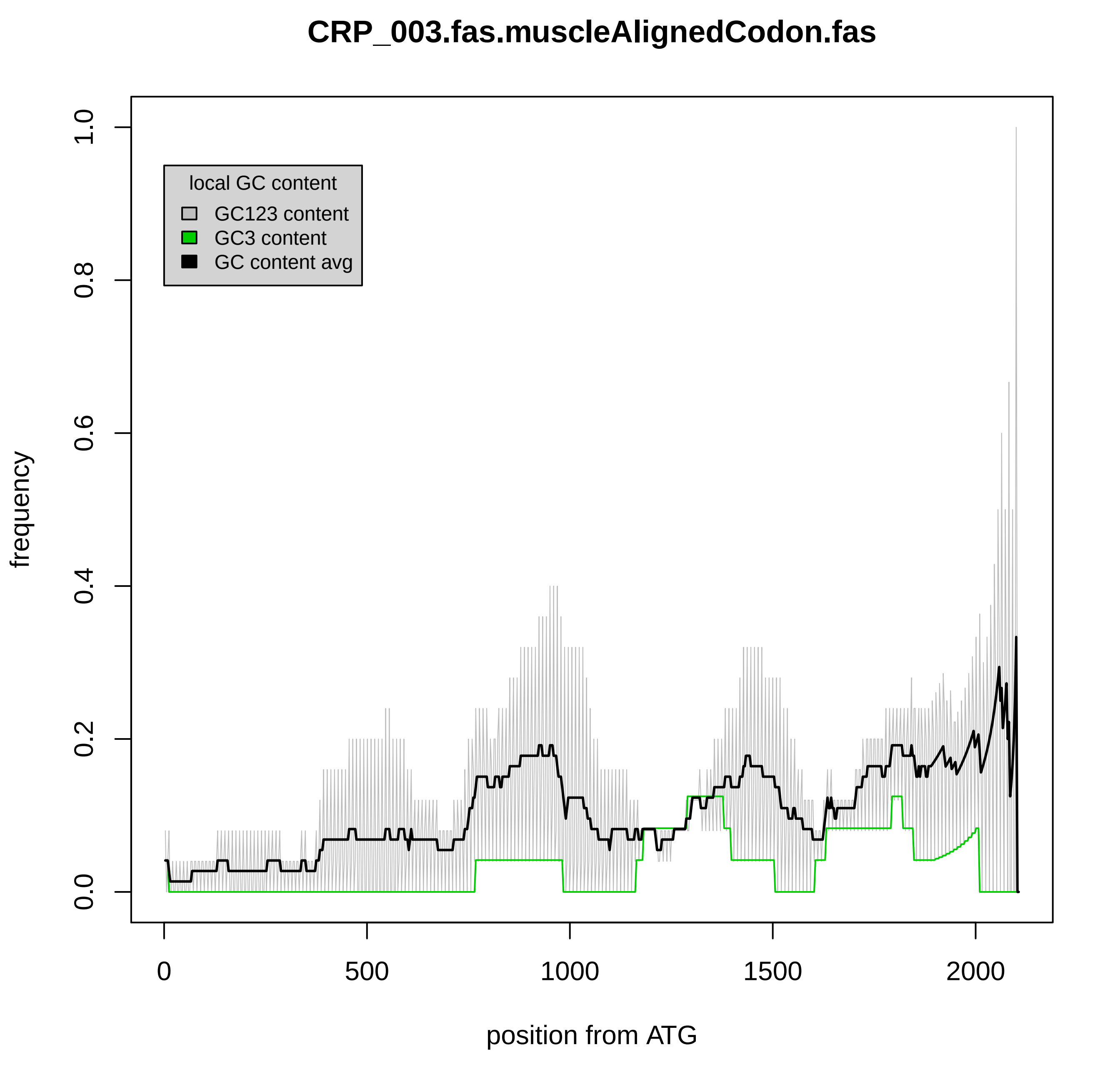
CRP_003.fas.muscleAlignedCodon.fas
local GC content
GC123 content
GC3 content
GC content avg
frequency
0.0
0.2
0.4
0.6
0.8
1.0
0
500
1000
1500
2000
position from ATG

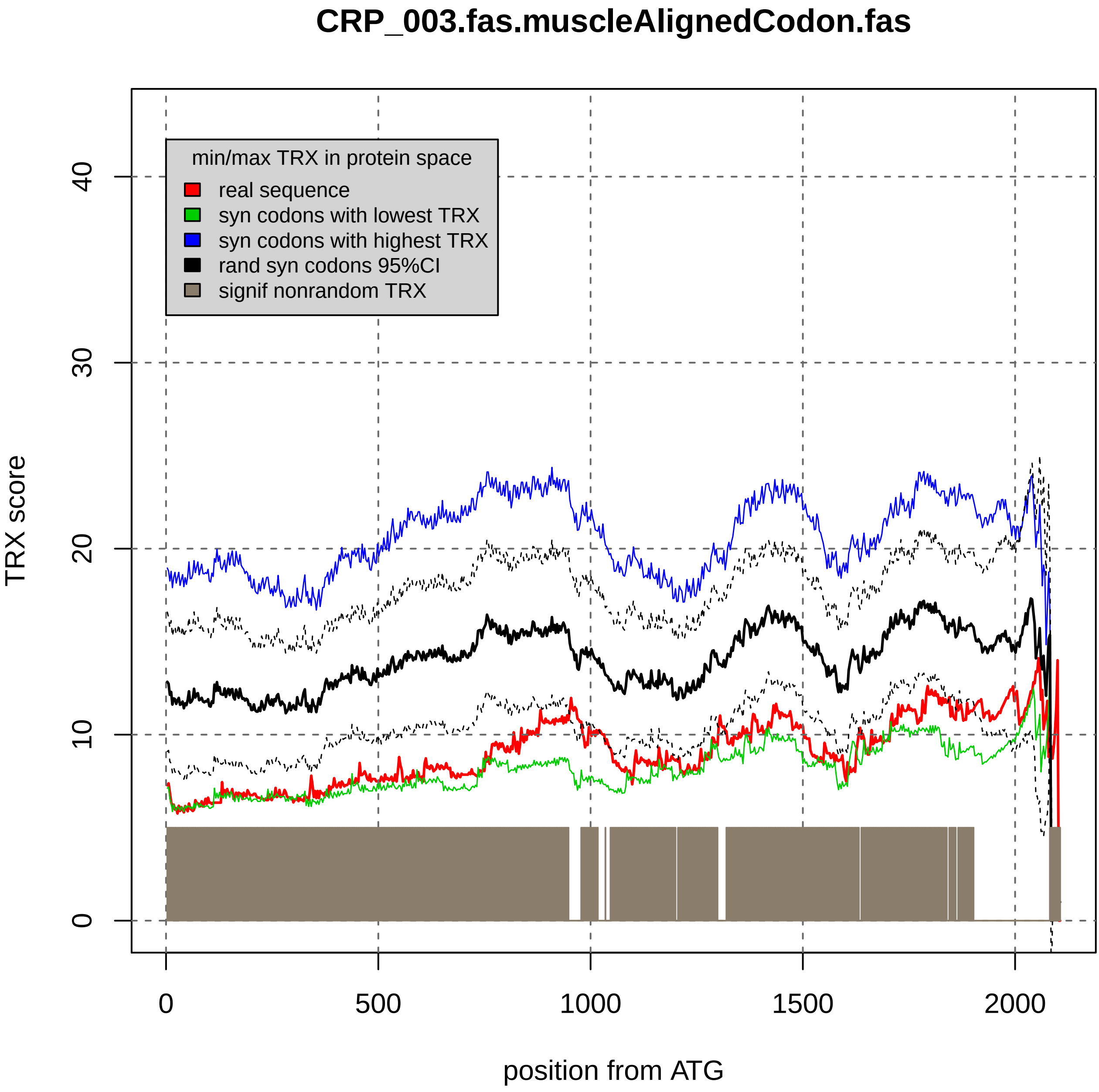
CRP_003.fas.muscleAlignedCodon.fas
min/max TRX in protein space
real sequence
syn codons with lowest TRX
syn codons with highest TRX
rand syn codons 95%CI
signif nonrandom TRX
TRX score
0
10
20
30
40
0
500
1000
1500
2000
position from ATG

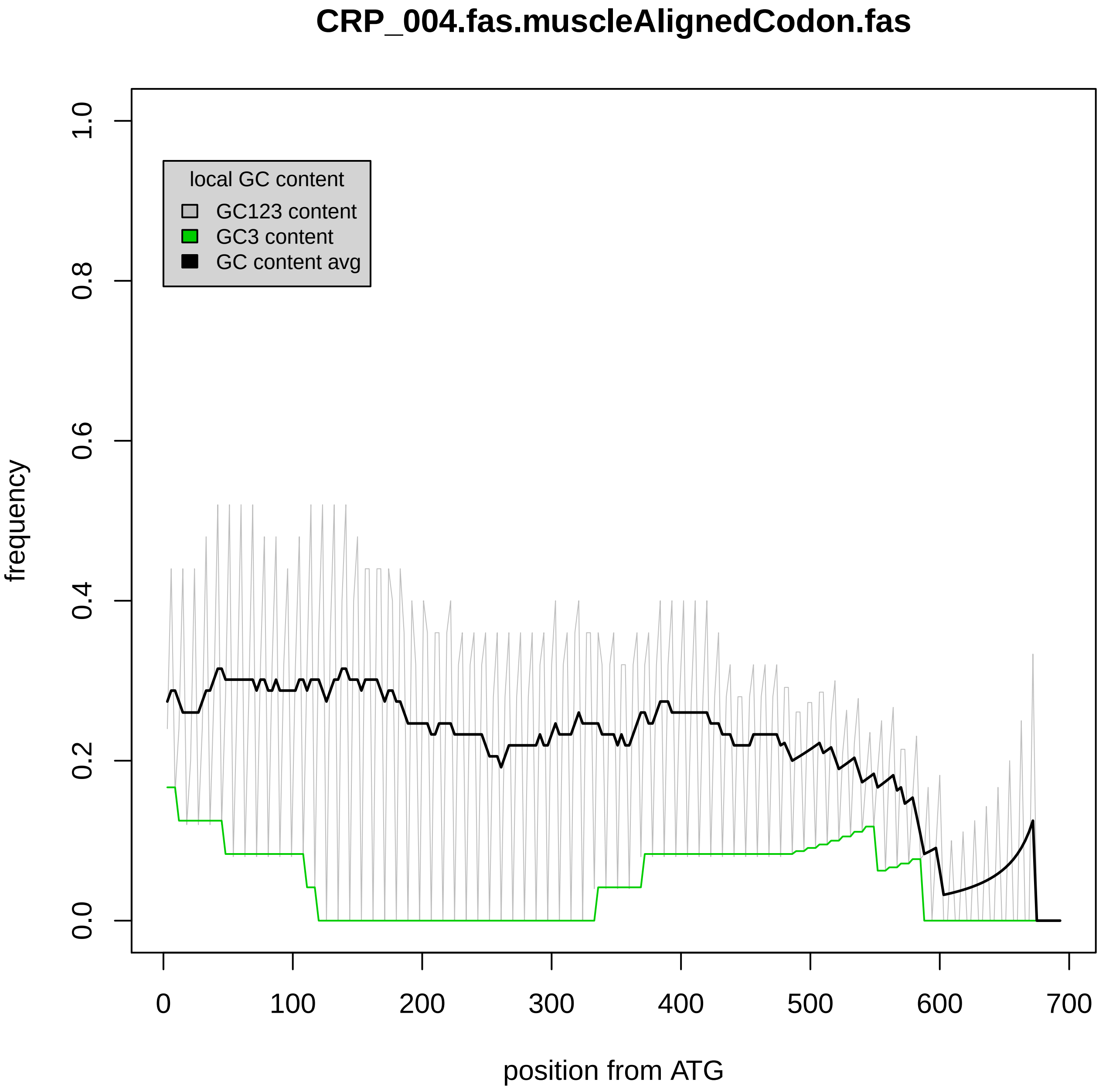

CRP_004.fas.muscleAlignedCodon.fas
local GC content
GC123 content
GC3 content
GC content avg
frequency
0.0
0.2
0.4
0.6
0.8
1.0
0
100
200
300
400
500
600
700
position from ATG

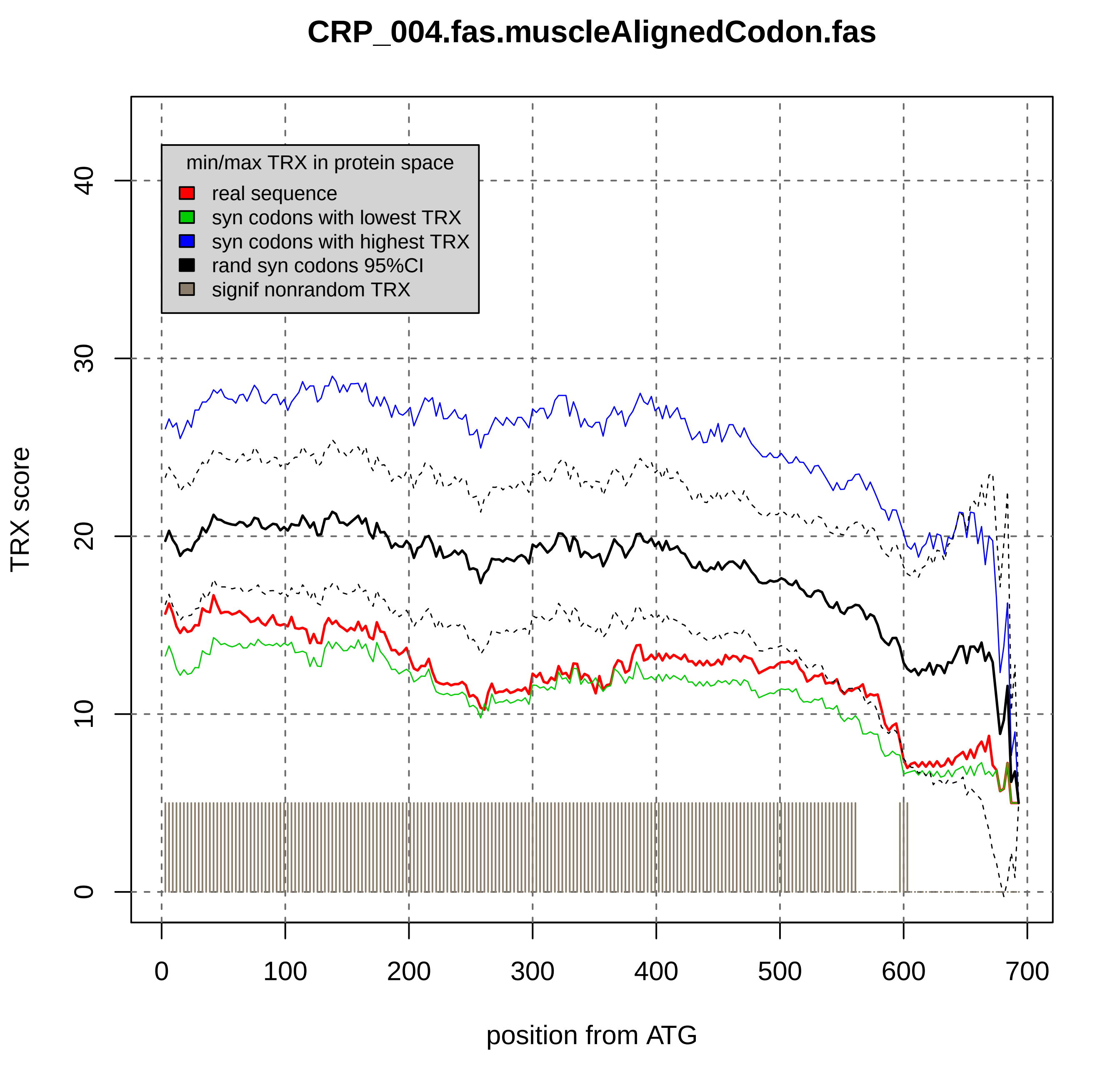

CRP_004.fas.muscleAlignedCodon.fas
min/max TRX in protein space
real sequence
syn codons with lowest TRX
syn codons with highest TRX
rand syn codons 95%CI
signif nonrandom TRX
TRX score
position from ATG
0
10
20
30
40
0
100
200
300
400
500
600
700

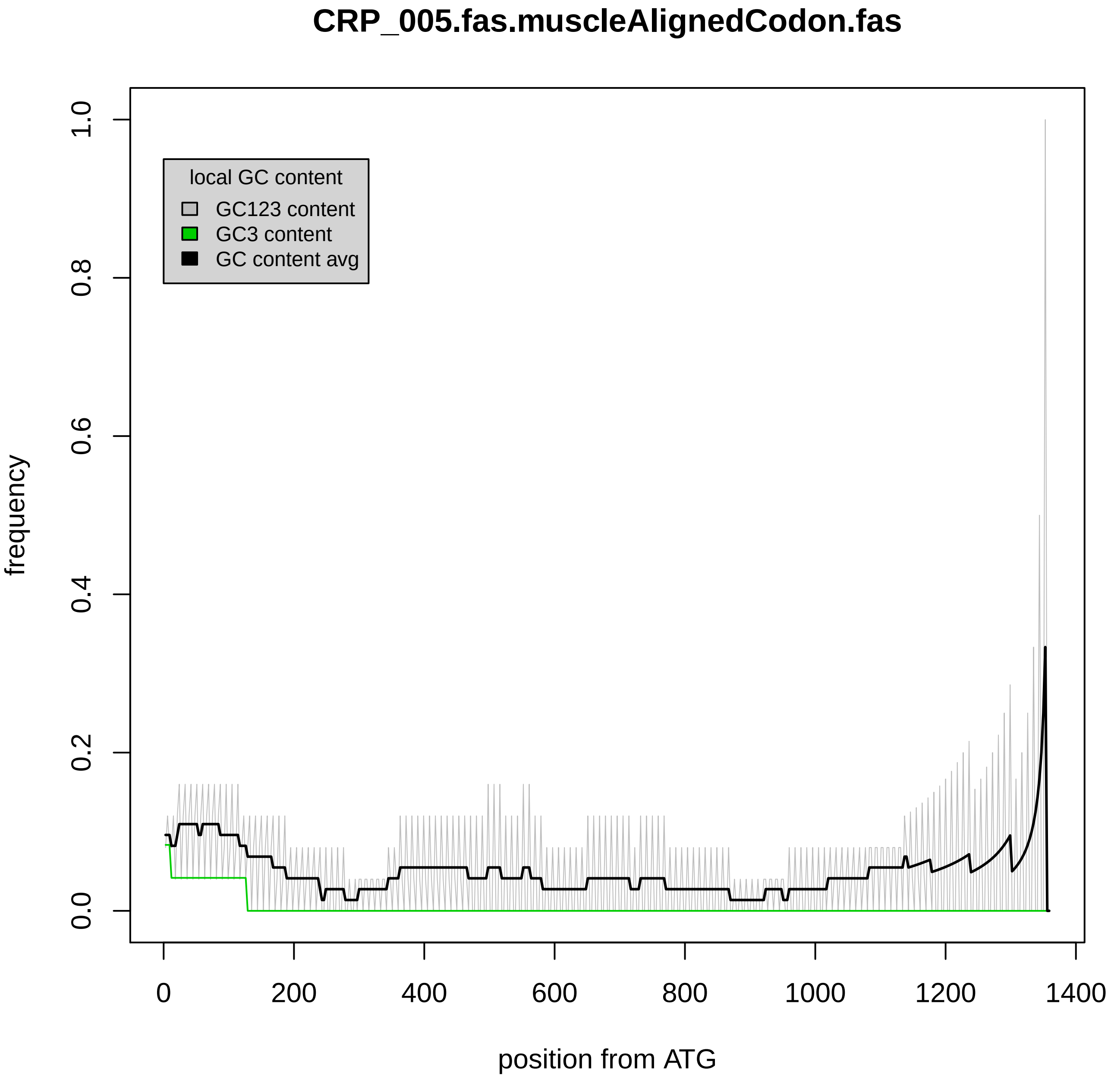

CRP_005.fas.muscleAlignedCodon.fas
local GC content
GC123 content
GC3 content
GC content avg
frequency
0.0
0.2
0.4
0.6
0.8
1.0
0
200
400
600
800
1000
1200
1400
position from ATG

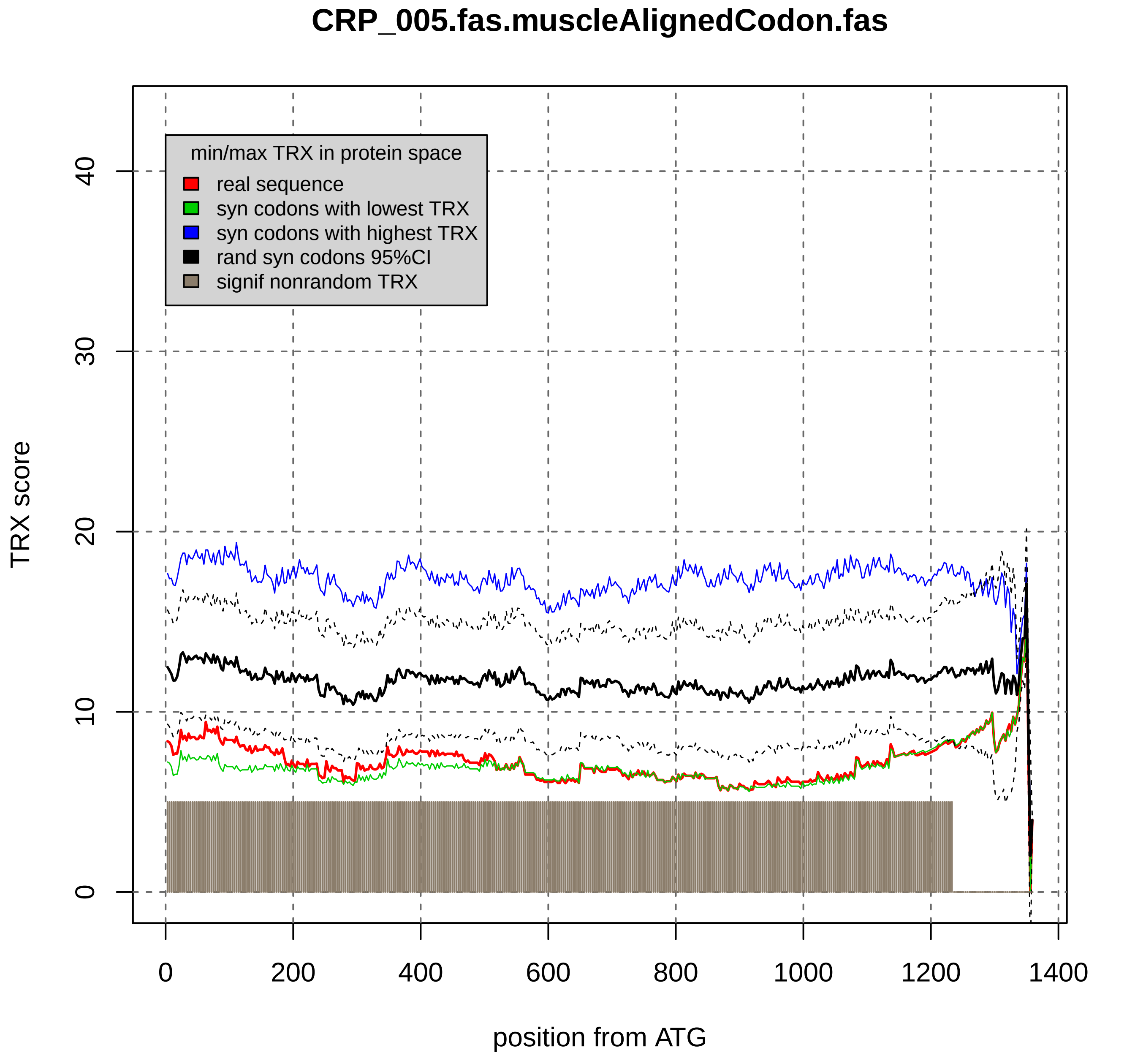
CRP_005.fas.muscleAlignedCodon.fas
min/max TRX in protein space
real sequence
syn codons with lowest TRX
syn codons with highest TRX
rand syn codons 95%CI
signif nonrandom TRX
TRX score
0
10
20
30
40
0
200
400
600
800
1000
1200
1400
position from ATG

CRP_006.fas.muscleAlignedCodon.fas

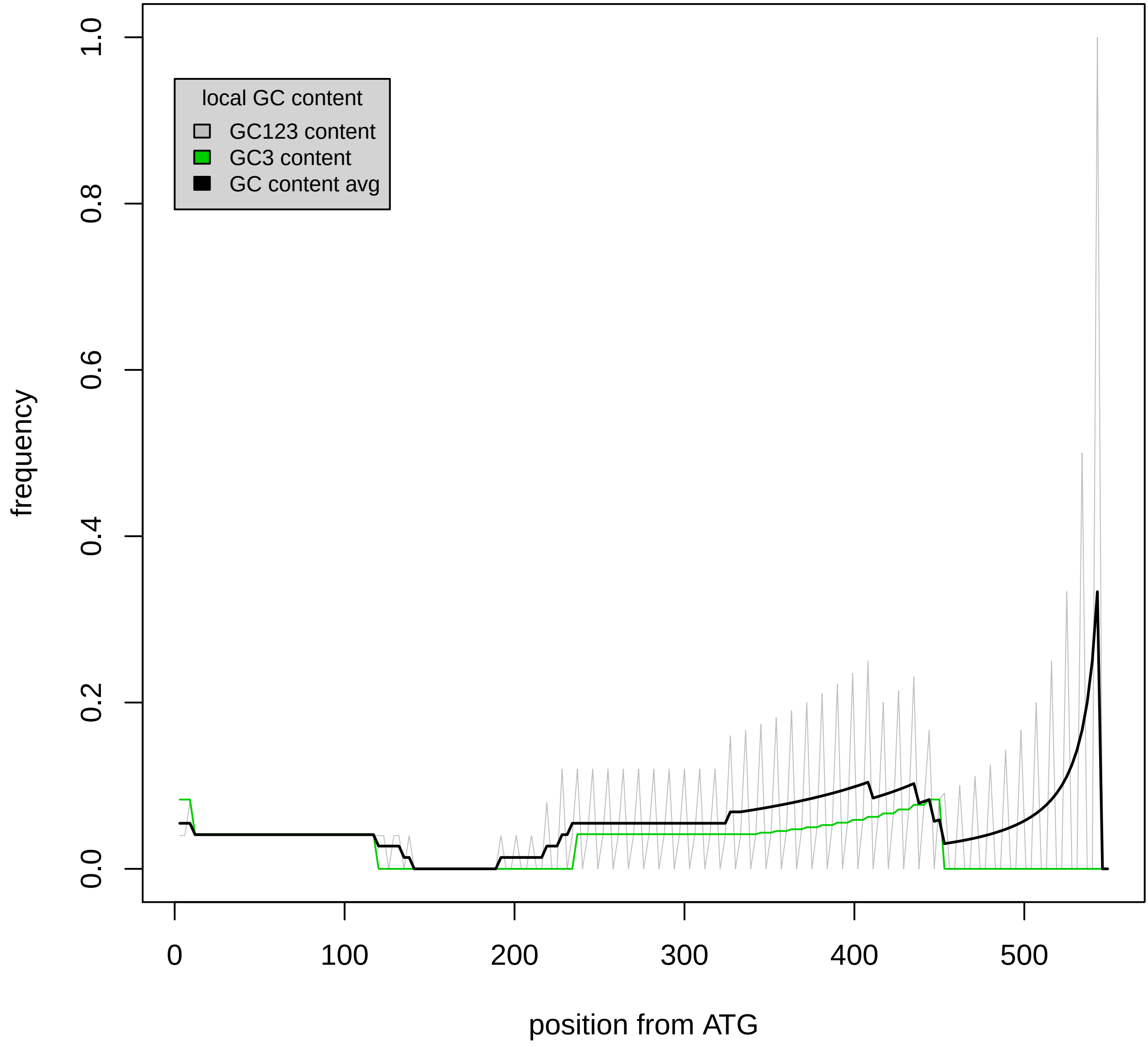

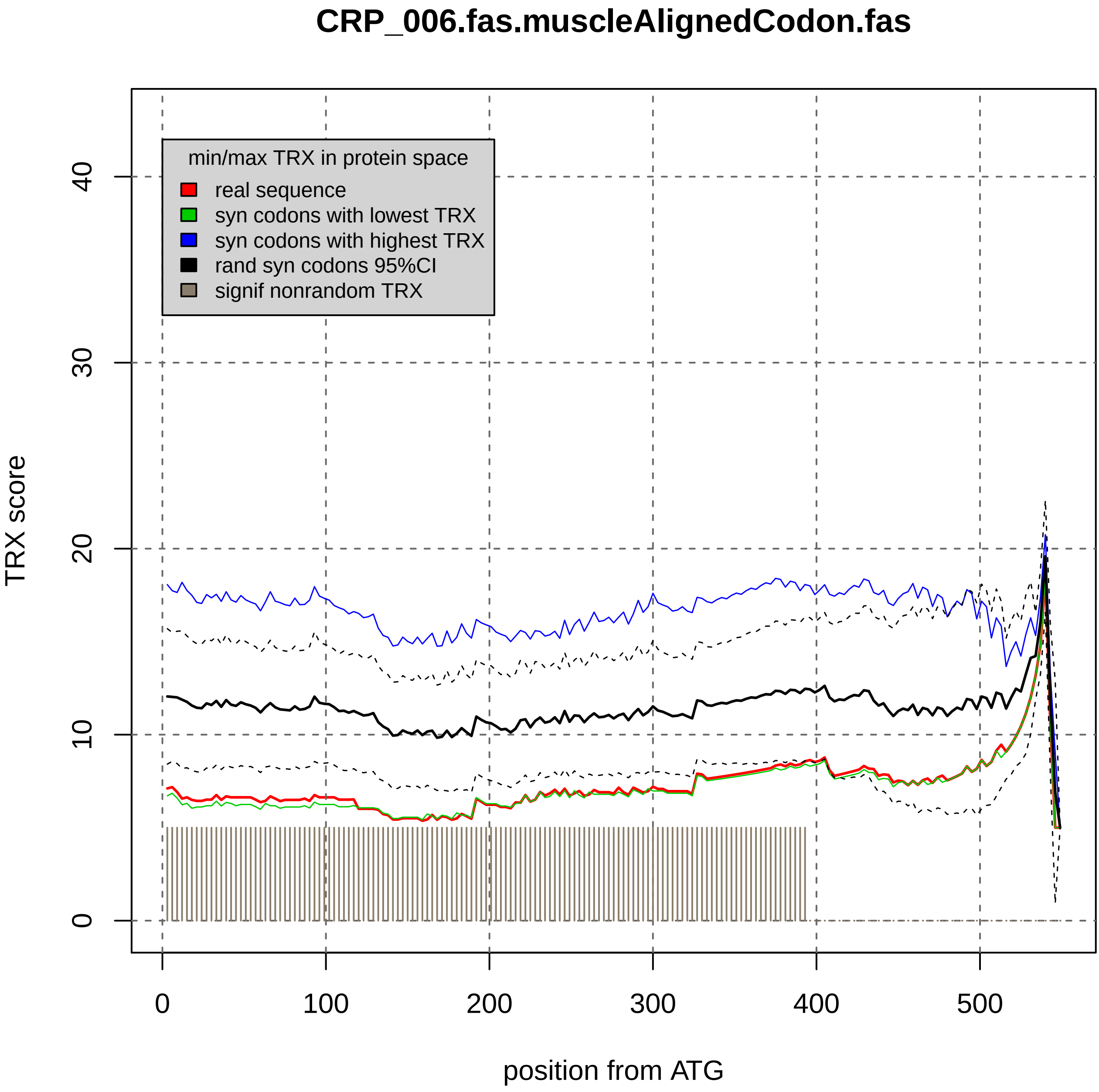
CRP_006.fas.muscleAlignedCodon.fas
min/max TRX in protein space
real sequence
syn codons with lowest TRX
syn codons with highest TRX
rand syn codons 95%CI
signif nonrandom TRX
TRX score
0
10
20
30
40
0
100
200
300
400
500
position from ATG

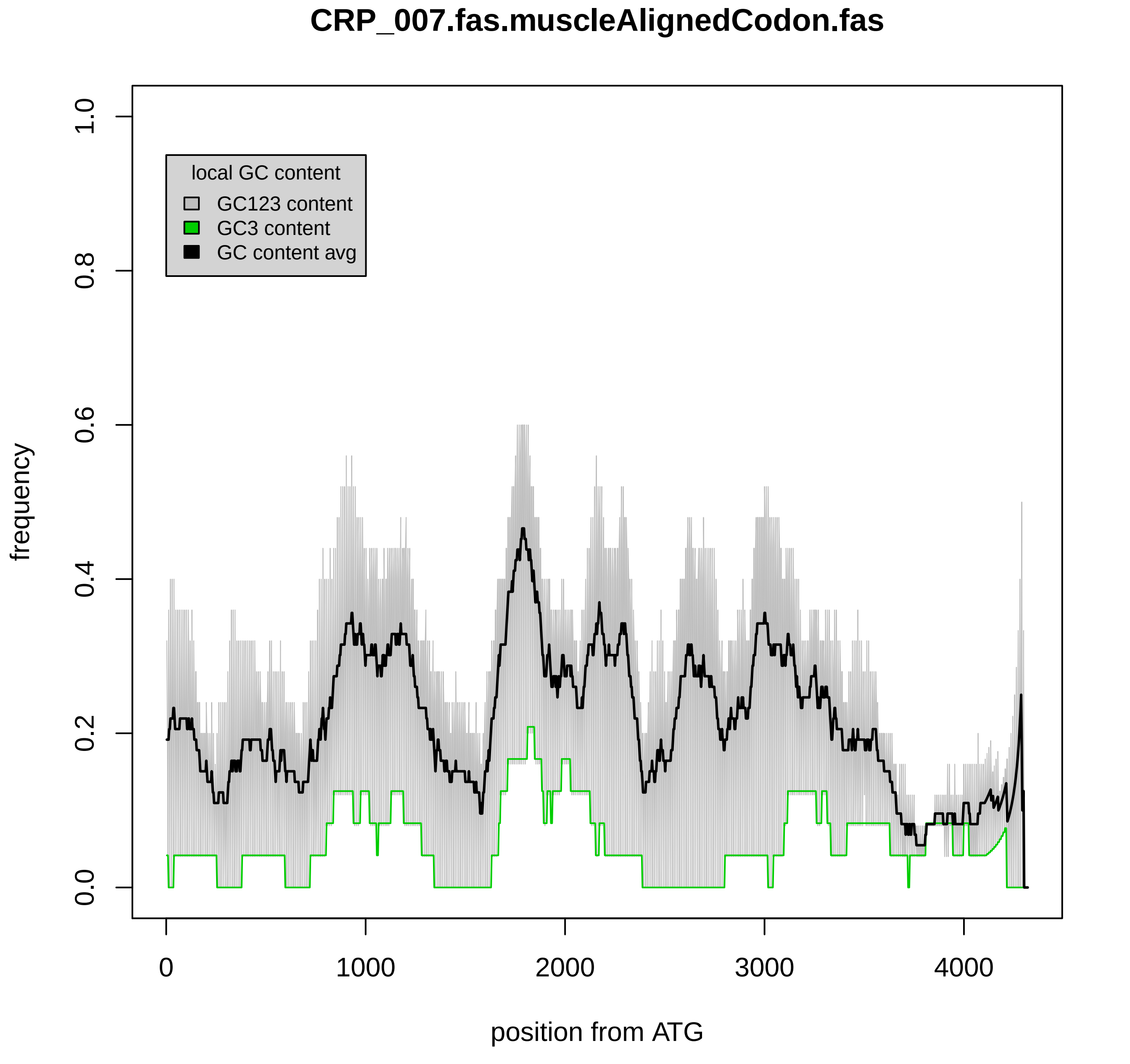
CRP_007.fas.muscleAlignedCodon.fas
local GC content
GC123 content
GC3 content
GC content avg
frequency
0.0
0.2
0.4
0.6
0.8
1.0
0
1000
2000
3000
4000
position from ATG

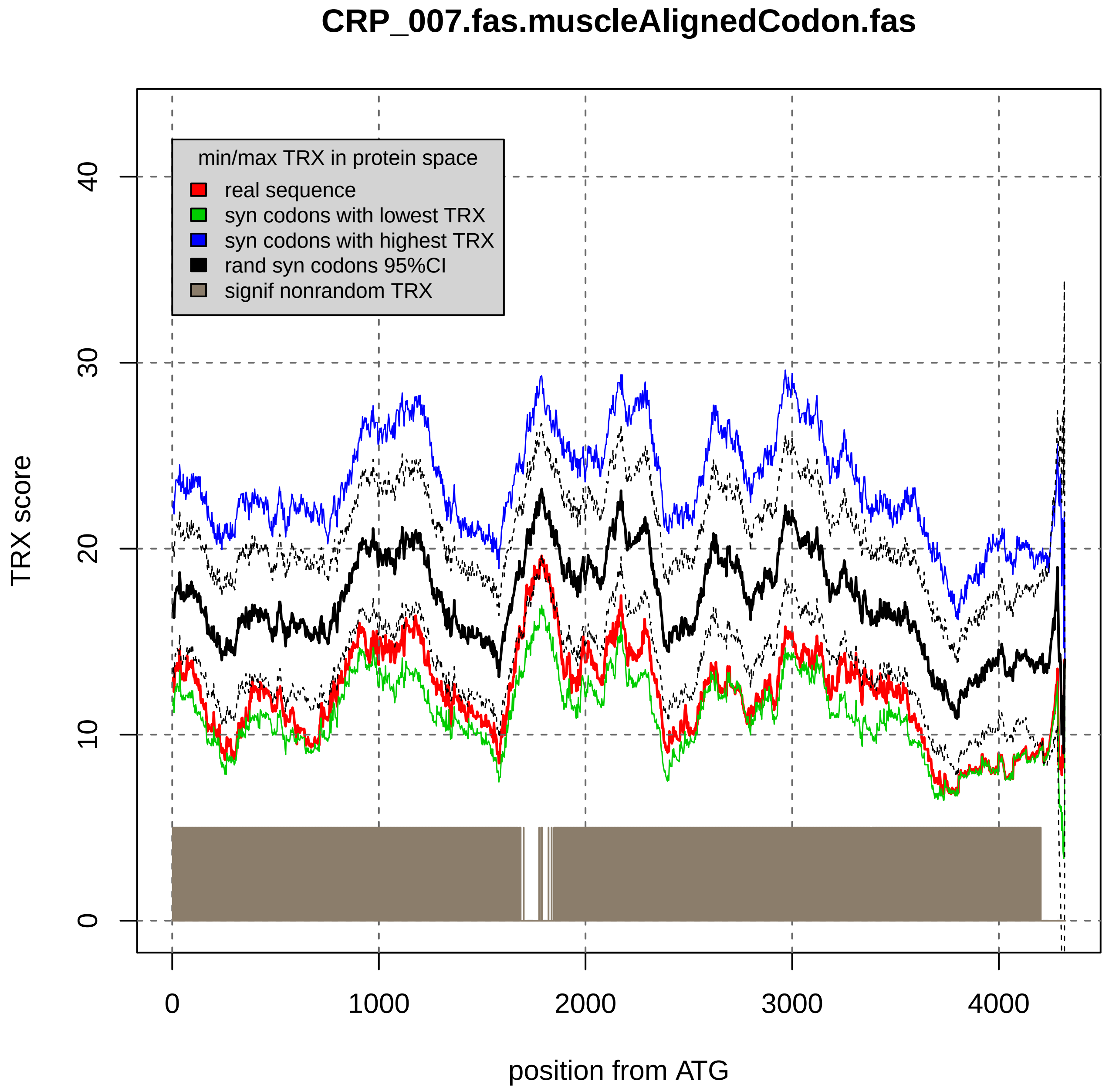
CRP_007.fas.muscleAlignedCodon.fas
min/max TRX in protein space
real sequence
syn codons with lowest TRX
syn codons with highest TRX
rand syn codons 95%CI
signif nonrandom TRX
TRX score
0
10
20
30
40
0
1000
2000
3000
4000
position from ATG

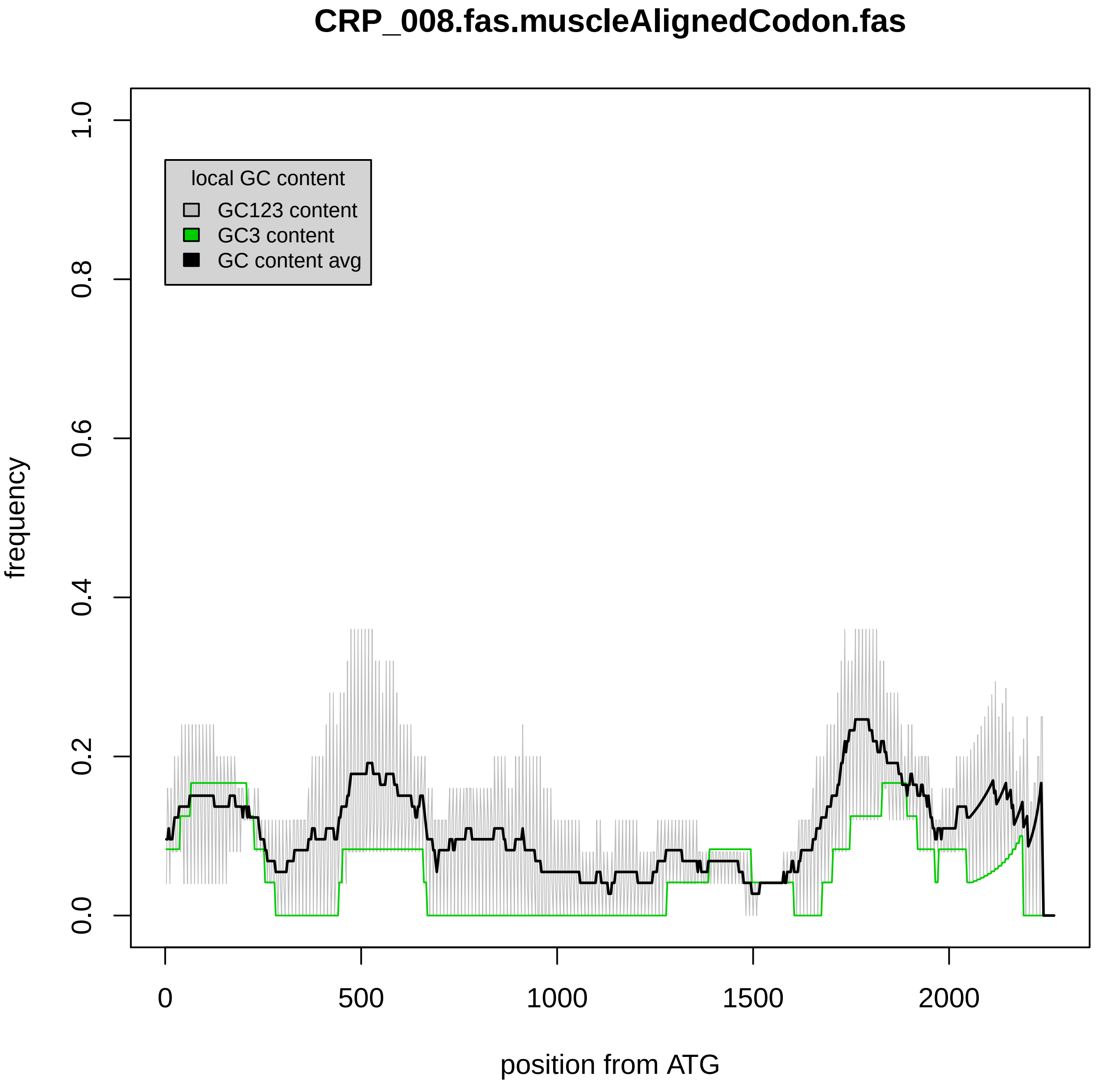

CRP_008.fas.muscleAlignedCodon.fas
local GC content
GC123 content
GC3 content
GC content avg
frequency
position from ATG
0.0
0.2
0.4
0.6
0.8
1.0
0
500
1000
1500
2000

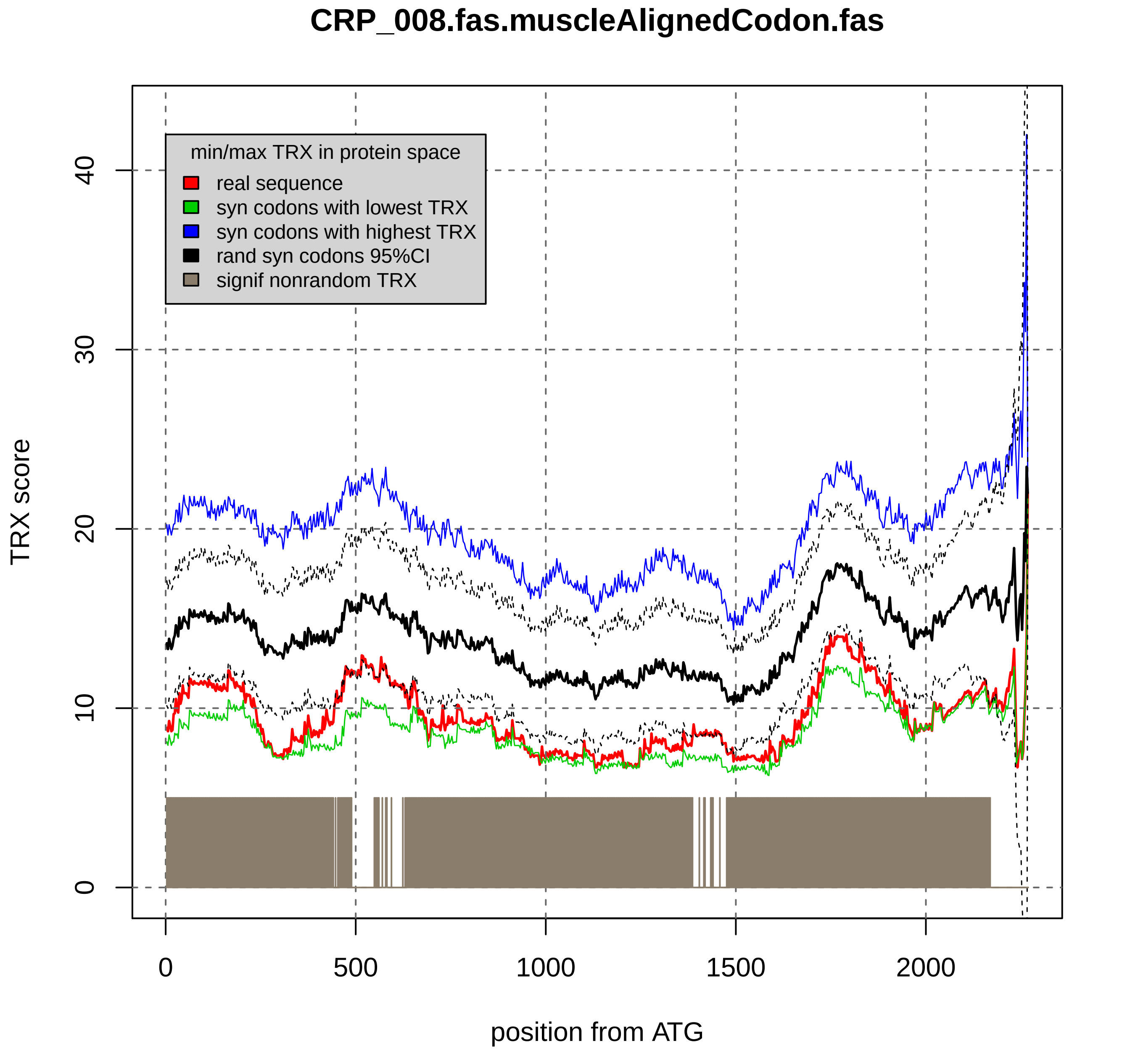

CRP_008.fas.muscleAlignedCodon.fas
min/max TRX in protein space
real sequence
syn codons with lowest TRX
syn codons with highest TRX
rand syn codons 95%CI
signif nonrandom TRX
TRX score
0
10
20
30
40
0
500
1000
1500
2000
position from ATG

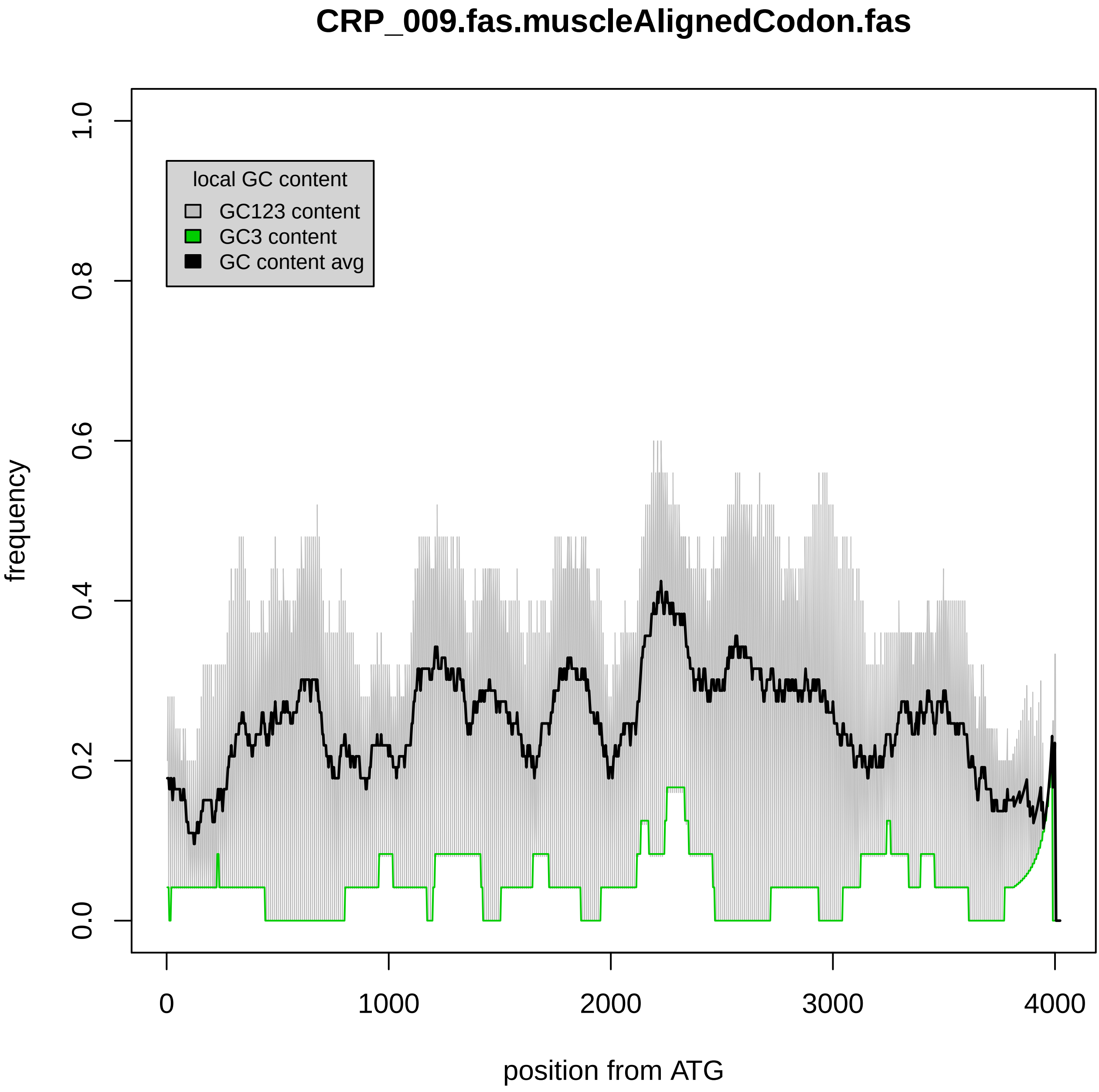
CRP_009.fas.muscleAlignedCodon.fas
local GC content
GC123 content
GC3 content
GC content avg
frequency
0.0
0.2
0.4
0.6
0.8
1.0
0
1000
2000
3000
4000
position from ATG

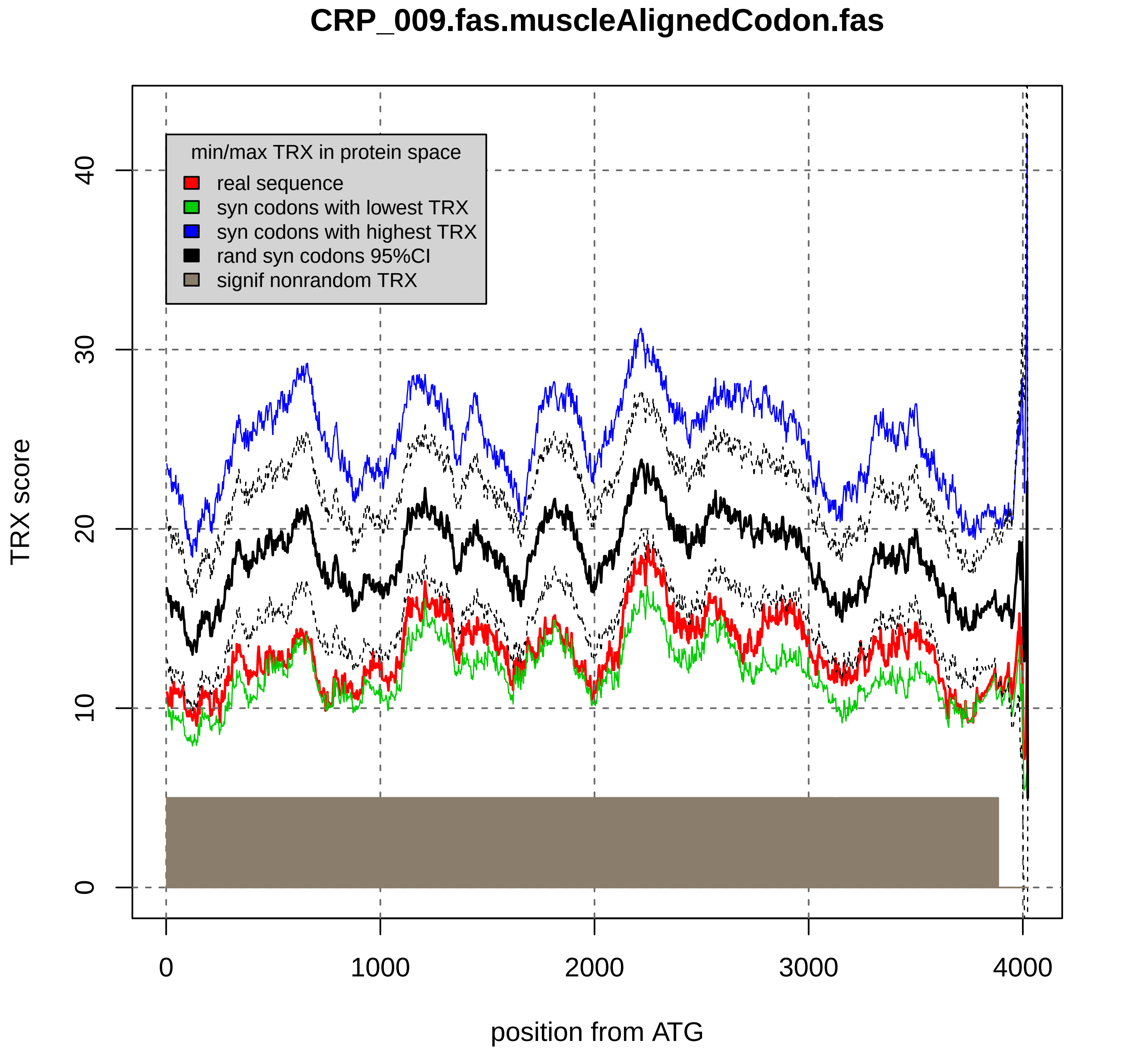
CRP_009.fas.muscleAlignedCodon.fas
min/max TRX in protein space
real sequence
syn codons with lowest TRX
syn codons with highest TRX
rand syn codons 95%CI
signif nonrandom TRX
TRX score
0
10
20
30
40
0
1000
2000
3000
4000
position from ATG

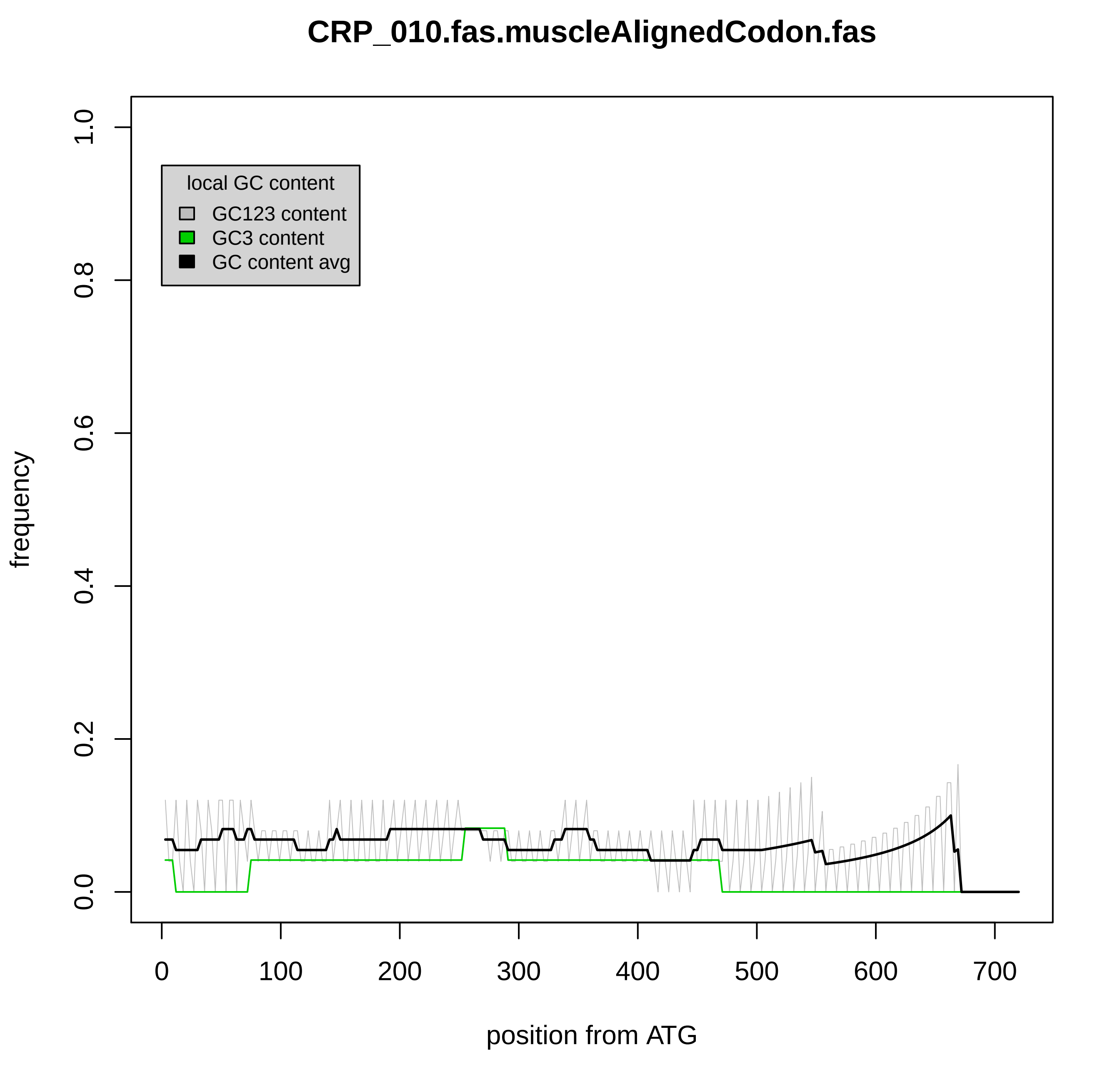
CRP_010.fas.muscleAlignedCodon.fas
local GC content
GC123 content
GC3 content
GC content avg
frequency
0.0
0.2
0.4
0.6
0.8
1.0
position from ATG
0
100
200
300
400
500
600
700

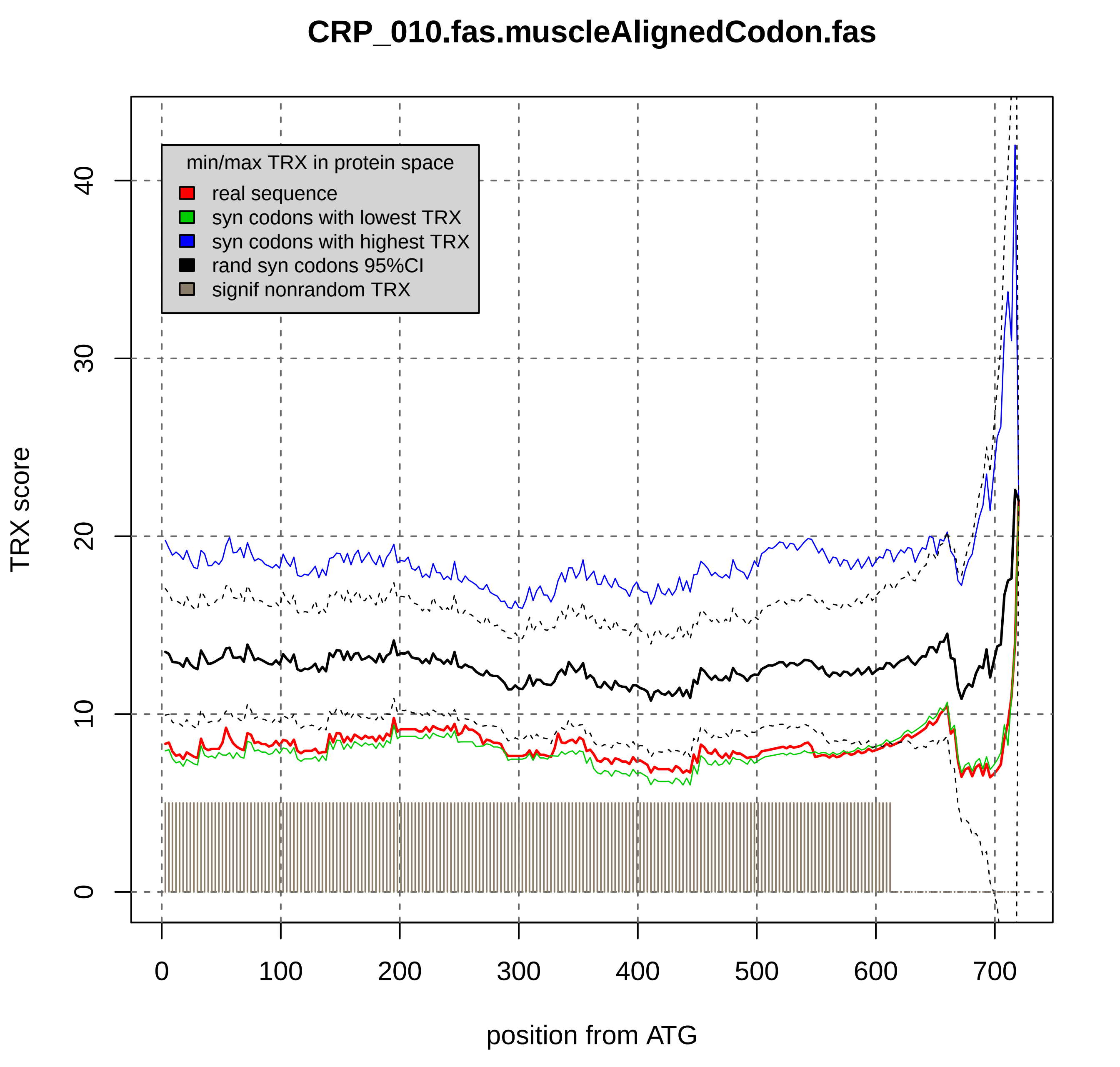
CRP_010.fas.muscleAlignedCodon.fas
min/max TRX in protein space
real sequence
syn codons with lowest TRX
syn codons with highest TRX
rand syn codons 95%CI
signif nonrandom TRX
TRX score
0
10
20
30
40
0
100
200
300
400
500
600
700
position from ATG

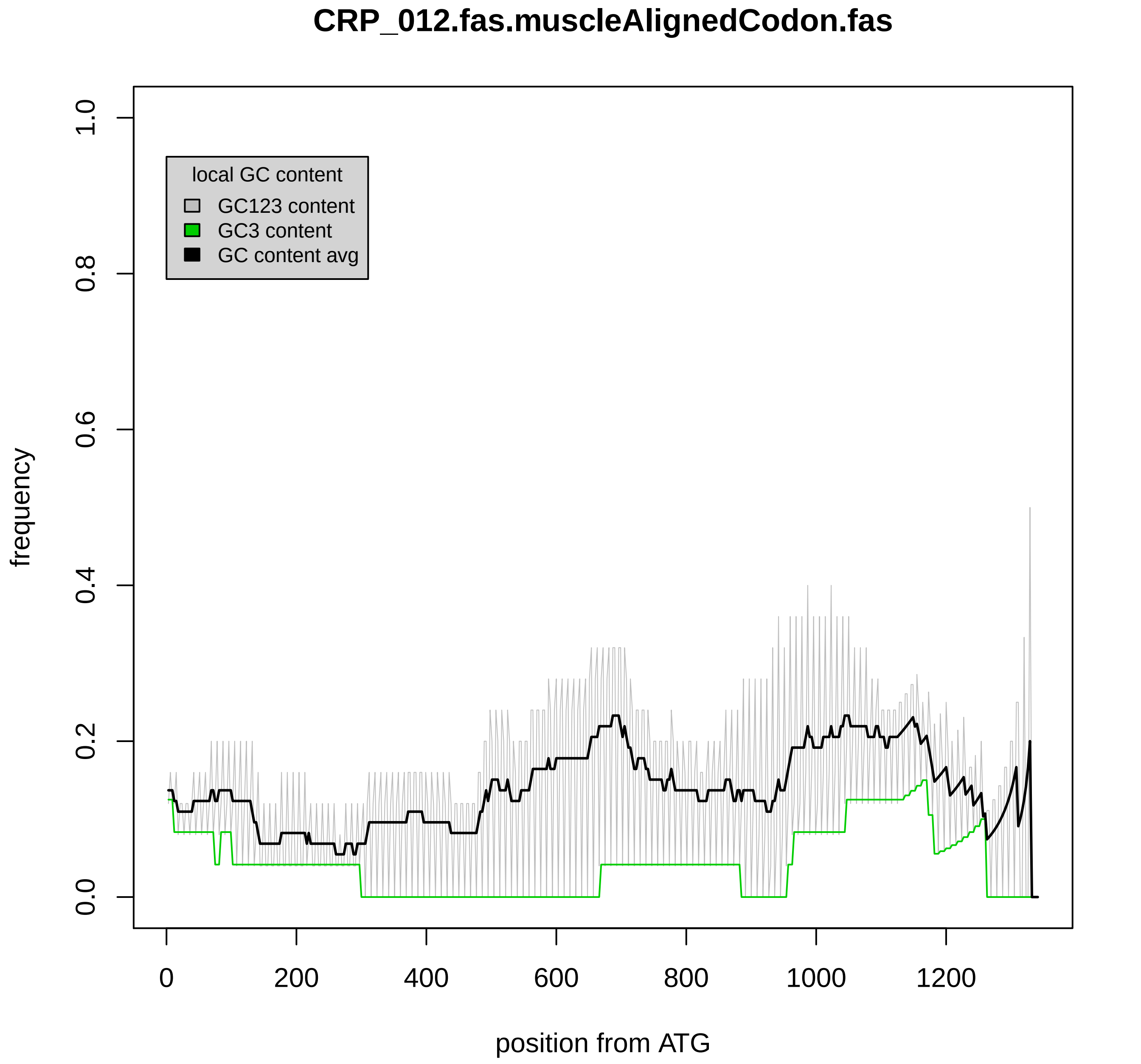

CRP_012.fas.muscleAlignedCodon.fas
local GC content
GC123 content
GC3 content
GC content avg
frequency
0.0
0.2
0.4
0.6
0.8
1.0
0
200
400
600
800
1000
1200
position from ATG

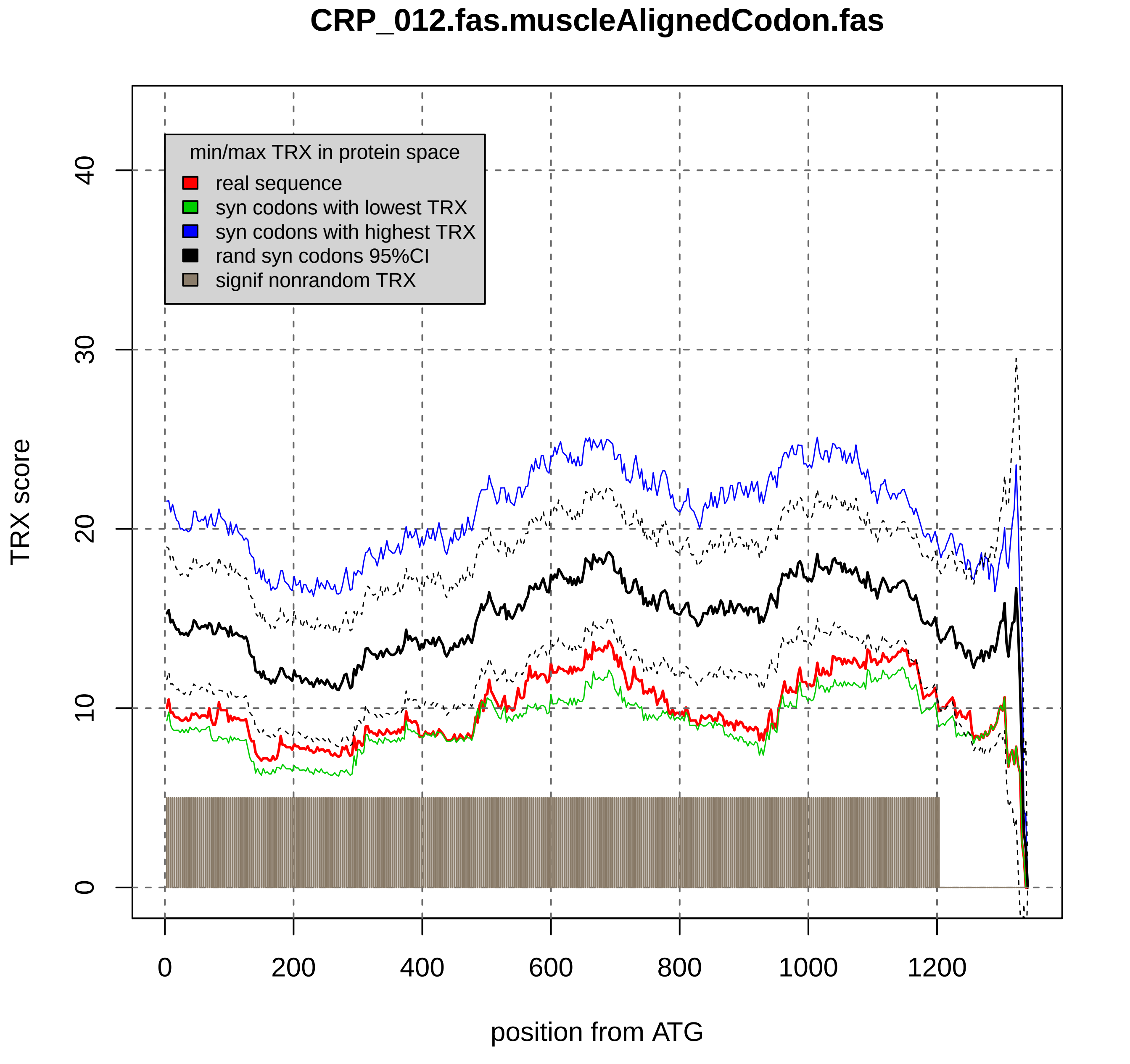

CRP_012.fas.muscleAlignedCodon.fas
min/max TRX in protein space
real sequence
syn codons with lowest TRX
syn codons with highest TRX
rand syn codons 95%CI
signif nonrandom TRX
TRX score
0
10
20
30
40
0
200
400
600
800
1000
1200
position from ATG

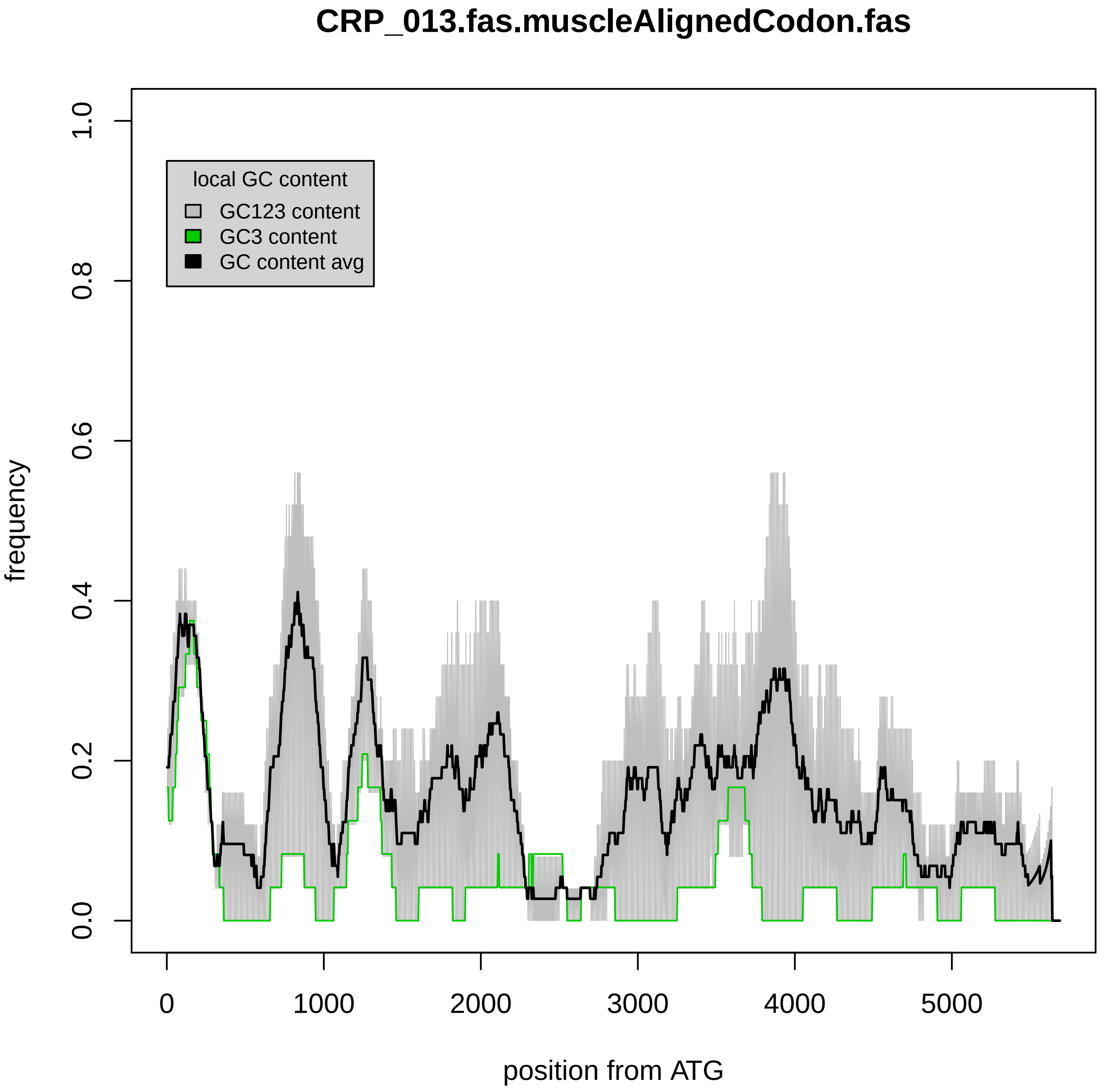
CRP_013.fas.muscleAlignedCodon.fas
local GC content
GC123 content
GC3 content
GC content avg
frequency
0.0
0.2
0.4
0.6
0.8
1.0
position from ATG
0
1000
2000
3000
4000
5000

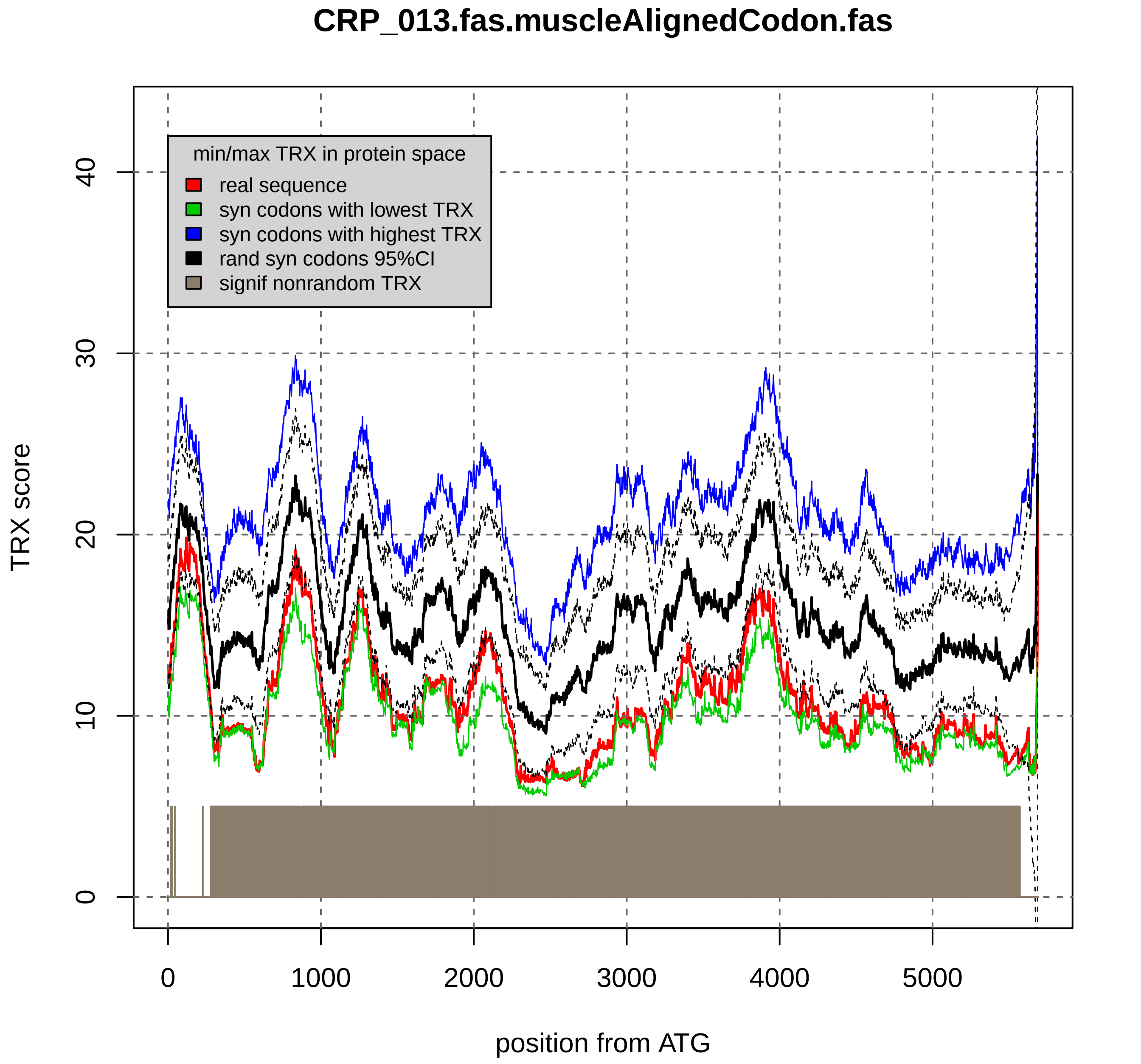
CRP_013.fas.muscleAlignedCodon.fas
min/max TRX in protein space
real sequence
syn codons with lowest TRX
syn codons with highest TRX
rand syn codons 95%CI
signif nonrandom TRX
TRX score
0
10
20
30
40
0
1000
2000
3000
4000
5000
position from ATG

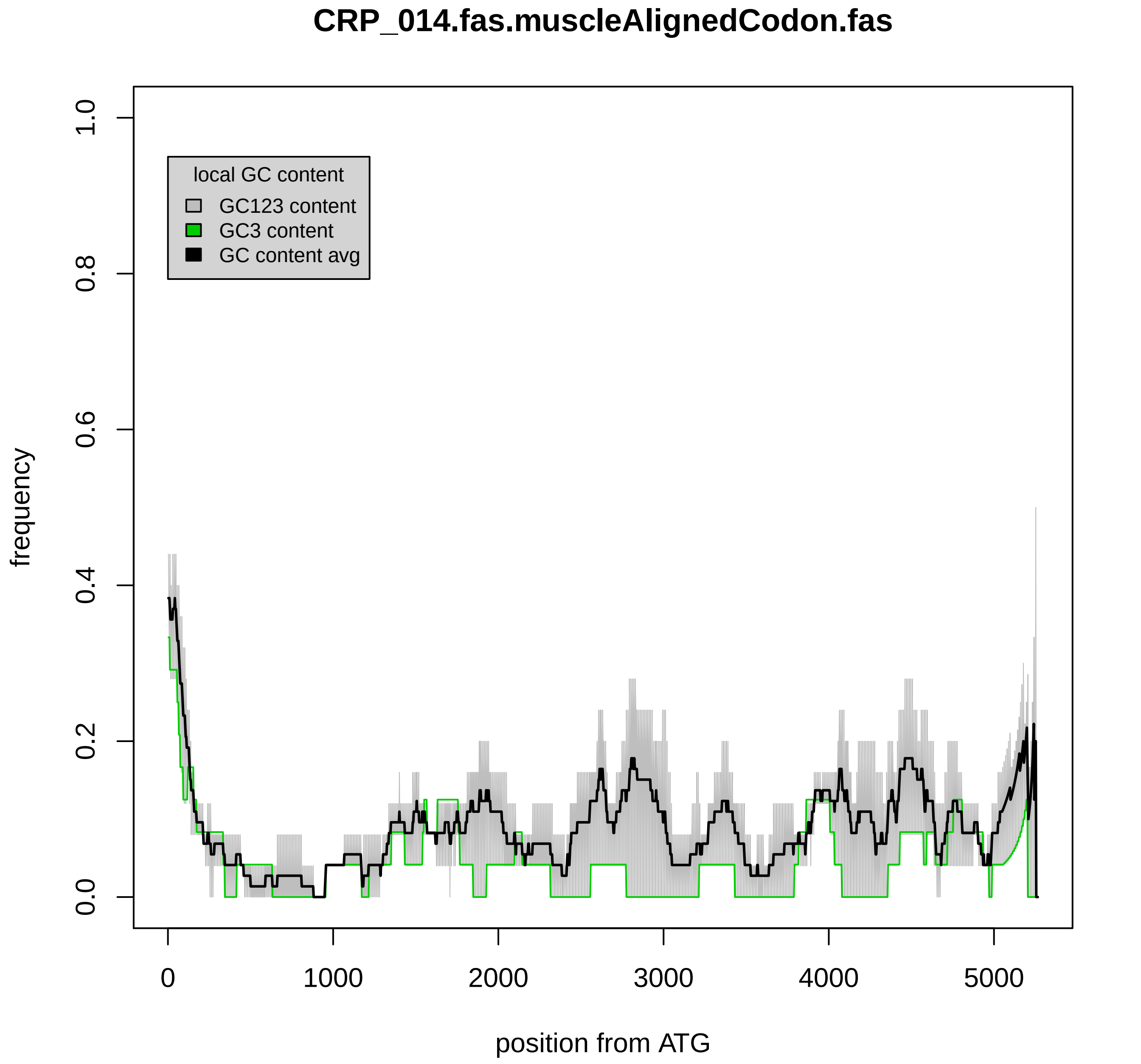

CRP_014.fas.muscleAlignedCodon.fas
local GC content
GC123 content
GC3 content
GC content avg
frequency
0.0
0.2
0.4
0.6
0.8
1.0
0
1000
2000
3000
4000
5000
position from ATG

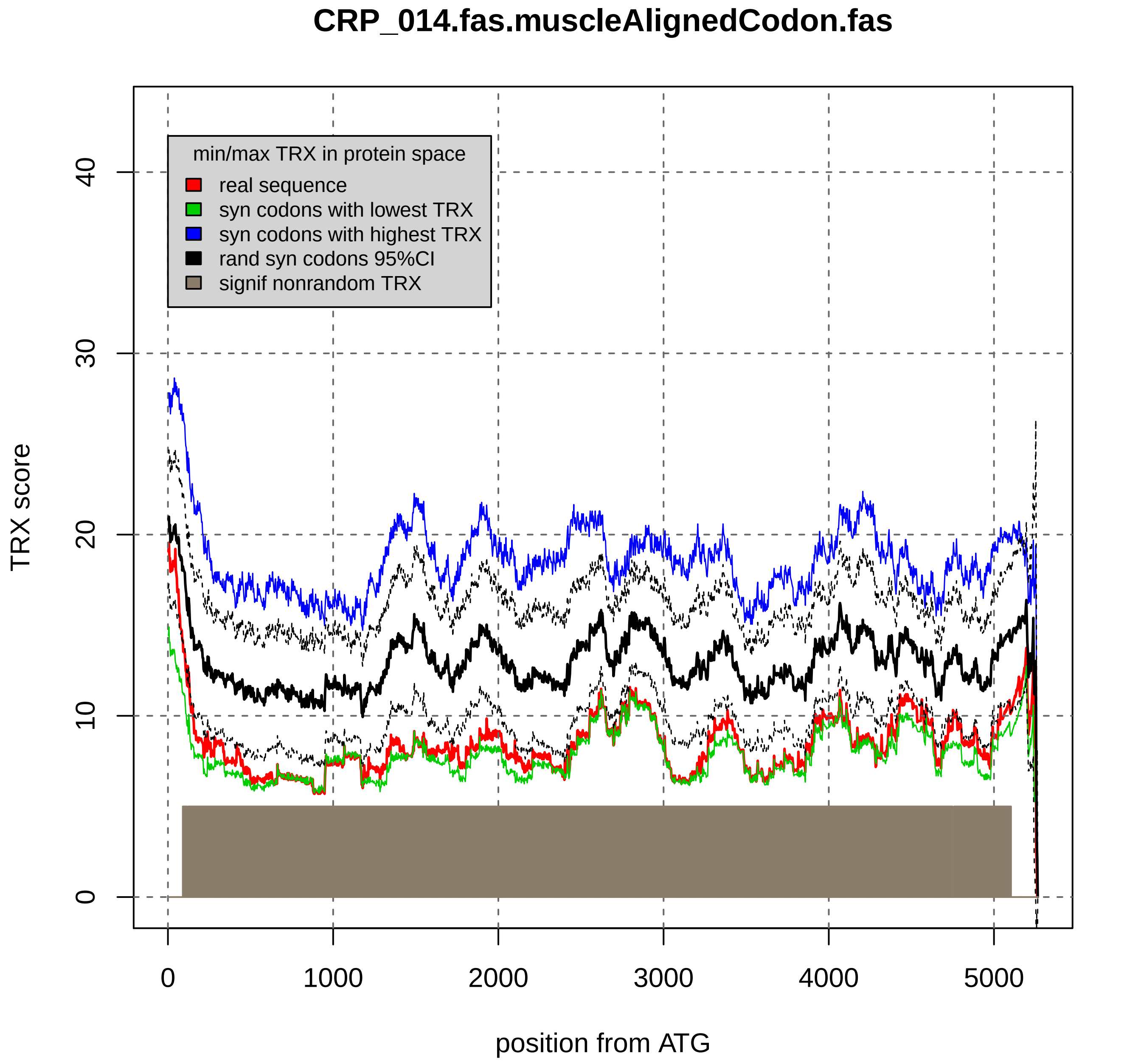

CRP_014.fas.muscleAlignedCodon.fas
min/max TRX in protein space
real sequence
syn codons with lowest TRX
syn codons with highest TRX
rand syn codons 95%CI
signif nonrandom TRX
TRX score
0
10
20
30
40
0
1000
2000
3000
4000
5000
position from ATG

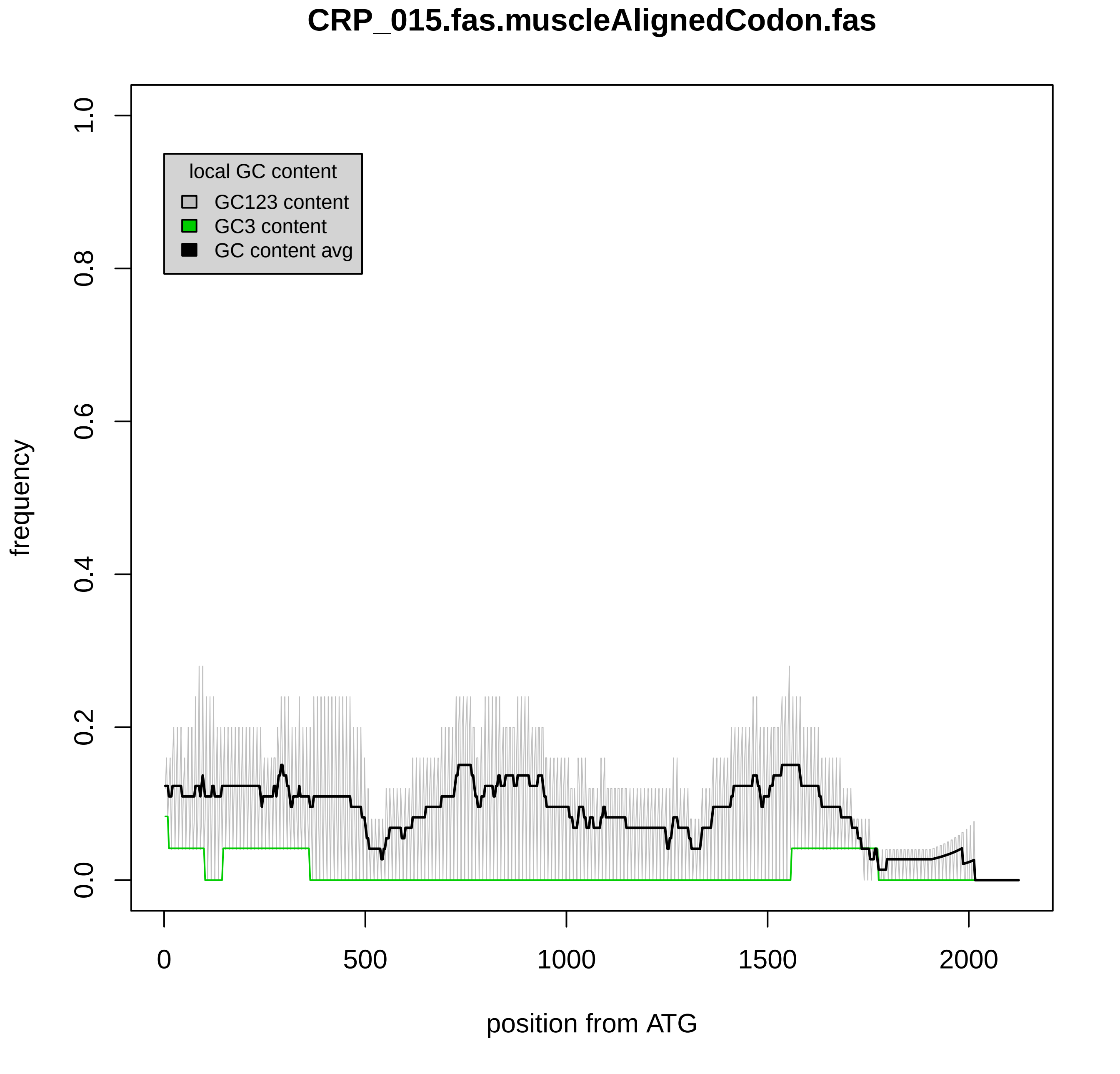
CRP_015.fas.muscleAlignedCodon.fas
local GC content
GC123 content
GC3 content
GC content avg
frequency
0.0
0.2
0.4
0.6
0.8
1.0
0
500
1000
1500
2000
position from ATG

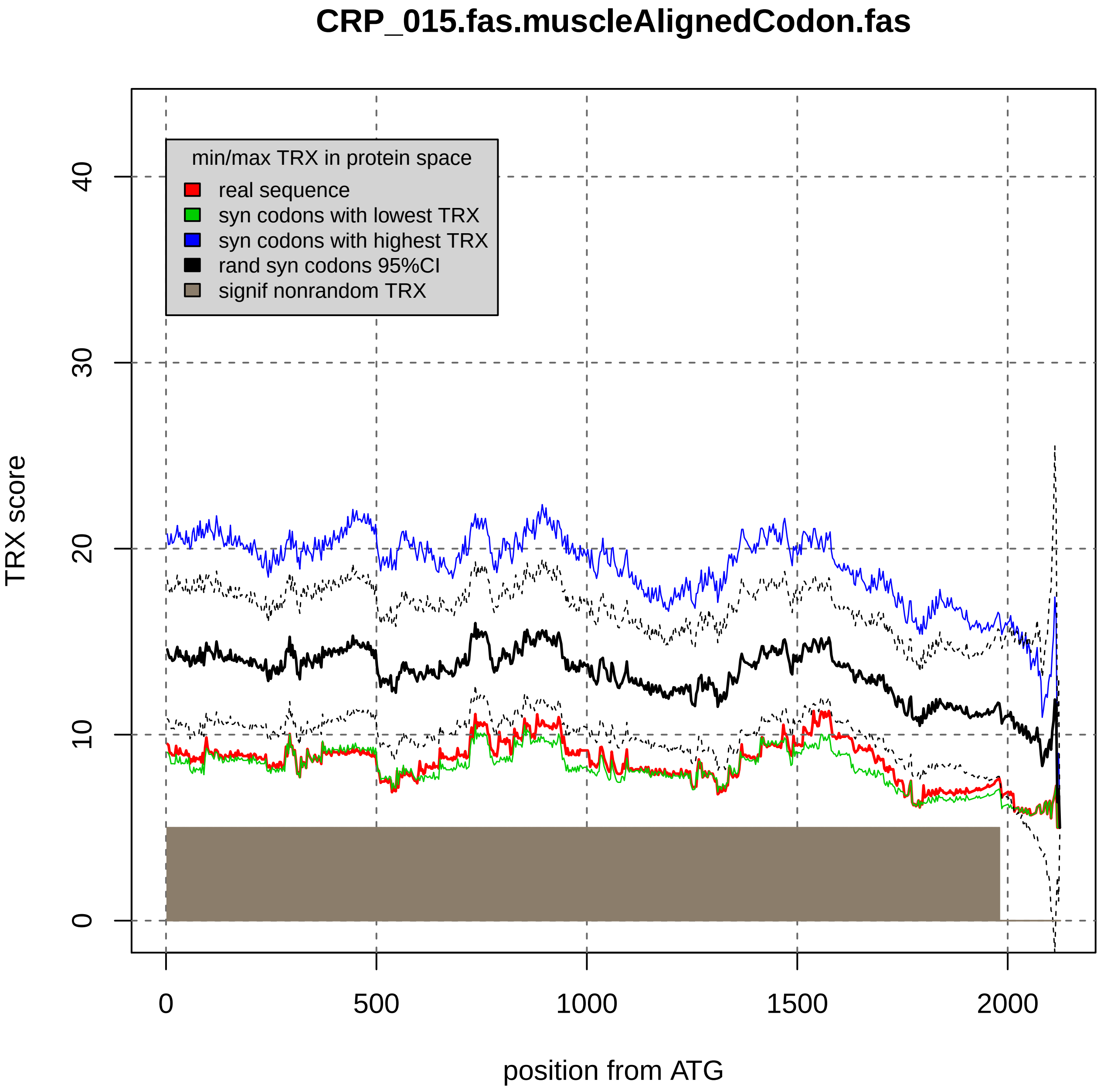

CRP_015.fas.muscleAlignedCodon.fas
min/max TRX in protein space
real sequence
syn codons with lowest TRX
syn codons with highest TRX
rand syn codons 95%CI
signif nonrandom TRX
TRX score
0
10
20
30
40
0
500
1000
1500
2000
position from ATG

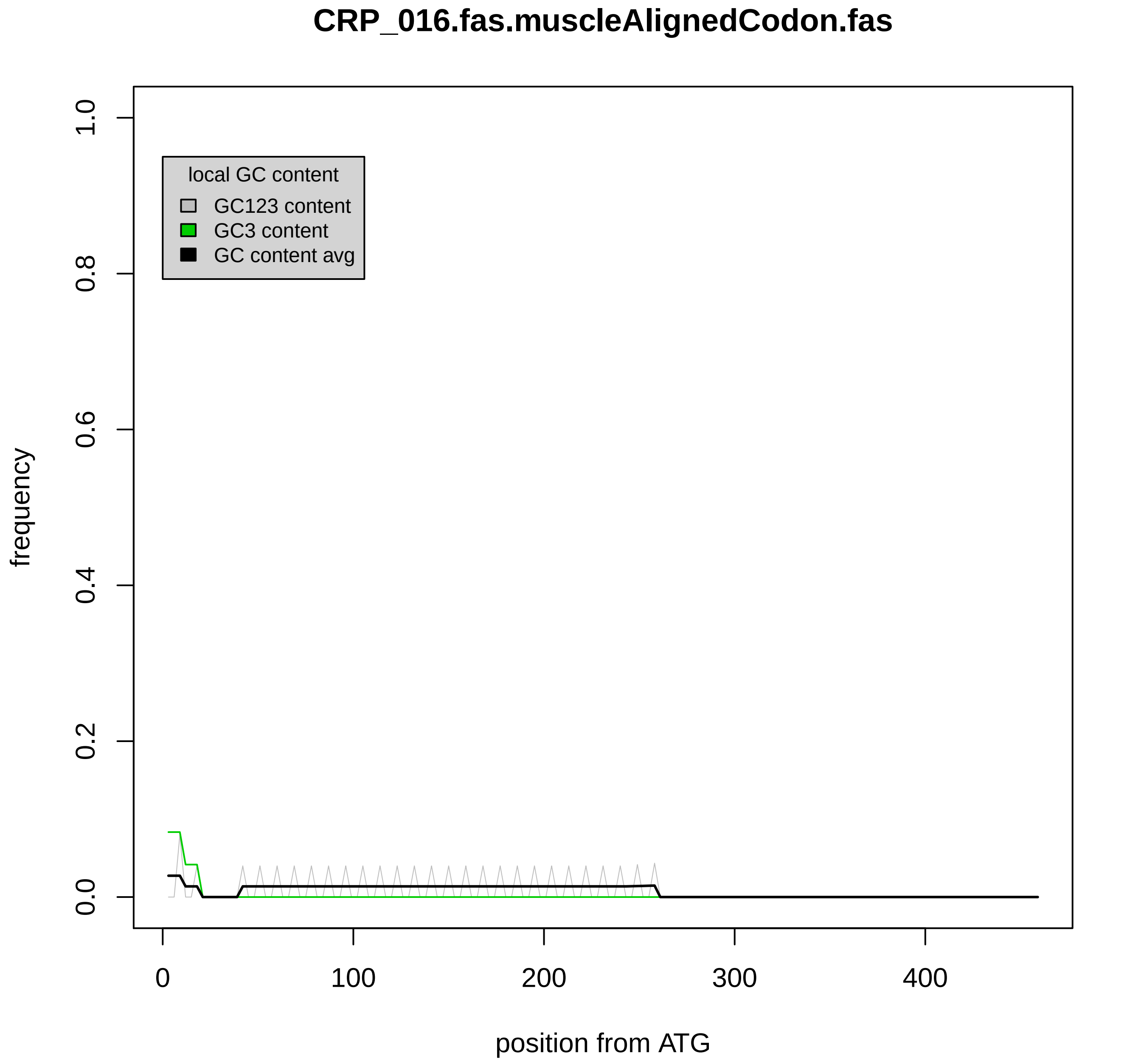
CRP_016.fas.muscleAlignedCodon.fas
local GC content
GC123 content
GC3 content
GC content avg
frequency
0.0
0.2
0.4
0.6
0.8
1.0
0
100
200
300
400
position from ATG

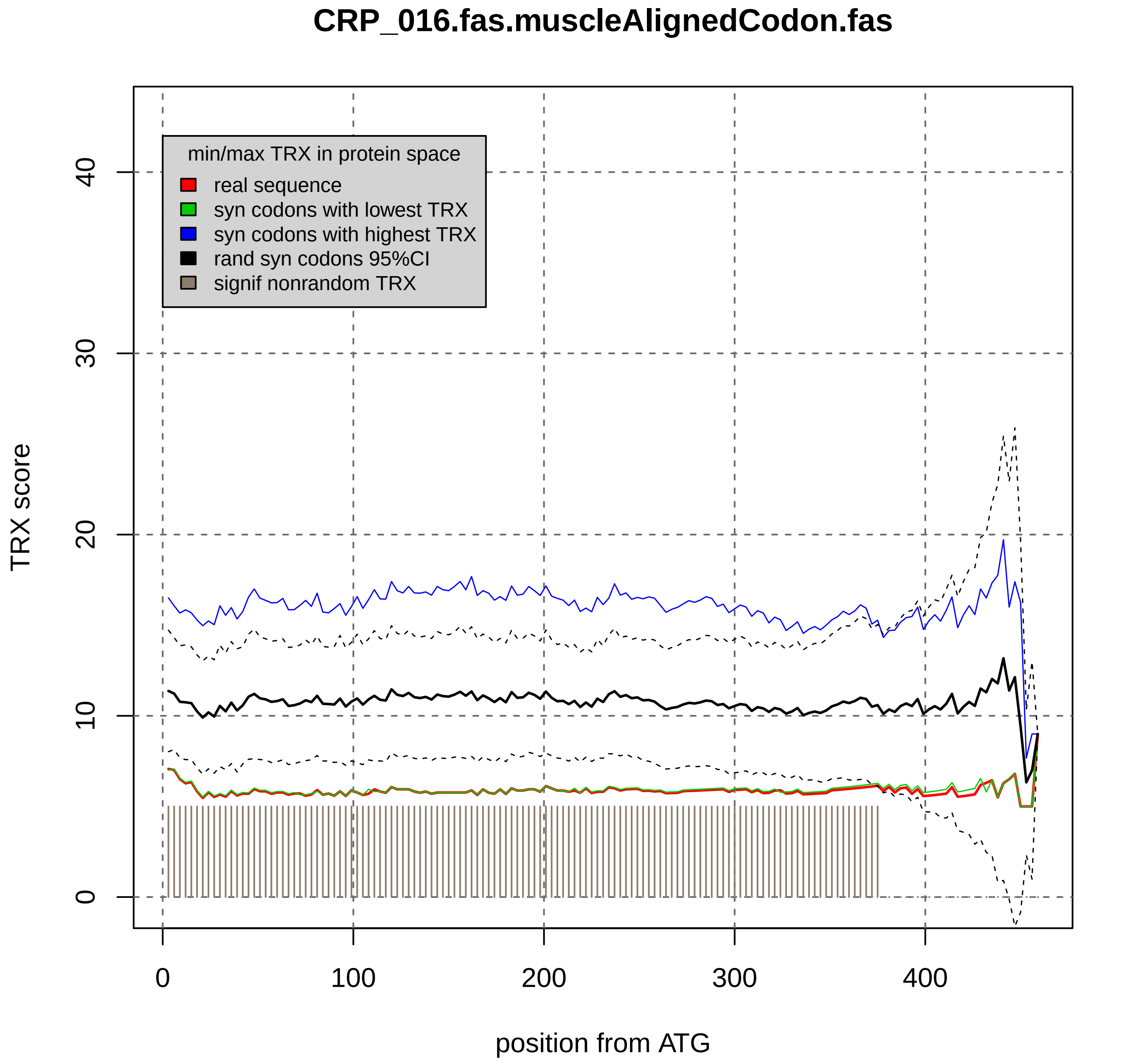

CRP_016.fas.muscleAlignedCodon.fas
min/max TRX in protein space
real sequence
syn codons with lowest TRX
syn codons with highest TRX
rand syn codons 95%CI
signif nonrandom TRX
TRX score
0
10
20
30
40
0
100
200
300
400
position from ATG

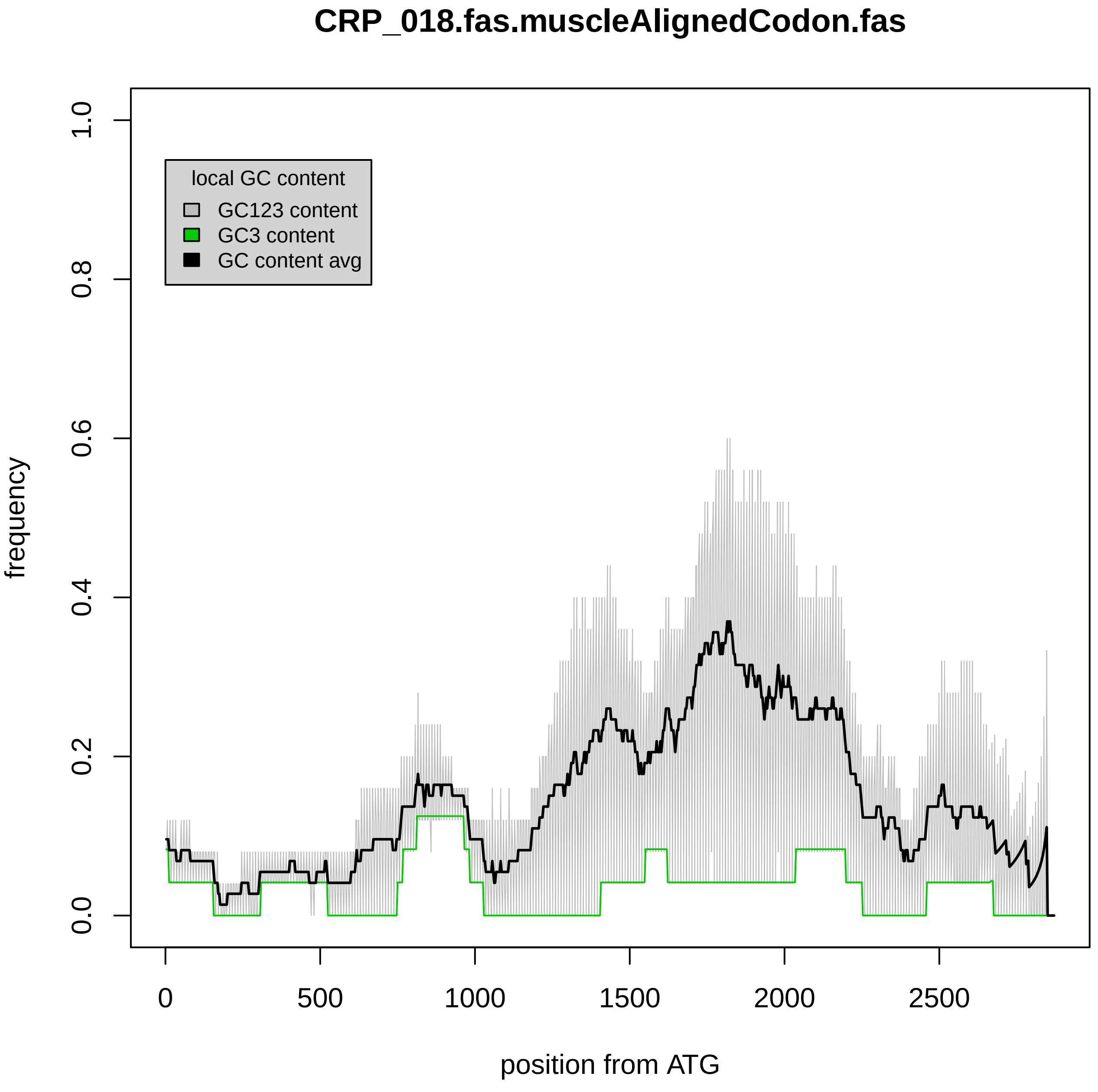

CRP_018.fas.muscleAlignedCodon.fas
local GC content
GC123 content
GC3 content
GC content avg
frequency
0.0
0.2
0.4
0.6
0.8
1.0
0
500
1000
1500
2000
2500
position from ATG

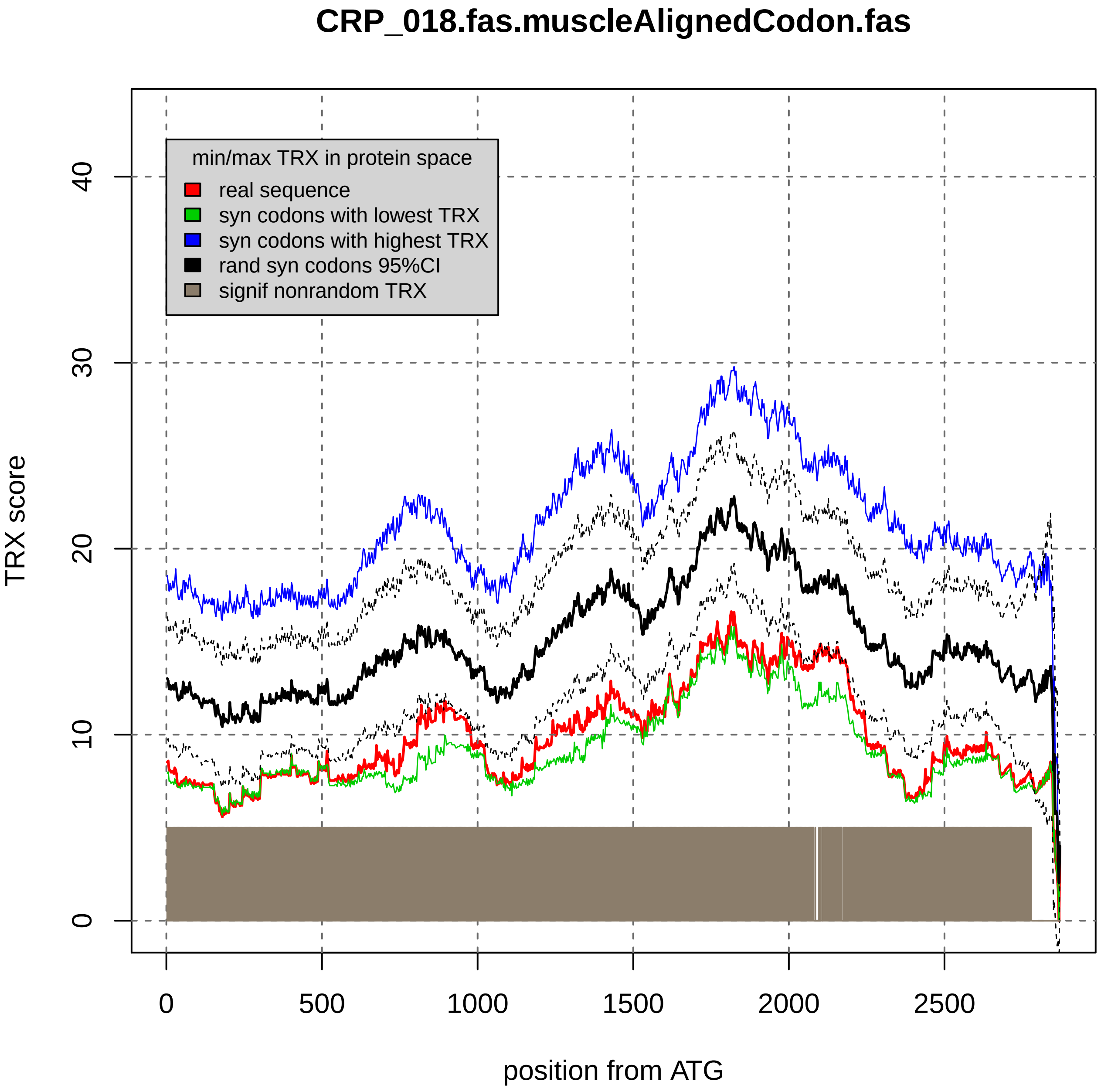

CRP_018.fas.muscleAlignedCodon.fas
min/max TRX in protein space
real sequence
syn codons with lowest TRX
syn codons with highest TRX
rand syn codons 95%CI
signif nonrandom TRX
TRX score
0
10
20
30
40
0
500
1000
1500
2000
2500
position from ATG

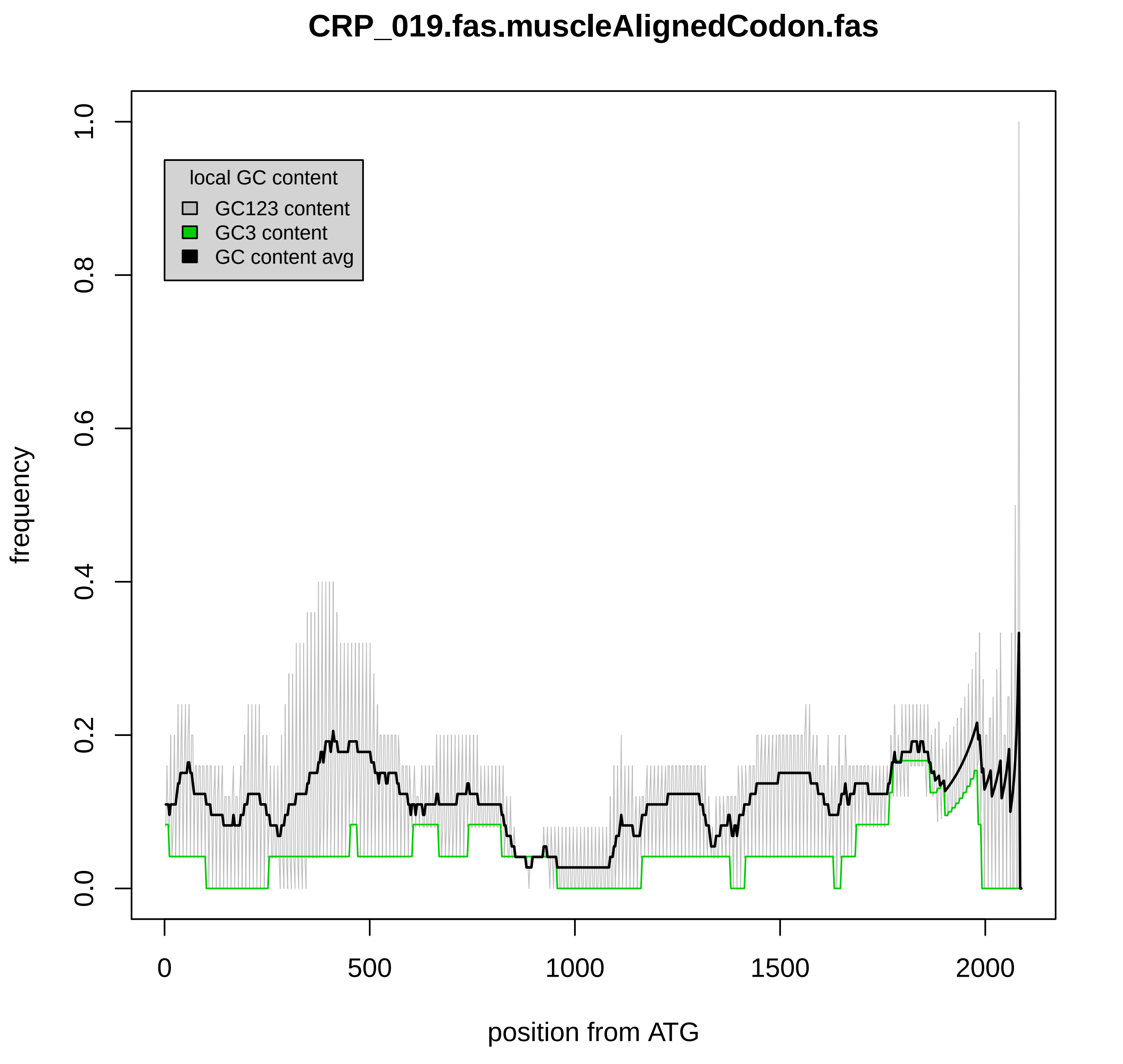
CRP_019.fas.muscleAlignedCodon.fas
local GC content
GC123 content
GC3 content
GC content avg
frequency
position from ATG
0.0
0.2
0.4
0.6
0.8
1.0
0
500
1000
1500
2000

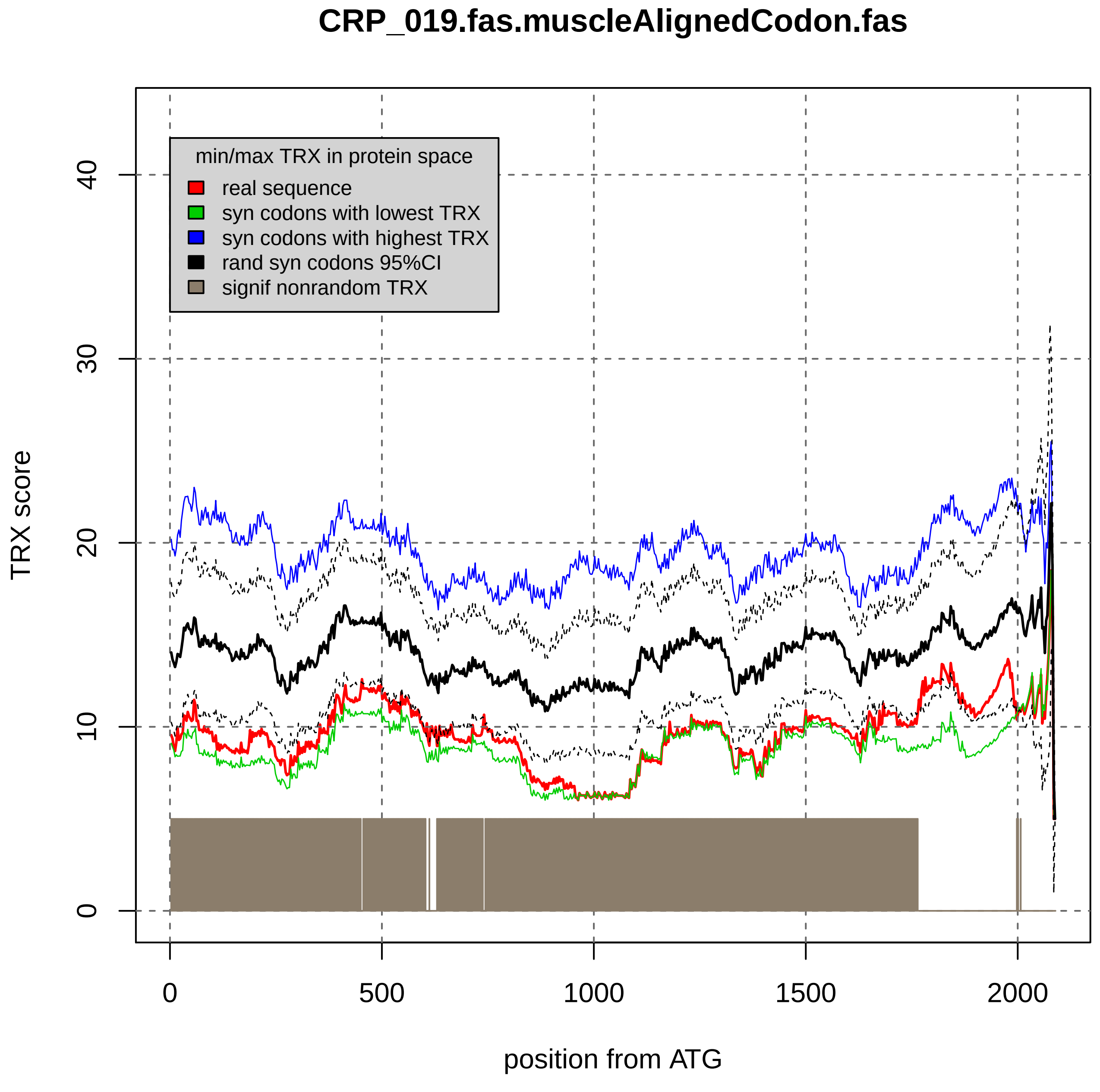

CRP_019.fas.muscleAlignedCodon.fas
min/max TRX in protein space
real sequence
syn codons with lowest TRX
syn codons with highest TRX
rand syn codons 95%CI
signif nonrandom TRX
TRX score
0
10
20
30
40
0
500
1000
1500
2000
position from ATG

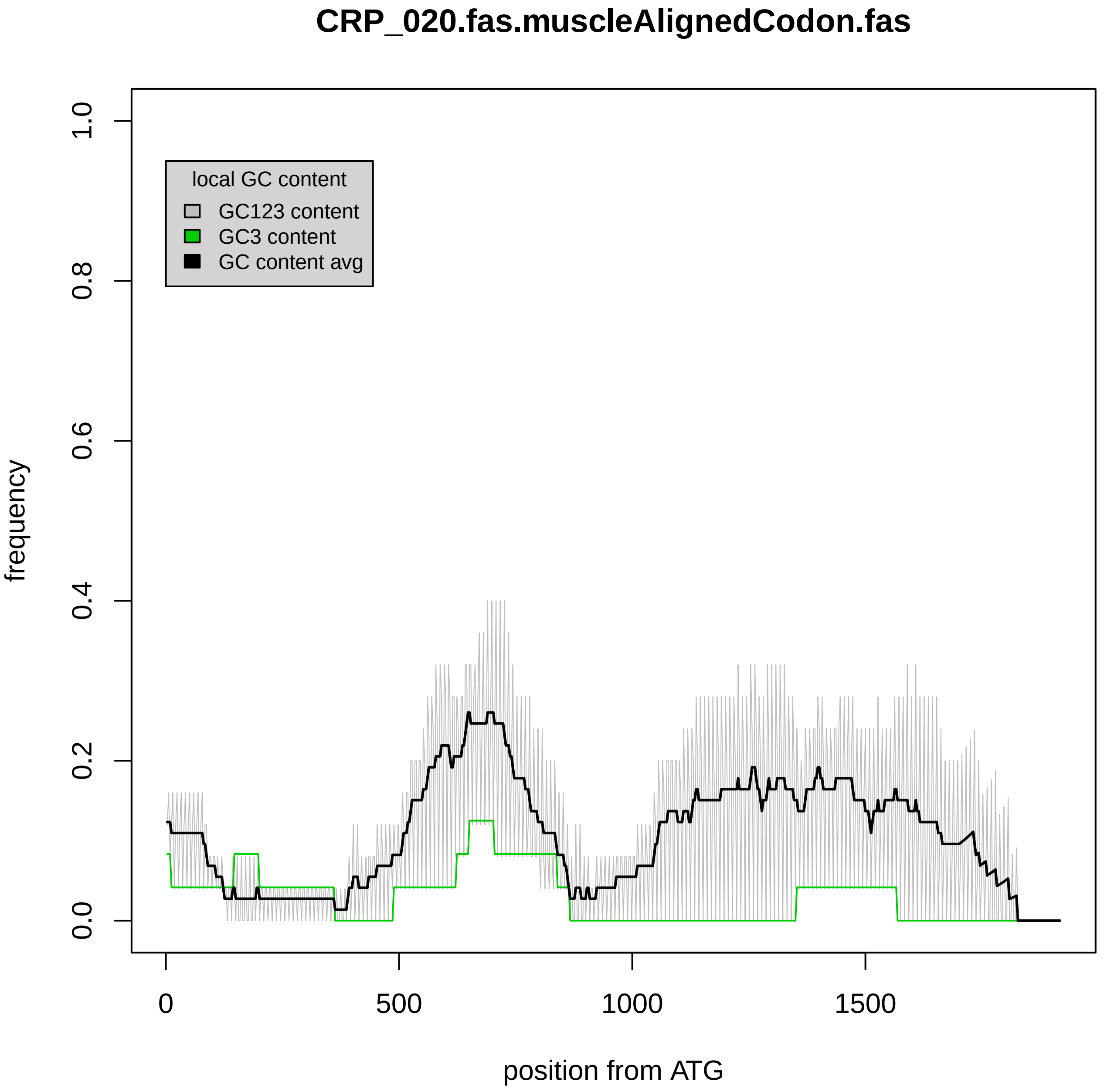

CRP_020.fas.muscleAlignedCodon.fas
local GC content
GC123 content
GC3 content
GC content avg
frequency
0.0
0.2
0.4
0.6
0.8
1.0
0
500
1000
1500
position from ATG

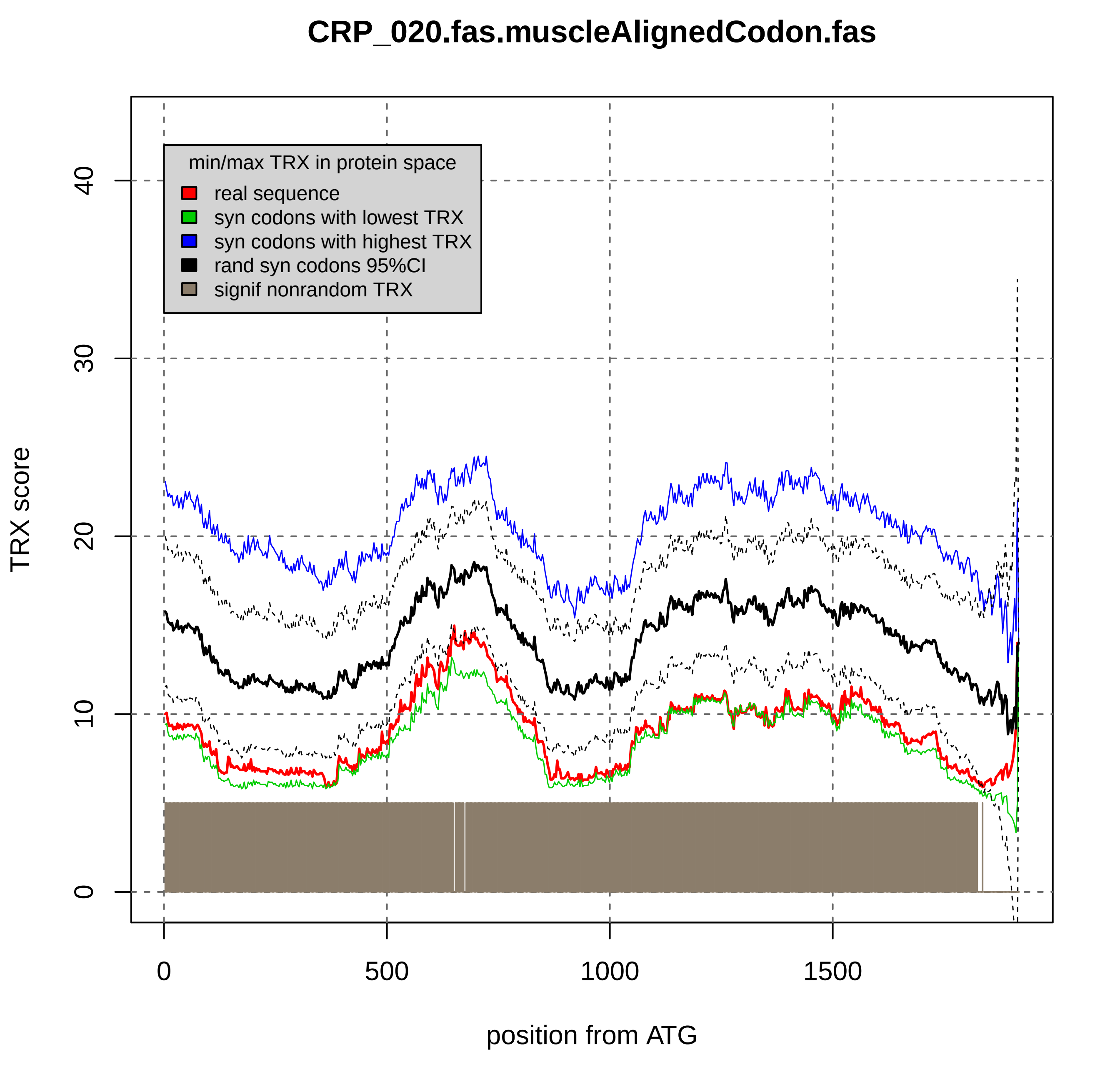

CRP_020.fas.muscleAlignedCodon.fas
min/max TRX in protein space
real sequence
syn codons with lowest TRX
syn codons with highest TRX
rand syn codons 95%CI
signif nonrandom TRX
TRX score
0
10
20
30
40
0
500
1000
1500
position from ATG
