# Supplementary material for: Synonymous codon bias and functional constraint on GC3-related DNA backbone dynamics in the prokaryotic nucleoid
Source: Nucleic Acids Res. 2014 Sep 8;42(17):10915–26. doi: 10.1093/nar/gku811 (PMC4176184; doi:10.1093/nar/gku811)
Supplement: SUPPLEMENTARY DATA [file supp_gku811_nar-01772-z-2014-File009.zip › NAR-01772-Z-2014.R1 Suppl files/SuppFileD_caption.pdf]

Supplementary File D - Fundamental constraints between protein evolution (dN/dS) and mutational impacts on intrinsic DNA flexibility (dTRX) or genome architecture. All species analyzed are shown here Within each plot set, genes functionally conserved at the protein-level (i.e. low dN/dS) are shown in black, while genes adaptively altered at protein level are shown colored. Mutational impacts on flexibility (dTRX) are shown separately for synonymous sites (left side of plot set) and non-synonymous sites (right side of plot set). dTRX for transitions and transversions are separated in the upper plots of each set.

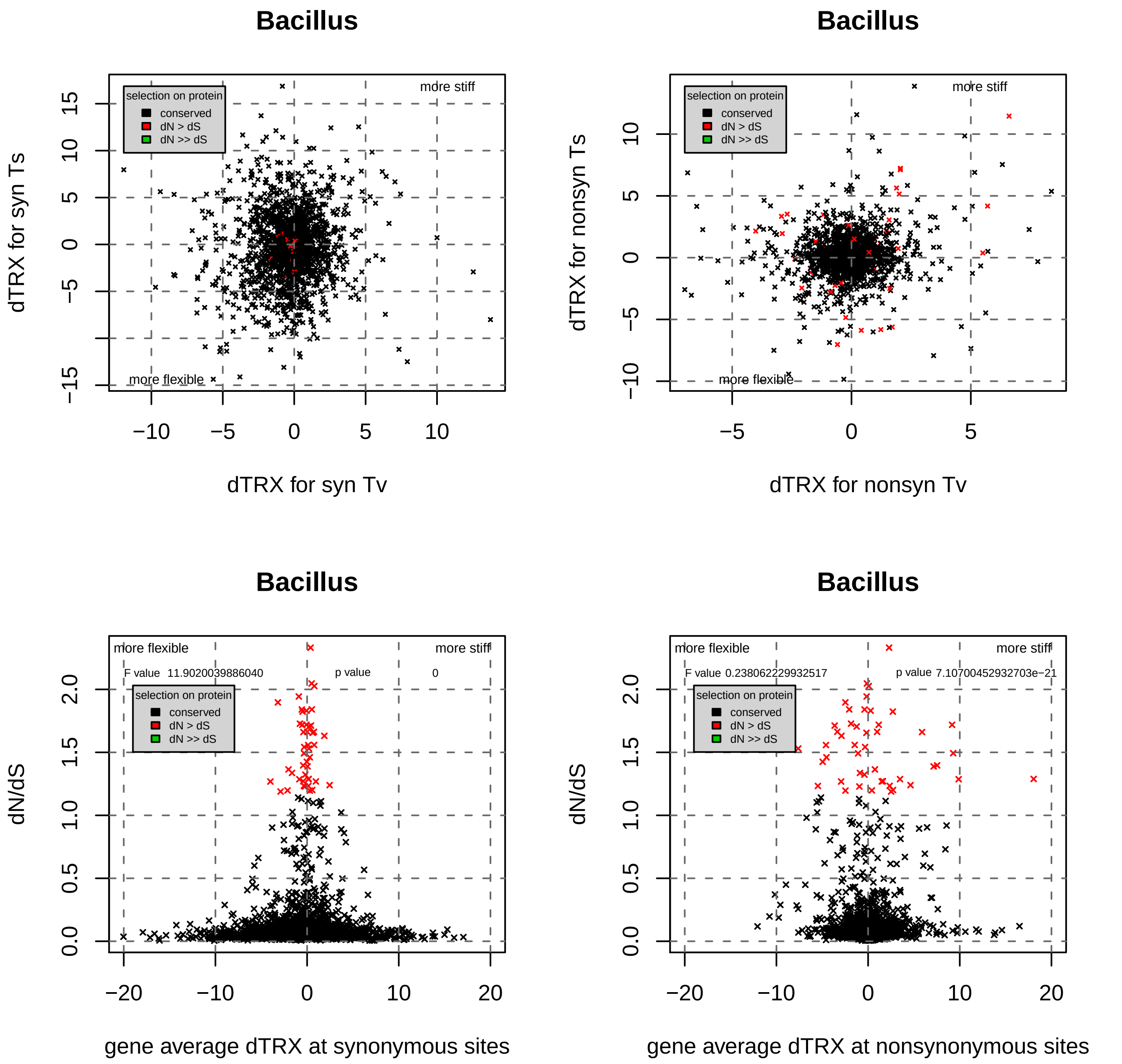
Bacillus
selection on protein
conserved
dN > dS
dN >> dS
more stiff
more flexible
dTRX for syn Ts
dTRX for syn Tv
Bacillus
selection on protein
conserved
dN > dS
dN >> dS
more stiff
more flexible
dTRX for nonsyn Ts
dTRX for nonsyn Tv
Bacillus
more flexible
more stiff
F value 11.9020039886040
p value 0
selection on protein
conserved
dN > dS
dN >> dS
dN/dS
gene average dTRX at synonymous sites
Bacillus
more flexible
more stiff
F value 0.238062229932517
p value 7.10700452932703e−21
selection on protein
conserved
dN > dS
dN >> dS
dN/dS
gene average dTRX at nonsynonymous sites

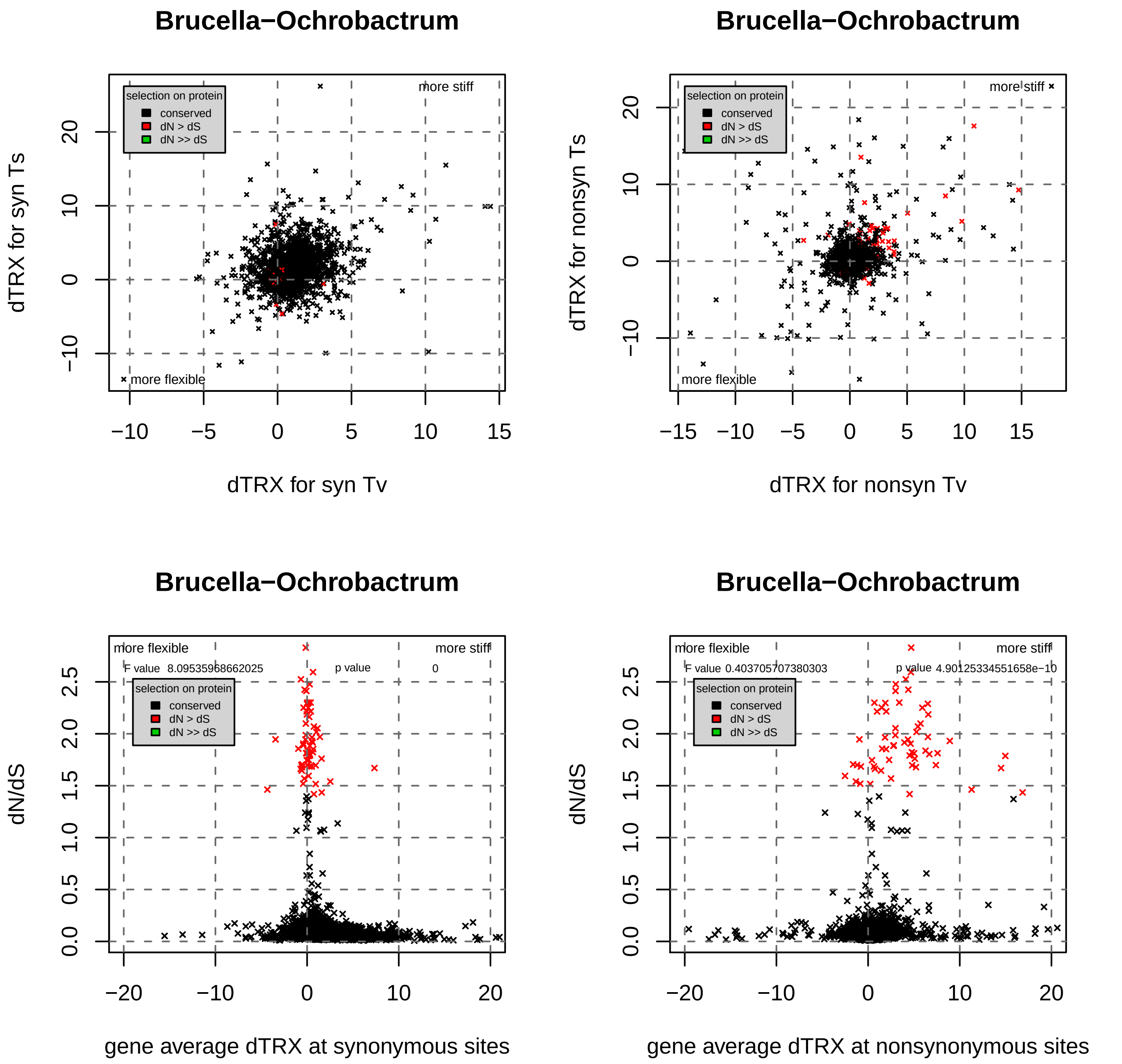

Brucella−Ochrobactrum
selection on protein
conserved
dN > dS
dN >> dS
more stiff
more flexible
dTRX for syn Ts
dTRX for syn Tv
Brucella−Ochrobactrum
selection on protein
conserved
dN > dS
dN >> dS
more stiff
more flexible
dTRX for nonsyn Ts
dTRX for nonsyn Tv
Brucella−Ochrobactrum
more flexible
more stiff
F value 8.09535968662025
p value
0
selection on protein
conserved
dN > dS
dN >> dS
dN/dS
gene average dTRX at synonymous sites
Brucella−Ochrobactrum
more flexible
more stiff
F value 0.403705707380303
p value 4.90125334551658e−10
selection on protein
conserved
dN > dS
dN >> dS
dN/dS
gene average dTRX at nonsynonymous sites

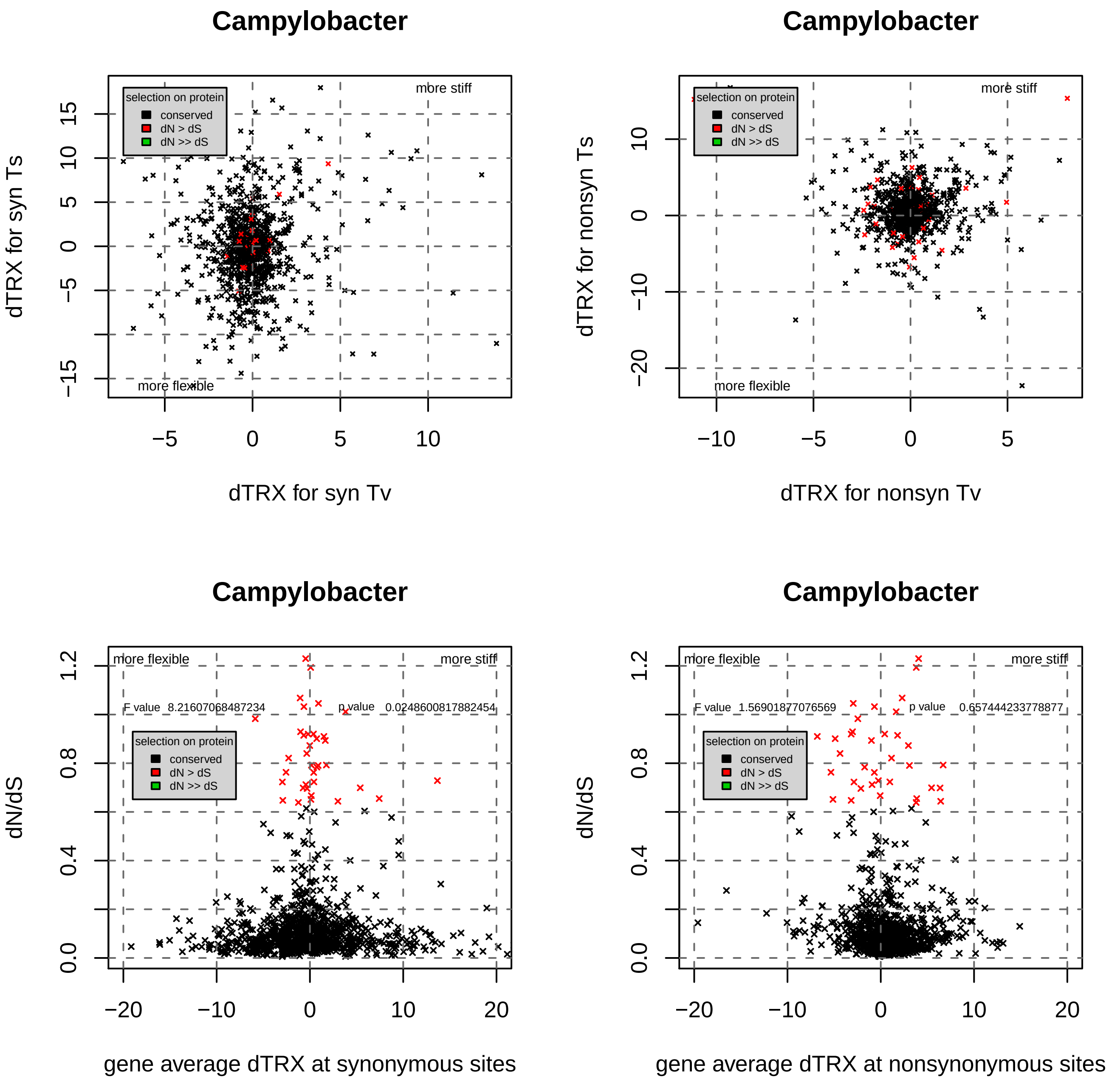

Campylobacter
selection on protein
conserved
dN > dS
dN >> dS
more stiff
more flexible
dTRX for syn Ts
dTRX for syn Tv
Campylobacter
selection on protein
conserved
dN > dS
dN >> dS
more stiff
more flexible
dTRX for nonsyn Ts
dTRX for nonsyn Tv
Campylobacter
more flexible
more stiff
F value 8.21607068487234
p value 0.0248600817882454
selection on protein
conserved
dN > dS
dN >> dS
dN/dS
gene average dTRX at synonymous sites
Campylobacter
more flexible
more stiff
F value 1.56901877076569
p value 0.657444233778877
selection on protein
conserved
dN > dS
dN >> dS
dN/dS
gene average dTRX at nonsynonymous sites

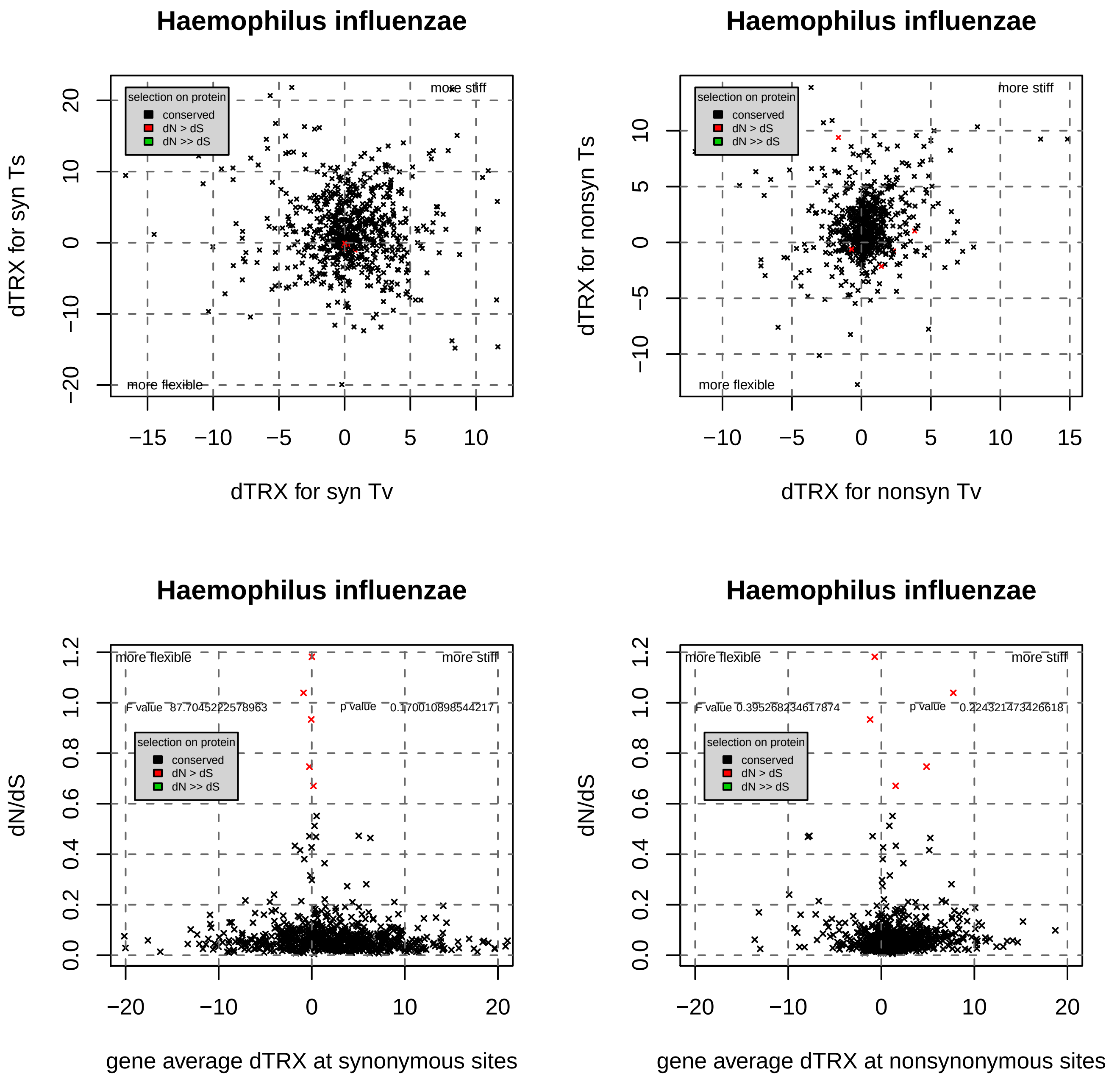
Haemophilus influenzae
selection on protein
conserved
dN > dS
dN >> dS
more stiff
more flexible
dTRX for syn Ts
dTRX for syn Tv
Haemophilus influenzae
selection on protein
conserved
dN > dS
dN >> dS
more stiff
more flexible
dTRX for nonsyn Ts
dTRX for nonsyn Tv
Haemophilus influenzae
more flexible
more stiff
F value 87.7045222578963
p value 0.170010898544217
selection on protein
conserved
dN > dS
dN >> dS
dN/dS
gene average dTRX at synonymous sites
Haemophilus influenzae
more flexible
more stiff
F value 0.395268234617874
p value 0.224321473426618
selection on protein
conserved
dN > dS
dN >> dS
dN/dS
gene average dTRX at nonsynonymous sites

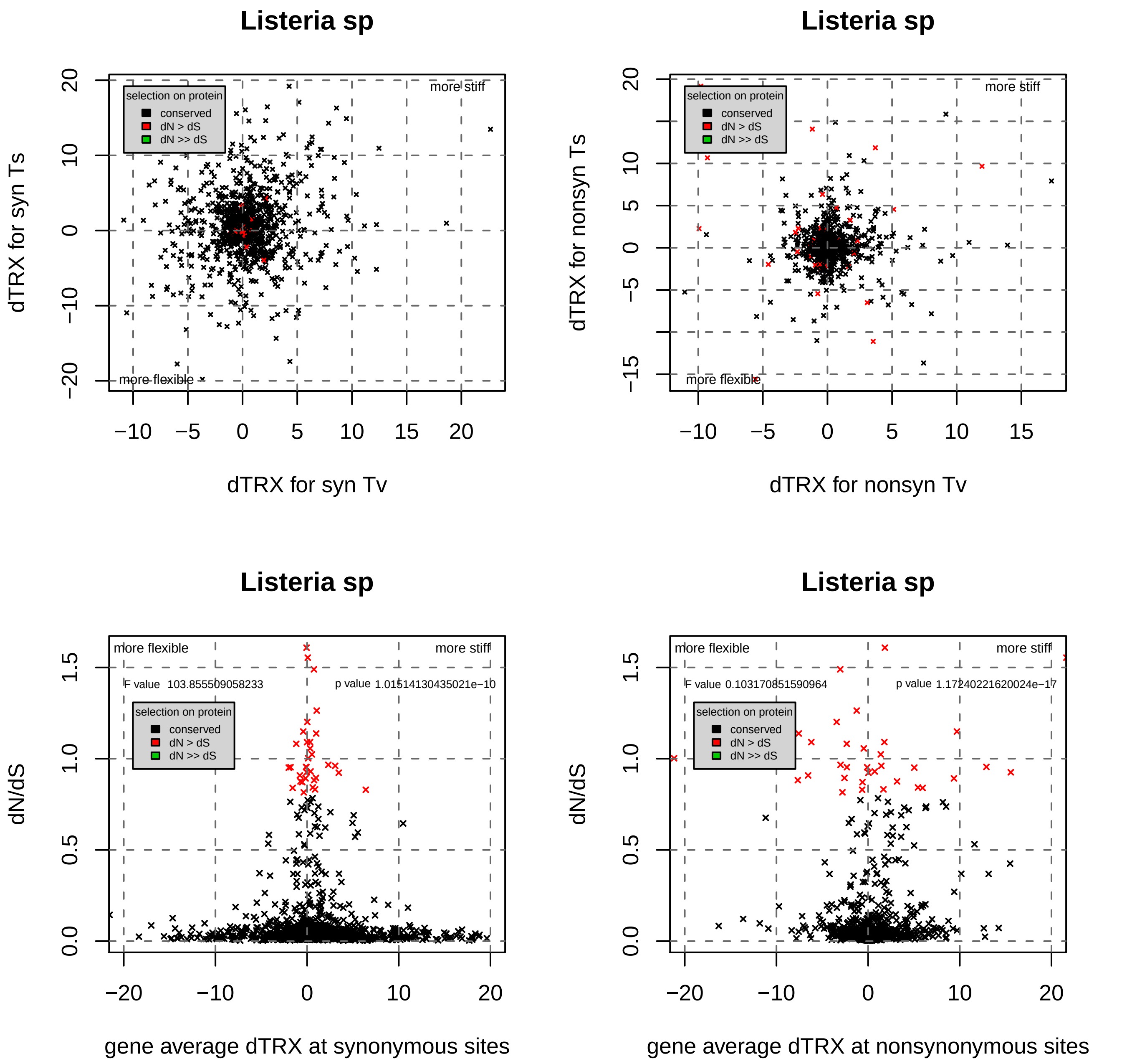
Listeria sp
dTRX for syn Ts
dTRX for syn Tv
more stiff
more flexible
selection on protein
conserved
dN > dS
dN >> dS
Listeria sp
dTRX for nonsyn Ts
dTRX for nonsyn Tv
more stiff
more flexible
selection on protein
conserved
dN > dS
dN >> dS
Listeria sp
dN/dS
gene average dTRX at synonymous sites
more flexible
more stiff
F value 103.855509058233
p value 1.01514130435021e−10
selection on protein
conserved
dN > dS
dN >> dS
Listeria sp
dN/dS
gene average dTRX at nonsynonymous sites
more flexible
more stiff
F value 0.103170851590964
p value 1.17240221620024e−17
selection on protein
conserved
dN > dS
dN >> dS

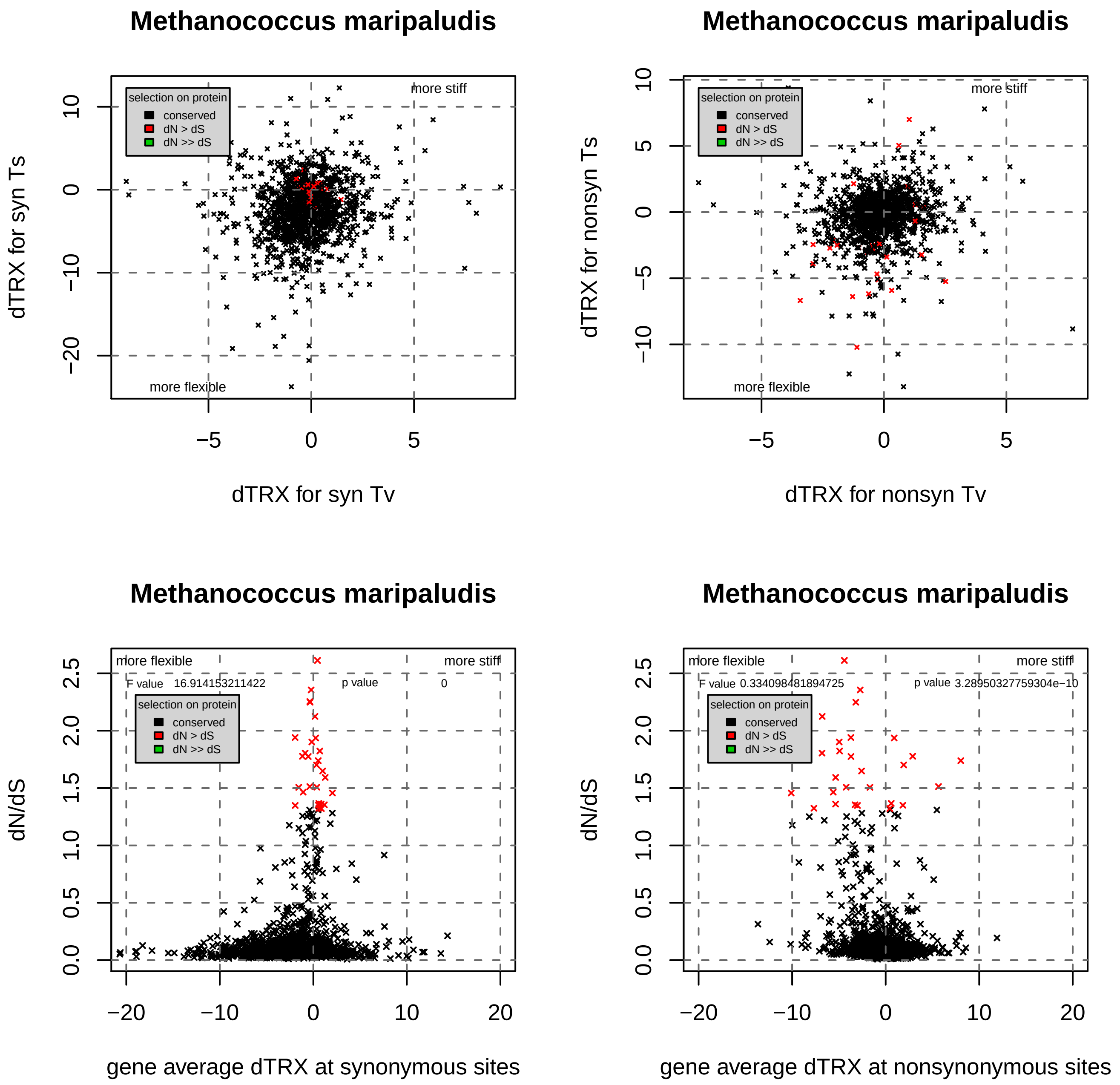
Methanococcus maripaludis
selection on protein
conserved
dN > dS
dN >> dS
more stiff
more flexible
dTRX for syn Ts
dTRX for syn Tv
Methanococcus maripaludis
selection on protein
conserved
dN > dS
dN >> dS
more stiff
more flexible
dTRX for nonsyn Ts
dTRX for nonsyn Tv
Methanococcus maripaludis
more flexible
more stiff
F value 16.914153211422
p value 0
selection on protein
conserved
dN > dS
dN >> dS
dN/dS
gene average dTRX at synonymous sites
Methanococcus maripaludis
more flexible
more stiff
F value 0.334098481894725
p value 3.28950327759304e−10
selection on protein
conserved
dN > dS
dN >> dS
dN/dS
gene average dTRX at nonsynonymous sites

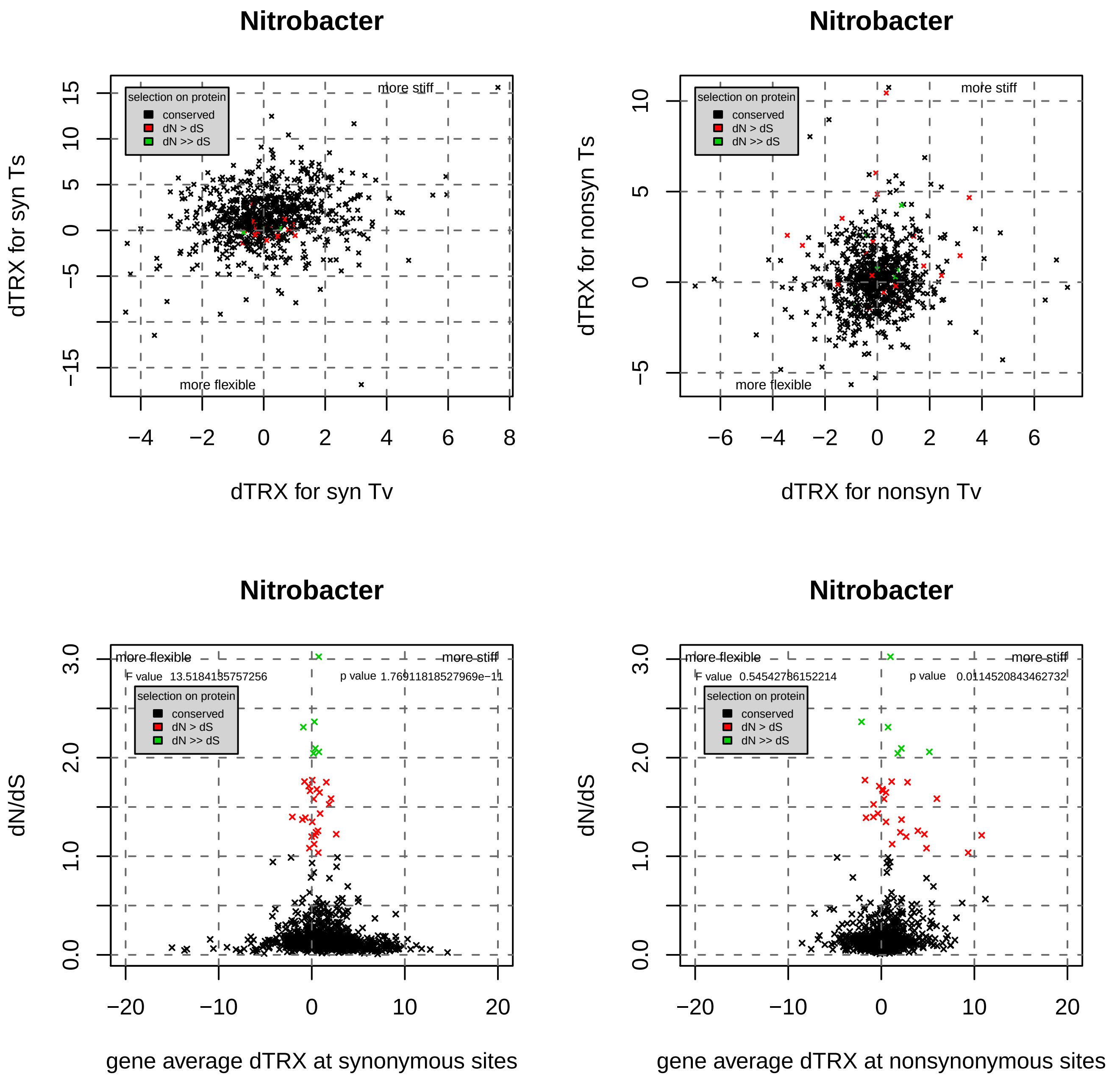
Nitrobacter
selection on protein
conserved
dN > dS
dN >> dS
more stiff
more flexible
dTRX for syn Ts
dTRX for syn Tv
Nitrobacter
selection on protein
conserved
dN > dS
dN >> dS
more stiff
more flexible
dTRX for nonsyn Ts
dTRX for nonsyn Tv
Nitrobacter
more flexible
more stiff
F value 13.5184135757256
p value 1.76911818527969e−11
selection on protein
conserved
dN > dS
dN >> dS
dN/dS
gene average dTRX at synonymous sites
Nitrobacter
more flexible
more stiff
F value 0.54542786152214
p value 0.0114520843462732
selection on protein
conserved
dN > dS
dN >> dS
dN/dS
gene average dTRX at nonsynonymous sites

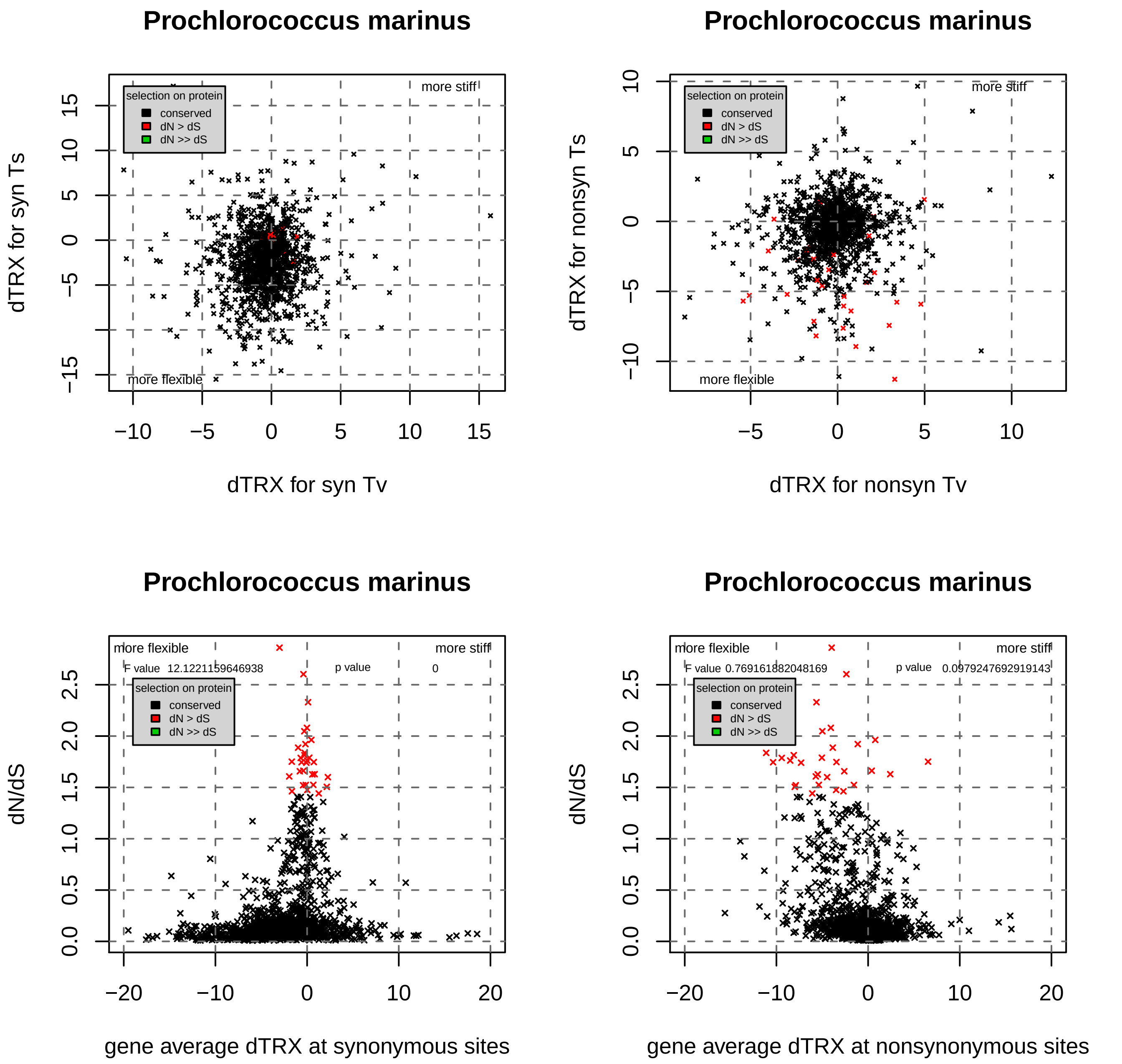
Prochlorococcus marinus
selection on protein
conserved
dN > dS
dN >> dS
more stiff
more flexible
dTRX for syn Ts
dTRX for syn Tv
Prochlorococcus marinus
selection on protein
conserved
dN > dS
dN >> dS
more stiff
more flexible
dTRX for nonsyn Ts
dTRX for nonsyn Tv
Prochlorococcus marinus
more flexible
more stiff
F value 12.1221159646938
p value
0
selection on protein
conserved
dN > dS
dN >> dS
dN/dS
gene average dTRX at synonymous sites
Prochlorococcus marinus
more flexible
more stiff
F value 0.769161882048169
p value 0.0979247692919143
selection on protein
conserved
dN > dS
dN >> dS
dN/dS
gene average dTRX at nonsynonymous sites

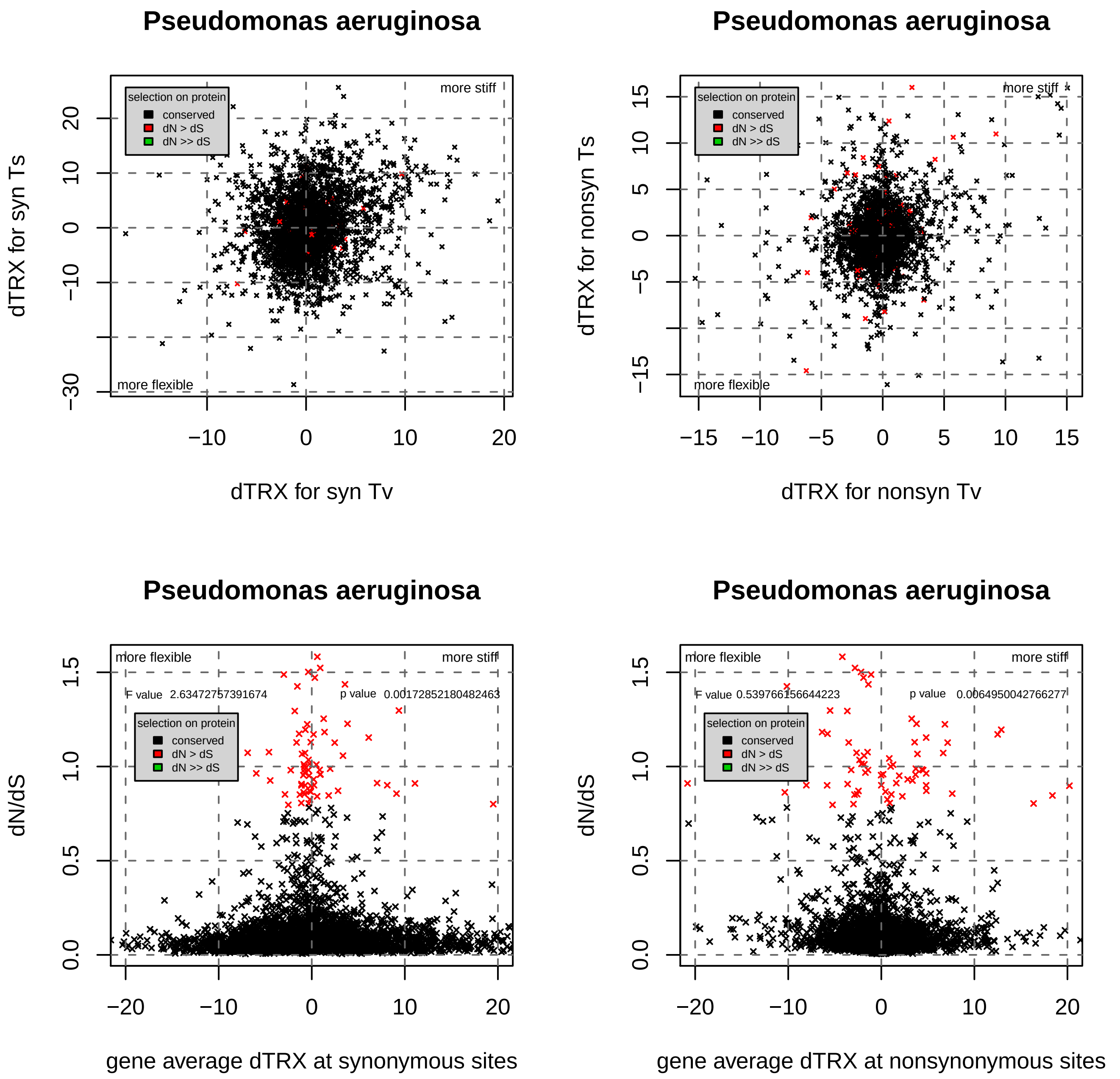
Pseudomonas aeruginosa
selection on protein
conserved
dN > dS
dN >> dS
more stiff
more flexible
dTRX for syn Ts
dTRX for syn Tv
Pseudomonas aeruginosa
selection on protein
conserved
dN > dS
dN >> dS
more stiff
more flexible
dTRX for nonsyn Ts
dTRX for nonsyn Tv
Pseudomonas aeruginosa
more flexible
more stiff
F value 2.63472757391674
p value 0.00172852180482463
selection on protein
conserved
dN > dS
dN >> dS
dN/dS
gene average dTRX at synonymous sites
Pseudomonas aeruginosa
more flexible
more stiff
F value 0.539766156644223
p value 0.0064950042766277
selection on protein
conserved
dN > dS
dN >> dS
dN/dS
gene average dTRX at nonsynonymous sites

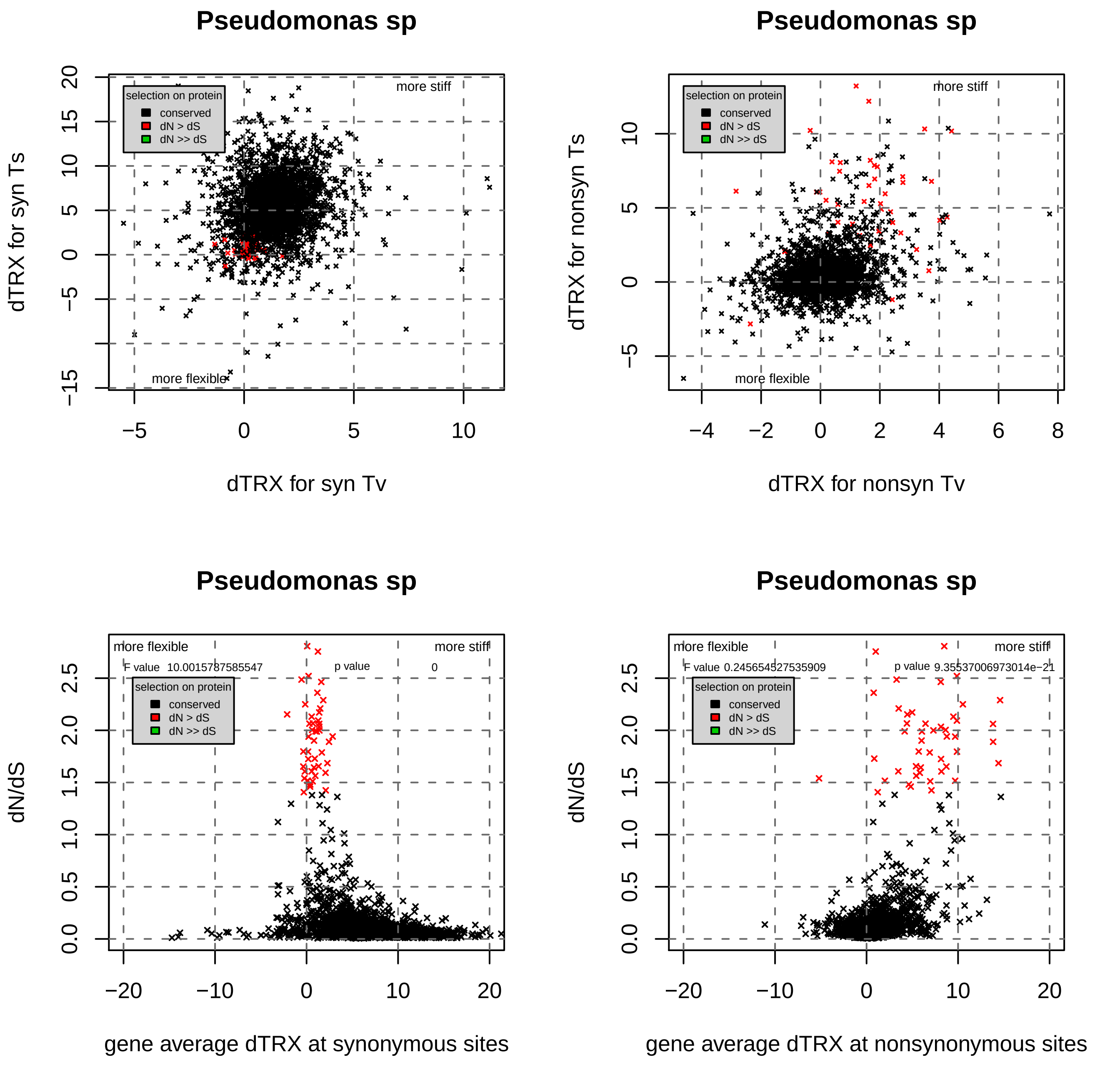
Pseudomonas sp
selection on protein
conserved
dN > dS
dN >> dS
more stiff
more flexible
dTRX for syn Ts
dTRX for syn Tv
Pseudomonas sp
selection on protein
conserved
dN > dS
dN >> dS
more stiff
more flexible
dTRX for nonsyn Ts
dTRX for nonsyn Tv
Pseudomonas sp
more flexible
more stiff
F value 10.0015787585547
p value
0
selection on protein
conserved
dN > dS
dN >> dS
dN/dS
gene average dTRX at synonymous sites
Pseudomonas sp
more flexible
more stiff
F value 0.245654527535909
p value 9.35537006973014e−21
selection on protein
conserved
dN > dS
dN >> dS
dN/dS
gene average dTRX at nonsynonymous sites

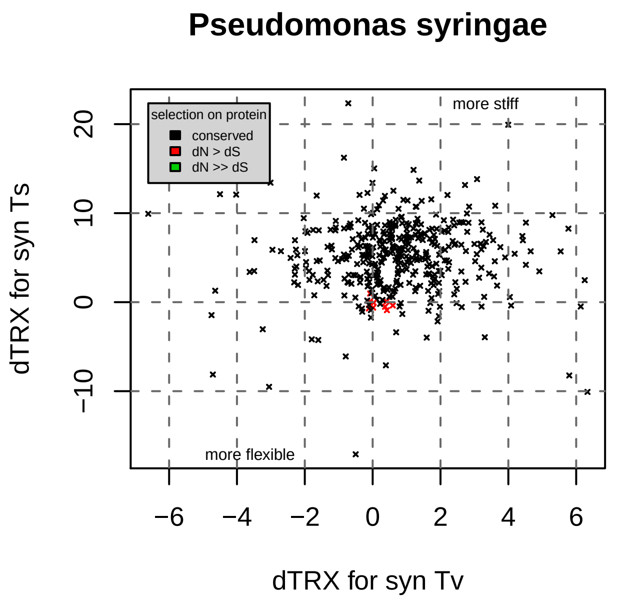
Pseudomonas syringae
selection on protein
conserved
dN > dS
dN >> dS
more stiff
more flexible
dTRX for syn Ts
dTRX for syn Tv
−10
0
10
20
−6
−4
−2
0
2
4
6

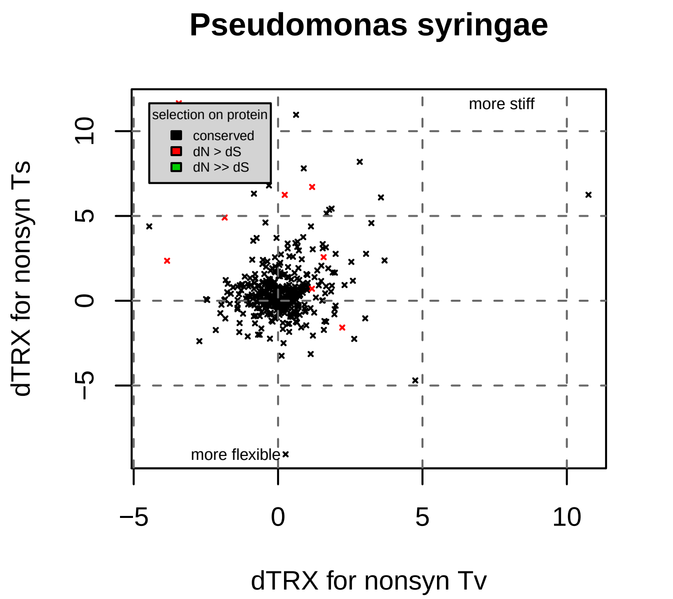
Pseudomonas syringae
selection on protein
conserved
dN > dS
dN >> dS
more stiff
more flexible
dTRX for nonsyn Ts
dTRX for nonsyn Tv
−5
0
5
10
−5
0
5
10

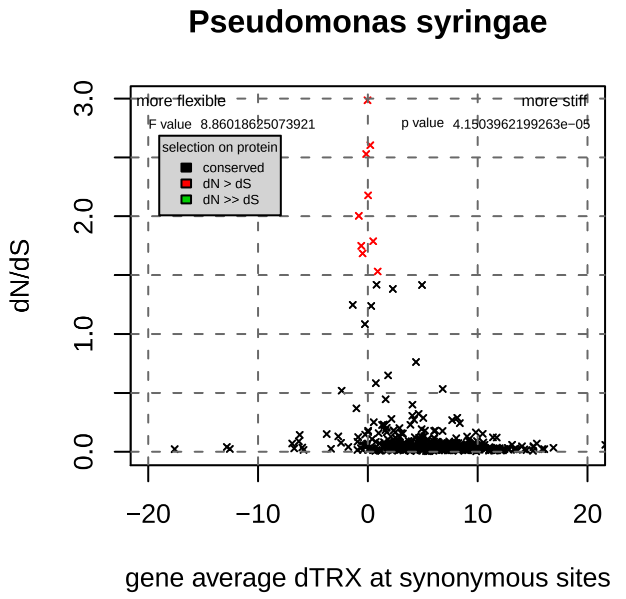
Pseudomonas syringae
more flexible
more stiff
F value 8.86018625073921
p value 4.1503962199263e−05
selection on protein
conserved
dN > dS
dN >> dS
dN/dS
gene average dTRX at synonymous sites
0.0
1.0
2.0
3.0
−20
−10
0
10
20

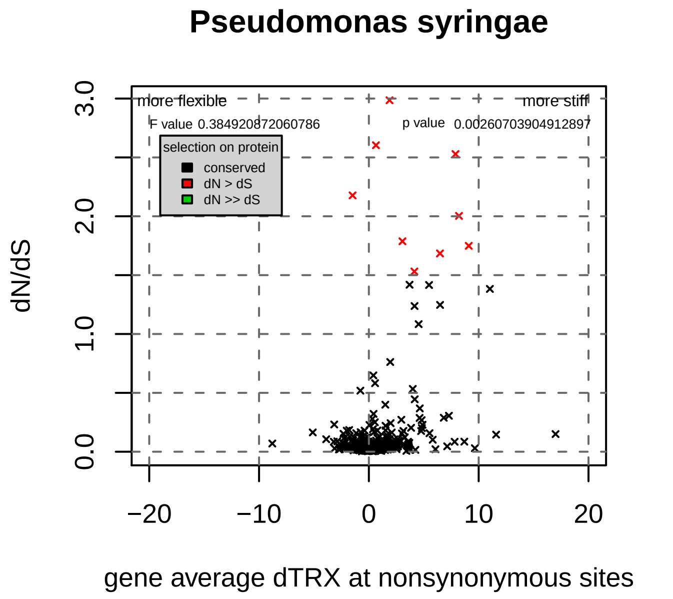
Pseudomonas syringae
more flexible
more stiff
F value 0.384920872060786
p value 0.00260703904912897
selection on protein
conserved
dN > dS
dN >> dS
dN/dS
gene average dTRX at nonsynonymous sites
0.0
1.0
2.0
3.0
−20
−10
0
10
20

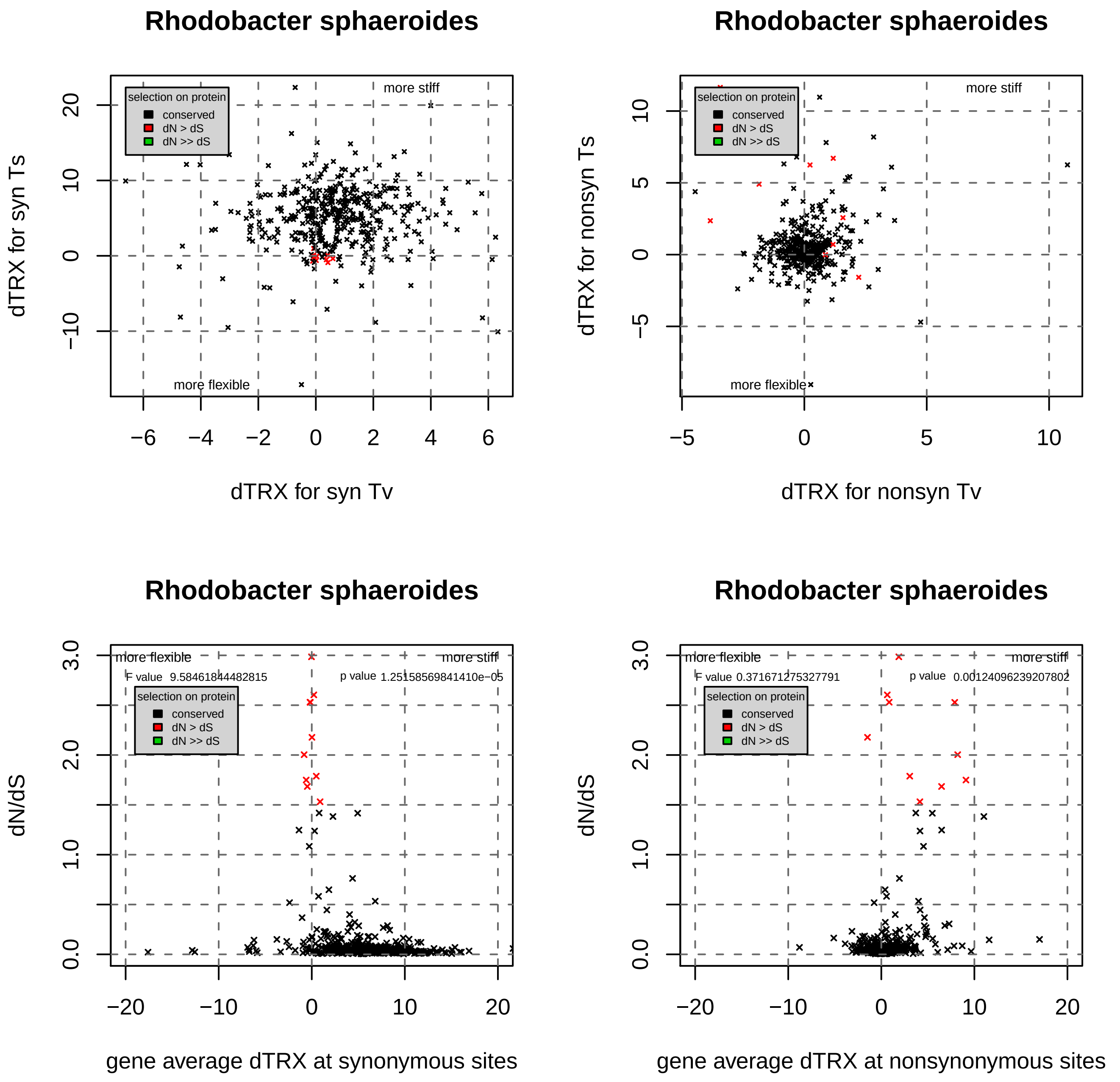
Rhodobacter sphaeroides
selection on protein
conserved
dN > dS
dN >> dS
more stiff
more flexible
dTRX for syn Ts
dTRX for syn Tv
Rhodobacter sphaeroides
selection on protein
conserved
dN > dS
dN >> dS
more stiff
more flexible
dTRX for nonsyn Ts
dTRX for nonsyn Tv
Rhodobacter sphaeroides
more flexible
more stiff
F value 9.58461844482815
p value 1.25158569841410e−05
selection on protein
conserved
dN > dS
dN >> dS
dN/dS
gene average dTRX at synonymous sites
Rhodobacter sphaeroides
more flexible
more stiff
F value 0.371671275327791
p value 0.00124096239207802
selection on protein
conserved
dN > dS
dN >> dS
dN/dS
gene average dTRX at nonsynonymous sites

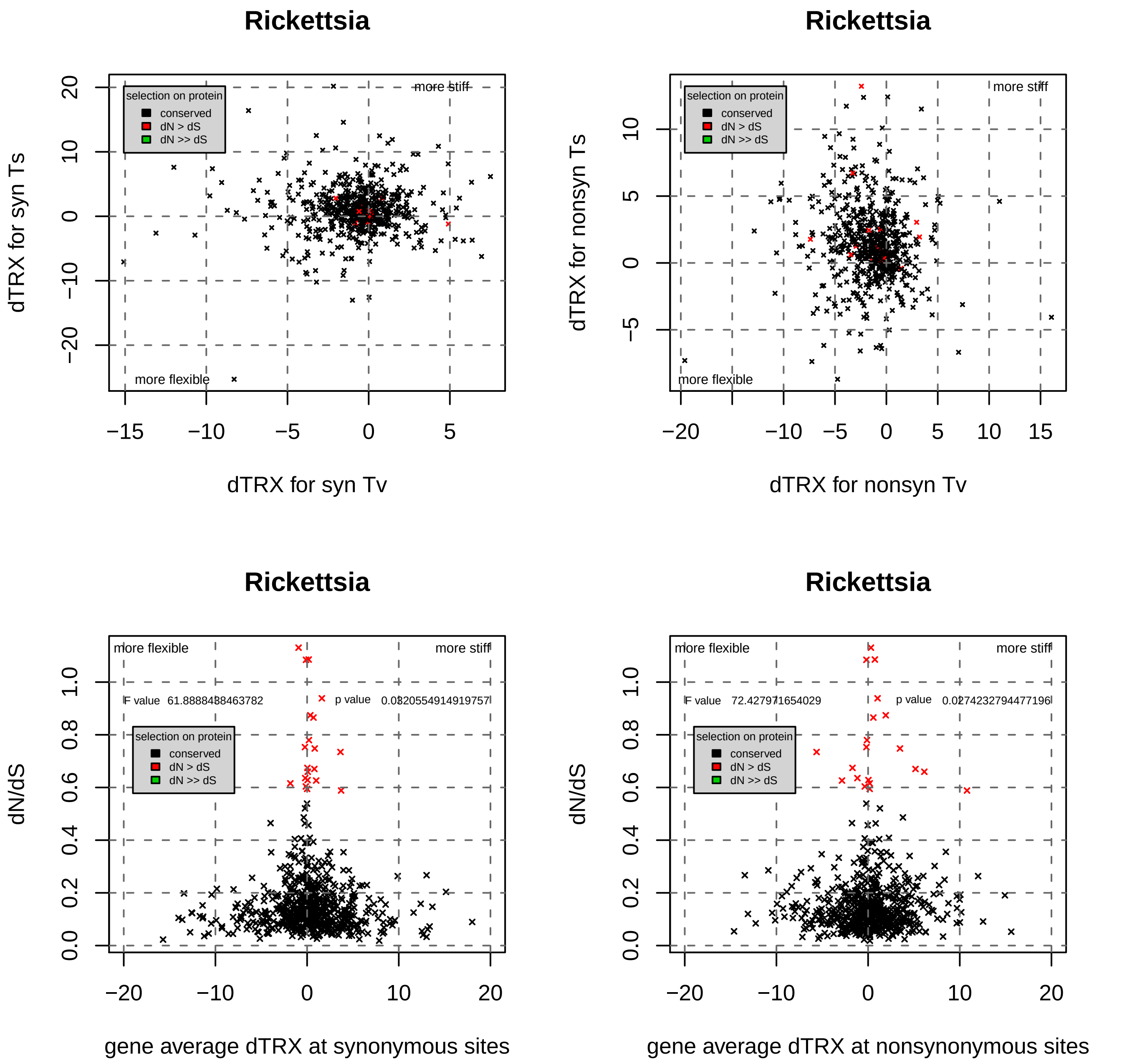

Rickettsia
Rickettsia
Rickettsia
Rickettsia
selection on protein
conserved
dN > dS
dN >> dS
more stiff
more flexible
dTRX for syn Ts
dTRX for syn Tv
dTRX for nonsyn Ts
dTRX for nonsyn Tv
F value 61.8888438463782
p value 0.0320554914919757
F value 72.427971654029
p value 0.0274232794477196
dN/dS
gene average dTRX at synonymous sites
gene average dTRX at nonsynonymous sites

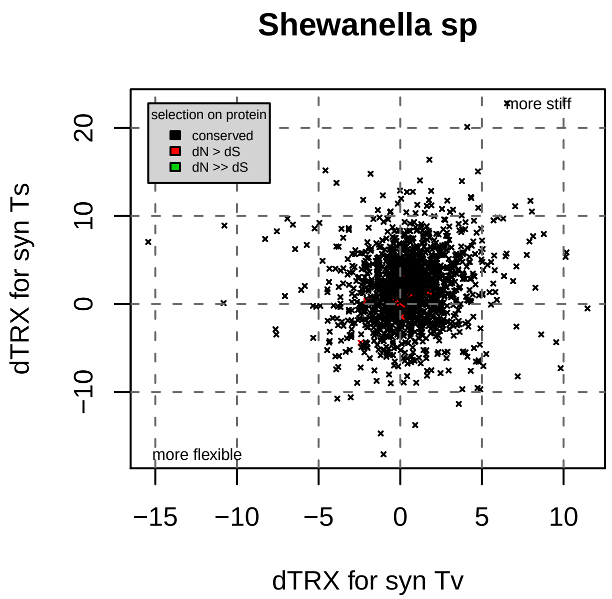

Shewanella sp
selection on protein
conserved
dN > dS
dN >> dS
more stiff
more flexible
20
10
0
−10
−15
−10
−5
0
5
10
dTRX for syn Ts
dTRX for syn Tv

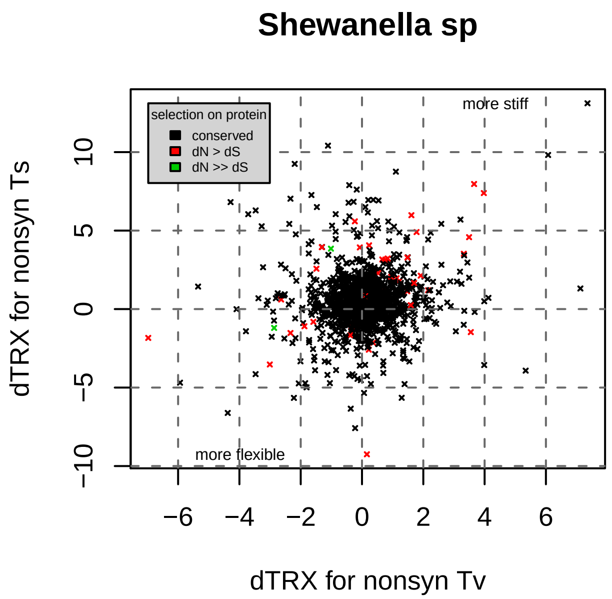

Shewanella sp
selection on protein
conserved
dN > dS
dN >> dS
more stiff
more flexible
10
5
0
−5
−10
−6
−4
−2
0
2
4
6
dTRX for nonsyn Ts
dTRX for nonsyn Tv

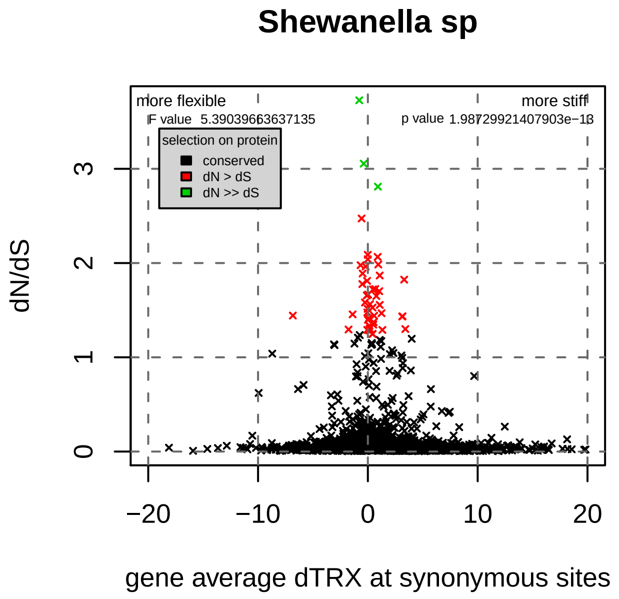

Shewanella sp
more flexible
more stiff
F value 5.39039663637135
p value 1.98729921407903e−13
selection on protein
conserved
dN > dS
dN >> dS
3
2
1
0
−20
−10
0
10
20
dN/dS
gene average dTRX at synonymous sites

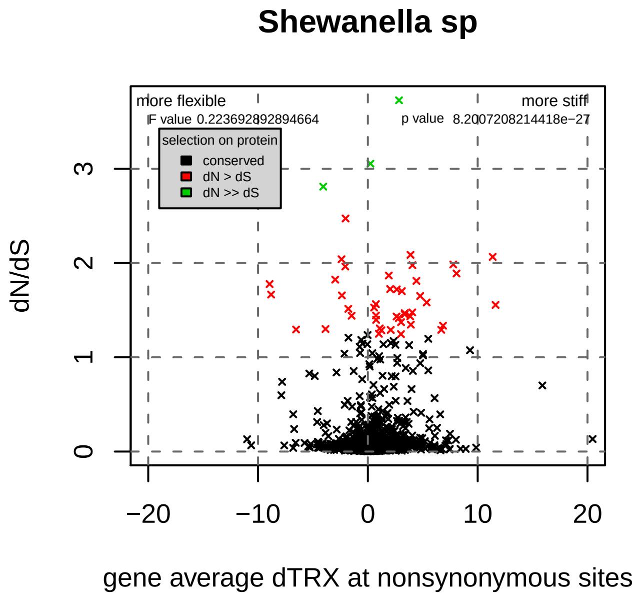

Shewanella sp
more flexible
more stiff
F value 0.223692892894664
p value 8.20072082144 18e−27
selection on protein
conserved
dN > dS
dN >> dS
3
2
1
0
−20
−10
0
10
20
dN/dS
gene average dTRX at nonsynonymous sites

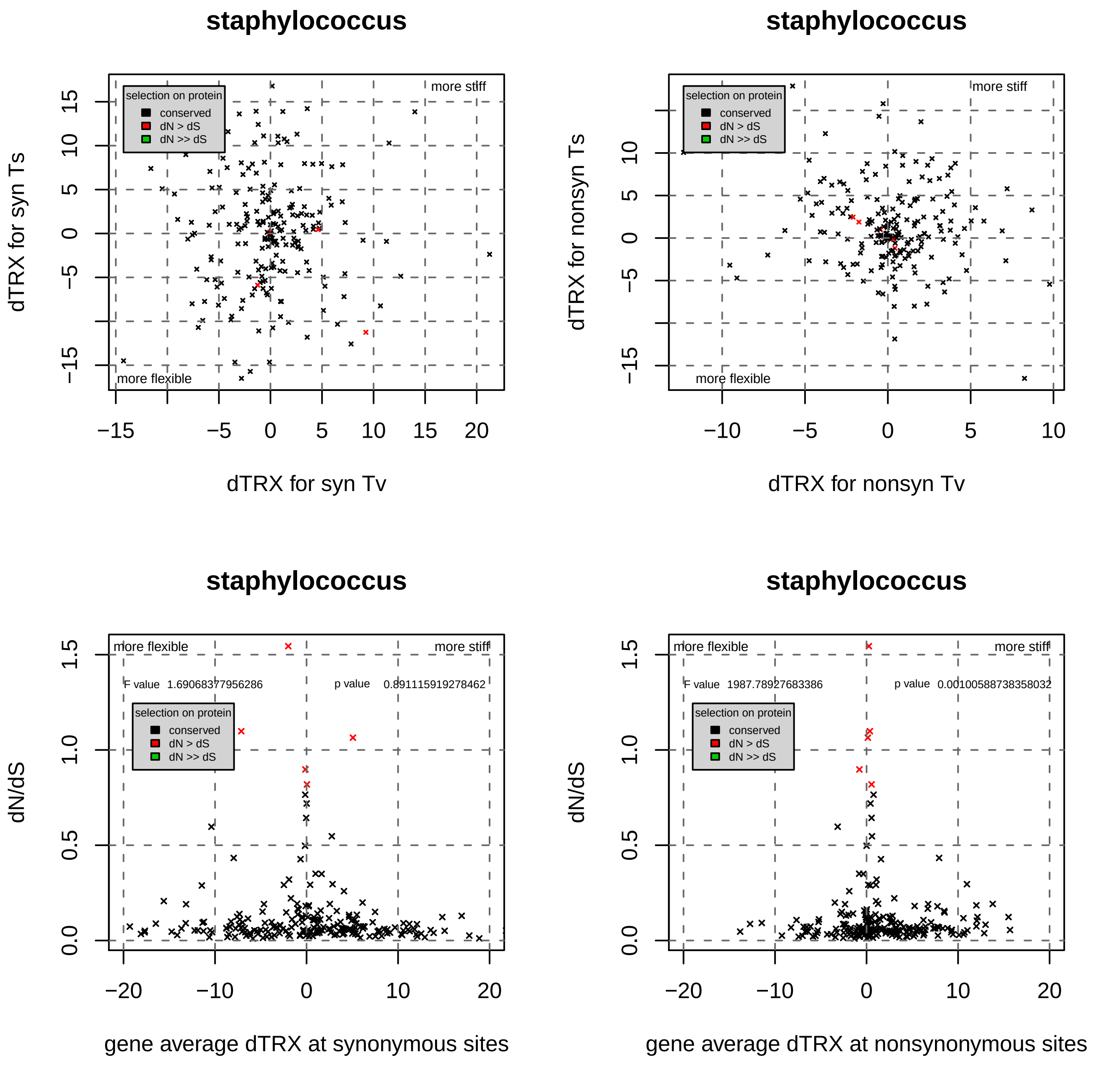

staphylococcus
selection on protein
conserved
dN > dS
dN >> dS
more stiff
more flexible
dTRX for syn Ts
dTRX for syn Tv
staphylococcus
dTRX for nonsyn Ts
dTRX for nonsyn Tv
staphylococcus
F value 1.69068377956286
p value 0.891115919278462
dN/dS
gene average dTRX at synonymous sites
staphylococcus
F value 1987.78927683386
p value 0.00100588738358032
gene average dTRX at nonsynonymous sites

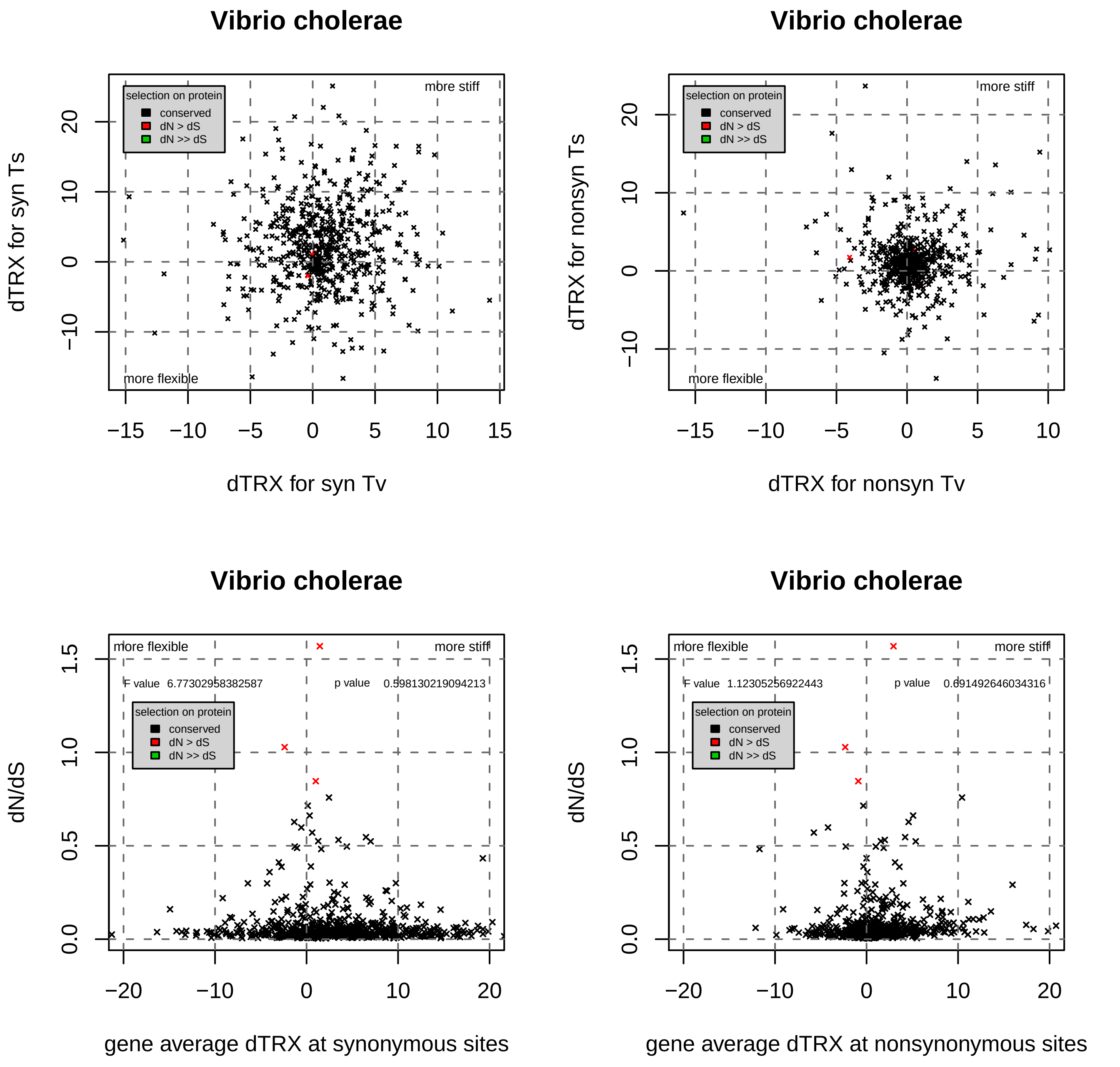
Vibrio cholerae
selection on protein
conserved
dN > dS
dN >> dS
more stiff
more flexible
dTRX for syn Ts
dTRX for syn Tv
−15
−10
−5
0
5
10
15
20
Vibrio cholerae
selection on protein
conserved
dN > dS
dN >> dS
more stiff
more flexible
dTRX for nonsyn Ts
dTRX for nonsyn Tv
Vibrio cholerae
more flexible
more stiff
F value 6.77302958382587
p value 0.598130219094213
selection on protein
conserved
dN > dS
dN >> dS
dN/dS
gene average dTRX at synonymous sites
0.0
0.5
1.0
1.5
−20
Vibrio cholerae
more flexible
more stiff
F value 1.12305256922443
p value 0.691492646034316
selection on protein
conserved
dN > dS
dN >> dS
dN/dS
gene average dTRX at nonsynonymous sites

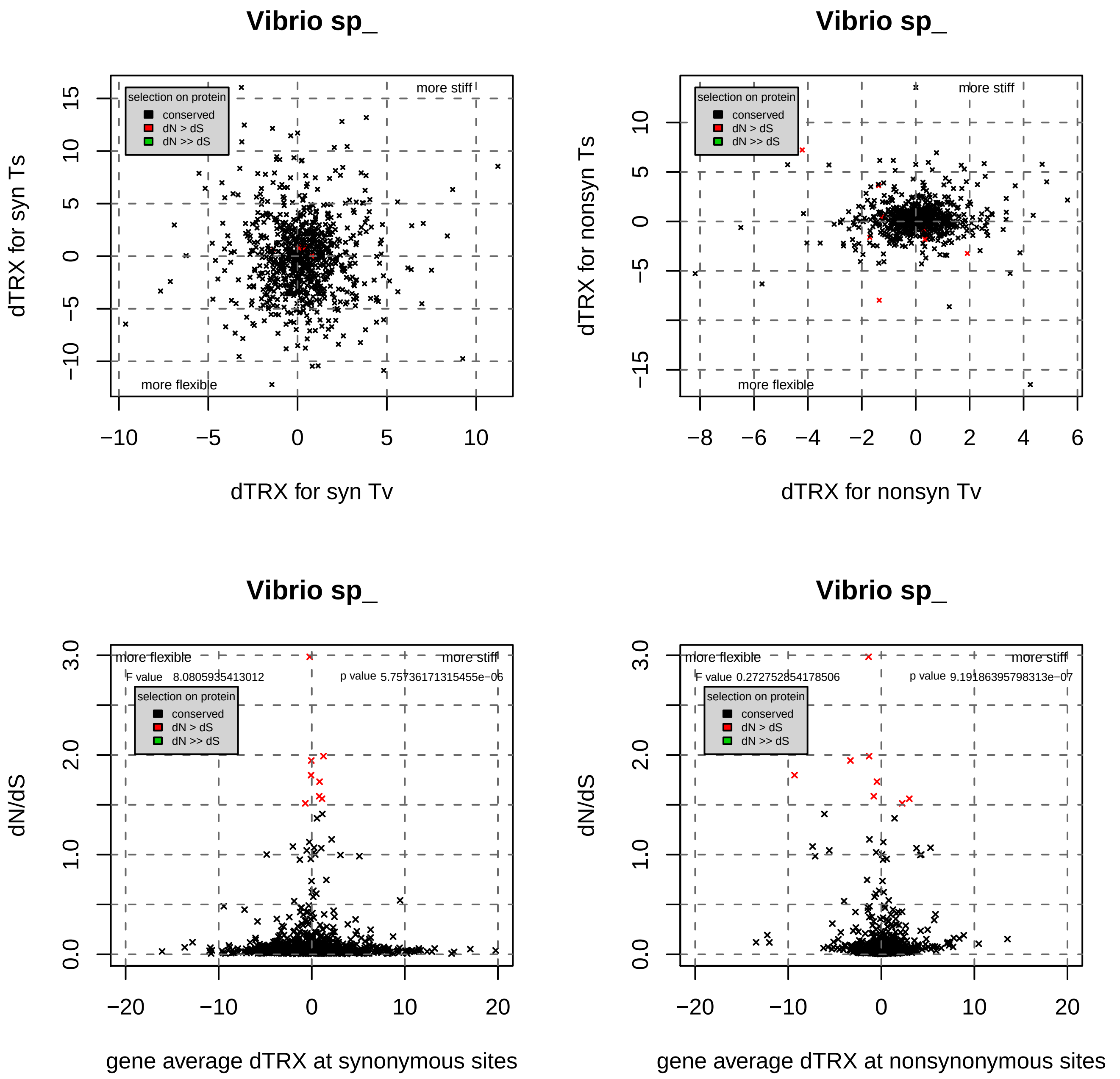
Vibrio sp_
selection on protein
conserved
dN > dS
dN >> dS
more stiff
more flexible
dTRX for syn Ts
dTRX for syn Tv
Vibrio sp_
selection on protein
conserved
dN > dS
dN >> dS
more stiff
more flexible
dTRX for nonsyn Ts
dTRX for nonsyn Tv
Vibrio sp_
more flexible
more stiff
F value 8.0805935413012
p value 5.75736171315455e−06
selection on protein
conserved
dN > dS
dN >> dS
dN/dS
gene average dTRX at synonymous sites
Vibrio sp_
more flexible
more stiff
F value 0.272752854178506
p value 9.19186395798313e−07
selection on protein
conserved
dN > dS
dN >> dS
dN/dS
gene average dTRX at nonsynonymous sites

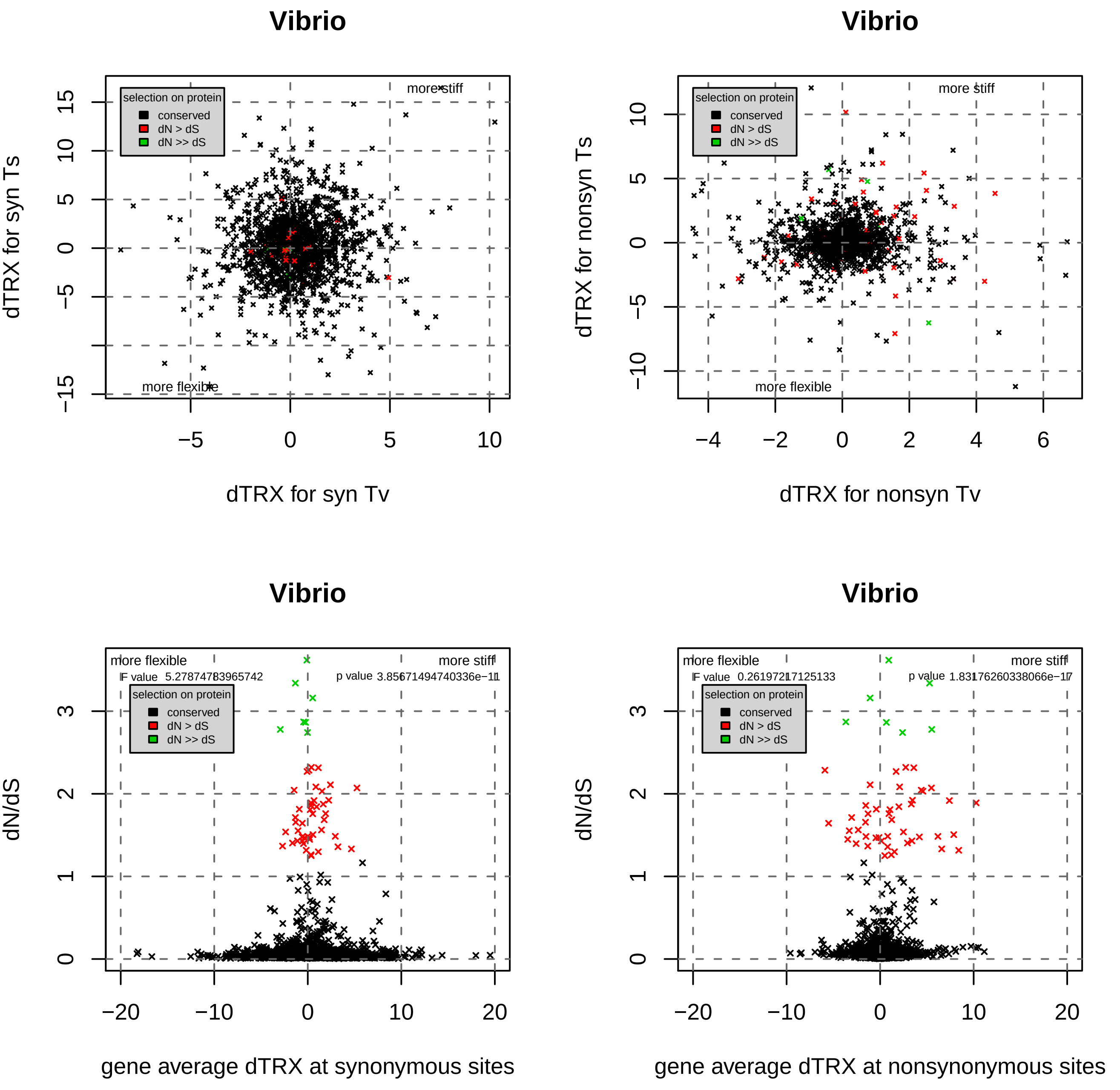
Vibrio
dTRX for syn Ts
dTRX for syn Tv
selection on protein
conserved
dN > dS
dN >> dS
more stiff
more flexible
Vibrio
dTRX for nonsyn Ts
dTRX for nonsyn Tv
selection on protein
conserved
dN > dS
dN >> dS
more stiff
more flexible
Vibrio
dN/dS
gene average dTRX at synonymous sites
more flexible
more stiff
F value 5.27874783965742
p value 3.85671494740336e−11
selection on protein
conserved
dN > dS
dN >> dS
Vibrio
dN/dS
gene average dTRX at nonsynonymous sites
more flexible
more stiff
F value 0.261972117125133
p value 1.83176260338066e−17
selection on protein
conserved
dN > dS
dN >> dS

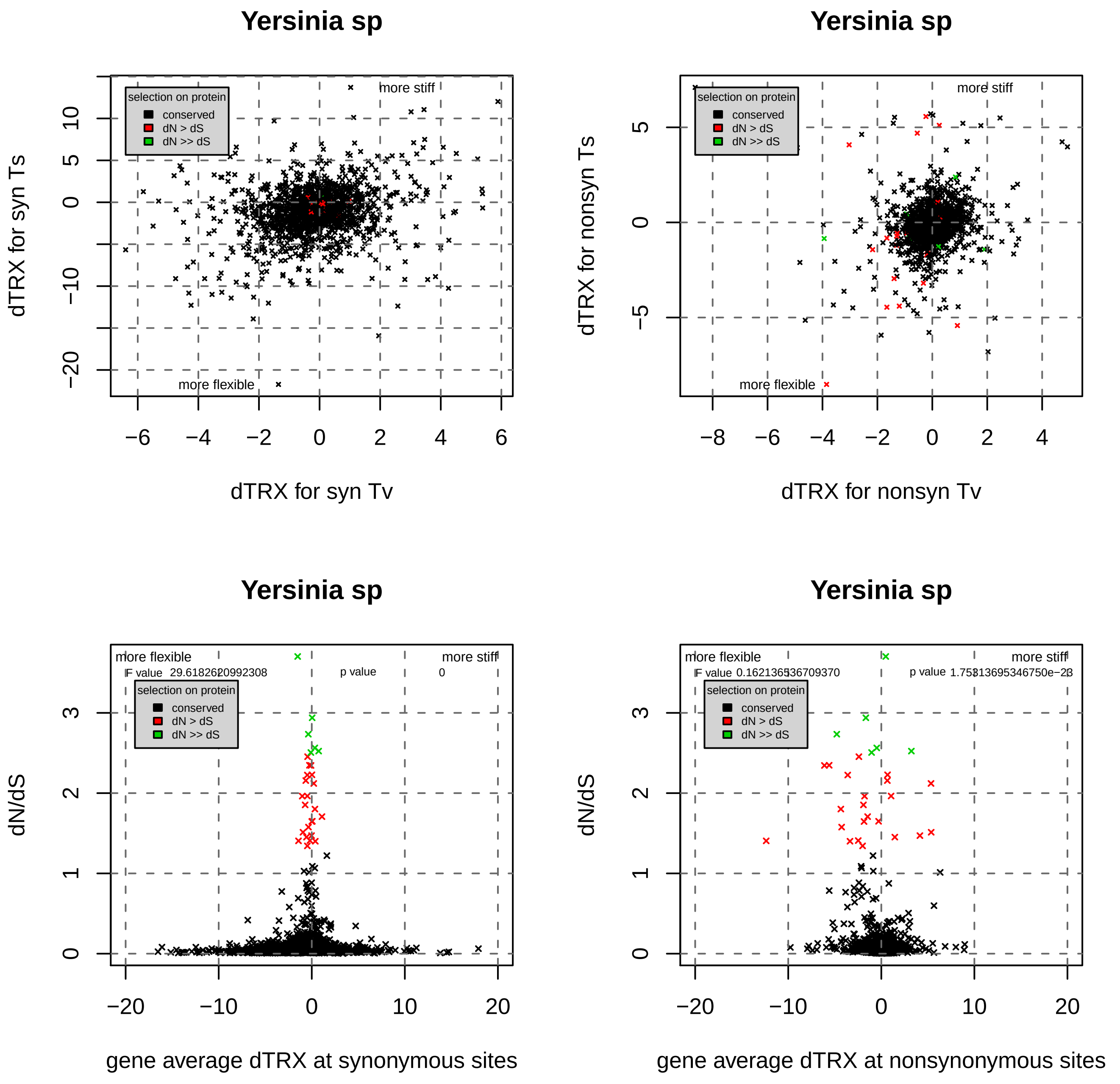
Yersinia sp
selection on protein
conserved
dN > dS
dN >> dS
more stiff
more flexible
dTRX for syn Ts
dTRX for syn Tv
Yersinia sp
selection on protein
conserved
dN > dS
dN >> dS
more stiff
more flexible
dTRX for nonsyn Ts
dTRX for nonsyn Tv
Yersinia sp
more flexible
more stiff
F value 29.61826209923O8
p value 0
selection on protein
conserved
dN > dS
dN >> dS
dN/dS
gene average dTRX at synonymous sites
Yersinia sp
more flexible
more stiff
F value 0.162136536709370
p value 1.75313695346750e−23
selection on protein
conserved
dN > dS
dN >> dS
dN/dS
gene average dTRX at nonsynonymous sites
